# Key Roles of p53 Signaling Pathway-Related Factors GADD45B and SERPINE1 in the Occurrence and Development of Gastric Cancer

**DOI:** 10.1155/2023/6368893

**Published:** 2023-08-24

**Authors:** Yaoqing Li, Liyijing Shen, Kelong Tao, Guangen Xu, Kewei Ji

**Affiliations:** ^1^Department of Gastrointestinal Surgery, Shaoxing People's Hospital, Shaoxing 312000, China; ^2^Department of Radiology, Shaoxing People's Hospital, Shaoxing 312000, China

## Abstract

p53 can function as an independent and unfavorable prognosis biomarker in cancer patients. We tried to identify the key factors of the p53 signaling pathway involved in gastric cancer (GC) occurrence and development based on the genotype-tissue expression (GTEx) and the Cancer Genome Atlas (TCGA) screening. We downloaded gene expression data and clinical data of GC included in the GTEx and TCGA databases, followed by differential analysis. Then, the key factors in the p53 signaling pathway were identified, followed by an analysis of the correlation between key factors and the prognosis of GC patients. Human GC cell lines were selected for *in vitro* cell experiments to verify the effects of key prognostic factors on the proliferation, migration, invasion, and apoptosis of GC cells. We found 4,944 significantly differentially expressed genes (DEGs), of which 2,465 were upregulated and 2,479 downregulated in GC. Then, 27 DEGs were found to be involved in the p53 signaling pathway. GADD45B and SERPINE1 genes were prognostic high-risk genes. The regression coefficients of GADD45B and SERPINE1 were positive. GADD45B was poorly expressed, while SERPINE1 was highly expressed in GC tissues, highlighting their prognostic role in GC. The *in vitro* cell experiments confirmed that overexpression of GADD45B or silencing of SERPINE1 could inhibit the proliferation, migration, and invasion and augment the apoptosis of GC cells. Collectively, the p53 signaling pathway-related factors GADD45B and SERPINE1 may be key genes that participate in the development of GC.

## 1. Introduction

Gastric cancer (GC) is a prevalent malignancy in the digestive system [1] and is a molecularly and phenotypically heterogeneous disorder [2]. Despite medical progress, the prognosis of patients with GC is poor, with a low 5-year survival rate [3]. Tumor-targeted gene therapy has been considered an effective way to control the proliferation of GC cells [4]. Furthermore, it has been reported that molecular prognostic markers can be related to the progression of cancers and tumors, and detection and analysis of these markers can dynamically reflect the prognosis of patients [5]. In this context, it is important to understand key genes involved in GC development.

Carcinogenesis is a multistep process, which can occur in the conditions of ectopic expression of oncogenes and depletion of tumor suppressor genes [6]. Increasing evidence shows that epigenetic silencing of tumor suppressor genes is essential for tumorigenesis and metastasis [7]. GC is reported to be partly attributed to multiple genetic and epigenetic changes that lead to the overexpression of oncogenes and the loss of tumor suppressor genes [8]. Therefore, bioinformatics is increasingly important in cancer research, including GC [9]. In previous studies, the genotype-tissue expression (GTEx) and the Cancer Genome Atlas (TCGA) databases were employed to comprehensively analyze the expression of normal tissues, tumor tissues, and cell lines in GC [10]. p53 expression is dysregulated in GC, and the p53 signaling pathway is involved in the development of GC [11]. p53 is an important tumor suppressor gene. Its main function is to control cell cycle arrest, DNA repair, and cell metabolism as a transcription activator by identifying and binding specific DNA sequences [12]. Intriguingly, existing research has also reported that highly expressed p53 in GC is associated with poor prognosis and overall survival in the entire population [13]. Of note, our GTEx and TCGA databases-based analysis identified the p53 signaling pathway-related factors GADD45B and SERPINE1 as the key genes that might participate in the development of GC. It was previously revealed that the methylation frequency of GADD45G was increased in GC tissues [14]. Moreover, SERPINE1 could serve as a prognostic marker in GC [15]. In the current experiment, we aimed to explore the function of GADD45B and SERPINE1 as possibly key factors mediated by the p53 signaling pathway in GC based on GTEx and TCGA databases.

## 2. Methods

### 2.1. Microarray Data

Gene expression profiles of patients with GC were downloaded from TCGA (https://www.cancer.gov), including 32 adjacent normal tissue samples and 375 GC tissue samples, and the downloaded data format was Fragments Per Kilobase of exon model per Million mapped fragments data. In addition, due to the small number of cancer samples in the TCGA database, we downloaded 209 healthy human gastric tissue samples from the GTEx database (https://www.GTEXportal.org/). Finally, the “Limma” software package was used to standardize the gene expression data, and the processed TCGA data and GTEx data were merged for differential analysis.

### 2.2. Differential Gene Analysis

Differential analysis was performed on gene expression data from GC tissue samples in TCGA and paired normal tissue samples in both GTEX and TCGA databases using the “Limma” package in R. The screening criteria for differentially expressed genes (DEGs) were set to |logFC| >1 and *p*-value < 0.05. A heatmap of the DEGs was generated using the “pheatmap” package in R. Race information was extracted from the TCGA_STAD dataset, and samples were grouped into Asian and White categories. Statistical analysis was performed using the “stats” package in R, and the results were visualized using the “ggplot2” package. The statistical method used was *t*-test.

### 2.3. Gene Set Enrichment Analysis (GSEA)

GSEA is a calculation method used to evaluate whether a priority-defined gene set shows a statistically significant and consistent difference between two biological states. In this study, GSEA first generated an ordered gene list based on the expression matrix of all genes, and a significant difference in survival between the high-expression group and the low-expression group was analyzed by GSEA. The “clusterprofiler” package in R software was used for GSEA analysis; the “species” was set at “*Homo sapiens*”, and the reference gene set was c2 cp.v7.2. symbols. gmt [Curated], and the gene set database was MSigDB Collections. False discovery rate <0.25 and *p*.adjust < 0.05 indicated significant enrichment, and the *p*-value correction method was Benjamini-Hochberg. The number of genes contained in each gene was set to 10–500.

### 2.4. Gene-Based Correlation and Interaction Analyses

The “Pearson” correlation coefficient of key factors was calculated by the R language package “Corrplot,” The correlation among key factors was obtained. Next, we obtained the interaction relationship among key factors through the STRING website (https://string-db.org/), and Cytoscape 3.5.1 software was utilized to visualize the gene interaction network.

### 2.5. Principal Component Analysis (PCA)

We extracted the expression matrix of p53 signaling pathway-related genes in the samples of GC patients from the TCGA database, followed by classification through the software package “Consensus Clusterplus” according to the expression of the pathway-related genes. Based on the classification results, PCA was performed on the DEGs in the GC tissue samples utilizing R package version 3.6.0 to evaluate the expression patterns of DEGs.

### 2.6. Lasso and Multivariate Cox Regression Analyses

The key factors of the p53 signaling pathway were selected. According to the survival information of GC patients in the TCGA database, univariate Cox analysis was performed using the R language package “Survival”. A forest map was constructed using the software package “Forestplot” to observe the survival risk of genes. Hazard ratio (HR) > 1 was a screening criterion for high-risk genes, and log-rank *p* < 0.05 was considered statistical significance. Next, we used the software package “GLMnet” in R language to conduct a Lasso regression analysis to identify the expression level of each gene. When the risk score value of a given sample was less than the average risk score of all samples, the latter was considered as a low-risk sample; otherwise, it was considered as a high-risk sample. The number of candidate genes with the smallest error and the corresponding log (*λ*) value were obtained. “Glmnet” software package was used to construct Lasso regression or elastic network regularization path, logistic and polynomial regression model, Poisson regression, and Cox model in linear regression.

### 2.7. Survival Analysis and Receiver Operating Characteristic (ROC) Analysis

The Kaplan–Meier method was used to estimate the survival curves of the high-risk and low-risk groups. In addition, ROC curves were constructed based on the expression levels and disease status of the samples in both the high-risk and low-risk groups using the R language pROC package (https://cran.r-project.org/web/packages/pROC/index.html). Finally, the ROC curve and the area under the ROC curve (AUC value) were used to evaluate the sensitivity and specificity of the survival analysis model. It is generally believed that when AUC > 0.5, the survival curve showed good predictive performance.

### 2.8. Correlation Analysis of Prognosis

According to the key factors enriched by the p53 pathway, combined with the clinical information of the key factors in TCGA GC patients, we used the R language “heatmap” software package to calculate the correlation between classification and clinical information according to the classification (clusters 1 and 2) and drew the correlation heatmap. We preliminarily explored the influence of key factors on the prognosis of GC patients according to the correlation of each attribute of samples screened by Lasso regression with high-risk genes as key factors and genes with clinical grouping.

### 2.9. Univariate- and Multivariate-Independent Prognostic Analyses

According to the risk score of samples predicted by Lasso regression, clinical information and survival of GC patients in the TCGA database were integrated. Univariate-independent prognostic analysis was conducted using the R language package “Survival” to the preliminary evaluation of the relationship between clinical attributes with prognosis and survival. The relationship between clinical attributes and prognostic survival could be more accurately measured through multivariate-independent prognostic analysis. The “ForestPlot” software package was used to plot the forest plots to observe the survival risk of clinical attributes. HR > 1 was set as the screening criteria for attributes. Log-rank *p* < 0.05 was regarded to be statistical significance.

### 2.10. Cell Culture

Human GC human gastric adenocarcinoma cell line (AGS) cells (CRL-1739) purchased from Cobioer (Nanjing, China) were cultured with Dulbecco's Modified Eagle's Medium (DMEM) (11965092, Gibco, Grand Island, NY) containing 10% fetal bovine serum (FBS) (16140071, Gibco) and 1% penicillin–streptomycin (15140148, Gibco), and then transferred to an incubator at 37°C with 5% CO_2_.

### 2.11. Plasmid Construction and Cell Transfection

AGS cells in the logarithmic growth phase (4 × 10^5^ cells/well) were seeded into a six-well cell culture plate. When the cell confluency reached 70%–80%, the cells were transfected according to the instructions of Lipofectamine 2000 (11668019, Invitrogen, Carlsbad, CA). The plasmids (2 *µ*g for each) carrying GADD45B overexpression (or-GADD45 B) and SERPINE1 knockdown (short hairpin RNA (sh)-SERPINE1), and their negative controls (NCs) were diluted with 250 *μ*L serum-free medium Opti-MEM (the final concentration added to the cells was 50 nM) and fully mixed with 5 *μ*L Lipofectamine 2,000 diluted with 250 *μ*L serum-free medium Opti-MEM. The mixture was allowed to rest for 20 min and then added to a six-well plate. After transfection, the cells were cultured at 37°C with 5% CO_2_ and saturated humidity. After 4–6 hr, the transfection fluid medium was discarded, and DMEM containing 10% FBS was added for further culturing. After 48 hr, the cells were used for subsequent experimentation.

### 2.12. Colony Formation Assay

The transfected cells in the logarithmic growth phase were detached with 0.25% trypsin and gently pipetted into a single-cell suspension. The detachment was terminated with DMEM containing 10% FBS after centrifugation. Next, the cell suspension was diluted and seeded in 10 mL of 37°C preheated culture medium at a gradient density of 200 cells in each group. It was followed by culture in a 37°C incubator with 5% CO_2_ for 1 week, during which the solution was renewed every 2 days. The culture was terminated when a visible colony was formed at the bottom. Next, the cells were fixed with 2 mL of methanol for 20 min, then with 2 mL of Giemsa staining solution (G4640, Solarbio, Beijing, China) for 40 min. Finally, the effective clones with >10 cells were counted under an inverted microscope (IX-50, Olympus, Tokyo, Japan).

### 2.13. Transwell Assay

*In vitro* cell invasion was detected in a 24-well plate using transwell chambers (3422, Corning Glass Works, Corning, NY). The transwell chambers were covered with Matrigel in advance, and 600 mL DMEM containing 20% FBS was added to the basolateral chamber in advance. The cells transfected for 48 hr were resuspended in FBS-free DMEM, and 1 × 10^6^/mL cells were seeded into the apical chamber for 24 hr of culture at 37°C with 5% CO_2_. The transwell chambers were taken out and fixed with 5% glutaraldehyde at 4°C, followed by 0.1% crystal violet staining for 5 min. The cells on the surface were wiped off with cotton balls; other cells were observed under an inverted fluorescence microscope (TE2000, Nikon, Tokyo, Japan), with five visual fields randomly selected and photographed. The average value was the number of cells passing through the chamber.

### 2.14. Scratch Test

The cells of each group were incubated in an incubator at 37°C with 5% CO_2_ for 24 hr, and then a pipette tip was used to make transverse scratches on the monolayer cells. The serum-free medium was added to continue the culture. The cell migration at 0 and 48 hr was observed under the inverted microscope. Three locations of each cell group were selected to take photos, which were analyzed using Image J software. Next, 6–8 horizontal lines were drawn randomly, and the average distance between cells was calculated. Scratch distance (%) = [scratch distance at 48 hr/scratch distance at 0 hr (the distance photographed and calculated immediately after making the scratch)] × 100%.

### 2.15. Flow Cytometry

Cells were collected in a flow tube and centrifuged, followed by removing the supernatant. According to the instructions of the Annexin-V-fluorescein isothiocyanate (FITC) cell apoptosis detection kit (C1062S, Beyotime, Shanghai, China), Annexin-V-FITC, propidium iodide (PI), and N-2-hydroxyethylpiperazine-N-ethane-sulfonic acid (HEPES) buffer solutions were prepared into Annexin-V-FITC/PI dye solution in the ratio of 1 : 2: 50. Every 100 *μ*L dye was used to resuspend 1 × 10^6^ cells. After incubation at room temperature for 15 min, the cells were incubated with 1 mL HEPES buffer solution at room temperature for 15 min. The 525 and 620 nm band-pass filters were excited at 488 nm wavelength to detect FITC and PI fluorescence for cell apoptosis detection.

### 2.16. Real-Time-Quantitative Polymerase Chain Reaction (RT-qPCR)

Cells of each group were collected and Trizol (15596018, Invitrogen) kits were used to extract total RNA. cDNA was obtained by reverse transcription using reverse transcription kits (RR047A, Takara, Dalian, China). Following the instructions of TB Green® Premix Ex Taq ™ Kit (RR420A, Takara), fluorescent qPCR was performed. The samples were subjected to RT-qPCR reaction in an ABI 7500 real-time fluorescent qPCR instrument. Glyceraldehyde-3-phosphate dehydrogenase (GAPDH) served as an internal parameter. Takara synthesized the primers used in this study (*Supplementary 1*).

### 2.17. Western Blot Assay

The cells of each group were collected, after which the total protein was extracted with Radioimmunoprecipitation assay lysate (P1300B, Beyotime), and the protein concentration was determined with BCA kits (P0012S, Beyotime). After protein separation by polyacrylamide gel electrophoresis, the protein was transferred to a PVDF membrane by wet or semidry transfer method and sealed with 5% skimmed milk powder at room temperature for 30 min. Next, the PVDF membrane was incubated with corresponding primary antibodies against Bax (ab32503, 1 : 1000, Abcam, Cambridge, UK), Bcl-2 (rabbit antibody, ab182858, 1 : 2000, Abcam), and GAPDH (rabbit antibody, ab181603, 1 : 10000, Abcam) at 4°C overnight. Horseradish peroxidase-labeled secondary antibody against IgG (ab6721, 1 : 1,000, Abcam) was used to incubate the samples at room temperature for 2 hr. Enhanced chemiluminescence fluorescence detection kits (32209, Thermo Fisher Scientific, Rockford, IL) were used for development. Photos were taken with a Biorad image analysis system (Bio-Rad Laboratories, Hercules, CA) and analyzed with Quantity One v4.6.2 software. The relative protein content was expressed by the gray value of the corresponding protein band to that of the GAPDH protein band.

### 2.18. Statistical Analysis

All data were analyzed using SPSS 21.0 (IBM, Armonk, NY, USA) statistical software. Measurement data were expressed as mean ± standard deviation. Comparisons of data between the two groups were performed using unpaired *t*-tests. Data comparison among multiple groups were performed by one-way analysis of variance (ANOVA), followed by Tukey's post hoc tests. *p* < 0.05 indicated a significant difference.

## 3. Results

### 3.1. 2,465 Genes with Upregulated Expression and 2,479 Genes with Downregulated Expression Are Obtained by Differential Analysis

**Figure 1:**
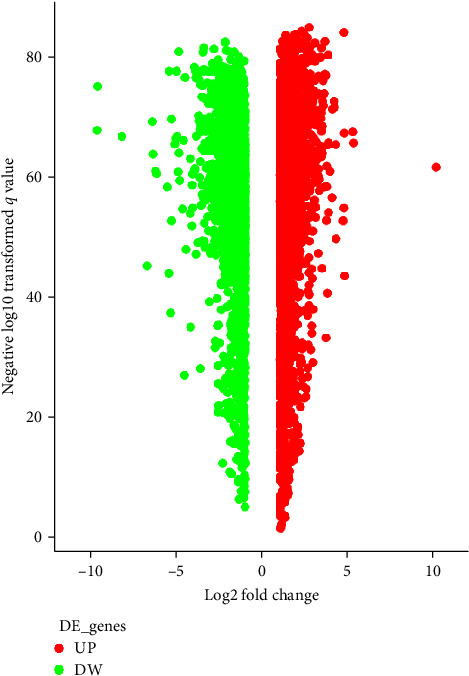
Volcanic map displaying differential gene analysis of GC-related microarray from TCGA and GTEx databases. The abscissa represents the log2FC value, and the ordinate represents -log10 *p*-value; the red dots represent upregulated genes, and the green dots represent downregulated genes.

Initially, 407 samples of GC patients were downloaded, including 32 adjacent normal tissue samples and 375 GC tissue samples, through the TCGA database. Then, 209 healthy human gastric tissue samples were downloaded from the GTEx database. Finally, the “Limma” software package standardized the gene expression data. The processed TCGA and GTEx data were combined, yielding 206 adjacent normal tissue samples and 375 GC tissue samples (*Supplementary 1*). Additionally, we obtained 4,944 DEGs through differential analysis of the combined gene expression data (*Supplementary 1*). Among them, 2,465 genes were upregulated, and 2,479 were downregulated (Figure 1).

### 3.2. 27 Genes in the p53 Signaling Pathway may be Related to the Occurrence and Development of GC Revealed by GSEA

**Figure 2:**
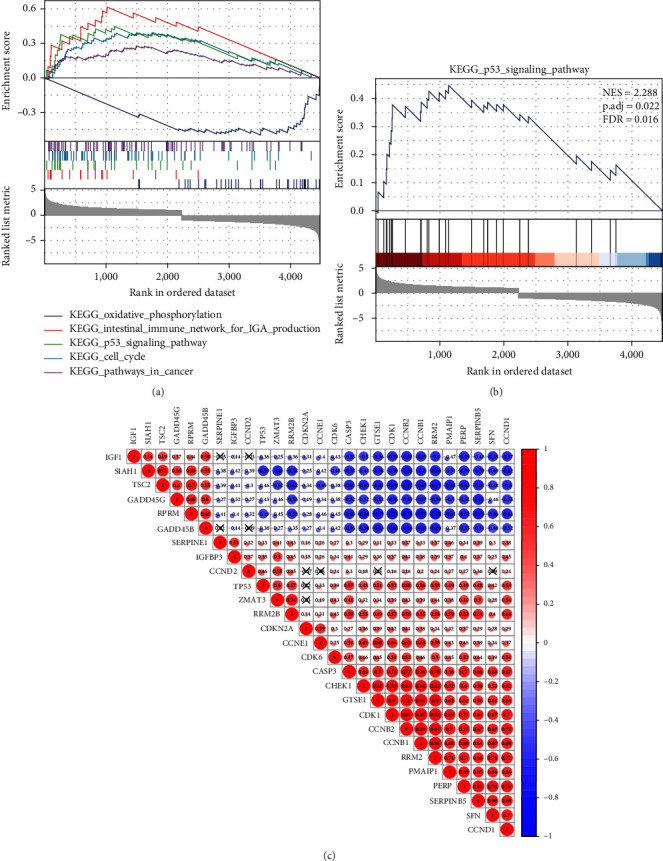
The main molecular biological functions of 4,944 differential genes analyzed by GSEA. (a) GSEA analysis results of KEGG signaling pathways such as oxidative phosphorylation, the intestinal immune network produced by IGA, p53 signal pathway, cell cycle, and cancer pathway. The ordinate represents an enrichment score. (b) GSEA analysis results of p53 signal pathway. NES in the upper right corner represents the normalized enrichment score, p.adj represents the corrected *p*-value, and FDR represents the false discovery rate. (c) Correlation analysis diagram of 27 DEGs, with X indicating significance less than 95% (*p* > 0.05).

To analyze the signaling pathways involved in DEGs in GC, GSEA was applied, and the results of GSEA showed that the number of phenotypic tumor marker genes was 2,465 (49.9%), and the number of phenotypic normal marker genes was 2,479 (50.1%) among the 4,944 DEGs involved in enrichment analysis. GSEA clarified that the genes were mainly enriched in KEGG signaling pathways such as oxidative phosphorylation, the intestinal immune network produced by IGA, the p53 signaling pathway, the cell cycle, and the cancer pathway (Figure 2(a)). As displayed in Figure 2(b), the overall genes involved in the p53 signaling pathway were downregulated in GC. The “Pearson” correlation coefficient among 27 genes was further calculated through the R language package “Corrplot”, and the correlation analysis diagram among genes was obtained (Figure 2(c)). The above results displayed that there were 27 genes in the p53 signaling pathway that may be related to the occurrence and development of GC.

### 3.3. 27 Candidate Genes of the p53 Signaling Pathway Can Divide GC Patients into Two Subtypes Observed by PCA

**Figure 3:**
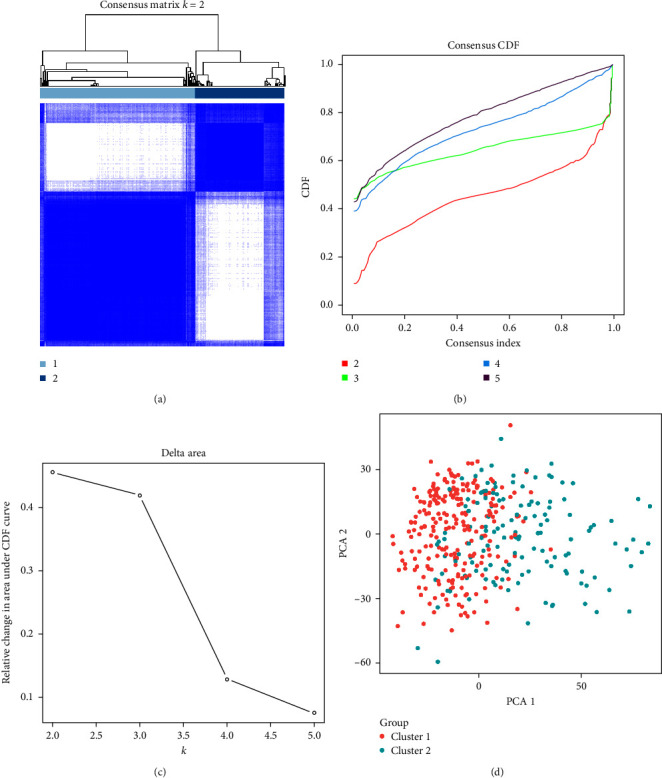
The expression matrix of 27 candidate genes in the p53 signaling pathway in GC tissue samples was classified. (a) The clustering situation of the samples when *k* = 2. (b) Changes of CDF curve when *k* = 2–5. (c) Relative change of area under CDF curve when *k* = 2–5. (d) PCA analysis of differential gene expression profiles. Red dots represent cluster 1, and green dots represent cluster 2.

Classification of the expression matrix of 27 candidate genes of a p53 signaling pathway in the samples of GC patients in the TCGA database had the best effect when *k* = 2 when the typing result was clusters 1 and 2 (Figure 3(a)). After *k* = 3, the coefficient dropped sharply, so it was more appropriate to classify into two or three subtypes, while the classification results of four subtypes and above were relatively poor, and there was no obvious boundary between the subtypes (Figures 3(b) and 3(c)). PCA was performed on the DEGs of GC tissue samples based on the classification results, and the results showed that the classification effect was good when *k* = 2 (Figure 3(d)). The above results of PCA revealed that the 27 candidate genes of the p53 signaling pathway could divide GC patients into two subtypes.

### 3.4. GADD45B and SERPINE1 Genes may be Key Genes Affecting the Prognosis of GC Patients

**Figure 4:**
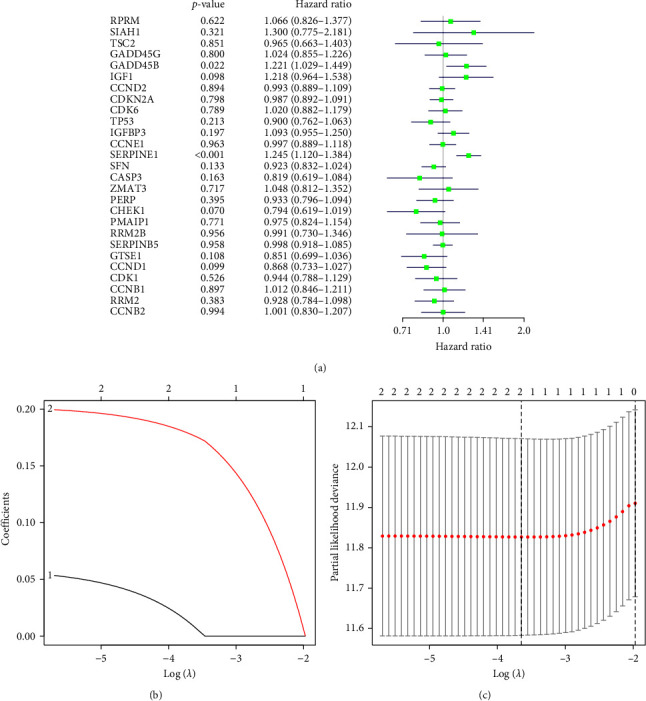
Correlation between 27 candidate genes in the p53 signaling pathway and prognosis in patients with GC analyzed by Lasso and multivariate cox regression. (a) In the figure, the name of the DEG is on the left, and the *p*-value is in the middle. The risk rate distribution of the genes is shown on the right side, where the left part indicates low risk and the right part indicates high risk. The HR indicates the risk rate; the risk rate greater than 1 indicates high risk, and less than 1 indicates low risk. (b) The abscissa represents the log (*λ*) value, the ordinate represents the coefficients value, the colored lines represent the genes used for the calculation, and the upper horizontal axis represents the number of genes. (c) The abscissa represents log (*λ*) value, and the ordinate represents partial likelihood deviance. The upper part is the corresponding log (*λ*) value, which can be used to calculate the number of genes retained. The dotted line shows partial likelihood deviance, the corresponding log (*λ*) value, and the number of genes retained in the optimal condition.

Univariate Cox analysis of key factors in the p53 signaling pathway showed that GADD45B and SERPINE1 were high-risk genes (Figure 4(a)). Furthermore, Lasso regression analysis showed that the error value gradually decreased with the reduction of the number of genes. Finally, the number of candidate genes with the smallest error was 2, and the corresponding log Lambda value was −2 (Figures 4(b) and 4(c)). Lasso and multivariate Cox regression analyses presented that GADD45B and SERPINE1 were high-risk genes with a positive regression coefficient, which may be the key genes affecting the prognosis of GC patients.

### 3.5. A Prognostic Risk Model Based on GADD45B and SERPINE1 Genes Can Accurately Predict the Prognosis of GC Patients

**Figure 5:**
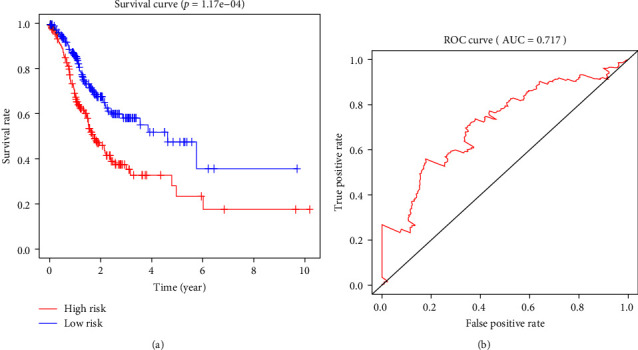
The accuracy of the prognostic risk model was evaluated using Kaplan–Meier survival curve analysis and ROC analysis. (a) Survival analysis of patients at risk. The figure above is the survival curve, with the abscissa denoting survival time, the ordinate survival rate, the blue denoting the low-risk group, and the high-risk red group. *p* = 1.17e−04. (b) The correlation between the established risk model and survival was analyzed by ROC, and the AUC value was 0.717.

The survival time, survival status, and risk value data of patients with GC were further extracted. A prognostic risk model was constructed based on GADD45B and SERPINE1, and the Kaplan–Meier method was used to estimate the survival curves of the high-risk and low-risk groups (Figure 5(a)). The results showed that the overall survival rate of GC patients in the high-risk group was significantly lower than that in the low-risk group, demonstrating the reliability of the prognostic risk model. In addition, the ROC curve was used to evaluate the sensitivity and specificity of the survival analysis model, and the results observed that the AUC value was 0.717, indicating that the prognostic risk model based on GADD45B and SERPINE1 had good accuracy, which could predict the prognosis of GC patients more accurately (Figure 5(b)).

### 3.6. GADD45B and SERPINE1 may be Important Molecular Markers for Predicting the Prognosis of Patients with GC

**Figure 6:**
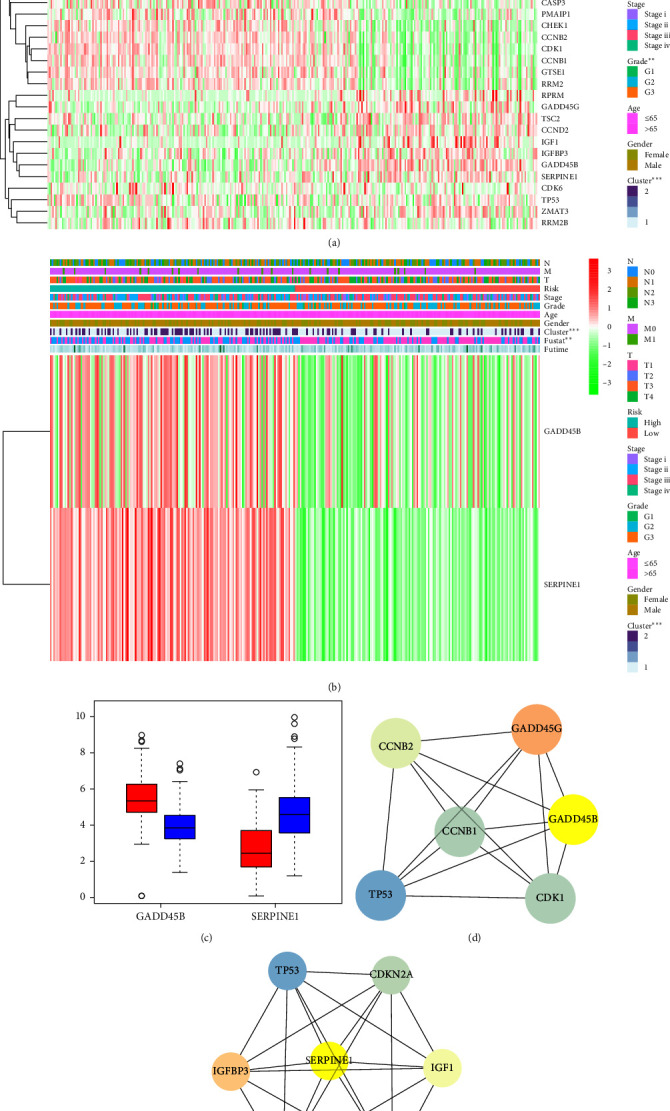
Correlation analysis of GADD45B and SERPINE1 with prognosis in patients with GC. (a) Expression heatmap of p53 signaling pathway-related genes in association with clinicopathological characteristics of GC patients. (b) Heatmap of expression of high-risk genes GADD45B and SERPINE1 in association with prognosis in patients with GC. (c) Box plot of expression of high-risk genes GADD45B and SERPINE1 in GC samples (red indicates adjacent normal tissue samples, and blue indicates GC tissue samples). (d) Network diagram of the interaction between GADD45B gene and other DEGs. (e) A network diagram of the interaction between the SERPINE1 gene and other DEGs.

According to the key factors enriched by the p53 pathway, combined with the clinical information of the key factors in TCGA GC patients, we obtained two GC subtypes (clusters 1 and 2) to calculate the correlation between the classification and the clinical information and the results showed that there was a significant correlation between grade and subtype of GC, clarifying that the grade of GC had a greater impact on the subtype (Figure 6(a)). Moreover, there was a significant correlation between high-risk genes (GADD45B/SERPINE1) and the prognosis of patients with GC (Figure 6(b)). We analyzed the expression of GADD45B and SERPINE1 in GC samples of different races (Asian and White) using the TCGA_STAD dataset. Our results showed a significant difference in the expression of both GADD45B and SERPINE1 between Asian and White samples (*Supplementary 2*), with higher expression levels observed in White samples. These findings suggest the presence of racial heterogeneity in the expression of GADD45B and SERPINE1. Among them, GADD45B was poorly expressed in GC, while SERPINE1 was highly expressed in GC (Figure 6(c)). The STRING website analyzed the interaction of DEGs with the interaction networks of GADD45B and SERPINE1 genes obtained, respectively (Figures 6(d) and 6(e)).

**Figure 7:**
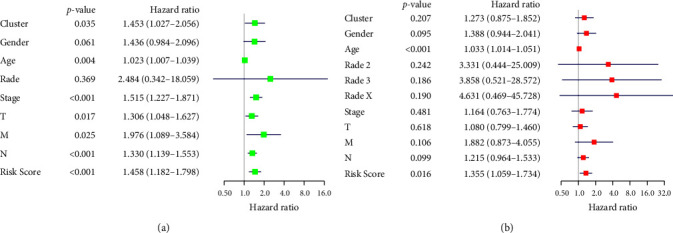
The reliability of the prognostic risk model based on GADD45B and SERPINE1 genes was evaluated using univariate and multivariate analyses. (a) Univariate independent prognostic analysis. The left side of the figure shows the name of the DEG, and the middle is the *p*-value. HR represents the risk ratio. The risk ratio is greater than 1, which means the gene is at high risk. The risk ratio is less than 1, which means low risk. The risk rate distribution of the genes is shown on the right side, where the left part indicates low risk and the right part indicates high risk. (b) Multivariate independent prognostic analysis. The left side of the figure represents the name of the DEG, where the middle part shows the *p*-value (the HR represents the risk rate. The risk rate greater than 1 indicates that the gene is at high risk; otherwise, it indicates low risk and the right side is the gene). The risk rate distribution of the gene is shown on the right side, where the left part indicates low-risk distribution and the right part indicates high-risk distribution.

According to Lasso regression analysis, the prognostic risk score of GC patients predicted by the prognostic risk model constructed based on GADD45B and SERPINE1 was calculated. Univariate analysis found that age, pathological stage, T stage, N stage, and risk score were significantly correlated with the prognosis of GC patients (Figure 7(a)). Furthermore, through multivariate analysis, age, and risk score were found to be independent risk factors for the prognosis of patients with GC (Figure 7(b)), which indicated that the prognostic risk model based on GADD45B and SERPINE1 was reliable.

In conclusion, GADD45B and SERPINE1 might significantly influence the occurrence and development of GC, and they might be important molecular markers for predicting the prognosis of GC patients and the prognostic risk model based on GADD45B and SERPINE1 could predict the prognosis of GC patients more accurately.

### 3.7. GADD45B Overexpression or SERPINE1 Silencing Inhibits the Biological Characteristics of GC Cells

**Figure 8:**
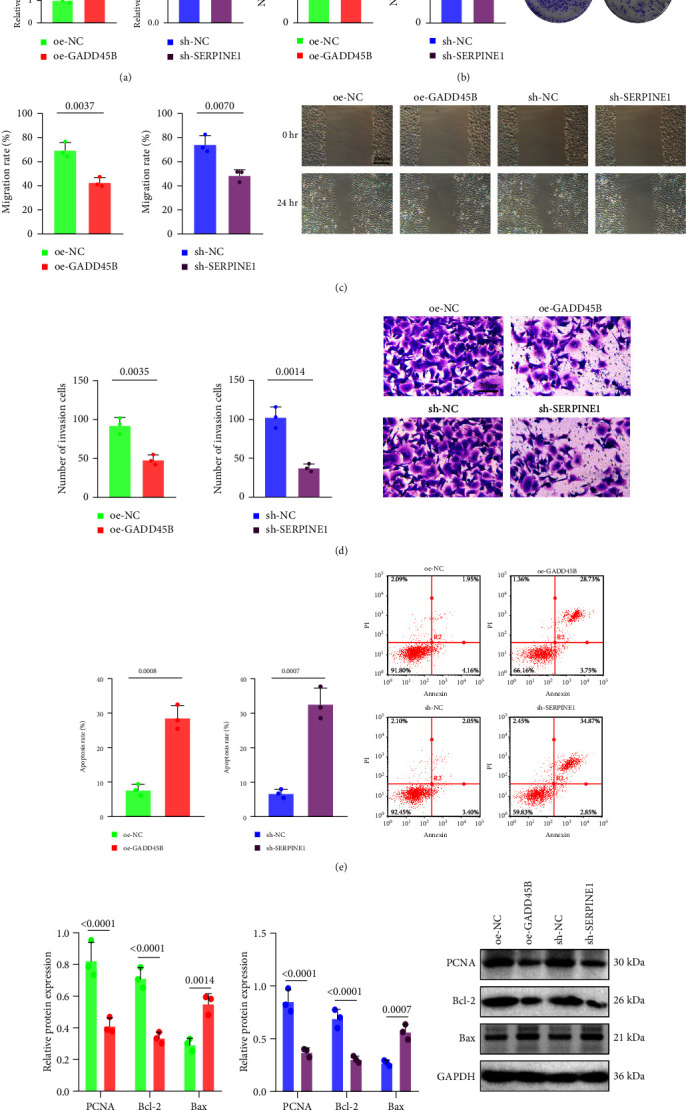
GADD45B overexpression or SERPINE1 silencing inhibits the biological characteristics of GC cells. (a) RT-qPCR was used to detect the expression of GADD45B or SERPINE1 in AGS cells. (b) Colony formation assay was used to detect the number of AGS cell clones after GADD45B overexpression or SERPINE1 silencing. (c) The migration rate of AGS cells in response to GADD45B overexpression or SERPINE1 silencing detected by scratch test (scale bar: 100 *μ*m). (d) The number of invading AGS cells in response to GADD45B overexpression or SERPINE1 silencing detected by transwell assay (scale bar: 50 *μ*m). (e) The apoptotic rate of AGS cells in response to GADD45B overexpression or SERPINE1 silencing detected by flow cytometry. (f) The protein expression of PCNA, Bcl-2, and Bax in AGS cells in response to GADD45B overexpression or SERPINE1 silencing detected by western blot assay.  ^*∗*^*p* < 0.05 vs. oe-NC or sh-NC group. The measurement data were expressed as mean ± standard deviation. An unpaired *t*-test was used for comparison between the two groups. The cell experiment was repeated three times.

Based on the above bioinformatics analysis, it was found that GADD45B and SERPINE1 might have a significant impact on the occurrence and development of GC; GADD45B was poorly expressed, while SERPINE1 was highly expressed in GC. In order to further understand the effects of GADD45B and SERPINE1 on the biological characteristics of GC cells, we overexpressed GADD45B or silenced SERPINE1 in AGS cells. RT-qPCR results showed that the expression of GADD45B in AGS cells was significantly increased after treatment with or-GADD45 B; the expression of SERPINE1 in AGS cells was notably decreased after treatment with sh-SERPINE1 (Figure 8(a)).

Next, colony formation assay, scratch test, Transwell assay, and flow cytometry showed that after overexpression of GADD45B or silencing of SERPINE1, the numbers of AGS cell clones and invading cells as well as migration rate were decreased, but the apoptotic rate increased significantly (Figure 8(b)–8(e)).

Western blot assay demonstrated that overexpression of GADD45B contributed to marked declines in the protein expression of proliferation-related factor proliferating cell nuclear antigen and apoptosis-related factor Bcl-2, accompanied by notably increased protein expression of Bax. Silencing of SERPINE1 could lead to the same effects (Figure 8(f)).

These results suggested that overexpression of GADD45B or silencing SERPINE1 could suppress the biological characteristics of GC cells.

## 4. Discussion

GC is a famous malignant disease with high morbidity and mortality and poor prognosis, although great achievement has been made [16]. Owing to the high incidence and mortality of GC, it is urgent to understand its underlying molecular mechanism to discover new biomarkers [17]. DEGs between GC and adjacent tissues are identified from TCGA and GTEx databases [18]. This study aimed to explore and verify the possible key factors in the p53 signaling pathway that participated in the initiation and progression of GC using GTEx and TCGA databases. Therefore, this study revealed that p53 signaling pathway-related factors GADD45B and SERPINE1 might be key genes that participate in the development of GC.

At the beginning of this study, 4,944 significant DEGs were obtained, in which 2,465 genes were highly expressed, and 2,479 were poorly expressed in GC. Accumulating evidence shows that GTEx and TCGA are widely used to predict cancer-related genes; for example, by taking advantage of the TCGA database, 585 lncRNAs, and 927 protein-coding genes associated with the overall survival rate of GC are determined [19]. In the present study, after GSEA analysis, we found that the DEGs were mainly involved in the p53 signaling pathway, of which 27 DEGs were involved in the occurrence and development of GC. Moreover, through PCA, the 27 candidate genes in the p53 pathway could classify GC patients into two subtypes. p53, a widely recognized tumor suppressor gene has been reported to regulate human cancers by synthesizing cytochrome c oxidase 2, cytochrome c oxidase complex, and TP53-induced glycolysis and apoptosis regulators [20]. Importantly, mutations in p53 can be seen in most human cancers and can enhance the ability of tumor invasion [21, 22]. Moreover, CREPT knockdown inhibits GC growth by regulating cycle arrest, migration, and apoptosis through the ROS-regulated p53 pathway [23]. Activation of the p53/caspase-3 signaling pathway by acting enhanced the inhibitory effect of TRAIL on GC progression [24]. Hence, studying the factors in the p53 signaling pathway is necessary.

Subsequently, our study found that GADD45B and SERPINE1 genes were prognostic high-risk genes, and the regression coefficients of GADD45B and SERPINE1 genes were positive. GADD45B expression was found to be lowly expressed in GC tissues, while SERPINE1 was highly expressed in GC tissues. Therefore, these two genes might become important molecular markers for predicting the prognosis of GC patients. In GC cells with p53 gene mutations, the p53 signaling pathway may be disrupted, resulting in abnormal vital activities such as cell proliferation and apoptosis, ultimately leading to differences in GADD45B and SERPINE1 expression levels between GC and normal gastric tissue samples [25]. In addition, previous studies have suggested that ALK5 mediates GADD45B protein levels by regulating Smad2/3 phosphorylation [26, 27]. The incidence rate of p53 gene mutations in GC tissue has been reported to be as high as 40.9%, and studies have found that GADD45B expression is inhibited in GC with p53 mutations, which may contribute to the development and progression of GC [28]. Therefore, p53 gene mutations are one of the main reasons for the differences in GADD45B and SERPINE1 expression levels observed between GC and normal gastric tissue samples.

Furthermore, we demonstrated through *in vitro* cell experiments that overexpressed GADD45B or silenced SERPINE1 repressed the biological characteristics of GC cells. Notably, GADD45B expression has been documented to be correlated with GC prognosis [29]. The expression of GADD45G was negatively correlated with methylation level, and the methylation frequency of GADD45G in GC tissues was significantly higher than that in normal tissues [14]. Besides, SERPINE1 was identified as a potential prognostic biomarker associated with poor prognosis in epithelial–mesenchymal transition in GC [30]. Elevated expression of SERPINE1 was found in GC tissues, and its high expression was correlated with poor outcomes, highlighting it as a diagnostic and prognostic biomarker for GC [31].

Additionally, SERPINE1 level is overexpressed and significantly associated with poor prognosis of gastric adenocarcinoma revealed by microarray and bioinformatics [32]. Interestingly, SERPINE1 was unveiled to promote the proliferation, migration, and invasion of gastric adenocarcinoma by regulating epithelial–mesenchymal transition [33]. The literature mentioned above further confirmed that GADD45B and SERPINE1 may have therapeutic potential and be used as prognostic markers for GC.

## 5. Conclusion

**Figure 9:**
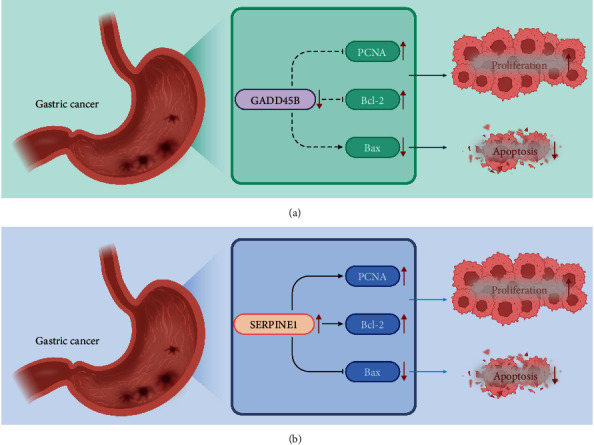
Molecular mechanism plot of GADD45B (a) and SERPINE1 (b) affecting biological characteristics of GC cells. GADD45B is downregulated, while SERPINE1 is upregulated in GC. Overexpression of GADD45B can inhibit the proliferation, migration, and invasion of GC cells and promote their apoptosis, while overexpression of SERPINE1 can promote the proliferation, migration, and invasion of GC cells and inhibit their apoptosis. GADD45B and SERPINE1 may be key genes participating in GC development.

Collectively, our data suggest that GADD45B and SERPINE1, involved in the p53 signaling pathway, may be key genes participating in GC development (Figure 9). Furthermore, the GADD45B- and SERPINE1-based risk models have good accuracy in predicting the prognosis of GC patients, which might aid in treatment decision-making in the clinic.

## Data Availability

All data generated or analyzed during this study are included in this published article.

## Supplementary Materials

Supplementary 1 RT-qPCR primer sequences. 206 adjacent normal tissue samples and 375 GC tissue samples. Information of 4,944 differential genes.

Supplementary 2 The boxplot of GADD45B and SERPINE1 expression in gastric cancer samples of different races (Asian and White) in the TCGA_STAD dataset.
