# Supplementary material for: Key Roles of p53 Signaling Pathway-Related Factors GADD45B and SERPINE1 in the Occurrence and Development of Gastric Cancer
Source: Mediators Inflamm. 2023 Aug 24;2023:6368893. doi: 10.1155/2023/6368893 (PMC10471451; doi:10.1155/2023/6368893)
Supplement: Supplementary 1 — RT-qPCR primer sequences. 206 adjacent normal tissue samples and 375 GC tissue samples. Information of 4,944 differential genes. [file 6368893.f1.docx]

**Supplementary Table 1** RT-qPCR primer sequences

| Gene | Forward primer | Reverse primer |
| --- | --- | --- |
| GADD45B | 5'-TGAATGTGGACCCAGACAGC-3' | 5'-TCGTGACCAGGAGACAATGC-3' |
| SERPINE1 | 5'-GCAAGGCACCTCTGAGAACT-3' | 5'-GGGTGAGAAAACCACGTTGC-3' |
| GAPDH | 5'-AATGGGCAGCCGTTAGGAAA-3' | 5'-GCGCCCAATACGACCAAATC-3' |

Note: RT-qPCR, reverse transcription quantitative polymerase chain reaction.

**Supplementary Table 2** 206 adjacent normal tissue samples and 375 GC tissue samples

| Normal | Tumor |
| --- | --- |
| GTEX-QLQ7-0826-SM-447B3 | TCGA-D7-5577-01A |
| GTEX-132AR-2426-SM-5IFFD | TCGA-D7-6818-01A |
| GTEX-11EQ9-1226-SM-5987E | TCGA-BR-4280-01A |
| GTEX-RWS6-0926-SM-47JXE | TCGA-D7-8572-01A |
| GTEX-13X6H-1626-SM-5Q5CT | TCGA-VQ-A91Z-01A |
| GTEX-ZTX8-0426-SM-59HLG | TCGA-HU-A4HD-01A |
| GTEX-11EM3-1026-SM-5A5KL | TCGA-D7-8573-01A |
| GTEX-S3XE-1026-SM-4AD4O | TCGA-BR-7959-01A |
| GTEX-ZYY3-1726-SM-5EGH3 | TCGA-BR-8679-01A |
| GTEX-111YS-1126-SM-5GZYQ | TCGA-CG-5726-01A |
| GTEX-139YR-1926-SM-5LZXM | TCGA-BR-6453-01A |
| GTEX-OHPK-1526-SM-3MJGM | TCGA-BR-A4QL-01A |
| GTEX-XXEK-0826-SM-4BRWG | TCGA-VQ-AA6A-01A |
| GTEX-145MO-2226-SM-5Q5BN | TCGA-HF-7132-01A |
| GTEX-Y8E4-1526-SM-4WWDI | TCGA-BR-A4IV-01A |
| GTEX-ZYVF-2726-SM-5GID4 | TCGA-CG-5718-01A |
| GTEX-WYJK-2526-SM-4ONDF | TCGA-BR-8296-01A |
| GTEX-11P82-0726-SM-5PNYL | TCGA-R5-A7ZR-01A |
| GTEX-12WS9-1426-SM-5FQT3 | TCGA-BR-8680-01A |
| GTEX-11EI6-2426-SM-5PNVS | TCGA-VQ-A8P2-01A |
| GTEX-ZLFU-1326-SM-4WWET | TCGA-CD-8533-01A |
| GTEX-13QBU-1126-SM-5LU44 | TCGA-RD-A8N2-01A |
| GTEX-ZV6S-0926-SM-57WGB | TCGA-VQ-A92D-01A |
| GTEX-WFON-1126-SM-4LVMA | TCGA-HU-A4GT-01A |
| GTEX-OOBJ-1526-SM-3NB1Q | TCGA-ZA-A8F6-01A |
| GTEX-12BJ1-1726-SM-5HL9B | TCGA-D7-A74A-01A |
| GTEX-XAJ8-0326-SM-47JYI | TCGA-IN-A7NR-01A |
| GTEX-XBED-1226-SM-4AT5V | TCGA-HU-A4H2-01A |
| GTEX-T6MO-0726-SM-4DM58 | TCGA-BR-6456-01A |
| GTEX-OIZH-1526-SM-3NB1J | TCGA-CG-4442-01A |
| GTEX-RM2N-0826-SM-48FD3 | TCGA-CD-8524-01A |
| GTEX-13RTK-0226-SM-5RQHR | TCGA-VQ-A8PO-01A |
| GTEX-S4P3-0726-SM-4AD57 | TCGA-BR-7717-01A |
| GTEX-13CF3-1926-SM-5K7WF | TCGA-CG-4475-01A |
| GTEX-P78B-1826-SM-3P5YX | TCGA-BR-4369-01A |
| GTEX-V955-1326-SM-4JBHR | TCGA-HU-A4GP-01A |
| GTEX-11GSP-2126-SM-5HL5E | TCGA-VQ-A94R-01A |
| GTEX-ZZ64-0426-SM-5E43F | TCGA-VQ-A91E-01A |
| GTEX-13D11-2126-SM-5IFH2 | TCGA-HU-A4GD-01A |
| GTEX-ZVT3-2126-SM-59HL2 | TCGA-FP-8211-01A |
| GTEX-SIU7-1426-SM-4BRWT | TCGA-D7-A747-01A |
| GTEX-P4PP-1526-SM-3P61M | TCGA-KB-A93G-01A |
| GTEX-12696-1726-SM-5EQLH | TCGA-RD-A8MV-01A |
| GTEX-ZEX8-1626-SM-4WKG7 | TCGA-BR-4256-01A |
| GTEX-12WSJ-0926-SM-5P9JD | TCGA-FP-7735-01A |
| GTEX-13111-1226-SM-5GCNC | TCGA-VQ-A8PK-01A |
| GTEX-QV31-0626-SM-447C5 | TCGA-BR-7707-01A |
| GTEX-SNMC-0626-SM-4DM6H | TCGA-IN-A6RS-01A |
| GTEX-14BMV-2226-SM-5RQHX | TCGA-D7-6519-01A |
| GTEX-XQ8I-2026-SM-4BOOL | TCGA-CG-4440-01A |
| GTEX-S4Z8-1226-SM-4AD6W | TCGA-BR-8487-01A |
| GTEX-PWN1-1526-SM-48TDA | TCGA-VQ-A94U-01A |
| GTEX-139D8-2326-SM-5IFGE | TCGA-CG-5717-01A |
| GTEX-QXCU-1926-SM-48FE4 | TCGA-BR-A4CR-01A |
| GTEX-WFJO-1126-SM-4LVLZ | TCGA-HU-8249-01A |
| GTEX-QCQG-0526-SM-48U2A | TCGA-BR-6454-01A |
| GTEX-YFCO-0826-SM-5LUAG | TCGA-BR-8295-01A |
| GTEX-WHSB-1226-SM-4M1XR | TCGA-BR-7722-01A |
| GTEX-NFK9-1526-SM-3LK7B | TCGA-HU-A4HB-01A |
| GTEX-132QS-1826-SM-5IFFN | TCGA-BR-7958-01A |
| GTEX-ZQUD-0826-SM-57WDQ | TCGA-BR-7197-01A |
| GTEX-XUZC-0726-SM-4BOPH | TCGA-VQ-A94P-01A |
| GTEX-13O21-0926-SM-5IFGT | TCGA-D7-8575-01A |
| GTEX-13113-0726-SM-5LZUF | TCGA-VQ-A8E0-01A |
| GTEX-QV44-1226-SM-4R1KE | TCGA-VQ-A8PP-01A |
| GTEX-S4Q7-0726-SM-4AD5F | TCGA-BR-8372-01A |
| GTEX-11NUK-2426-SM-5BC4U | TCGA-VQ-A91Y-01A |
| GTEX-YJ8A-1526-SM-5P9FT | TCGA-IN-A6RL-01A |
| GTEX-14BMU-1126-SM-5RQJ8 | TCGA-CG-5732-01A |
| GTEX-Q2AH-1126-SM-48TZM | TCGA-RD-A8MW-01A |
| GTEX-P4PQ-1526-SM-3NMCK | TCGA-KB-A6F7-01A |
| GTEX-Y3I4-1626-SM-4TT7W | TCGA-BR-8382-01A |
| GTEX-Q734-1026-SM-48U16 | TCGA-BR-8060-01A |
| GTEX-144GN-1226-SM-5O991 | TCGA-HU-A4GF-01A |
| GTEX-11VI4-0326-SM-5EQ6L | TCGA-R5-A7ZI-01A |
| GTEX-UJMC-1226-SM-4IHLI | TCGA-BR-8588-01A |
| GTEX-PLZ6-0826-SM-3P61K | TCGA-CD-5801-01A |
| GTEX-TKQ2-0926-SM-4DXU5 | TCGA-CD-5800-01A |
| GTEX-WYVS-1326-SM-4ONCQ | TCGA-CG-4462-01A |
| GTEX-RU1J-0526-SM-46MUT | TCGA-CD-8530-01A |
| GTEX-QDVN-1226-SM-48TZ5 | TCGA-VQ-A8PX-01A |
| GTEX-11NSD-1426-SM-5HL67 | TCGA-D7-8578-01A |
| GTEX-S341-0626-SM-4AD5T | TCGA-BR-6458-01A |
| GTEX-YEC3-1426-SM-5PNXW | TCGA-VQ-A8PB-01A |
| GTEX-ZDYS-1926-SM-5HL59 | TCGA-D7-6822-01A |
| GTEX-Y3IK-1426-SM-4YCES | TCGA-BR-8284-01A |
| GTEX-UPK5-2126-SM-4JBJK | TCGA-3M-AB47-01A |
| GTEX-R53T-1226-SM-48FCT | TCGA-BR-8381-01A |
| GTEX-ZF29-1526-SM-4WKF7 | TCGA-BR-4191-01A |
| GTEX-13OW6-2526-SM-5IJEC | TCGA-MX-A666-01A |
| GTEX-Y8LW-0826-SM-4WWDO | TCGA-CD-A486-01A |
| GTEX-1339X-1426-SM-5K7YO | TCGA-BR-8077-01A |
| GTEX-PW2O-1226-SM-48TCH | TCGA-MX-A663-01A |
| GTEX-146FH-2126-SM-5SI9U | TCGA-R5-A7O7-01A |
| GTEX-146FR-2026-SM-5NQAI | TCGA-VQ-A8DZ-01A |
| GTEX-T5JW-0926-SM-4DM5K | TCGA-BR-A44T-01A |
| GTEX-13FTW-1726-SM-5KM2B | TCGA-HU-A4H6-01A |
| GTEX-UJHI-1026-SM-4IHJP | TCGA-BR-A4CS-01A |
| GTEX-T5JC-1926-SM-4DM6Q | TCGA-CG-4438-01A |
| GTEX-ZXES-1426-SM-5NQ8S | TCGA-RD-A7BW-01A |
| GTEX-131XF-1226-SM-5HL8V | TCGA-D7-8570-01A |
| GTEX-ZE7O-3026-SM-51MS4 | TCGA-BR-4368-01A |
| GTEX-131XE-2226-SM-5PNYX | TCGA-CG-5720-01A |
| GTEX-R55G-1126-SM-48FDG | TCGA-D7-8579-01A |
| GTEX-13OVI-2426-SM-5KM4J | TCGA-IN-A6RO-01A |
| GTEX-11I78-1726-SM-5A5M3 | TCGA-VQ-AA69-01A |
| GTEX-U8XE-2126-SM-5CHRC | TCGA-IP-7968-01A |
| GTEX-S7SF-0626-SM-4AD4V | TCGA-BR-8371-01A |
| GTEX-WFG8-1326-SM-4LVN3 | TCGA-KB-A93J-01A |
| GTEX-12WSD-2326-SM-59HKQ | TCGA-D7-A4YU-01A |
| GTEX-ZAB4-1526-SM-5CVN7 | TCGA-BR-6705-01A |
| GTEX-111CU-0926-SM-5EGIK | TCGA-BR-6803-01A |
| GTEX-WY7C-1726-SM-4ONCC | TCGA-D7-A6EX-01A |
| GTEX-ZP4G-1226-SM-4WWCJ | TCGA-HU-A4G9-01A |
| GTEX-S4UY-1626-SM-4AD55 | TCGA-BR-6564-01A |
| GTEX-QDVJ-1426-SM-48U1Y | TCGA-D7-A4YX-01A |
| GTEX-WXYG-1626-SM-4ONCR | TCGA-CG-4441-01A |
| GTEX-QMRM-1126-SM-447BN | TCGA-VQ-A8DV-01A |
| GTEX-O5YW-1526-SM-3MJGL | TCGA-CG-5719-01A |
| GTEX-YEC4-1426-SM-5IFHS | TCGA-HU-A4GX-01A |
| GTEX-VJYA-1026-SM-4KL21 | TCGA-BR-8483-01A |
| GTEX-ZVZP-1726-SM-5GZWY | TCGA-VQ-A8PC-01A |
| GTEX-Y114-1226-SM-4TT88 | TCGA-CD-8526-01A |
| GTEX-SNOS-0826-SM-4DM5N | TCGA-BR-8380-01A |
| GTEX-TKQ1-0526-SM-4DXTG | TCGA-VQ-A8PQ-01A |
| GTEX-V1D1-1726-SM-4JBHB | TCGA-BR-8291-01A |
| GTEX-11WQK-2626-SM-5EQ4K | TCGA-HU-A4GQ-01A |
| GTEX-ZVP2-1626-SM-5GU5D | TCGA-BR-8081-01A |
| GTEX-XV7Q-1326-SM-4BRWM | TCGA-HU-A4H4-01A |
| GTEX-X15G-1126-SM-4PQZG | TCGA-VQ-A91K-01A |
| GTEX-1399R-1126-SM-5IFIO | TCGA-R5-A7ZE-01B |
| GTEX-OXRK-1626-SM-3NB17 | TCGA-BR-4367-01A |
| GTEX-Q2AI-0826-SM-48TZO | TCGA-BR-8297-01A |
| GTEX-Y5V6-1326-SM-4VDTF | TCGA-BR-8687-01A |
| GTEX-13PVQ-2126-SM-5L3FW | TCGA-BR-8683-01A |
| GTEX-SUCS-0926-SM-4DM4Z | TCGA-F1-A72C-01A |
| GTEX-WH7G-1526-SM-4LVMX | TCGA-VQ-A8PM-01A |
| GTEX-W5WG-1726-SM-4LMI5 | TCGA-VQ-A925-01A |
| GTEX-WHWD-1426-SM-4OORU | TCGA-IN-7808-01A |
| GTEX-ZZPU-1426-SM-5GZZ6 | TCGA-HU-A4G8-01A |
| GTEX-ZF2S-1426-SM-57WET | TCGA-CD-A48A-01A |
| GTEX-11DXX-1326-SM-5GIDZ | TCGA-B7-A5TJ-01A |
| GTEX-T8EM-1226-SM-4DM5J | TCGA-IN-8462-01A |
| GTEX-PWOO-1226-SM-48TCO | TCGA-BR-4294-01A |
| GTEX-13S7M-1826-SM-5RQK6 | TCGA-BR-A4J4-01A |
| GTEX-WZTO-2126-SM-4PQYW | TCGA-IN-7806-01A |
| GTEX-WRHK-0626-SM-4MVOE | TCGA-VQ-AA6F-01A |
| GTEX-POMQ-0826-SM-3P61H | TCGA-BR-A4J8-01A |
| GTEX-117YW-2226-SM-5N9DB | TCGA-BR-6707-01A |
| GTEX-ZYFG-1326-SM-5GICJ | TCGA-HU-A4GC-01A |
| GTEX-QLQW-0726-SM-447AA | TCGA-BR-8682-01A |
| GTEX-U8T8-1226-SM-4E3IH | TCGA-HU-A4H0-01A |
| GTEX-ZV7C-1626-SM-5NQ7E | TCGA-BR-8677-01A |
| GTEX-13FH7-1026-SM-5IJGF | TCGA-FP-7998-01A |
| GTEX-ZVT2-1626-SM-51MRC | TCGA-D7-8574-01A |
| GTEX-YEC3-1426-SM-4WWEM | TCGA-VQ-A8P5-01A |
| GTEX-11TT1-0726-SM-5GU5A | TCGA-CD-8532-01A |
| GTEX-131XG-1226-SM-5EGH9 | TCGA-VQ-A91A-01A |
| GTEX-R55D-1526-SM-48FEJ | TCGA-CG-4466-01A |
| GTEX-P4QT-1526-SM-3NMCT | TCGA-BR-8369-01A |
| GTEX-Y5LM-1326-SM-5RQIS | TCGA-D7-A4Z0-01A |
| GTEX-145ME-1026-SM-5O9B4 | TCGA-CG-4444-01A |
| GTEX-WFON-1126-SM-5CHTG | TCGA-BR-7715-01A |
| GTEX-1211K-1426-SM-5FQTF | TCGA-BR-8590-01A |
| GTEX-1122O-1926-SM-5EGIQ | TCGA-VQ-A927-01A |
| GTEX-13O61-1126-SM-5L3FI | TCGA-D7-A6EV-01A |
| GTEX-ZPIC-2026-SM-57WG3 | TCGA-D7-A748-01A |
| GTEX-Y9LG-1026-SM-5IFJN | TCGA-VQ-A91U-01A |
| GTEX-13O3Q-2126-SM-5KM4C | TCGA-IN-8663-01A |
| GTEX-ZLV1-0826-SM-4WWEQ | TCGA-CG-4477-01A |
| GTEX-PX3G-1526-SM-48U11 | TCGA-CG-4460-01A |
| GTEX-13FTX-0726-SM-5N9BI | TCGA-RD-A8N5-01A |
| GTEX-WFG7-1326-SM-4LMK1 | TCGA-BR-4363-01A |
| GTEX-12C56-0526-SM-5FQST | TCGA-D7-A6EZ-01A |
| TCGA-HU-A4GY-11A | TCGA-IN-A7NT-01A |
| TCGA-IP-7968-11A | TCGA-VQ-A8PD-01A |
| TCGA-CG-5721-11A | TCGA-VQ-A924-01A |
| TCGA-HU-A4GH-11A | TCGA-IN-A6RN-01A |
| TCGA-HU-A4GP-11A | TCGA-HU-A4GH-01A |
| TCGA-BR-6454-11A | TCGA-HU-A4H8-01A |
| TCGA-CG-5730-11A | TCGA-BR-4370-01A |
| TCGA-HU-A4GN-11A | TCGA-FP-A9TM-01A |
| TCGA-CG-5722-11A | TCGA-BR-6852-01A |
| TCGA-FP-7829-11A | TCGA-CG-4476-01A |
| TCGA-IN-AB1X-11A | TCGA-VQ-A8DU-01A |
| TCGA-BR-7704-11A | TCGA-CG-4443-01A |
| TCGA-BR-7703-11A | TCGA-BR-4257-01A |
| TCGA-BR-6453-11A | TCGA-D7-6815-01A |
| TCGA-BR-7717-11A | TCGA-BR-A4J9-01A |
| TCGA-BR-7716-11A | TCGA-BR-7723-01A |
| TCGA-IN-7806-11A | TCGA-RD-A8NB-01A |
| TCGA-HU-A4GC-11A | TCGA-D7-6520-01A |
| TCGA-CG-5734-11A | TCGA-BR-6452-01A |
| TCGA-HU-8238-11A | TCGA-IN-A6RR-01A |
| TCGA-CG-5728-11A | TCGA-VQ-A928-01A |
| TCGA-BR-6802-11A | TCGA-BR-A4J7-01A |
| TCGA-HU-A4HB-11A | TCGA-BR-8059-01A |
| TCGA-BR-6457-11A | TCGA-VQ-A8DT-01A |
| TCGA-BR-7851-11A | TCGA-VQ-A8P8-01A |
| TCGA-FP-7735-11A | TCGA-VQ-A91D-01A |
| TCGA-BR-8060-11A | TCGA-BR-7901-01A |
| TCGA-IN-AB1V-11A | TCGA-CG-4306-01A |
| TCGA-BR-7715-11A | TCGA-BR-6565-01A |
| TCGA-CG-5733-11A | TCGA-BR-7704-01A |
| TCGA-CG-5720-11A | TCGA-CG-4436-01A |
| TCGA-IN-8663-11A | TCGA-HU-A4G2-01A |
|  | TCGA-VQ-A94T-01A |
|  | TCGA-VQ-A8PU-01A |
|  | TCGA-VQ-A8PJ-01A |
|  | TCGA-IN-A6RI-01A |
|  | TCGA-IN-A7NU-01A |
|  | TCGA-CD-8534-01A |
|  | TCGA-VQ-AA68-01A |
|  | TCGA-FP-8631-01A |
|  | TCGA-D7-A6EY-01A |
|  | TCGA-D7-6526-01A |
|  | TCGA-BR-8364-01A |
|  | TCGA-BR-6563-01A |
|  | TCGA-BR-8676-01A |
|  | TCGA-CG-5721-01A |
|  | TCGA-HF-7133-01A |
|  | TCGA-CD-8528-01A |
|  | TCGA-HU-8244-01A |
|  | TCGA-CD-5813-01A |
|  | TCGA-BR-8484-01A |
|  | TCGA-BR-6457-01A |
|  | TCGA-FP-7916-01A |
|  | TCGA-BR-4187-01A |
|  | TCGA-KB-A93H-01A |
|  | TCGA-BR-8592-01A |
|  | TCGA-BR-4366-01A |
|  | TCGA-BR-7851-01A |
|  | TCGA-SW-A7EA-01A |
|  | TCGA-CD-5803-01A |
|  | TCGA-D7-6525-01A |
|  | TCGA-RD-A8N9-01A |
|  | TCGA-BR-4279-01A |
|  | TCGA-BR-8080-01A |
|  | TCGA-BR-A4PF-01A |
|  | TCGA-HU-A4G3-01A |
|  | TCGA-CD-8531-01A |
|  | TCGA-R5-A7ZF-01A |
|  | TCGA-CD-A487-01A |
|  | TCGA-BR-8384-01A |
|  | TCGA-VQ-A922-01A |
|  | TCGA-B7-A5TI-01A |
|  | TCGA-BR-8368-01A |
|  | TCGA-BR-4357-01A |
|  | TCGA-BR-4371-01A |
|  | TCGA-VQ-A91N-01A |
|  | TCGA-VQ-AA6J-01A |
|  | TCGA-CG-5724-01A |
|  | TCGA-VQ-A94O-01A |
|  | TCGA-BR-8591-01A |
|  | TCGA-VQ-AA64-01A |
|  | TCGA-FP-8099-01A |
|  | TCGA-F1-A448-01A |
|  | TCGA-VQ-A91V-01A |
|  | TCGA-RD-A8N0-01A |
|  | TCGA-CD-8525-01A |
|  | TCGA-FP-A4BF-01A |
|  | TCGA-FP-A8CX-01A |
|  | TCGA-RD-A7BS-01A |
|  | TCGA-BR-7716-01A |
|  | TCGA-BR-8366-01A |
|  | TCGA-VQ-AA6G-01A |
|  | TCGA-CG-4465-01A |
|  | TCGA-HU-A4H5-01A |
|  | TCGA-CG-4437-01A |
|  | TCGA-CD-5804-01A |
|  | TCGA-F1-6875-01A |
|  | TCGA-HF-A5NB-01A |
|  | TCGA-BR-8365-01A |
|  | TCGA-CD-8527-01A |
|  | TCGA-D7-A6F0-01A |
|  | TCGA-BR-7957-01A |
|  | TCGA-IN-AB1X-01A |
|  | TCGA-BR-8678-01A |
|  | TCGA-D7-6528-01A |
|  | TCGA-HU-8238-01A |
|  | TCGA-BR-8367-01A |
|  | TCGA-BR-7196-01A |
|  | TCGA-FP-7829-01A |
|  | TCGA-B7-A5TN-01A |
|  | TCGA-VQ-A8E3-01A |
|  | TCGA-CG-5725-01A |
|  | TCGA-CG-4305-01A |
|  | TCGA-BR-6455-01A |
|  | TCGA-BR-6802-01A |
|  | TCGA-BR-6709-01A |
|  | TCGA-B7-A5TK-01A |
|  | TCGA-CD-A4MH-01A |
|  | TCGA-BR-6710-01A |
|  | TCGA-CD-A4MG-01A |
|  | TCGA-D7-6522-01A |
|  | TCGA-F1-6177-01A |
|  | TCGA-BR-4201-01A |
|  | TCGA-BR-4361-01A |
|  | TCGA-FP-8209-01A |
|  | TCGA-FP-8210-01A |
|  | TCGA-HU-8604-01A |
|  | TCGA-CG-4301-01A |
|  | TCGA-RD-A8N1-01A |
|  | TCGA-BR-A44U-01A |
|  | TCGA-CG-4469-01A |
|  | TCGA-VQ-A8PH-01A |
|  | TCGA-D7-8576-01A |
|  | TCGA-VQ-AA6K-01A |
|  | TCGA-HU-8602-01A |
|  | TCGA-BR-8485-01A |
|  | TCGA-VQ-A8E7-01B |
|  | TCGA-HU-A4GJ-01A |
|  | TCGA-BR-8686-01A |
|  | TCGA-BR-8589-01A |
|  | TCGA-RD-A8N6-01A |
|  | TCGA-D7-6524-01A |
|  | TCGA-CG-5722-01A |
|  | TCGA-BR-8486-01A |
|  | TCGA-VQ-A8PE-01A |
|  | TCGA-CD-8535-01A |
|  | TCGA-VQ-A91X-01A |
|  | TCGA-BR-A4J5-01A |
|  | TCGA-CD-5798-01A |
|  | TCGA-ZQ-A9CR-01A |
|  | TCGA-D7-6527-01A |
|  | TCGA-BR-8289-01A |
|  | TCGA-BR-6801-01A |
|  | TCGA-CG-5734-01A |
|  | TCGA-R5-A805-01A |
|  | TCGA-3M-AB46-01A |
|  | TCGA-CG-5716-01A |
|  | TCGA-BR-4253-01A |
|  | TCGA-BR-8058-01A |
|  | TCGA-VQ-A8P3-01A |
|  | TCGA-HU-A4H3-01A |
|  | TCGA-BR-4267-01A |
|  | TCGA-VQ-A8E2-01A |
|  | TCGA-MX-A5UG-01A |
|  | TCGA-RD-A8N4-01A |
|  | TCGA-IN-A6RJ-01A |
|  | TCGA-CG-5723-01A |
|  | TCGA-VQ-AA6D-01A |
|  | TCGA-F1-6874-01A |
|  | TCGA-CG-4304-01A |
|  | TCGA-VQ-A923-01A |
|  | TCGA-VQ-A8PF-01A |
|  | TCGA-BR-8361-01A |
|  | TCGA-BR-8690-01A |
|  | TCGA-HJ-7597-01A |
|  | TCGA-RD-A7BT-01A |
|  | TCGA-MX-A5UJ-01A |
|  | TCGA-VQ-A91Q-01A |
|  | TCGA-D7-5578-01A |
|  | TCGA-SW-A7EB-01A |
|  | TCGA-HU-A4GU-01A |
|  | TCGA-D7-6521-01A |
|  | TCGA-IN-AB1V-01A |
|  | TCGA-HF-7134-01A |
|  | TCGA-CD-A48C-01A |
|  | TCGA-HU-8610-01A |
|  | TCGA-BR-8373-01A |
|  | TCGA-EQ-8122-01A |
|  | TCGA-B7-5818-01A |
|  | TCGA-D7-A6F2-01A |
|  | TCGA-RD-A7C1-01A |
|  | TCGA-HF-7131-01A |
|  | TCGA-CD-5799-01A |
|  | TCGA-VQ-A91S-01A |
|  | TCGA-HU-A4GY-01A |
|  | TCGA-HU-8608-01A |
|  | TCGA-CD-A489-01A |
|  | TCGA-BR-6566-01A |
|  | TCGA-BR-A4J6-01A |
|  | TCGA-BR-8286-01A |
|  | TCGA-CD-8529-01A |

**Supplementary Table 3** Information of 4944 differential genes

| Gene | ConMean | TreatMean | LogFC | pValue | Fdr |
| --- | --- | --- | --- | --- | --- |
| RAB4B | 3.70753 | 2.178763 | -1.52877 | 3.78E-63 | 2.66E-62 |
| TIGAR | 1.535 | 2.712976 | 1.177976 | 1.59E-71 | 3.06E-70 |
| AP000892.3 | 3.571116 | 1.171735 | -2.39938 | 1.38E-55 | 5.54E-55 |
| RMND5A | 2.517982 | 3.707196 | 1.189214 | 1.56E-66 | 1.56E-65 |
| AL671277.1 | 1.425774 | 3.23638 | 1.810606 | 4.11E-45 | 1.11E-44 |
| IL32 | 3.946564 | 5.473953 | 1.527389 | 1.23E-43 | 3.19E-43 |
| RPS4XP3 | 0.423577 | 1.672895 | 1.249317 | 9.55E-58 | 4.32E-57 |
| NUCB2 | 4.223822 | 2.9138 | -1.31002 | 8.52E-63 | 5.76E-62 |
| SPATA2 | 1.991567 | 3.396763 | 1.405196 | 7.01E-73 | 1.70E-71 |
| COLCA1 | 2.696475 | 1.262195 | -1.43428 | 3.72E-33 | 7.55E-33 |
| ST3GAL6 | 1.969774 | 0.757235 | -1.21254 | 5.06E-63 | 3.50E-62 |
| PKP2 | 2.636722 | 3.807718 | 1.170996 | 4.35E-44 | 1.14E-43 |
| IGHV3-43 | 1.618941 | 2.650346 | 1.031406 | 1.60E-12 | 2.18E-12 |
| ESM1 | 0.286922 | 1.764347 | 1.477425 | 1.51E-72 | 3.47E-71 |
| AC011481.1 | 3.651461 | 4.667951 | 1.01649 | 2.41E-54 | 9.06E-54 |
| MT1L | 3.347158 | 1.988864 | -1.35829 | 4.55E-45 | 1.22E-44 |
| MYOM2 | 1.427297 | 0.254029 | -1.17327 | 5.96E-75 | 2.08E-73 |
| APIP | 3.340058 | 2.184356 | -1.1557 | 1.14E-55 | 4.59E-55 |
| NPHP3 | 2.610982 | 0.7375 | -1.87348 | 5.44E-62 | 3.42E-61 |
| MITD1 | 3.275781 | 2.235902 | -1.03988 | 7.57E-49 | 2.28E-48 |
| ZMAT3 | 1.484943 | 2.573977 | 1.089034 | 3.94E-60 | 2.13E-59 |
| MT1A | 3.554668 | 1.577669 | -1.977 | 1.09E-45 | 2.99E-45 |
| PKMYT1 | 1.262458 | 2.520148 | 1.25769 | 9.69E-45 | 2.58E-44 |
| PHF1 | 5.09805 | 3.488167 | -1.60988 | 1.32E-72 | 3.08E-71 |
| IGHV1-24 | 2.156169 | 4.16585 | 2.009681 | 9.43E-24 | 1.58E-23 |
| HLA-DRB1 | 5.053642 | 7.838745 | 2.785104 | 1.98E-59 | 1.02E-58 |
| NCAPG | 0.727958 | 2.461123 | 1.733165 | 4.21E-70 | 6.65E-69 |
| MELTF | 1.082161 | 2.21723 | 1.135068 | 2.21E-33 | 4.50E-33 |
| KLK10 | 1.039734 | 2.755535 | 1.7158 | 4.77E-16 | 6.91E-16 |
| MIR4292 | 0.208703 | 2.302028 | 2.093325 | 1.13E-74 | 3.74E-73 |
| FRMD8 | 2.823689 | 3.936074 | 1.112385 | 1.20E-69 | 1.77E-68 |
| HELZ2 | 2.566666 | 3.794466 | 1.2278 | 2.03E-53 | 7.32E-53 |
| ZNF767P | 2.675215 | 1.556302 | -1.11891 | 4.30E-58 | 1.99E-57 |
| SNORD99 | 0.124391 | 1.865162 | 1.740771 | 1.19E-75 | 4.65E-74 |
| SLC2A1 | 3.241525 | 4.690453 | 1.448928 | 3.63E-42 | 9.02E-42 |
| FOXD2-AS1 | 0.483159 | 1.83218 | 1.349021 | 6.95E-78 | 4.55E-76 |
| RPRM | 2.094197 | 0.373565 | -1.72063 | 1.62E-72 | 3.70E-71 |
| AC009220.1 | 0.266305 | 1.362569 | 1.096263 | 9.91E-59 | 4.81E-58 |
| CAMK1 | 3.076687 | 1.439011 | -1.63768 | 2.12E-68 | 2.66E-67 |
| WFDC1 | 2.771211 | 1.199669 | -1.57154 | 1.00E-59 | 5.23E-59 |
| ADIRF-AS1 | 2.194043 | 0.545565 | -1.64848 | 1.45E-75 | 5.56E-74 |
| PHYKPL | 4.290802 | 2.387079 | -1.90372 | 5.98E-68 | 7.06E-67 |
| NME1-NME2 | 2.017404 | 0.544786 | -1.47262 | 7.41E-55 | 2.86E-54 |
| PHLDA2 | 2.591911 | 6.016159 | 3.424248 | 1.55E-68 | 1.98E-67 |
| EVPL | 1.715413 | 3.47092 | 1.755507 | 2.30E-59 | 1.18E-58 |
| RDH12 | 1.798974 | 0.522192 | -1.27678 | 7.69E-38 | 1.72E-37 |
| IL27RA | 1.375567 | 2.796954 | 1.421387 | 1.68E-65 | 1.51E-64 |
| CENPH | 1.205664 | 2.23761 | 1.031946 | 4.67E-61 | 2.71E-60 |
| AC005165.1 | 1.402532 | 0.277837 | -1.1247 | 2.46E-58 | 1.16E-57 |
| ATP6AP2 | 4.366235 | 5.746859 | 1.380623 | 2.38E-73 | 6.24E-72 |
| MTND4P24 | 0.523269 | 3.501972 | 2.978703 | 2.46E-67 | 2.68E-66 |
| ACOX2 | 2.404366 | 1.231786 | -1.17258 | 1.43E-37 | 3.19E-37 |
| MIR100HG | 2.430893 | 1.316178 | -1.11472 | 1.24E-18 | 1.90E-18 |
| CYP2E1 | 1.911231 | 0.411023 | -1.50021 | 3.89E-60 | 2.11E-59 |
| RPS13P2 | 1.214038 | 3.231139 | 2.017101 | 8.38E-60 | 4.41E-59 |
| RPL41 | 9.881611 | 8.28819 | -1.59342 | 2.33E-56 | 9.76E-56 |
| TP73-AS1 | 2.817395 | 1.119363 | -1.69803 | 9.76E-72 | 1.95E-70 |
| ASB2 | 2.571482 | 1.404127 | -1.16736 | 1.15E-22 | 1.89E-22 |
| MZT2A | 4.681774 | 3.313291 | -1.36848 | 1.27E-58 | 6.11E-58 |
| EXOSC7 | 3.559128 | 2.371274 | -1.18785 | 2.61E-59 | 1.33E-58 |
| IGHGP | 0.734332 | 2.552083 | 1.817751 | 5.11E-47 | 1.46E-46 |
| RECQL5 | 3.782711 | 1.960503 | -1.82221 | 1.19E-60 | 6.69E-60 |
| NOX1 | 0.140612 | 1.350392 | 1.20978 | 1.62E-61 | 9.78E-61 |
| AC136632.1 | 1.943555 | 3.946517 | 2.002962 | 7.65E-62 | 4.76E-61 |
| RCN3 | 3.173896 | 4.201269 | 1.027373 | 4.67E-30 | 8.87E-30 |
| NCEH1 | 1.788014 | 3.864322 | 2.076308 | 1.15E-75 | 4.51E-74 |
| ZFYVE28 | 2.422017 | 1.241109 | -1.18091 | 7.26E-52 | 2.46E-51 |
| RNF24 | 1.799235 | 2.93468 | 1.135445 | 2.99E-55 | 1.18E-54 |
| UQCRB | 6.596336 | 4.097512 | -2.49882 | 2.08E-67 | 2.29E-66 |
| PRRT1 | 2.427847 | 0.9617 | -1.46615 | 1.86E-67 | 2.06E-66 |
| RNF5 | 4.734719 | 5.965598 | 1.23088 | 2.52E-57 | 1.12E-56 |
| NPIPB14P | 2.802139 | 0.283074 | -2.51907 | 1.37E-53 | 4.99E-53 |
| SNORA11F | 0.380186 | 1.392098 | 1.011912 | 2.18E-49 | 6.69E-49 |
| MRPL22 | 3.734815 | 2.556295 | -1.17852 | 4.14E-61 | 2.42E-60 |
| EEF1A1P11 | 0.864622 | 2.325211 | 1.460589 | 1.83E-54 | 6.93E-54 |
| RMI2 | 1.192654 | 2.793367 | 1.600713 | 2.81E-66 | 2.72E-65 |
| UROD | 4.508987 | 3.46837 | -1.04062 | 5.05E-73 | 1.25E-71 |
| SOSTDC1 | 2.45896 | 1.08568 | -1.37328 | 2.04E-32 | 4.08E-32 |
| CAPN3 | 2.201428 | 0.178491 | -2.02294 | 6.76E-64 | 5.15E-63 |
| MEP1A | 0.459547 | 1.728873 | 1.269326 | 1.56E-28 | 2.87E-28 |
| POGK | 2.500602 | 3.540347 | 1.039745 | 3.96E-78 | 2.72E-76 |
| AC091390.3 | 0.183815 | 1.426245 | 1.24243 | 4.57E-75 | 1.63E-73 |
| RAD54L | 0.764326 | 1.900219 | 1.135893 | 3.92E-57 | 1.72E-56 |
| AC245033.3 | 3.277856 | 0.436145 | -2.84171 | 4.55E-57 | 1.99E-56 |
| RPL3P4 | 4.578375 | 6.077972 | 1.499596 | 2.93E-47 | 8.39E-47 |
| SNORD63 | 0.140896 | 1.346971 | 1.206075 | 4.39E-59 | 2.19E-58 |
| AKR1C2 | 4.496428 | 1.163231 | -3.3332 | 1.68E-68 | 2.14E-67 |
| ADCY2 | 1.294464 | 0.24443 | -1.05003 | 1.35E-68 | 1.75E-67 |
| TMEM86B | 2.496534 | 0.912995 | -1.58354 | 3.59E-54 | 1.34E-53 |
| PDE1C | 1.556793 | 0.369707 | -1.18709 | 7.23E-74 | 2.08E-72 |
| MRPS25 | 4.059304 | 2.991136 | -1.06817 | 2.58E-58 | 1.21E-57 |
| MZT1 | 1.853533 | 3.779968 | 1.926436 | 3.91E-82 | 1.43E-79 |
| CREM | 3.18625 | 2.080988 | -1.10526 | 1.09E-55 | 4.40E-55 |
| AC139768.1 | 2.441645 | 1.048434 | -1.39321 | 4.61E-67 | 4.87E-66 |
| BIN3 | 3.140148 | 1.984896 | -1.15525 | 3.45E-63 | 2.44E-62 |
| PLP1 | 2.168601 | 0.330852 | -1.83775 | 1.64E-70 | 2.75E-69 |
| LYZ | 6.338828 | 9.01922 | 2.680392 | 6.25E-33 | 1.26E-32 |
| XYLT2 | 4.767583 | 3.052725 | -1.71486 | 6.30E-28 | 1.15E-27 |
| DDX21 | 3.335467 | 5.154187 | 1.818719 | 7.42E-79 | 8.57E-77 |
| SH3BGR | 2.530977 | 1.262109 | -1.26887 | 8.10E-48 | 2.36E-47 |
| LINC01145 | 2.125116 | 0.664074 | -1.46104 | 7.88E-63 | 5.36E-62 |
| CCDC167 | 3.000546 | 4.470242 | 1.469696 | 3.71E-59 | 1.86E-58 |
| FGFR4 | 2.350838 | 3.893125 | 1.542287 | 1.27E-44 | 3.36E-44 |
| WDR36 | 1.858822 | 2.877817 | 1.018996 | 1.63E-75 | 6.17E-74 |
| XPO4 | 1.552903 | 2.586757 | 1.033854 | 6.27E-73 | 1.54E-71 |
| AL138721.1 | 0.389137 | 1.789441 | 1.400304 | 5.87E-58 | 2.70E-57 |
| ECHDC3 | 3.129172 | 1.756325 | -1.37285 | 5.16E-30 | 9.80E-30 |
| SPI1 | 2.528491 | 3.921278 | 1.392787 | 3.20E-44 | 8.40E-44 |
| NBPF9 | 2.248905 | 1.002894 | -1.24601 | 4.37E-52 | 1.49E-51 |
| SCPEP1 | 4.888304 | 3.66311 | -1.22519 | 5.59E-53 | 1.97E-52 |
| GC | 2.096284 | 1.022606 | -1.07368 | 3.38E-21 | 5.40E-21 |
| FBXO28 | 2.18825 | 3.370726 | 1.182476 | 2.59E-71 | 4.86E-70 |
| PDIA3 | 6.221265 | 7.447073 | 1.225809 | 1.10E-65 | 1.01E-64 |
| UPF2 | 2.511536 | 3.667578 | 1.156042 | 2.51E-69 | 3.56E-68 |
| SLPI | 5.281633 | 7.008647 | 1.727014 | 5.62E-27 | 1.00E-26 |
| TOR4A | 2.609458 | 4.404947 | 1.795489 | 2.26E-48 | 6.73E-48 |
| HAUS7 | 2.715739 | 0.600767 | -2.11497 | 4.05E-49 | 1.23E-48 |
| MED1 | 2.311563 | 3.338636 | 1.027073 | 5.88E-71 | 1.04E-69 |
| PDCD4 | 5.779062 | 3.955032 | -1.82403 | 1.45E-52 | 5.02E-52 |
| IGKJ5 | 0.564396 | 2.601234 | 2.036839 | 2.40E-36 | 5.21E-36 |
| FP236383.3 | 6.952022 | 1.638306 | -5.31372 | 1.55E-70 | 2.60E-69 |
| WDR90 | 3.409214 | 2.221079 | -1.18813 | 2.27E-43 | 5.81E-43 |
| CASC19 | 0.249925 | 1.564878 | 1.314953 | 2.40E-55 | 9.51E-55 |
| NPIPB3 | 4.20516 | 0.736558 | -3.4686 | 1.80E-57 | 8.03E-57 |
| F2RL2 | 0.812237 | 2.680753 | 1.868516 | 7.39E-65 | 6.23E-64 |
| RPL38 | 8.102475 | 7.010544 | -1.09193 | 1.35E-51 | 4.51E-51 |
| AC011933.1 | 0.225078 | 1.722894 | 1.497816 | 3.30E-73 | 8.46E-72 |
| PRRT2 | 2.089729 | 0.682932 | -1.4068 | 9.56E-62 | 5.90E-61 |
| TONSL | 1.37101 | 2.846111 | 1.4751 | 3.57E-71 | 6.56E-70 |
| TYK2 | 5.00165 | 3.710678 | -1.29097 | 3.58E-50 | 1.13E-49 |
| RNU4-80P | 0.130969 | 1.449133 | 1.318164 | 5.53E-59 | 2.74E-58 |
| FAHD2B | 3.463992 | 1.362672 | -2.10132 | 7.69E-76 | 3.13E-74 |
| IGHV3-21 | 3.330451 | 5.539645 | 2.209194 | 1.20E-22 | 1.98E-22 |
| FXYD6 | 4.11246 | 1.806823 | -2.30564 | 4.35E-53 | 1.54E-52 |
| RPS26P31 | 0.313525 | 1.428725 | 1.1152 | 8.60E-61 | 4.89E-60 |
| CXCL8 | 1.422742 | 4.447532 | 3.02479 | 4.94E-57 | 2.15E-56 |
| RPS2P5 | 6.2787 | 7.386618 | 1.107917 | 2.84E-28 | 5.20E-28 |
| MICOS10 | 5.480426 | 3.013481 | -2.46694 | 9.97E-65 | 8.32E-64 |
| UBE2J1 | 3.416837 | 4.867011 | 1.450174 | 1.06E-56 | 4.52E-56 |
| RPL23AP82 | 2.502254 | 1.164395 | -1.33786 | 4.83E-68 | 5.78E-67 |
| MZT2B | 6.086785 | 4.596082 | -1.4907 | 6.42E-66 | 5.99E-65 |
| RNY4P34 | 0.272134 | 1.551773 | 1.279639 | 3.99E-51 | 1.31E-50 |
| VCAM1 | 1.389436 | 2.458483 | 1.069047 | 4.30E-36 | 9.29E-36 |
| POPDC3 | 1.656732 | 0.414164 | -1.24257 | 1.09E-54 | 4.19E-54 |
| PBX1 | 3.145074 | 1.933233 | -1.21184 | 6.12E-45 | 1.64E-44 |
| LAMTOR4 | 5.819603 | 4.710475 | -1.10913 | 2.00E-56 | 8.44E-56 |
| ADAMTSL4 | 3.651316 | 1.9526 | -1.69872 | 7.64E-53 | 2.68E-52 |
| SORBS2 | 4.326776 | 1.510003 | -2.81677 | 4.65E-73 | 1.15E-71 |
| RARRES1 | 2.361434 | 3.542114 | 1.18068 | 3.24E-18 | 4.89E-18 |
| LINC00982 | 4.707614 | 0.745704 | -3.96191 | 4.80E-62 | 3.03E-61 |
| CFB | 4.148016 | 2.890061 | -1.25795 | 9.20E-26 | 1.61E-25 |
| NDUFA3 | 6.013521 | 4.658012 | -1.35551 | 7.26E-62 | 4.53E-61 |
| AC004057.1 | 6.685839 | 1.849351 | -4.83649 | 2.79E-60 | 1.53E-59 |
| RNASET2 | 4.593323 | 3.309852 | -1.28347 | 6.32E-34 | 1.30E-33 |
| RPS24 | 9.045493 | 7.342875 | -1.70262 | 7.21E-54 | 2.66E-53 |
| RPL9 | 8.219271 | 7.189306 | -1.02997 | 5.44E-58 | 2.50E-57 |
| AC011558.1 | 3.199869 | 0.002008 | -3.19786 | 1.78E-78 | 1.35E-76 |
| GCNT2 | 2.373092 | 0.99412 | -1.37897 | 3.48E-59 | 1.76E-58 |
| SNRNP35 | 3.316816 | 2.087105 | -1.22971 | 8.37E-70 | 1.27E-68 |
| DCAF7 | 3.212084 | 4.68359 | 1.471506 | 4.79E-82 | 1.68E-79 |
| CENPE | 0.495945 | 1.993155 | 1.49721 | 3.44E-77 | 1.87E-75 |
| EIF2AK2 | 2.053927 | 3.786571 | 1.732643 | 1.14E-74 | 3.77E-73 |
| ENDOV | 2.633961 | 1.131416 | -1.50254 | 1.29E-70 | 2.18E-69 |
| TXNP4 | 0.205823 | 1.25479 | 1.048967 | 2.06E-65 | 1.84E-64 |
| XDH | 0.586448 | 1.671253 | 1.084805 | 2.38E-39 | 5.52E-39 |
| ZDHHC20 | 3.29155 | 4.835734 | 1.544184 | 2.71E-68 | 3.35E-67 |
| CAPN2 | 6.030023 | 5.015542 | -1.01448 | 8.15E-47 | 2.31E-46 |
| BMP8A | 0.436903 | 1.644804 | 1.207901 | 1.44E-71 | 2.80E-70 |
| STAT1 | 3.625739 | 5.877445 | 2.251706 | 3.92E-79 | 5.16E-77 |
| AC015813.2 | 1.523206 | 0.074511 | -1.44869 | 8.93E-62 | 5.52E-61 |
| TSC2 | 4.691975 | 3.343585 | -1.34839 | 4.64E-69 | 6.30E-68 |
| METTL22 | 2.933102 | 1.281742 | -1.65136 | 1.04E-63 | 7.74E-63 |
| RN7SL752P | 0.232964 | 1.685566 | 1.452601 | 1.01E-64 | 8.42E-64 |
| ACAD8 | 3.356535 | 1.749745 | -1.60679 | 7.06E-68 | 8.25E-67 |
| ZNF528 | 2.188896 | 0.780532 | -1.40836 | 1.49E-64 | 1.22E-63 |
| NEK2 | 0.688047 | 2.953186 | 2.265139 | 1.00E-77 | 6.28E-76 |
| NOMO3 | 2.739836 | 0.80376 | -1.93608 | 1.98E-61 | 1.19E-60 |
| CTHRC1 | 1.337812 | 4.265187 | 2.927375 | 5.40E-81 | 1.32E-78 |
| TAF3 | 1.618554 | 2.857852 | 1.239297 | 6.42E-68 | 7.54E-67 |
| FOXD1 | 0.367521 | 1.371685 | 1.004164 | 1.90E-24 | 3.23E-24 |
| ALDH3A1 | 4.609271 | 2.710072 | -1.8992 | 7.25E-17 | 1.07E-16 |
| C9orf16 | 4.822388 | 5.839251 | 1.016863 | 5.34E-46 | 1.47E-45 |
| CRIP1 | 4.124994 | 1.816348 | -2.30865 | 1.23E-57 | 5.53E-57 |
| FZD6 | 2.294863 | 3.338486 | 1.043623 | 1.44E-55 | 5.78E-55 |
| POLR1D | 5.588117 | 4.093234 | -1.49488 | 2.69E-64 | 2.14E-63 |
| RNF112 | 1.234382 | 0.158191 | -1.07619 | 3.03E-80 | 5.37E-78 |
| PHGDH | 3.867685 | 2.493871 | -1.37381 | 8.66E-34 | 1.78E-33 |
| TENT5B | 2.56071 | 1.545717 | -1.01499 | 5.37E-18 | 8.08E-18 |
| PTS | 3.66164 | 2.530565 | -1.13108 | 6.17E-59 | 3.04E-58 |
| FNBP4 | 4.516989 | 3.389529 | -1.12746 | 8.11E-51 | 2.62E-50 |
| IQANK1 | 1.521144 | 3.622624 | 2.101481 | 6.33E-64 | 4.83E-63 |
| RPL37AP1 | 0.680928 | 3.129754 | 2.448826 | 1.08E-61 | 6.60E-61 |
| RAC2 | 3.064396 | 4.561515 | 1.497119 | 8.25E-47 | 2.33E-46 |
| TNFAIP3 | 3.009347 | 4.182131 | 1.172784 | 1.45E-41 | 3.54E-41 |
| CFL2 | 3.857963 | 2.298796 | -1.55917 | 4.96E-44 | 1.29E-43 |
| ZFHX3 | 1.499664 | 2.557919 | 1.058256 | 1.21E-54 | 4.62E-54 |
| ZNF862 | 2.280503 | 1.133054 | -1.14745 | 1.06E-62 | 7.09E-62 |
| PBK | 0.864983 | 3.40386 | 2.538877 | 2.75E-75 | 1.02E-73 |
| IGKV1-12 | 1.477474 | 2.873214 | 1.395741 | 2.10E-21 | 3.38E-21 |
| NUTM2A-AS1 | 2.808284 | 1.561013 | -1.24727 | 1.04E-58 | 5.05E-58 |
| SLC30A7 | 1.946986 | 3.350296 | 1.40331 | 6.38E-80 | 1.04E-77 |
| PRPF19 | 4.517599 | 5.586269 | 1.06867 | 5.15E-73 | 1.27E-71 |
| ZNF23 | 1.853472 | 0.177358 | -1.67611 | 2.54E-64 | 2.03E-63 |
| FMO5 | 4.016522 | 1.926873 | -2.08965 | 1.45E-21 | 2.34E-21 |
| MARVELD3 | 1.125267 | 2.252215 | 1.126948 | 3.34E-48 | 9.90E-48 |
| C9orf147 | 2.0962 | 0.351297 | -1.7449 | 3.69E-34 | 7.65E-34 |
| OARD1 | 3.639431 | 2.378322 | -1.26111 | 1.45E-53 | 5.25E-53 |
| LY86 | 1.057562 | 2.335222 | 1.27766 | 1.74E-46 | 4.87E-46 |
| AC006970.1 | 0.219932 | 1.286655 | 1.066723 | 5.66E-69 | 7.62E-68 |
| LCAT | 3.051683 | 1.617651 | -1.43403 | 6.51E-57 | 2.82E-56 |
| SPNS1 | 3.250264 | 1.223583 | -2.02668 | 1.26E-52 | 4.38E-52 |
| HLA-DRB6 | 1.298055 | 3.175726 | 1.877671 | 2.68E-37 | 5.94E-37 |
| AKR1C1 | 5.466099 | 1.567986 | -3.89811 | 1.75E-77 | 1.02E-75 |
| RNU7-45P | 0.296039 | 2.24843 | 1.952391 | 3.08E-59 | 1.56E-58 |
| MMP11 | 1.047924 | 3.568778 | 2.520854 | 2.11E-70 | 3.47E-69 |
| NFASC | 2.097794 | 1.006808 | -1.09099 | 6.36E-40 | 1.50E-39 |
| APEX2 | 2.375663 | 3.864122 | 1.488459 | 1.37E-71 | 2.67E-70 |
| RPS19P1 | 0.337621 | 1.745226 | 1.407604 | 8.89E-70 | 1.34E-68 |
| RNA5SP216 | 2.273844 | 0.002008 | -2.27184 | 1.83E-78 | 1.36E-76 |
| ZBTB33 | 1.695402 | 3.266062 | 1.57066 | 4.02E-80 | 6.93E-78 |
| POMT1 | 3.43614 | 2.267535 | -1.1686 | 1.58E-62 | 1.04E-61 |
| MTATP8P2 | 0.586608 | 3.75986 | 3.173252 | 1.20E-64 | 9.95E-64 |
| ACAP1 | 2.359169 | 1.318839 | -1.04033 | 4.50E-46 | 1.25E-45 |
| SRXN1 | 3.242347 | 1.654251 | -1.5881 | 8.82E-63 | 5.96E-62 |
| RBM4 | 4.713048 | 1.576065 | -3.13698 | 5.77E-63 | 3.97E-62 |
| AL355574.1 | 0.256583 | 1.54017 | 1.283587 | 2.79E-74 | 8.58E-73 |
| AC008755.1 | 2.628572 | 3.6657 | 1.037128 | 1.25E-66 | 1.26E-65 |
| MOB1A | 3.547367 | 5.178724 | 1.631357 | 2.39E-77 | 1.36E-75 |
| IGLV2-14 | 5.106686 | 6.575577 | 1.468891 | 5.46E-08 | 6.76E-08 |
| LRPAP1 | 5.090807 | 3.673744 | -1.41706 | 1.61E-68 | 2.05E-67 |
| SP6 | 0.456818 | 1.62556 | 1.168741 | 2.52E-52 | 8.65E-52 |
| PTPRCAP | 1.782803 | 0.002008 | -1.78079 | 1.78E-78 | 1.35E-76 |
| SPTSSA | 3.370985 | 4.472333 | 1.101349 | 1.56E-48 | 4.66E-48 |
| FURIN | 4.057069 | 5.388587 | 1.331518 | 1.34E-64 | 1.10E-63 |
| RPL34P27 | 0.563689 | 1.882842 | 1.319153 | 2.99E-62 | 1.92E-61 |
| FPR3 | 1.086195 | 3.144767 | 2.058573 | 4.23E-66 | 4.03E-65 |
| BCS1L | 4.048533 | 2.728024 | -1.32051 | 2.57E-57 | 1.14E-56 |
| SLBP | 3.680859 | 5.011991 | 1.331132 | 1.08E-78 | 1.14E-76 |
| CA4 | 2.020062 | 0.624147 | -1.39592 | 3.74E-50 | 1.18E-49 |
| RNASEH2B | 3.13137 | 2.089078 | -1.04229 | 3.84E-53 | 1.36E-52 |
| TMPRSS3 | 1.672062 | 2.722906 | 1.050845 | 2.75E-18 | 4.17E-18 |
| KLF16 | 2.254766 | 4.188658 | 1.933892 | 2.70E-71 | 5.06E-70 |
| ARSE | 1.192909 | 2.850512 | 1.657603 | 1.16E-44 | 3.08E-44 |
| NLRP1 | 2.656054 | 1.387411 | -1.26864 | 2.91E-59 | 1.48E-58 |
| ZNF662 | 1.888461 | 0.517502 | -1.37096 | 1.18E-74 | 3.88E-73 |
| KLK7 | 0.424845 | 1.618838 | 1.193993 | 5.49E-14 | 7.68E-14 |
| SNORD46 | 0.151252 | 1.200582 | 1.04933 | 3.78E-63 | 2.66E-62 |
| ADAM8 | 1.803521 | 2.938446 | 1.134925 | 8.58E-41 | 2.06E-40 |
| LNPK | 1.658199 | 2.67363 | 1.015431 | 3.51E-68 | 4.29E-67 |
| ASAP3 | 3.445125 | 1.95555 | -1.48958 | 1.75E-67 | 1.94E-66 |
| MFHAS1 | 2.078529 | 3.679569 | 1.601041 | 5.57E-78 | 3.69E-76 |
| NMRK1 | 3.587498 | 2.230768 | -1.35673 | 8.27E-63 | 5.61E-62 |
| LINC01001 | 2.767085 | 0.428322 | -2.33876 | 3.62E-59 | 1.82E-58 |
| MOCOS | 0.930193 | 2.10441 | 1.174217 | 2.06E-55 | 8.17E-55 |
| HTR1D | 0.1997 | 1.520279 | 1.320578 | 9.98E-62 | 6.15E-61 |
| ARNTL | 2.645321 | 1.47657 | -1.16875 | 2.41E-61 | 1.43E-60 |
| SRRM2 | 7.626228 | 6.267968 | -1.35826 | 3.30E-52 | 1.13E-51 |
| IFITM1 | 5.59479 | 6.894185 | 1.299395 | 8.17E-31 | 1.58E-30 |
| ELFN1-AS1 | 0.426025 | 1.954775 | 1.528749 | 4.65E-43 | 1.18E-42 |
| FKBP10 | 3.282928 | 4.31447 | 1.031542 | 3.51E-20 | 5.49E-20 |
| AC104794.4 | 1.693201 | 0.161615 | -1.53159 | 4.47E-69 | 6.08E-68 |
| ADAMTS12 | 0.592909 | 1.892022 | 1.299113 | 4.45E-55 | 1.74E-54 |
| NDUFA9 | 4.584385 | 2.213674 | -2.37071 | 3.12E-61 | 1.84E-60 |
| RRP9 | 2.719823 | 3.842683 | 1.122859 | 1.24E-65 | 1.13E-64 |
| PLAGL2 | 2.200412 | 3.845956 | 1.645544 | 1.43E-71 | 2.77E-70 |
| MIGA2 | 3.634548 | 2.416972 | -1.21758 | 1.03E-71 | 2.04E-70 |
| TRPM2-AS | 0.158848 | 1.330956 | 1.172108 | 8.56E-45 | 2.28E-44 |
| BPTFP1 | 1.744321 | 0.019355 | -1.72497 | 4.52E-68 | 5.43E-67 |
| ATRIP | 1.383035 | 0.169098 | -1.21394 | 9.84E-64 | 7.36E-63 |
| S100A6 | 7.949029 | 9.830878 | 1.881849 | 2.66E-63 | 1.91E-62 |
| AC004967.1 | 1.008834 | 3.230826 | 2.221992 | 5.36E-71 | 9.54E-70 |
| HGS | 5.137396 | 3.78831 | -1.34909 | 4.25E-55 | 1.66E-54 |
| TMPRSS4 | 1.94649 | 3.794661 | 1.84817 | 2.84E-42 | 7.08E-42 |
| AC091042.1 | 0.483742 | 1.5121 | 1.028358 | 6.68E-40 | 1.57E-39 |
| FTH1P16 | 0.717473 | 2.080256 | 1.362783 | 4.89E-64 | 3.78E-63 |
| PPFIBP2 | 3.840181 | 2.506689 | -1.33349 | 1.81E-29 | 3.40E-29 |
| IZUMO1 | 1.230445 | 0.142115 | -1.08833 | 5.27E-56 | 2.16E-55 |
| SDR39U1 | 4.508582 | 1.41776 | -3.09082 | 1.82E-70 | 3.02E-69 |
| SLC7A1 | 2.708949 | 4.421114 | 1.712164 | 7.39E-68 | 8.61E-67 |
| NUP210 | 1.886833 | 3.13788 | 1.251046 | 1.35E-33 | 2.75E-33 |
| DLGAP5 | 0.721235 | 2.861958 | 2.140722 | 5.40E-76 | 2.27E-74 |
| FUS | 5.919638 | 4.539872 | -1.37977 | 9.95E-50 | 3.09E-49 |
| AC005586.1 | 0.253694 | 1.383475 | 1.129781 | 1.19E-70 | 2.03E-69 |
| RCC1 | 2.517456 | 3.827971 | 1.310516 | 3.19E-60 | 1.74E-59 |
| SNORD12C | 0.09647 | 1.228847 | 1.132377 | 3.40E-62 | 2.18E-61 |
| LPIN2 | 2.967458 | 4.08366 | 1.116202 | 1.72E-44 | 4.55E-44 |
| IL17RC | 3.973423 | 2.705617 | -1.26781 | 2.35E-73 | 6.19E-72 |
| PDIA4 | 4.575529 | 6.546622 | 1.971092 | 5.09E-72 | 1.06E-70 |
| IGKV1-33 | 3.832621 | 1.758579 | -2.07404 | 2.46E-21 | 3.95E-21 |
| IGKV3-20 | 5.437404 | 7.654389 | 2.216985 | 1.35E-16 | 1.97E-16 |
| FHIT | 2.141343 | 1.043273 | -1.09807 | 6.10E-63 | 4.19E-62 |
| SVIL-AS1 | 2.993469 | 1.733886 | -1.25958 | 1.77E-70 | 2.95E-69 |
| MIR3176 | 0.146004 | 2.026483 | 1.880479 | 7.57E-77 | 3.82E-75 |
| TEPSIN | 3.812255 | 1.948946 | -1.86331 | 5.65E-50 | 1.77E-49 |
| EGFL8 | 2.984085 | 0.430967 | -2.55312 | 9.54E-65 | 7.97E-64 |
| DDT | 5.654351 | 3.425964 | -2.22839 | 2.03E-64 | 1.63E-63 |
| AC011462.2 | 2.844775 | 0.031939 | -2.81284 | 9.16E-66 | 8.45E-65 |
| SMG6 | 2.874407 | 1.809579 | -1.06483 | 2.18E-67 | 2.38E-66 |
| SPCS1 | 5.693446 | 4.3558 | -1.33765 | 4.83E-63 | 3.35E-62 |
| SIRT2 | 4.296882 | 3.175647 | -1.12124 | 2.59E-75 | 9.66E-74 |
| ITIH5 | 3.146945 | 1.40495 | -1.742 | 7.87E-54 | 2.89E-53 |
| CBWD3 | 2.355482 | 0.127379 | -2.2281 | 5.08E-64 | 3.92E-63 |
| RELB | 2.641377 | 3.737874 | 1.096497 | 1.51E-51 | 5.05E-51 |
| RACGAP1 | 2.064213 | 3.696418 | 1.632205 | 2.95E-72 | 6.48E-71 |
| DNLZ | 2.205153 | 0.073352 | -2.1318 | 1.00E-68 | 1.31E-67 |
| MYOC | 3.400261 | 0.65102 | -2.74924 | 2.42E-60 | 1.33E-59 |
| PRRX2 | 1.315734 | 2.551966 | 1.236232 | 4.49E-34 | 9.29E-34 |
| PLIN4 | 3.153295 | 1.250072 | -1.90322 | 1.58E-43 | 4.08E-43 |
| TTK | 0.763872 | 2.404784 | 1.640912 | 4.83E-68 | 5.78E-67 |
| RPL14 | 7.83684 | 5.846673 | -1.99017 | 9.78E-65 | 8.16E-64 |
| APOBEC2 | 2.292832 | 0.790707 | -1.50212 | 2.54E-50 | 8.05E-50 |
| GADD45G | 3.797281 | 2.322192 | -1.47509 | 2.40E-52 | 8.27E-52 |
| PFDN2 | 4.529369 | 5.795022 | 1.265653 | 1.32E-71 | 2.58E-70 |
| IGLV3-1 | 3.524015 | 4.717206 | 1.19319 | 2.01E-08 | 2.52E-08 |
| PDZD4 | 1.962022 | 0.690212 | -1.27181 | 4.23E-51 | 1.39E-50 |
| BCL2L2 | 4.311917 | 3.25888 | -1.05304 | 5.40E-65 | 4.62E-64 |
| PTX3 | 1.937096 | 0.533475 | -1.40362 | 1.03E-56 | 4.42E-56 |
| IGLV3-21 | 3.203752 | 5.603388 | 2.399636 | 5.97E-26 | 1.05E-25 |
| MMP15 | 3.538866 | 5.008815 | 1.469948 | 8.90E-43 | 2.25E-42 |
| IPO4 | 2.524242 | 1.001022 | -1.52322 | 1.76E-47 | 5.09E-47 |
| BSCL2 | 3.787267 | 1.512031 | -2.27524 | 1.71E-64 | 1.38E-63 |
| MAP3K20-AS1 | 0.225847 | 1.251362 | 1.025515 | 1.13E-32 | 2.27E-32 |
| NPW | 0.961335 | 2.009312 | 1.047978 | 2.25E-16 | 3.29E-16 |
| RPL12P47 | 0.291541 | 1.527854 | 1.236313 | 3.66E-53 | 1.30E-52 |
| MYEOV | 1.448742 | 3.122546 | 1.673804 | 6.52E-35 | 1.37E-34 |
| RAB11FIP1 | 2.590477 | 4.157906 | 1.567429 | 1.51E-46 | 4.24E-46 |
| DNAJB5 | 2.94761 | 1.619844 | -1.32777 | 5.58E-36 | 1.20E-35 |
| NACA2 | 0.481073 | 1.565504 | 1.084431 | 2.33E-62 | 1.51E-61 |
| PCBP1-AS1 | 3.252958 | 0.818451 | -2.43451 | 2.21E-79 | 3.12E-77 |
| PROCR | 3.089671 | 4.330754 | 1.241083 | 7.47E-39 | 1.71E-38 |
| MIR4665 | 0.241566 | 1.378489 | 1.136922 | 3.99E-44 | 1.05E-43 |
| GYPC | 3.851215 | 2.790587 | -1.06063 | 1.40E-25 | 2.43E-25 |
| REC8 | 4.129881 | 2.508577 | -1.6213 | 1.29E-35 | 2.75E-35 |
| AL109811.2 | 2.919384 | 1.207456 | -1.71193 | 4.70E-63 | 3.27E-62 |
| AC090114.1 | 0.601168 | 2.426545 | 1.825377 | 2.53E-62 | 1.63E-61 |
| GAL3ST2 | 0.109 | 1.157665 | 1.048665 | 1.08E-50 | 3.47E-50 |
| PPP1R14A | 4.729759 | 2.858929 | -1.87083 | 4.20E-36 | 9.08E-36 |
| LDHB | 7.107862 | 5.462124 | -1.64574 | 7.68E-51 | 2.49E-50 |
| COMMD4 | 4.424912 | 2.897923 | -1.52699 | 7.96E-60 | 4.20E-59 |
| CNTFR | 1.966688 | 0.96505 | -1.00164 | 3.62E-33 | 7.34E-33 |
| HLA-H | 2.78974 | 4.310957 | 1.521217 | 2.13E-43 | 5.47E-43 |
| AP002807.1 | 2.18956 | 1.121246 | -1.06831 | 2.26E-40 | 5.38E-40 |
| RNU4-2 | 1.750584 | 3.096129 | 1.345546 | 2.40E-18 | 3.65E-18 |
| MACC1 | 0.864711 | 3.086045 | 2.221334 | 1.63E-70 | 2.72E-69 |
| NDRG3 | 2.987022 | 4.356208 | 1.369186 | 2.99E-75 | 1.11E-73 |
| TYMP | 3.555896 | 4.992243 | 1.436347 | 1.55E-46 | 4.35E-46 |
| SNX15 | 2.571645 | 0.238443 | -2.3332 | 8.89E-65 | 7.45E-64 |
| ITPR1 | 2.736035 | 1.605241 | -1.13079 | 2.55E-48 | 7.56E-48 |
| TRMT2A | 4.320771 | 2.675126 | -1.64564 | 4.05E-58 | 1.88E-57 |
| CSNK2B | 5.786482 | 4.560167 | -1.22631 | 4.37E-52 | 1.49E-51 |
| GNG5P2 | 0.254116 | 1.49657 | 1.242454 | 5.43E-63 | 3.75E-62 |
| GRINA | 5.596995 | 6.607709 | 1.010714 | 4.96E-53 | 1.75E-52 |
| ENHO | 1.544914 | 0.470942 | -1.07397 | 4.10E-55 | 1.61E-54 |
| PBDC1 | 3.429544 | 4.521679 | 1.092135 | 3.18E-54 | 1.19E-53 |
| P2RX4 | 3.34111 | 2.328712 | -1.0124 | 1.04E-47 | 3.03E-47 |
| AC234582.1 | 1.799204 | 0.588325 | -1.21088 | 2.10E-58 | 9.95E-58 |
| FAM83H | 2.356175 | 5.630266 | 3.274091 | 9.17E-75 | 3.12E-73 |
| SLC35F6 | 2.808871 | 4.483986 | 1.675116 | 1.75E-69 | 2.53E-68 |
| RAP1GAP | 4.31262 | 2.69934 | -1.61328 | 8.66E-17 | 1.28E-16 |
| RPL23AP42 | 7.30976 | 4.161175 | -3.14858 | 1.71E-58 | 8.13E-58 |
| TNFRSF14 | 5.282479 | 3.54055 | -1.74193 | 3.58E-58 | 1.67E-57 |
| RBPJL | 1.371014 | 0.111092 | -1.25992 | 1.07E-49 | 3.33E-49 |
| WASH9P | 3.230524 | 1.396501 | -1.83402 | 6.43E-61 | 3.69E-60 |
| AGAP4 | 2.541119 | 0.832823 | -1.7083 | 1.97E-54 | 7.44E-54 |
| MIR1-1HG-AS1 | 1.438602 | 0.243539 | -1.19506 | 8.52E-63 | 5.76E-62 |
| KPNA6 | 2.989831 | 4.056458 | 1.066627 | 5.82E-66 | 5.46E-65 |
| MOCS2 | 3.797271 | 2.600367 | -1.1969 | 1.21E-73 | 3.36E-72 |
| EP400P1 | 1.966633 | 0.894317 | -1.07232 | 1.76E-53 | 6.36E-53 |
| FAM156A | 2.780705 | 0.060297 | -2.72041 | 1.59E-71 | 3.06E-70 |
| ZNF75A | 2.58596 | 1.274494 | -1.31147 | 3.33E-62 | 2.13E-61 |
| AC020656.1 | 2.693609 | 0.002008 | -2.6916 | 1.80E-78 | 1.35E-76 |
| TMSB4XP1 | 0.818044 | 2.169896 | 1.351852 | 1.62E-32 | 3.24E-32 |
| CCN5 | 2.239351 | 0.325378 | -1.91397 | 1.17E-77 | 7.16E-76 |
| N4BP2L1 | 2.663933 | 1.59908 | -1.06485 | 2.28E-60 | 1.26E-59 |
| EML4 | 3.119849 | 4.157444 | 1.037595 | 2.14E-65 | 1.90E-64 |
| ANO6 | 2.751563 | 3.81468 | 1.063117 | 7.12E-36 | 1.53E-35 |
| FABP5 | 5.173842 | 3.977475 | -1.19637 | 1.81E-29 | 3.39E-29 |
| INPP5B | 2.75808 | 1.704974 | -1.05311 | 8.52E-63 | 5.76E-62 |
| GRIP2 | 1.81233 | 0.211275 | -1.60105 | 2.59E-66 | 2.52E-65 |
| KCTD15 | 3.14007 | 1.989581 | -1.15049 | 1.59E-45 | 4.35E-45 |
| IFIT5 | 1.705587 | 2.813363 | 1.107777 | 5.75E-58 | 2.64E-57 |
| ZNF559 | 2.31153 | 1.220216 | -1.09131 | 2.31E-59 | 1.18E-58 |
| SUMO2P1 | 0.844796 | 2.006571 | 1.161776 | 1.07E-48 | 3.20E-48 |
| ISY1-RAB43 | 1.731695 | 0.01242 | -1.71927 | 2.64E-69 | 3.72E-68 |
| NRBP2 | 4.130471 | 2.900219 | -1.23025 | 6.29E-53 | 2.22E-52 |
| NPIPB15 | 2.344577 | 1.343453 | -1.00112 | 1.15E-16 | 1.69E-16 |
| DNALI1 | 2.797479 | 1.608279 | -1.1892 | 1.27E-40 | 3.02E-40 |
| IPMK | 1.074027 | 2.284927 | 1.210901 | 3.54E-68 | 4.32E-67 |
| UBTD2 | 2.295219 | 3.424471 | 1.129252 | 4.00E-64 | 3.12E-63 |
| UGGT1 | 2.430078 | 3.877513 | 1.447436 | 2.55E-81 | 7.02E-79 |
| HSPA5 | 5.77213 | 7.713345 | 1.941215 | 7.91E-76 | 3.20E-74 |
| POLR2K | 4.310168 | 5.371995 | 1.061827 | 6.50E-65 | 5.53E-64 |
| MFSD4A | 4.246749 | 1.754867 | -2.49188 | 2.66E-33 | 5.41E-33 |
| MPZ | 1.929674 | 0.748509 | -1.18117 | 2.29E-62 | 1.48E-61 |
| CIAO3 | 3.229902 | 1.80792 | -1.42198 | 9.00E-62 | 5.56E-61 |
| MLPH | 5.238327 | 3.316197 | -1.92213 | 2.02E-23 | 3.36E-23 |
| CIRBP | 7.011288 | 3.996334 | -3.01495 | 2.09E-75 | 7.87E-74 |
| TEAD4 | 2.006857 | 3.649118 | 1.64226 | 2.09E-67 | 2.30E-66 |
| CDR2L | 2.986763 | 4.447419 | 1.460656 | 2.31E-53 | 8.29E-53 |
| TRRAP | 2.179819 | 3.558025 | 1.378206 | 1.08E-77 | 6.69E-76 |
| SKP1 | 6.585816 | 3.837249 | -2.74857 | 5.75E-70 | 8.92E-69 |
| CXCL9 | 0.867669 | 4.368668 | 3.500999 | 1.66E-73 | 4.45E-72 |
| DPY19L1 | 1.932424 | 3.434878 | 1.502454 | 2.27E-80 | 4.48E-78 |
| LGALS2 | 1.44973 | 3.855416 | 2.405687 | 5.10E-51 | 1.66E-50 |
| CARS | 3.759283 | 2.607883 | -1.1514 | 6.62E-58 | 3.03E-57 |
| ZNF148 | 1.723045 | 2.821611 | 1.098566 | 9.64E-69 | 1.27E-67 |
| IGLC2 | 7.210628 | 8.381705 | 1.171076 | 0.000286 | 0.000323 |
| AKR1B10 | 5.737293 | 4.322527 | -1.41477 | 1.41E-10 | 1.84E-10 |
| PTPRZ1 | 2.404554 | 0.608098 | -1.79646 | 7.72E-63 | 5.24E-62 |
| SLC27A3 | 4.323377 | 2.450492 | -1.87289 | 2.18E-62 | 1.41E-61 |
| RPL23AP2 | 0.875615 | 2.027083 | 1.151468 | 1.18E-56 | 5.02E-56 |
| ITGA10 | 1.597551 | 0.37332 | -1.22423 | 7.79E-65 | 6.56E-64 |
| BCL2L2-PABPN1 | 1.752299 | 0.31423 | -1.43807 | 1.39E-50 | 4.46E-50 |
| NFKBIE | 2.106734 | 3.413775 | 1.307041 | 2.32E-64 | 1.85E-63 |
| HINT2 | 4.466738 | 3.076704 | -1.39003 | 7.38E-64 | 5.58E-63 |
| TSPAN8 | 5.529234 | 7.215345 | 1.686112 | 2.11E-18 | 3.20E-18 |
| APMAP | 4.058417 | 5.417578 | 1.359161 | 1.40E-83 | 1.56E-80 |
| AC074143.2 | 1.712088 | 0.006373 | -1.70571 | 1.59E-71 | 3.06E-70 |
| AL162151.2 | 5.888804 | 4.152246 | -1.73656 | 5.86E-38 | 1.32E-37 |
| SULT1A4 | 2.184307 | 0.016946 | -2.16736 | 7.12E-64 | 5.40E-63 |
| TNS4 | 0.715323 | 3.517819 | 2.802496 | 2.94E-54 | 1.10E-53 |
| LINC02041 | 0.623977 | 1.793237 | 1.16926 | 2.47E-38 | 5.60E-38 |
| EIF4A1 | 6.265523 | 1.58151 | -4.68401 | 1.43E-55 | 5.74E-55 |
| PI3 | 1.836523 | 6.126693 | 4.29017 | 1.23E-50 | 3.96E-50 |
| OR2I1P | 0.549838 | 3.623849 | 3.074012 | 2.18E-74 | 6.82E-73 |
| TRIM34 | 1.405368 | 0.200127 | -1.20524 | 9.47E-68 | 1.09E-66 |
| MSX2 | 0.721132 | 2.636825 | 1.915693 | 1.21E-69 | 1.78E-68 |
| AAMDC | 4.390392 | 2.442009 | -1.94838 | 4.83E-75 | 1.72E-73 |
| CLSTN1 | 4.359574 | 5.739066 | 1.379492 | 4.07E-68 | 4.92E-67 |
| SATB1 | 3.003726 | 1.706047 | -1.29768 | 3.11E-60 | 1.70E-59 |
| REEP6 | 1.786505 | 3.504228 | 1.717723 | 1.04E-41 | 2.55E-41 |
| AC083798.2 | 2.216629 | 0.558042 | -1.65859 | 3.52E-68 | 4.30E-67 |
| POLR2J3 | 4.160944 | 0.586146 | -3.5748 | 3.04E-56 | 1.27E-55 |
| ST6GALNAC6 | 4.250241 | 2.492193 | -1.75805 | 1.46E-72 | 3.37E-71 |
| FABP4 | 4.083803 | 1.249848 | -2.83396 | 2.17E-67 | 2.38E-66 |
| LINC02381 | 4.78247 | 3.261612 | -1.52086 | 1.65E-27 | 2.97E-27 |
| ELN | 4.533022 | 3.357218 | -1.1758 | 2.00E-20 | 3.14E-20 |
| ARHGAP33 | 2.769211 | 1.54641 | -1.2228 | 2.67E-51 | 8.82E-51 |
| NAGK | 4.317284 | 2.736054 | -1.58123 | 3.75E-70 | 5.99E-69 |
| H3F3AP6 | 0.54413 | 2.285846 | 1.741716 | 6.97E-70 | 1.06E-68 |
| HERC2P3 | 2.714239 | 0.370397 | -2.34384 | 2.62E-66 | 2.55E-65 |
| FLT3LG | 2.313041 | 0.647969 | -1.66507 | 1.58E-73 | 4.26E-72 |
| AC023157.1 | 0.656289 | 2.036878 | 1.38059 | 4.61E-57 | 2.01E-56 |
| SF3B5 | 5.642803 | 6.69123 | 1.048427 | 9.19E-66 | 8.48E-65 |
| FBLN5 | 4.426235 | 3.216728 | -1.20951 | 3.47E-30 | 6.61E-30 |
| ERVK3-1 | 3.68162 | 2.067864 | -1.61376 | 1.48E-53 | 5.35E-53 |
| CNOT6 | 2.085011 | 3.172119 | 1.087108 | 9.41E-74 | 2.66E-72 |
| FAM83D | 3.10896 | 4.36533 | 1.25637 | 2.04E-16 | 2.98E-16 |
| TNFRSF11B | 0.544704 | 1.894415 | 1.34971 | 5.68E-48 | 1.67E-47 |
| ADSSL1 | 2.514407 | 0.879533 | -1.63487 | 1.43E-62 | 9.47E-62 |
| RHOF | 2.30513 | 1.260078 | -1.04505 | 1.07E-37 | 2.39E-37 |
| RBP7 | 2.726529 | 1.513995 | -1.21253 | 1.54E-48 | 4.59E-48 |
| STX4 | 4.405895 | 3.335198 | -1.0707 | 1.00E-59 | 5.25E-59 |
| TMTC3 | 1.419371 | 2.569211 | 1.14984 | 4.53E-65 | 3.89E-64 |
| AC060780.3 | 0.162525 | 1.597971 | 1.435447 | 7.82E-58 | 3.56E-57 |
| RNF6 | 2.6499 | 3.763404 | 1.113504 | 3.18E-74 | 9.66E-73 |
| RPL37 | 8.219589 | 6.540103 | -1.67949 | 2.28E-54 | 8.59E-54 |
| ENO3 | 2.483567 | 0.908359 | -1.57521 | 3.29E-58 | 1.54E-57 |
| AFG3L1P | 2.850076 | 1.308739 | -1.54134 | 1.97E-50 | 6.29E-50 |
| DKC1 | 3.372346 | 4.407866 | 1.035521 | 3.00E-67 | 3.24E-66 |
| FYN | 3.983546 | 2.716264 | -1.26728 | 2.71E-59 | 1.38E-58 |
| DBNDD2 | 4.523288 | 2.12174 | -2.40155 | 6.69E-67 | 6.95E-66 |
| ZNF638 | 4.458962 | 2.902914 | -1.55605 | 4.17E-55 | 1.63E-54 |
| HPN | 3.518379 | 1.104693 | -2.41369 | 7.94E-39 | 1.82E-38 |
| TOP2A | 1.521845 | 4.868543 | 3.346698 | 7.62E-81 | 1.75E-78 |
| MIR5587 | 0.120128 | 1.856833 | 1.736704 | 1.14E-60 | 6.45E-60 |
| C5orf51 | 2.151774 | 3.455665 | 1.303891 | 2.97E-71 | 5.50E-70 |
| IGHV3-11 | 2.860552 | 4.280227 | 1.419675 | 6.77E-13 | 9.29E-13 |
| JUND | 6.264592 | 7.436341 | 1.171749 | 1.55E-35 | 3.32E-35 |
| LTF | 3.884278 | 2.636886 | -1.24739 | 1.28E-08 | 1.61E-08 |
| Z74021.1 | 1.975055 | 3.68392 | 1.708865 | 3.00E-55 | 1.18E-54 |
| DSP | 3.435025 | 5.472075 | 2.03705 | 1.16E-56 | 4.96E-56 |
| AL583805.1 | 0.248468 | 1.36422 | 1.115752 | 9.12E-62 | 5.63E-61 |
| SKA1 | 0.492523 | 2.097097 | 1.604574 | 9.57E-75 | 3.23E-73 |
| ATP1A2 | 2.310823 | 0.743127 | -1.5677 | 1.54E-46 | 4.32E-46 |
| SLC25A27 | 3.406933 | 0.83692 | -2.57001 | 5.70E-65 | 4.86E-64 |
| ZBED5 | 3.355704 | 2.299969 | -1.05573 | 4.62E-56 | 1.90E-55 |
| NALT1 | 1.649651 | 0.566379 | -1.08327 | 3.39E-40 | 8.03E-40 |
| RPL13P12 | 4.258609 | 6.125616 | 1.867007 | 5.72E-25 | 9.85E-25 |
| NCAPH | 0.892771 | 2.858875 | 1.966103 | 1.26E-72 | 2.95E-71 |
| TPSB2 | 4.780852 | 2.781618 | -1.99923 | 1.16E-44 | 3.09E-44 |
| HNRNPAB | 5.218636 | 6.672849 | 1.454212 | 4.00E-78 | 2.74E-76 |
| AC233968.1 | 1.464429 | 0.068782 | -1.39565 | 1.21E-56 | 5.16E-56 |
| SLC52A3 | 1.427578 | 3.12875 | 1.701172 | 8.02E-63 | 5.45E-62 |
| LGR4 | 3.042599 | 4.204117 | 1.161518 | 2.13E-49 | 6.54E-49 |
| IRX3 | 2.626433 | 0.935479 | -1.69095 | 9.40E-37 | 2.06E-36 |
| RBM38 | 3.453904 | 4.689294 | 1.235389 | 7.34E-38 | 1.64E-37 |
| SPPL2B | 4.516218 | 3.316181 | -1.20004 | 8.17E-49 | 2.46E-48 |
| PLA2G4A | 1.666351 | 2.750344 | 1.083993 | 2.50E-28 | 4.59E-28 |
| TANGO2 | 3.252191 | 2.192723 | -1.05947 | 8.74E-67 | 9.00E-66 |
| POLI | 2.820508 | 1.15687 | -1.66364 | 4.97E-64 | 3.84E-63 |
| MIR200A | 0.200113 | 1.268004 | 1.067891 | 8.40E-52 | 2.84E-51 |
| NFATC4 | 4.128181 | 1.352025 | -2.77616 | 3.33E-69 | 4.62E-68 |
| IGHV3-20 | 1.173321 | 2.540459 | 1.367138 | 6.37E-26 | 1.12E-25 |
| ALYREF | 3.994676 | 5.075746 | 1.08107 | 1.41E-64 | 1.16E-63 |
| DEPDC1 | 0.473628 | 2.062335 | 1.588706 | 3.63E-72 | 7.85E-71 |
| PMS2P6 | 1.712392 | 0.070836 | -1.64156 | 8.70E-60 | 4.58E-59 |
| ERP44 | 3.241334 | 4.620491 | 1.379157 | 1.19E-66 | 1.20E-65 |
| PPIE | 3.724902 | 2.089382 | -1.63552 | 6.40E-68 | 7.52E-67 |
| SARM1 | 2.122375 | 0.889297 | -1.23308 | 1.36E-69 | 2.00E-68 |
| LTB | 1.384802 | 3.128735 | 1.743934 | 6.26E-49 | 1.90E-48 |
| SULT1A3 | 2.450701 | 0.083941 | -2.36676 | 9.16E-62 | 5.65E-61 |
| MICAL3 | 2.416816 | 1.400376 | -1.01644 | 2.27E-43 | 5.83E-43 |
| CHMP4C | 2.435818 | 3.898886 | 1.463068 | 1.35E-42 | 3.40E-42 |
| FOXJ1 | 0.335638 | 1.735251 | 1.399612 | 2.03E-15 | 2.92E-15 |
| DENND5A | 3.613204 | 2.489772 | -1.12343 | 4.24E-43 | 1.08E-42 |
| GTF2IP4 | 3.645224 | 1.975788 | -1.66944 | 1.38E-64 | 1.13E-63 |
| APOBEC3C | 2.794971 | 4.033847 | 1.238876 | 3.86E-38 | 8.70E-38 |
| AKIRIN2 | 3.363681 | 4.629978 | 1.266297 | 3.46E-64 | 2.72E-63 |
| SUN2 | 5.3227 | 4.174271 | -1.14843 | 8.94E-64 | 6.71E-63 |
| MFF | 4.420213 | 3.392205 | -1.02801 | 3.04E-64 | 2.41E-63 |
| MTATP6P2 | 0.178377 | 1.356135 | 1.177758 | 2.16E-66 | 2.12E-65 |
| ALDH1B1 | 3.270518 | 4.648021 | 1.377503 | 2.09E-28 | 3.85E-28 |
| FYB1 | 1.181057 | 2.866551 | 1.685494 | 7.15E-64 | 5.43E-63 |
| CTCF | 3.076546 | 4.263432 | 1.186887 | 6.43E-77 | 3.31E-75 |
| ERCC5 | 3.702591 | 2.130093 | -1.5725 | 3.14E-56 | 1.31E-55 |
| DDX58 | 1.799036 | 2.909969 | 1.110933 | 1.84E-57 | 8.20E-57 |
| REX1BD | 4.336548 | 3.016065 | -1.32048 | 6.15E-60 | 3.28E-59 |
| CENPB | 4.348468 | 5.61088 | 1.262411 | 2.72E-56 | 1.14E-55 |
| ZBTB48 | 3.258338 | 1.993025 | -1.26531 | 2.08E-67 | 2.29E-66 |
| STC1 | 1.944238 | 3.345876 | 1.401638 | 6.44E-42 | 1.59E-41 |
| JPT2 | 3.42283 | 4.584744 | 1.161914 | 8.79E-76 | 3.51E-74 |
| LYAR | 2.292644 | 3.672959 | 1.380315 | 1.12E-73 | 3.13E-72 |
| RGMB-AS1 | 1.587909 | 0.445482 | -1.14243 | 2.09E-67 | 2.30E-66 |
| MIR3682 | 0.200559 | 1.835014 | 1.634455 | 7.42E-65 | 6.25E-64 |
| DSN1 | 2.008219 | 3.293985 | 1.285766 | 1.91E-73 | 5.07E-72 |
| EGLN2 | 4.059097 | 1.783502 | -2.27559 | 7.13E-60 | 3.78E-59 |
| ATP5PO | 6.378015 | 4.07217 | -2.30585 | 3.12E-73 | 8.04E-72 |
| ERAP2 | 1.906136 | 2.940518 | 1.034381 | 5.34E-17 | 7.89E-17 |
| SLC25A4 | 5.018523 | 2.705223 | -2.3133 | 2.33E-76 | 1.05E-74 |
| NME2P1 | 0.570336 | 2.571425 | 2.00109 | 6.29E-76 | 2.60E-74 |
| MATR3 | 2.893098 | 0.419523 | -2.47357 | 1.39E-55 | 5.58E-55 |
| SMIM15 | 3.265691 | 4.4775 | 1.211809 | 4.68E-68 | 5.62E-67 |
| EFHD1 | 2.736436 | 1.671845 | -1.06459 | 4.15E-39 | 9.59E-39 |
| INTS6-AS1 | 1.172538 | 0.158404 | -1.01413 | 2.55E-65 | 2.25E-64 |
| CLHC1 | 1.776801 | 0.613497 | -1.1633 | 3.00E-58 | 1.41E-57 |
| AUNIP | 0.459596 | 1.460935 | 1.001339 | 5.80E-66 | 5.44E-65 |
| TRAF3IP2-AS1 | 1.550301 | 0.54979 | -1.00051 | 2.75E-65 | 2.41E-64 |
| CENPA | 0.717565 | 2.475239 | 1.757674 | 6.81E-70 | 1.04E-68 |
| OBSL1 | 4.198174 | 1.886695 | -2.31148 | 1.58E-63 | 1.16E-62 |
| CHEK1 | 1.104171 | 2.163822 | 1.05965 | 1.95E-64 | 1.57E-63 |
| TMEM80 | 3.775386 | 2.361267 | -1.41412 | 2.10E-68 | 2.64E-67 |
| ZACN | 1.782682 | 0.151479 | -1.6312 | 4.27E-60 | 2.30E-59 |
| RPIA | 2.829367 | 3.933829 | 1.104461 | 6.84E-62 | 4.27E-61 |
| RNF2 | 1.739681 | 2.817247 | 1.077566 | 1.70E-74 | 5.44E-73 |
| AL513327.1 | 2.242043 | 0.573714 | -1.66833 | 4.06E-54 | 1.51E-53 |
| RGS11 | 2.889785 | 0.599188 | -2.2906 | 2.85E-76 | 1.25E-74 |
| EIF3F | 6.258206 | 4.219777 | -2.03843 | 7.14E-77 | 3.64E-75 |
| PVR | 3.101775 | 4.535174 | 1.433399 | 6.10E-73 | 1.50E-71 |
| IGLV2-11 | 4.113798 | 5.5342 | 1.420401 | 3.47E-09 | 4.41E-09 |
| COX6A1P2 | 2.371801 | 4.111373 | 1.739572 | 3.69E-57 | 1.62E-56 |
| AC005884.2 | 0.405937 | 1.967642 | 1.561705 | 1.34E-58 | 6.44E-58 |
| MTURN | 3.383577 | 2.171663 | -1.21191 | 1.14E-52 | 3.98E-52 |
| GRN | 5.812312 | 7.484862 | 1.67255 | 7.91E-76 | 3.20E-74 |
| AL449212.1 | 0.201005 | 1.488943 | 1.287938 | 1.71E-76 | 7.95E-75 |
| DCAF12 | 2.729037 | 4.273253 | 1.544216 | 2.23E-74 | 6.96E-73 |
| ZNF460 | 0.743434 | 1.96622 | 1.222786 | 1.13E-63 | 8.38E-63 |
| ZNF777 | 2.222344 | 3.305653 | 1.083309 | 1.40E-62 | 9.23E-62 |
| EIF4EBP3 | 4.082518 | 2.365628 | -1.71689 | 5.91E-62 | 3.71E-61 |
| SLC25A24 | 2.532452 | 3.730926 | 1.198473 | 3.38E-65 | 2.94E-64 |
| TDRP | 1.820023 | 0.766916 | -1.05311 | 1.66E-56 | 7.05E-56 |
| MMP23B | 2.299263 | 0.413336 | -1.88593 | 4.48E-68 | 5.39E-67 |
| CRADD | 2.588865 | 1.374496 | -1.21437 | 5.67E-80 | 9.49E-78 |
| CCDC136 | 1.874682 | 0.737924 | -1.13676 | 1.28E-44 | 3.40E-44 |
| TNKS1BP1 | 5.422879 | 4.346315 | -1.07656 | 4.61E-60 | 2.48E-59 |
| 5-Sep | 2.922995 | 1.172904 | -1.75009 | 3.29E-66 | 3.16E-65 |
| DHCR7 | 2.983698 | 4.261985 | 1.278287 | 7.22E-44 | 1.88E-43 |
| IGHG4 | 2.620134 | 6.464634 | 3.8445 | 5.42E-55 | 2.11E-54 |
| DEF8 | 4.099436 | 2.570347 | -1.52909 | 3.99E-69 | 5.46E-68 |
| MYL12B | 6.898528 | 8.17818 | 1.279652 | 4.96E-70 | 7.75E-69 |
| EML2 | 3.643398 | 2.525394 | -1.118 | 1.88E-50 | 6.01E-50 |
| USP34 | 3.906714 | 2.623598 | -1.28312 | 4.35E-59 | 2.18E-58 |
| GDA | 0.620354 | 2.202367 | 1.582013 | 1.70E-45 | 4.63E-45 |
| TAF1C | 4.222455 | 2.868431 | -1.35402 | 3.54E-54 | 1.32E-53 |
| GABARAP | 7.257757 | 4.103856 | -3.1539 | 1.22E-73 | 3.39E-72 |
| RCOR3 | 3.535165 | 2.451945 | -1.08322 | 1.55E-61 | 9.38E-61 |
| LMCD1 | 3.639705 | 1.790881 | -1.84882 | 2.23E-57 | 9.88E-57 |
| LETMD1 | 4.612928 | 3.121705 | -1.49122 | 1.68E-59 | 8.68E-59 |
| HLA-DPB1 | 4.484064 | 6.079169 | 1.595105 | 3.15E-38 | 7.13E-38 |
| ZNF540 | 1.361294 | 0.291271 | -1.07002 | 2.22E-76 | 1.02E-74 |
| SNORD13E | 0.179663 | 1.31898 | 1.139317 | 2.25E-58 | 1.06E-57 |
| TMEM160 | 3.172833 | 4.566544 | 1.393711 | 8.26E-58 | 3.75E-57 |
| TMEM259 | 6.015433 | 5.010734 | -1.0047 | 3.33E-59 | 1.68E-58 |
| INTS7 | 1.663231 | 2.940871 | 1.27764 | 1.73E-80 | 3.52E-78 |
| SAFB2 | 4.256617 | 3.235221 | -1.0214 | 1.31E-58 | 6.29E-58 |
| FAM3B | 4.241325 | 2.42083 | -1.8205 | 2.70E-26 | 4.76E-26 |
| C5orf56 | 2.334217 | 1.072 | -1.26222 | 1.12E-68 | 1.46E-67 |
| CPD | 2.77632 | 4.081162 | 1.304842 | 4.02E-61 | 2.35E-60 |
| VIL1 | 0.97261 | 4.135904 | 3.163294 | 3.46E-62 | 2.21E-61 |
| PRXL2B | 3.196535 | 4.756019 | 1.559484 | 4.09E-68 | 4.94E-67 |
| RPL13 | 9.700688 | 7.856804 | -1.84388 | 1.69E-60 | 9.36E-60 |
| ADNP | 3.42218 | 4.45225 | 1.03007 | 8.41E-77 | 4.20E-75 |
| RNA5SP498 | 0.321854 | 2.539621 | 2.217767 | 5.60E-63 | 3.85E-62 |
| BPNT1 | 2.590382 | 3.701879 | 1.111497 | 1.17E-62 | 7.83E-62 |
| HOXB13 | 0.173306 | 1.711084 | 1.537778 | 7.53E-57 | 3.25E-56 |
| TTLL3 | 3.819744 | 1.117243 | -2.7025 | 5.85E-60 | 3.12E-59 |
| HEPH | 2.27345 | 3.369901 | 1.096451 | 1.23E-19 | 1.90E-19 |
| CDC73 | 2.29522 | 3.522116 | 1.226895 | 2.42E-66 | 2.37E-65 |
| BLACAT1 | 0.183955 | 1.221144 | 1.037189 | 4.01E-60 | 2.17E-59 |
| AC244197.3 | 1.969716 | 0.271664 | -1.69805 | 1.67E-58 | 7.95E-58 |
| SDE2 | 2.091062 | 3.219866 | 1.128804 | 1.79E-69 | 2.58E-68 |
| COPZ2 | 3.251489 | 2.147423 | -1.10407 | 5.83E-24 | 9.83E-24 |
| RN7SL2 | 6.608635 | 7.712677 | 1.104041 | 3.67E-16 | 5.33E-16 |
| NEURL4 | 3.326225 | 1.94771 | -1.37851 | 1.18E-64 | 9.79E-64 |
| CDC6 | 0.77755 | 2.80978 | 2.03223 | 3.53E-74 | 1.07E-72 |
| WDR19 | 3.209842 | 1.379191 | -1.83065 | 5.35E-60 | 2.86E-59 |
| AP001024.1 | 0.458543 | 2.689805 | 2.231263 | 4.00E-61 | 2.34E-60 |
| SIK1 | 2.011871 | 0.544645 | -1.46723 | 2.00E-49 | 6.16E-49 |
| TMED6 | 3.16629 | 0.907081 | -2.25921 | 4.55E-30 | 8.64E-30 |
| TRIM73 | 2.563117 | 0.054936 | -2.50818 | 3.37E-77 | 1.84E-75 |
| PRSS21 | 0.672266 | 1.809305 | 1.137039 | 0.00012 | 0.000137 |
| MGAM2 | 0.358979 | 2.70179 | 2.342811 | 7.16E-61 | 4.09E-60 |
| ACSL5 | 3.304865 | 5.161675 | 1.85681 | 4.81E-51 | 1.57E-50 |
| COL16A1 | 4.250789 | 2.639872 | -1.61092 | 1.29E-46 | 3.65E-46 |
| KIAA1109 | 3.271635 | 2.223437 | -1.0482 | 3.21E-63 | 2.28E-62 |
| ATP5F1A | 6.893736 | 4.972634 | -1.9211 | 1.11E-72 | 2.62E-71 |
| RPS15A | 8.27812 | 5.679239 | -2.59888 | 2.34E-61 | 1.40E-60 |
| CLIC6 | 4.328751 | 2.20988 | -2.11887 | 3.13E-23 | 5.20E-23 |
| AC022149.1 | 3.435988 | 2.331364 | -1.10462 | 2.53E-30 | 4.83E-30 |
| FAM229A | 2.174096 | 1.056687 | -1.11741 | 1.13E-51 | 3.79E-51 |
| PNMT | 1.588078 | 0.42794 | -1.16014 | 2.77E-50 | 8.79E-50 |
| TPP1 | 4.799708 | 6.147121 | 1.347413 | 2.15E-74 | 6.73E-73 |
| WDR45 | 4.768285 | 2.981577 | -1.78671 | 2.80E-69 | 3.93E-68 |
| CXCL14 | 3.468368 | 4.941336 | 1.472968 | 4.63E-18 | 6.98E-18 |
| EPCAM | 4.290411 | 7.888289 | 3.597878 | 8.61E-75 | 2.94E-73 |
| NME2 | 6.666751 | 4.480716 | -2.18603 | 2.86E-50 | 9.05E-50 |
| CAPRIN1 | 4.391371 | 5.43794 | 1.046568 | 1.71E-74 | 5.46E-73 |
| MUC20-OT1 | 4.235242 | 1.25879 | -2.97645 | 9.41E-56 | 3.81E-55 |
| METTL27 | 0.910551 | 1.954613 | 1.044062 | 1.02E-31 | 2.00E-31 |
| MTCO2P2 | 1.115157 | 4.075655 | 2.960497 | 1.36E-62 | 9.01E-62 |
| CENPF | 1.18903 | 3.081302 | 1.892272 | 3.76E-61 | 2.20E-60 |
| ST14 | 3.654648 | 6.287985 | 2.633337 | 3.22E-68 | 3.95E-67 |
| BAG1 | 4.8935 | 3.509322 | -1.38418 | 7.32E-65 | 6.18E-64 |
| AL160408.2 | 3.611298 | 0.002008 | -3.60929 | 1.78E-78 | 1.35E-76 |
| GAPDHP1 | 0.934675 | 3.482793 | 2.548118 | 1.16E-65 | 1.06E-64 |
| ELK1 | 2.790177 | 3.904733 | 1.114556 | 8.50E-73 | 2.04E-71 |
| AC004552.1 | 0.470553 | 1.892116 | 1.421563 | 3.11E-64 | 2.46E-63 |
| ADCY4 | 3.104133 | 1.202597 | -1.90154 | 3.53E-60 | 1.92E-59 |
| NDEL1 | 3.67679 | 2.595627 | -1.08116 | 3.39E-71 | 6.26E-70 |
| GID8 | 3.231991 | 4.329212 | 1.097221 | 1.77E-75 | 6.71E-74 |
| JUP | 4.865617 | 7.610917 | 2.745299 | 6.91E-70 | 1.06E-68 |
| AP5B1 | 2.038169 | 3.132292 | 1.094123 | 4.93E-60 | 2.65E-59 |
| DCTPP1 | 3.518386 | 4.961562 | 1.443177 | 6.67E-69 | 8.90E-68 |
| CKM | 2.545102 | 0.297041 | -2.24806 | 5.82E-69 | 7.83E-68 |
| ARHGEF28 | 2.90759 | 1.264499 | -1.64309 | 6.84E-41 | 1.65E-40 |
| C12orf76 | 2.181025 | 0.848429 | -1.3326 | 8.75E-58 | 3.97E-57 |
| SLC2A4 | 2.323926 | 1.020076 | -1.30385 | 1.71E-41 | 4.18E-41 |
| DCP1A | 2.251779 | 3.402551 | 1.150772 | 1.35E-62 | 8.95E-62 |
| UGT1A6 | 2.047277 | 0.632108 | -1.41517 | 4.62E-24 | 7.81E-24 |
| KIAA1211 | 0.596291 | 2.133993 | 1.537702 | 1.12E-64 | 9.27E-64 |
| PRMT7 | 3.420008 | 1.945656 | -1.47435 | 1.60E-59 | 8.26E-59 |
| ANXA2P2 | 1.675394 | 3.919206 | 2.243812 | 6.92E-66 | 6.44E-65 |
| RPP25 | 2.062661 | 3.79653 | 1.733869 | 3.75E-72 | 8.05E-71 |
| CORO6 | 2.53927 | 0.349605 | -2.18966 | 4.31E-69 | 5.89E-68 |
| SNRPF | 3.245052 | 4.395793 | 1.15074 | 6.76E-64 | 5.15E-63 |
| PAFAH1B3 | 2.907669 | 4.477836 | 1.570167 | 2.55E-65 | 2.25E-64 |
| DRG2 | 3.354738 | 2.017379 | -1.33736 | 1.87E-69 | 2.69E-68 |
| AL031665.2 | 0.491603 | 1.734837 | 1.243234 | 2.06E-55 | 8.17E-55 |
| INCENP | 1.930783 | 3.371514 | 1.440731 | 2.91E-69 | 4.09E-68 |
| RNU6-199P | 0.095588 | 1.179311 | 1.083723 | 2.04E-55 | 8.11E-55 |
| IGKV4-1 | 4.299741 | 7.226575 | 2.926834 | 4.74E-30 | 8.99E-30 |
| RPL7P6 | 0.218467 | 1.315611 | 1.097144 | 1.47E-68 | 1.89E-67 |
| RAF1 | 4.858953 | 3.752896 | -1.10606 | 1.31E-72 | 3.05E-71 |
| C8orf59 | 5.122311 | 3.772791 | -1.34952 | 5.23E-57 | 2.28E-56 |
| GTF2IP1 | 3.830245 | 0.294863 | -3.53538 | 5.65E-58 | 2.60E-57 |
| TTC21A | 1.549655 | 0.522724 | -1.02693 | 1.79E-62 | 1.17E-61 |
| RNF138 | 2.291419 | 3.337853 | 1.046434 | 8.35E-68 | 9.66E-67 |
| DACT3 | 2.546122 | 1.499445 | -1.04668 | 3.83E-24 | 6.49E-24 |
| FABP3 | 4.145837 | 2.792409 | -1.35343 | 8.28E-32 | 1.63E-31 |
| RABIF | 1.681797 | 3.20073 | 1.518933 | 1.80E-69 | 2.60E-68 |
| ACTN4 | 6.225131 | 7.865861 | 1.640731 | 1.59E-68 | 2.03E-67 |
| TBC1D8 | 3.865151 | 2.622286 | -1.24287 | 9.72E-47 | 2.75E-46 |
| AC098591.2 | 0.478415 | 2.275716 | 1.7973 | 2.03E-66 | 2.00E-65 |
| MAMDC2 | 2.980587 | 0.922096 | -2.05849 | 3.02E-60 | 1.65E-59 |
| GFRA1 | 2.034142 | 1.007268 | -1.02687 | 6.69E-32 | 1.32E-31 |
| COA7 | 1.677419 | 2.873787 | 1.196368 | 8.79E-79 | 9.85E-77 |
| H2AFY | 4.794651 | 3.77703 | -1.01762 | 1.25E-51 | 4.21E-51 |
| IGHV3-73 | 1.855988 | 3.465377 | 1.609389 | 6.03E-20 | 9.40E-20 |
| IL1RN | 2.70074 | 3.907796 | 1.207057 | 1.02E-18 | 1.56E-18 |
| YBX1P10 | 1.26123 | 3.150489 | 1.889259 | 2.90E-63 | 2.07E-62 |
| AC025262.3 | 1.531326 | 0.002008 | -1.52932 | 1.78E-78 | 1.35E-76 |
| PSMD1 | 4.088879 | 5.166793 | 1.077914 | 6.45E-76 | 2.65E-74 |
| PARP14 | 2.47284 | 3.907028 | 1.434188 | 1.50E-72 | 3.44E-71 |
| MRPS35 | 3.829165 | 5.454511 | 1.625346 | 2.96E-77 | 1.64E-75 |
| ADCY6 | 3.966143 | 2.764996 | -1.20115 | 5.41E-51 | 1.76E-50 |
| YWHAZP2 | 0.240314 | 1.339197 | 1.098883 | 3.86E-68 | 4.68E-67 |
| STK11 | 4.070637 | 3.001781 | -1.06886 | 4.79E-63 | 3.32E-62 |
| TMEM161B-AS1 | 2.788462 | 1.012031 | -1.77643 | 1.35E-70 | 2.30E-69 |
| FNTA | 3.898415 | 2.792499 | -1.10592 | 1.66E-53 | 6.02E-53 |
| TNFSF13 | 3.866478 | 2.781792 | -1.08469 | 5.38E-39 | 1.24E-38 |
| CD27-AS1 | 3.862177 | 1.937093 | -1.92508 | 4.17E-66 | 3.98E-65 |
| POLR2F | 4.032516 | 0.040526 | -3.99199 | 3.24E-79 | 4.43E-77 |
| HEBP2 | 4.314475 | 3.286989 | -1.02749 | 7.13E-61 | 4.07E-60 |
| POLA1 | 1.42875 | 2.467582 | 1.038832 | 1.11E-75 | 4.35E-74 |
| B3GALT6 | 2.238877 | 3.373309 | 1.134432 | 1.37E-70 | 2.31E-69 |
| MRPL38 | 4.350696 | 1.942399 | -2.4083 | 4.18E-61 | 2.44E-60 |
| MIR497HG | 1.779825 | 0.182903 | -1.59692 | 6.60E-79 | 7.78E-77 |
| BTF3L4P2 | 2.94689 | 1.151472 | -1.79542 | 8.74E-55 | 3.37E-54 |
| TMEM158 | 1.065692 | 3.137943 | 2.072251 | 3.60E-67 | 3.86E-66 |
| AL161431.1 | 0.081237 | 1.123881 | 1.042643 | 1.71E-29 | 3.22E-29 |
| ETFA | 5.173012 | 3.916263 | -1.25675 | 6.56E-66 | 6.12E-65 |
| SLC6A20 | 0.684489 | 2.048353 | 1.363864 | 1.55E-29 | 2.92E-29 |
| WDR43 | 2.53498 | 4.00551 | 1.470529 | 1.63E-82 | 8.57E-80 |
| TMC7 | 0.651703 | 1.994101 | 1.342398 | 1.49E-68 | 1.91E-67 |
| AC007969.1 | 2.378586 | 5.564206 | 3.18562 | 7.77E-64 | 5.87E-63 |
| SLC31A1 | 2.768009 | 4.017271 | 1.249262 | 2.06E-55 | 8.17E-55 |
| MISP | 2.07257 | 6.262494 | 4.189924 | 1.69E-72 | 3.83E-71 |
| EZH2 | 1.520215 | 2.796814 | 1.276599 | 3.53E-59 | 1.78E-58 |
| SCN1B | 2.201162 | 0.969177 | -1.23199 | 4.69E-61 | 2.72E-60 |
| CARD8 | 2.992551 | 1.618121 | -1.37443 | 2.41E-57 | 1.07E-56 |
| TPX2 | 1.247536 | 4.885581 | 3.638045 | 1.90E-83 | 1.82E-80 |
| LAMC2 | 1.783669 | 4.704615 | 2.920946 | 2.15E-73 | 5.69E-72 |
| CNOT11 | 3.830751 | 4.833434 | 1.002684 | 2.90E-74 | 8.87E-73 |
| ZNF76 | 3.709618 | 2.185804 | -1.52381 | 1.23E-66 | 1.24E-65 |
| KANK2 | 5.085848 | 3.60743 | -1.47842 | 5.48E-43 | 1.39E-42 |
| MKNK1 | 3.911265 | 2.311807 | -1.59946 | 4.28E-66 | 4.08E-65 |
| LRP5 | 3.8763 | 5.134767 | 1.258467 | 3.24E-64 | 2.55E-63 |
| ESF1 | 2.019956 | 3.611645 | 1.591689 | 1.14E-77 | 7.04E-76 |
| AC080112.1 | 1.191159 | 2.227647 | 1.036488 | 3.12E-58 | 1.46E-57 |
| AL590139.1 | 0.315465 | 1.563146 | 1.247681 | 1.99E-68 | 2.50E-67 |
| RPL13AP6 | 0.366537 | 1.676903 | 1.310366 | 4.46E-60 | 2.40E-59 |
| NKX6-2 | 1.355193 | 0.354089 | -1.0011 | 5.26E-48 | 1.55E-47 |
| SIRPA | 2.621463 | 3.853676 | 1.232213 | 1.94E-47 | 5.58E-47 |
| MYC | 4.439102 | 5.469882 | 1.03078 | 6.73E-25 | 1.16E-24 |
| ZNF558 | 2.66454 | 1.573851 | -1.09069 | 5.88E-56 | 2.41E-55 |
| SFI1 | 3.089277 | 1.659234 | -1.43004 | 5.89E-50 | 1.84E-49 |
| ZNF446 | 2.35811 | 1.275049 | -1.08306 | 1.26E-66 | 1.27E-65 |
| TOMM20 | 4.827404 | 5.853003 | 1.025598 | 5.22E-59 | 2.59E-58 |
| AC116049.1 | 0.139716 | 1.155334 | 1.015617 | 6.22E-74 | 1.82E-72 |
| MYL6 | 8.879033 | 7.00661 | -1.87242 | 8.06E-65 | 6.78E-64 |
| KIF2A | 1.857463 | 3.010877 | 1.153414 | 2.31E-68 | 2.89E-67 |
| ORMDL1 | 4.455998 | 3.200251 | -1.25575 | 8.81E-52 | 2.97E-51 |
| EPPK1 | 1.163223 | 2.357475 | 1.194252 | 4.88E-45 | 1.31E-44 |
| TAGLN2P1 | 0.752527 | 2.188281 | 1.435754 | 3.26E-63 | 2.31E-62 |
| SCUBE2 | 3.264877 | 0.679524 | -2.58535 | 1.83E-76 | 8.45E-75 |
| TREX1 | 2.932676 | 0.002008 | -2.93067 | 1.78E-78 | 1.35E-76 |
| CCDC134 | 1.271525 | 2.356814 | 1.085289 | 6.58E-64 | 5.02E-63 |
| COX7A1 | 3.87118 | 2.644309 | -1.22687 | 1.19E-28 | 2.19E-28 |
| HPSE | 0.740247 | 1.987324 | 1.247077 | 1.32E-65 | 1.20E-64 |
| IGLV6-57 | 2.72161 | 4.580513 | 1.858904 | 4.38E-20 | 6.85E-20 |
| TMBIM4 | 5.072414 | 3.384262 | -1.68815 | 3.10E-61 | 1.82E-60 |
| RAP1B | 4.591308 | 2.474598 | -2.11671 | 2.63E-63 | 1.88E-62 |
| CSTF2 | 1.97049 | 3.500822 | 1.530333 | 3.67E-75 | 1.33E-73 |
| SLMAP | 4.108744 | 3.103664 | -1.00508 | 3.54E-29 | 6.61E-29 |
| HSD11B2 | 2.198331 | 3.66117 | 1.462839 | 3.11E-36 | 6.75E-36 |
| AP003108.2 | 3.006005 | 0.176465 | -2.82954 | 1.11E-61 | 6.80E-61 |
| SIGLEC11 | 1.983659 | 0.228528 | -1.75513 | 4.06E-67 | 4.31E-66 |
| KIF26B | 0.472443 | 1.611751 | 1.139308 | 6.88E-59 | 3.38E-58 |
| CDCA2 | 0.476398 | 2.190369 | 1.713971 | 9.86E-76 | 3.90E-74 |
| RRP1B | 2.291286 | 3.623777 | 1.332491 | 3.90E-79 | 5.16E-77 |
| DNER | 2.042942 | 0.424269 | -1.61867 | 1.45E-67 | 1.62E-66 |
| UNG | 3.126967 | 4.15322 | 1.026252 | 7.55E-59 | 3.70E-58 |
| TPM1 | 7.299725 | 4.429806 | -2.86992 | 4.75E-61 | 2.75E-60 |
| NDUFS3 | 5.219197 | 4.095932 | -1.12326 | 2.81E-64 | 2.24E-63 |
| UBD | 1.309179 | 4.066684 | 2.757504 | 1.52E-58 | 7.28E-58 |
| PTOV1 | 5.551862 | 4.318386 | -1.23348 | 2.70E-63 | 1.93E-62 |
| AL445189.2 | 0.327412 | 1.793078 | 1.465666 | 1.42E-64 | 1.16E-63 |
| ERO1B | 3.475475 | 1.672852 | -1.80262 | 7.68E-47 | 2.18E-46 |
| RNU6-118P | 0.240799 | 1.860309 | 1.61951 | 2.40E-65 | 2.12E-64 |
| HDGFL3 | 2.261073 | 1.105104 | -1.15597 | 3.01E-46 | 8.36E-46 |
| JMJD7-PLA2G4B | 1.999766 | 0.465597 | -1.53417 | 9.75E-59 | 4.73E-58 |
| GSTM5 | 2.537725 | 0.507575 | -2.03015 | 1.38E-78 | 1.35E-76 |
| CCNL1 | 5.190919 | 3.515506 | -1.67541 | 2.11E-55 | 8.37E-55 |
| FAM120A | 4.339922 | 5.898761 | 1.55884 | 9.87E-80 | 1.50E-77 |
| RPS6KA5 | 1.728641 | 0.695193 | -1.03345 | 8.61E-72 | 1.74E-70 |
| COA1 | 3.392897 | 2.07994 | -1.31296 | 1.37E-49 | 4.23E-49 |
| BTNL9 | 2.77277 | 1.362327 | -1.41044 | 8.05E-45 | 2.15E-44 |
| GPR17 | 1.438494 | 0.305117 | -1.13338 | 4.29E-63 | 3.00E-62 |
| RNF220 | 4.155549 | 2.819847 | -1.3357 | 3.22E-59 | 1.63E-58 |
| ATG4B | 4.505197 | 3.081183 | -1.42401 | 7.78E-57 | 3.35E-56 |
| NCR3LG1 | 0.644731 | 1.773146 | 1.128415 | 3.18E-55 | 1.25E-54 |
| NGRN | 4.124857 | 2.751274 | -1.37358 | 1.38E-61 | 8.40E-61 |
| ING5 | 3.021983 | 1.687509 | -1.33447 | 1.05E-59 | 5.50E-59 |
| AC159540.2 | 2.03147 | 0.024092 | -2.00738 | 1.64E-67 | 1.83E-66 |
| L3MBTL1 | 2.027187 | 0.699195 | -1.32799 | 9.36E-57 | 4.01E-56 |
| FXR1 | 4.257197 | 3.031983 | -1.22521 | 3.37E-53 | 1.20E-52 |
| VPS35L | 3.005545 | 1.987776 | -1.01777 | 6.17E-59 | 3.04E-58 |
| TTR | 2.13956 | 1.038632 | -1.10093 | 1.55E-24 | 2.64E-24 |
| NR1I2 | 0.453148 | 1.470293 | 1.017145 | 3.84E-17 | 5.69E-17 |
| RGS19 | 2.191443 | 3.486358 | 1.294916 | 4.30E-59 | 2.15E-58 |
| GNPNAT1 | 2.548971 | 3.817791 | 1.26882 | 1.67E-65 | 1.50E-64 |
| LMBR1L | 3.902267 | 2.090038 | -1.81223 | 7.84E-68 | 9.10E-67 |
| ABCD4 | 3.802072 | 2.11844 | -1.68363 | 2.83E-64 | 2.24E-63 |
| HSPB7 | 3.949982 | 2.065866 | -1.88412 | 3.63E-30 | 6.91E-30 |
| KRT13 | 2.136605 | 0.892519 | -1.24409 | 4.20E-34 | 8.70E-34 |
| IGHV3-15 | 3.350589 | 5.38786 | 2.037271 | 2.07E-19 | 3.20E-19 |
| AC098934.3 | 0.555237 | 1.993125 | 1.437888 | 5.64E-54 | 2.09E-53 |
| RPL23AP65 | 1.044189 | 2.759402 | 1.715213 | 5.91E-61 | 3.39E-60 |
| F5 | 0.775012 | 2.401755 | 1.626743 | 1.19E-37 | 2.66E-37 |
| TMEM218 | 3.04066 | 1.570235 | -1.47042 | 1.00E-71 | 2.00E-70 |
| NOC4L | 3.173515 | 4.205969 | 1.032454 | 3.54E-65 | 3.07E-64 |
| EDAR | 0.28445 | 1.334678 | 1.050228 | 3.44E-41 | 8.35E-41 |
| PSAP | 7.775673 | 8.872228 | 1.096554 | 1.07E-56 | 4.58E-56 |
| TXNL4A | 4.670395 | 3.372534 | -1.29786 | 6.55E-60 | 3.48E-59 |
| NGDN | 3.689424 | 2.418378 | -1.27105 | 2.80E-56 | 1.17E-55 |
| TMEM150B | 0.858539 | 2.455115 | 1.596577 | 2.67E-54 | 1.00E-53 |
| LUC7L2 | 4.469056 | 2.900995 | -1.56806 | 4.78E-59 | 2.38E-58 |
| CYP2W1 | 0.439386 | 2.094517 | 1.655131 | 1.32E-30 | 2.53E-30 |
| BYSL | 2.251752 | 3.312366 | 1.060614 | 1.26E-61 | 7.70E-61 |
| RPS18P9 | 1.440622 | 2.475162 | 1.034539 | 1.86E-59 | 9.59E-59 |
| MPP3 | 1.996181 | 0.989373 | -1.00681 | 6.93E-54 | 2.55E-53 |
| SI | 0.444947 | 1.614454 | 1.169506 | 7.33E-34 | 1.51E-33 |
| MAP7 | 2.158425 | 3.698761 | 1.540336 | 1.11E-56 | 4.75E-56 |
| ANKRD52 | 2.586049 | 3.702755 | 1.116706 | 1.80E-69 | 2.59E-68 |
| CCNJL | 1.709481 | 0.688783 | -1.0207 | 1.21E-37 | 2.71E-37 |
| ANKIB1 | 2.483412 | 3.599167 | 1.115755 | 3.01E-75 | 1.11E-73 |
| NOP10 | 5.724242 | 6.91756 | 1.193318 | 5.38E-68 | 6.40E-67 |
| TMEM30B | 3.007718 | 4.233479 | 1.225761 | 9.56E-32 | 1.88E-31 |
| PAX8-AS1 | 2.579199 | 1.121467 | -1.45773 | 1.93E-32 | 3.85E-32 |
| TMEM33 | 2.435933 | 3.743698 | 1.307765 | 2.34E-76 | 1.05E-74 |
| ZNF865 | 2.48151 | 3.540365 | 1.058854 | 5.38E-54 | 1.99E-53 |
| ACLY | 3.849654 | 5.147463 | 1.297809 | 7.79E-77 | 3.92E-75 |
| MLEC | 4.350147 | 5.738609 | 1.388463 | 1.92E-68 | 2.42E-67 |
| AC138207.5 | 0.376082 | 1.533043 | 1.156961 | 3.13E-67 | 3.38E-66 |
| RHOT2 | 5.374793 | 3.649585 | -1.72521 | 2.19E-58 | 1.04E-57 |
| HSP90AB1 | 7.449841 | 9.204192 | 1.754351 | 1.93E-83 | 1.82E-80 |
| TMEM185B | 2.400132 | 4.296378 | 1.896246 | 8.64E-78 | 5.47E-76 |
| AC073569.2 | 0.256268 | 1.580214 | 1.323946 | 1.04E-65 | 9.58E-65 |
| CACNB2 | 1.720365 | 0.716877 | -1.00349 | 2.96E-42 | 7.38E-42 |
| RPL22 | 6.934851 | 5.427707 | -1.50714 | 1.49E-58 | 7.13E-58 |
| TBPL1 | 3.079919 | 2.077119 | -1.0028 | 2.58E-59 | 1.32E-58 |
| SNX14 | 4.195165 | 3.115034 | -1.08013 | 1.50E-51 | 5.01E-51 |
| CBWD1 | 2.993202 | 0.81116 | -2.18204 | 1.02E-61 | 6.25E-61 |
| ZDHHC11 | 2.31585 | 0.701276 | -1.61457 | 9.43E-54 | 3.45E-53 |
| CANX | 5.792724 | 6.907843 | 1.115119 | 2.66E-69 | 3.75E-68 |
| AL157392.3 | 3.274867 | 0.648251 | -2.62662 | 5.36E-58 | 2.47E-57 |
| ITSN1 | 2.600323 | 1.556248 | -1.04408 | 2.03E-66 | 2.01E-65 |
| IFT27 | 3.183026 | 1.489686 | -1.69334 | 3.38E-75 | 1.24E-73 |
| CHGA | 5.336283 | 1.565942 | -3.77034 | 6.12E-50 | 1.92E-49 |
| LAPTM4A | 6.37753 | 7.411058 | 1.033528 | 1.46E-63 | 1.08E-62 |
| C22orf39 | 3.539316 | 2.057454 | -1.48186 | 4.90E-75 | 1.74E-73 |
| STAG3L1 | 1.735715 | 0.285738 | -1.44998 | 3.30E-61 | 1.94E-60 |
| ABCA10 | 1.741847 | 0.349548 | -1.3923 | 5.83E-70 | 9.01E-69 |
| TTC4 | 2.397808 | 0.74654 | -1.65127 | 1.46E-61 | 8.81E-61 |
| ZNF213-AS1 | 2.334907 | 1.251376 | -1.08353 | 1.80E-57 | 8.03E-57 |
| AL353813.1 | 1.901343 | 0.002008 | -1.89933 | 1.78E-78 | 1.35E-76 |
| PMEPA1 | 2.454603 | 4.847363 | 2.39276 | 2.72E-71 | 5.08E-70 |
| ABCA5 | 2.921666 | 1.142451 | -1.77922 | 2.71E-65 | 2.39E-64 |
| PARP4 | 3.344458 | 4.825805 | 1.481347 | 2.39E-69 | 3.41E-68 |
| PSMA2 | 4.924771 | 2.849479 | -2.07529 | 1.95E-56 | 8.23E-56 |
| RPS7P10 | 1.000889 | 2.345448 | 1.344559 | 3.53E-63 | 2.49E-62 |
| CHAF1B | 0.798196 | 1.862095 | 1.063899 | 9.43E-68 | 1.08E-66 |
| CES4A | 1.983361 | 0.582805 | -1.40056 | 2.80E-61 | 1.65E-60 |
| AL354740.1 | 2.24587 | 0.055465 | -2.19041 | 7.38E-73 | 1.78E-71 |
| PGA5 | 8.765521 | 0.557881 | -8.20764 | 1.13E-67 | 1.28E-66 |
| IARS2 | 3.883605 | 5.121645 | 1.23804 | 3.01E-78 | 2.11E-76 |
| AKT2 | 4.515709 | 3.03806 | -1.47765 | 5.65E-70 | 8.78E-69 |
| EP300 | 3.021301 | 4.41122 | 1.389919 | 2.63E-68 | 3.25E-67 |
| DDIAS | 0.394583 | 1.549929 | 1.155345 | 3.52E-73 | 8.95E-72 |
| ACAP3 | 4.17061 | 2.51496 | -1.65565 | 2.09E-67 | 2.30E-66 |
| TCEAL3 | 4.276648 | 2.553223 | -1.72342 | 4.74E-66 | 4.50E-65 |
| CMTM8 | 2.722564 | 3.985396 | 1.262832 | 1.78E-46 | 4.98E-46 |
| SEMA3B | 4.850332 | 3.095619 | -1.75471 | 1.11E-55 | 4.47E-55 |
| PCF11 | 3.871026 | 2.464518 | -1.40651 | 7.63E-68 | 8.87E-67 |
| GARNL3 | 1.774575 | 0.483418 | -1.29116 | 1.09E-70 | 1.87E-69 |
| PFN1P1 | 0.878692 | 3.168983 | 2.290291 | 1.38E-62 | 9.12E-62 |
| NDC80 | 0.88321 | 2.463829 | 1.580619 | 6.15E-69 | 8.24E-68 |
| AL133260.1 | 0.682775 | 2.184719 | 1.501944 | 1.25E-60 | 7.01E-60 |
| AC126755.1 | 3.405474 | 0.625779 | -2.7797 | 4.06E-66 | 3.87E-65 |
| STEAP1 | 2.332181 | 3.837392 | 1.505211 | 1.93E-51 | 6.41E-51 |
| TMOD1 | 2.877942 | 1.18394 | -1.694 | 1.27E-53 | 4.61E-53 |
| AC010343.1 | 1.676634 | 3.826424 | 2.14979 | 4.39E-58 | 2.03E-57 |
| ENTPD7 | 1.357859 | 2.73515 | 1.377291 | 3.43E-65 | 2.98E-64 |
| METTL7A | 5.462336 | 3.871341 | -1.591 | 1.27E-57 | 5.69E-57 |
| RGPD6 | 2.4277 | 0.022768 | -2.40493 | 5.96E-75 | 2.08E-73 |
| RCOR1 | 2.419931 | 3.486776 | 1.066845 | 1.84E-58 | 8.76E-58 |
| MRPS11 | 3.750006 | 2.492919 | -1.25709 | 4.70E-69 | 6.37E-68 |
| RP2 | 1.615995 | 3.250662 | 1.634667 | 4.17E-70 | 6.60E-69 |
| RIOX1 | 1.801854 | 2.806148 | 1.004293 | 4.89E-62 | 3.08E-61 |
| NSUN5P1 | 4.165597 | 1.812043 | -2.35355 | 1.99E-48 | 5.92E-48 |
| CCDC34 | 1.525369 | 2.742225 | 1.216856 | 2.09E-62 | 1.36E-61 |
| HLA-B | 7.60319 | 8.993729 | 1.39054 | 5.33E-50 | 1.67E-49 |
| INTU | 1.687798 | 0.671763 | -1.01604 | 1.18E-63 | 8.73E-63 |
| PPIAP29 | 1.067403 | 2.722763 | 1.65536 | 1.29E-69 | 1.90E-68 |
| IFIH1 | 2.0684 | 3.130065 | 1.061665 | 2.65E-57 | 1.17E-56 |
| SEC11C | 4.892763 | 3.249822 | -1.64294 | 7.40E-57 | 3.20E-56 |
| ITPR3 | 3.635529 | 4.962263 | 1.326733 | 2.35E-48 | 6.98E-48 |
| TPT1P9 | 1.346042 | 3.319742 | 1.9737 | 4.66E-56 | 1.92E-55 |
| EIF6 | 5.377419 | 6.709871 | 1.332452 | 1.21E-72 | 2.82E-71 |
| TCEA3 | 4.639114 | 3.251706 | -1.38741 | 1.69E-38 | 3.85E-38 |
| SPINK4 | 0.836739 | 2.89811 | 2.061371 | 8.57E-31 | 1.65E-30 |
| SFRP2 | 3.192029 | 4.878724 | 1.686695 | 6.66E-15 | 9.47E-15 |
| KREMEN1 | 1.898007 | 0.002008 | -1.896 | 1.78E-78 | 1.35E-76 |
| ABCC8 | 2.444771 | 0.15811 | -2.28666 | 7.90E-77 | 3.97E-75 |
| DPH7 | 3.797419 | 2.273992 | -1.52343 | 1.64E-51 | 5.47E-51 |
| PPM1H | 1.469053 | 2.600962 | 1.131909 | 8.47E-45 | 2.26E-44 |
| KDM5D | 2.4377 | 1.112806 | -1.32489 | 2.82E-17 | 4.19E-17 |
| TSN | 3.210983 | 4.383881 | 1.172899 | 1.77E-83 | 1.82E-80 |
| EIF4EBP1 | 4.63683 | 5.799285 | 1.162455 | 4.61E-44 | 1.21E-43 |
| SMDT1 | 5.513299 | 4.40535 | -1.10795 | 1.56E-73 | 4.24E-72 |
| FEN1 | 2.104244 | 4.042511 | 1.938267 | 6.57E-79 | 7.78E-77 |
| SGSH | 3.842143 | 2.708408 | -1.13373 | 1.72E-63 | 1.26E-62 |
| RPS12P23 | 0.313672 | 1.357014 | 1.043342 | 1.03E-64 | 8.59E-64 |
| SERF2 | 7.726766 | 5.524213 | -2.20255 | 9.57E-65 | 8.00E-64 |
| KCTD5 | 2.845564 | 3.915934 | 1.07037 | 6.68E-58 | 3.05E-57 |
| MIR4728 | 0.069511 | 1.132513 | 1.063002 | 2.64E-30 | 5.04E-30 |
| NUDT22 | 4.330166 | 2.649181 | -1.68099 | 1.57E-69 | 2.28E-68 |
| SMC1A | 2.836608 | 3.969691 | 1.133083 | 9.11E-74 | 2.59E-72 |
| ACAT1 | 4.879741 | 2.973651 | -1.90609 | 8.21E-77 | 4.11E-75 |
| ASB3 | 1.883796 | 0.611224 | -1.27257 | 3.37E-63 | 2.38E-62 |
| KALRN | 3.32431 | 1.512476 | -1.81183 | 3.59E-71 | 6.59E-70 |
| RPS26P15 | 0.342323 | 1.398808 | 1.056484 | 4.82E-59 | 2.40E-58 |
| DHRS7B | 3.252185 | 2.210556 | -1.04163 | 3.78E-63 | 2.66E-62 |
| ARFGEF2 | 2.78408 | 3.933995 | 1.149916 | 4.00E-68 | 4.84E-67 |
| VMO1 | 0.933158 | 2.011456 | 1.078297 | 9.72E-47 | 2.75E-46 |
| SLC25A36 | 4.109329 | 3.083737 | -1.02559 | 2.72E-50 | 8.62E-50 |
| DNAJB6 | 4.370202 | 2.592116 | -1.77809 | 6.86E-63 | 4.69E-62 |
| MT-TY | 0.924152 | 4.642202 | 3.71805 | 1.17E-53 | 4.28E-53 |
| CBFB | 2.585957 | 4.006815 | 1.420858 | 4.09E-81 | 1.03E-78 |
| MYO7B | 0.942048 | 2.656498 | 1.71445 | 1.19E-28 | 2.19E-28 |
| CACNA1C | 2.175521 | 1.084053 | -1.09147 | 2.77E-33 | 5.63E-33 |
| RFK | 2.879535 | 3.999963 | 1.120428 | 6.93E-58 | 3.17E-57 |
| RNVU1-18 | 1.687892 | 0.216619 | -1.47127 | 2.97E-60 | 1.62E-59 |
| ARNTL2 | 1.6116 | 2.907479 | 1.295879 | 1.89E-52 | 6.52E-52 |
| GULP1 | 2.77316 | 1.585612 | -1.18755 | 2.95E-57 | 1.30E-56 |
| TMED3 | 4.620916 | 2.630887 | -1.99003 | 1.63E-54 | 6.18E-54 |
| AC005261.5 | 0.612335 | 1.846151 | 1.233816 | 5.89E-70 | 9.08E-69 |
| RAD51AP1 | 0.926469 | 2.721068 | 1.794599 | 7.06E-76 | 2.89E-74 |
| PTPN11 | 3.450937 | 4.504465 | 1.053528 | 2.93E-59 | 1.49E-58 |
| ARHGEF25 | 3.766295 | 1.700579 | -2.06572 | 3.81E-54 | 1.42E-53 |
| RGPD5 | 1.599915 | 0.021947 | -1.57797 | 1.67E-74 | 5.35E-73 |
| MTND4LP30 | 0.761136 | 3.149427 | 2.388291 | 1.78E-64 | 1.44E-63 |
| FAM91A1 | 2.692976 | 3.937252 | 1.244276 | 7.03E-79 | 8.23E-77 |
| AP1S3 | 1.162015 | 2.36465 | 1.202636 | 7.23E-55 | 2.80E-54 |
| MAL | 4.372239 | 1.227083 | -3.14516 | 3.68E-69 | 5.06E-68 |
| NAGA | 2.93972 | 4.176109 | 1.236389 | 5.91E-70 | 9.10E-69 |
| NDUFA11 | 6.096051 | 2.750203 | -3.34585 | 6.70E-74 | 1.95E-72 |
| DAZ1 | 1.858196 | 0.074645 | -1.78355 | 4.08E-37 | 9.02E-37 |
| RGL2 | 5.006134 | 3.613794 | -1.39234 | 5.30E-60 | 2.84E-59 |
| HOXA11 | 0.073377 | 1.25873 | 1.185352 | 6.22E-54 | 2.29E-53 |
| GPX2 | 5.268884 | 7.997307 | 2.728422 | 2.43E-41 | 5.92E-41 |
| MTCO1P53 | 0.450792 | 2.734432 | 2.28364 | 4.80E-64 | 3.72E-63 |
| TLCD2 | 1.454719 | 2.547714 | 1.092994 | 7.59E-48 | 2.22E-47 |
| AC138969.1 | 5.162476 | 0.096908 | -5.06557 | 2.65E-67 | 2.88E-66 |
| LCN6 | 2.306767 | 0.122244 | -2.18452 | 2.50E-83 | 2.24E-80 |
| SLC35A2 | 3.052121 | 4.078777 | 1.026656 | 3.56E-60 | 1.93E-59 |
| CLCNKA | 1.401495 | 0.11345 | -1.28804 | 2.19E-73 | 5.78E-72 |
| XIST | 2.715196 | 1.02587 | -1.68933 | 1.52E-25 | 2.65E-25 |
| MIR8071-2 | 0.253391 | 1.277379 | 1.023988 | 1.23E-19 | 1.90E-19 |
| KRT80 | 0.528046 | 2.452392 | 1.924346 | 3.03E-59 | 1.54E-58 |
| RCC2 | 3.247918 | 5.651733 | 2.403815 | 9.30E-85 | 3.62E-81 |
| CD3D | 1.881683 | 3.090605 | 1.208923 | 1.05E-29 | 1.98E-29 |
| AC009065.2 | 1.12784 | 2.956546 | 1.828705 | 8.32E-56 | 3.38E-55 |
| PRPF4 | 2.611781 | 3.675372 | 1.063591 | 2.23E-76 | 1.02E-74 |
| TET3 | 1.392367 | 2.507225 | 1.114859 | 3.71E-65 | 3.21E-64 |
| AQP4 | 1.345454 | 0.080353 | -1.2651 | 1.06E-78 | 1.13E-76 |
| LINC01389 | 0.144892 | 1.173555 | 1.028663 | 6.21E-73 | 1.52E-71 |
| RPS19BP1 | 5.099214 | 3.505873 | -1.59334 | 2.11E-68 | 2.65E-67 |
| RIC3 | 2.564148 | 0.286574 | -2.27757 | 3.08E-79 | 4.25E-77 |
| PLXNA1 | 2.3503 | 3.547285 | 1.196985 | 3.21E-72 | 7.00E-71 |
| RNU6-850P | 0.148787 | 1.928839 | 1.780051 | 6.74E-74 | 1.96E-72 |
| GPT2 | 4.260533 | 2.755794 | -1.50474 | 2.84E-22 | 4.65E-22 |
| TXNDC5 | 4.819337 | 2.409161 | -2.41018 | 2.76E-67 | 2.99E-66 |
| XIAP | 2.406515 | 3.457323 | 1.050809 | 8.68E-69 | 1.15E-67 |
| FCGR3A | 1.641615 | 3.767971 | 2.126356 | 6.38E-58 | 2.92E-57 |
| AC012618.1 | 0.452872 | 2.559092 | 2.10622 | 6.27E-62 | 3.93E-61 |
| GAS5 | 6.764167 | 5.00046 | -1.76371 | 2.08E-52 | 7.19E-52 |
| SH3BP5-AS1 | 2.416626 | 1.079437 | -1.33719 | 3.68E-59 | 1.85E-58 |
| C1QTNF7 | 1.711092 | 0.6385 | -1.07259 | 9.52E-41 | 2.28E-40 |
| AC023509.1 | 1.721071 | 0.098217 | -1.62285 | 2.80E-65 | 2.45E-64 |
| TUT4 | 3.319733 | 1.623526 | -1.69621 | 7.01E-60 | 3.72E-59 |
| PHKG2 | 3.547047 | 2.255134 | -1.29191 | 5.43E-53 | 1.92E-52 |
| SH3BP5 | 2.574738 | 1.39688 | -1.17786 | 1.06E-50 | 3.42E-50 |
| AC068587.2 | 0.207349 | 1.288832 | 1.081483 | 3.26E-53 | 1.16E-52 |
| MYL9 | 8.003283 | 6.567603 | -1.43568 | 2.13E-14 | 3.00E-14 |
| PHLDB2 | 3.080547 | 1.630956 | -1.44959 | 2.84E-48 | 8.43E-48 |
| BAIAP2 | 4.510726 | 2.327845 | -2.18288 | 1.42E-70 | 2.39E-69 |
| LTO1 | 2.845178 | 1.454271 | -1.39091 | 3.26E-50 | 1.03E-49 |
| MMP7 | 0.693066 | 3.981445 | 3.288379 | 1.68E-60 | 9.33E-60 |
| PAK1IP1 | 2.189185 | 3.67368 | 1.484494 | 3.29E-77 | 1.80E-75 |
| RPL21P75 | 0.659616 | 2.110118 | 1.450502 | 5.47E-55 | 2.13E-54 |
| TCF21 | 2.244975 | 1.216664 | -1.02831 | 1.87E-51 | 6.21E-51 |
| AC009120.2 | 2.372977 | 0.960396 | -1.41258 | 9.87E-55 | 3.79E-54 |
| CDS1 | 1.746521 | 3.385345 | 1.638824 | 1.66E-47 | 4.78E-47 |
| CAMTA2 | 4.4902 | 3.459614 | -1.03059 | 9.85E-72 | 1.96E-70 |
| SLC40A1 | 3.749879 | 5.653766 | 1.903888 | 6.70E-56 | 2.73E-55 |
| SPC24 | 1.035138 | 2.078594 | 1.043457 | 5.52E-52 | 1.88E-51 |
| ALDH2 | 6.061364 | 4.16394 | -1.89742 | 5.90E-75 | 2.07E-73 |
| RPL37A | 9.440885 | 6.752935 | -2.68795 | 4.48E-63 | 3.12E-62 |
| TRIM31 | 1.848116 | 3.917183 | 2.069066 | 3.11E-38 | 7.04E-38 |
| DICER1-AS1 | 1.866732 | 0.505676 | -1.36106 | 5.53E-64 | 4.23E-63 |
| CAPN9 | 2.689284 | 1.357521 | -1.33176 | 1.37E-16 | 2.00E-16 |
| SLC16A13 | 0.869544 | 2.173758 | 1.304213 | 1.81E-63 | 1.32E-62 |
| MTMR9LP | 2.42415 | 1.387099 | -1.03705 | 2.41E-54 | 9.06E-54 |
| PYROXD2 | 2.24945 | 1.128112 | -1.12134 | 4.39E-59 | 2.19E-58 |
| AC002075.2 | 0.558546 | 2.234864 | 1.676318 | 4.29E-62 | 2.72E-61 |
| PIK3C3 | 3.148626 | 1.551474 | -1.59715 | 7.17E-65 | 6.05E-64 |
| IGF1 | 1.504928 | 0.460034 | -1.04489 | 8.03E-48 | 2.35E-47 |
| IGHV1OR15-2 | 0.445289 | 1.475727 | 1.030439 | 1.79E-44 | 4.74E-44 |
| LAMB2 | 6.650679 | 4.870536 | -1.78014 | 8.89E-62 | 5.50E-61 |
| S100A7 | 0.409269 | 1.532485 | 1.123215 | 0.00404 | 0.004408 |
| FAM160B2 | 4.659947 | 3.102649 | -1.5573 | 7.38E-64 | 5.58E-63 |
| AL022718.1 | 0.757269 | 4.357072 | 3.599803 | 3.30E-59 | 1.67E-58 |
| SKI | 3.502185 | 4.627496 | 1.125311 | 1.31E-51 | 4.39E-51 |
| GUF1 | 2.507389 | 3.516682 | 1.009292 | 3.83E-67 | 4.09E-66 |
| GPRC5C | 5.513844 | 2.8801 | -2.63374 | 3.84E-36 | 8.30E-36 |
| ABHD17C | 2.790688 | 4.800109 | 2.009421 | 7.61E-63 | 5.18E-62 |
| ASCL2 | 0.684573 | 3.356589 | 2.672016 | 2.03E-62 | 1.32E-61 |
| RPL10P9 | 2.841909 | 4.557802 | 1.715893 | 1.37E-24 | 2.34E-24 |
| SMG1P7 | 1.771764 | 0.477967 | -1.2938 | 3.40E-56 | 1.41E-55 |
| FAM84B | 3.021127 | 4.120239 | 1.099113 | 2.07E-35 | 4.41E-35 |
| ZNRF1 | 3.319328 | 2.235993 | -1.08333 | 1.62E-69 | 2.36E-68 |
| CLDN7 | 1.995939 | 6.062941 | 4.067002 | 3.38E-72 | 7.33E-71 |
| YWHAB | 5.300923 | 7.347731 | 2.046808 | 9.48E-83 | 6.80E-80 |
| DBF4 | 1.534502 | 2.61795 | 1.083448 | 1.17E-65 | 1.07E-64 |
| GNL3L | 1.632831 | 4.038032 | 2.405201 | 2.77E-78 | 1.98E-76 |
| ACTBP2 | 0.604508 | 2.562201 | 1.957693 | 1.19E-58 | 5.75E-58 |
| ZNF337 | 2.418943 | 0.80901 | -1.60993 | 2.12E-57 | 9.42E-57 |
| KCNMB1 | 2.72588 | 1.664451 | -1.06143 | 1.25E-20 | 1.97E-20 |
| PIGP | 3.104758 | 1.687376 | -1.41738 | 9.86E-69 | 1.29E-67 |
| ADARB1 | 2.700294 | 1.619486 | -1.08081 | 4.92E-43 | 1.25E-42 |
| APOC1 | 2.601465 | 4.624308 | 2.022842 | 2.24E-46 | 6.27E-46 |
| NOS2 | 0.381694 | 1.804311 | 1.422617 | 5.89E-43 | 1.49E-42 |
| SNORD60 | 0.184313 | 1.564666 | 1.380354 | 8.22E-69 | 1.09E-67 |
| SGPP2 | 2.198948 | 4.34862 | 2.149672 | 2.33E-52 | 8.04E-52 |
| LINC02575 | 0.485325 | 1.548719 | 1.063394 | 1.75E-21 | 2.82E-21 |
| SH3BP4 | 3.336032 | 4.353091 | 1.017059 | 8.10E-54 | 2.98E-53 |
| SP1 | 3.282275 | 4.680319 | 1.398044 | 3.33E-73 | 8.51E-72 |
| MT1X | 6.952846 | 3.405034 | -3.54781 | 3.12E-74 | 9.50E-73 |
| EIF4E | 3.214525 | 1.789662 | -1.42486 | 1.43E-66 | 1.43E-65 |
| CAPS | 4.074481 | 1.926843 | -2.14764 | 8.81E-59 | 4.30E-58 |
| MIR320E | 0.266391 | 2.000399 | 1.734009 | 2.93E-68 | 3.60E-67 |
| CBX7 | 4.170657 | 2.116337 | -2.05432 | 5.61E-71 | 9.96E-70 |
| CCDC84 | 3.410202 | 1.927506 | -1.4827 | 6.32E-53 | 2.22E-52 |
| IFRD1 | 4.140508 | 2.53569 | -1.60482 | 1.16E-61 | 7.06E-61 |
| C1QTNF5 | 3.027415 | 0.002008 | -3.02541 | 1.78E-78 | 1.35E-76 |
| PLK1 | 1.833941 | 3.799357 | 1.965416 | 5.72E-64 | 4.38E-63 |
| C5orf38 | 2.75434 | 0.885807 | -1.86853 | 1.28E-34 | 2.67E-34 |
| STAG2 | 2.973322 | 4.167928 | 1.194606 | 4.66E-70 | 7.31E-69 |
| PDX1 | 0.563636 | 2.791033 | 2.227397 | 7.60E-61 | 4.34E-60 |
| CHMP4B | 5.641427 | 6.756177 | 1.11475 | 1.14E-56 | 4.86E-56 |
| HERPUD1 | 5.845491 | 4.764263 | -1.08123 | 7.67E-39 | 1.76E-38 |
| HPDL | 1.055518 | 3.051115 | 1.995597 | 6.22E-59 | 3.07E-58 |
| IGHJ3 | 0.614768 | 3.28772 | 2.672952 | 5.32E-52 | 1.81E-51 |
| AC021016.3 | 1.850561 | 0.582688 | -1.26787 | 1.58E-57 | 7.09E-57 |
| RAB7A | 5.654972 | 7.094808 | 1.439836 | 5.32E-77 | 2.80E-75 |
| SIRT7 | 3.766346 | 2.5081 | -1.25825 | 9.43E-54 | 3.45E-53 |
| RPL37P23 | 0.802514 | 2.724293 | 1.921779 | 2.53E-63 | 1.82E-62 |
| OGG1 | 2.78521 | 1.725708 | -1.0595 | 1.98E-65 | 1.77E-64 |
| DTX4 | 2.440402 | 3.697844 | 1.257441 | 6.15E-51 | 2.00E-50 |
| HDDC2 | 4.2703 | 2.761443 | -1.50886 | 1.38E-63 | 1.02E-62 |
| NBPF8 | 2.383613 | 0.879004 | -1.50461 | 3.14E-58 | 1.47E-57 |
| MRPS10 | 3.231847 | 4.755827 | 1.52398 | 3.21E-70 | 5.17E-69 |
| FKBP5 | 4.997414 | 2.669774 | -2.32764 | 5.81E-55 | 2.26E-54 |
| VIP | 2.045674 | 0.819204 | -1.22647 | 3.69E-31 | 7.16E-31 |
| TNFSF10 | 3.471815 | 4.558286 | 1.086471 | 2.69E-37 | 5.96E-37 |
| CASP3 | 2.953814 | 4.161624 | 1.20781 | 5.02E-60 | 2.69E-59 |
| AMDHD2 | 2.937582 | 1.782416 | -1.15517 | 1.00E-55 | 4.04E-55 |
| HSP90AA2P | 0.363899 | 1.482286 | 1.118386 | 1.80E-74 | 5.73E-73 |
| NOTUM | 0.433331 | 1.541857 | 1.108526 | 2.02E-12 | 2.75E-12 |
| PTMAP2 | 1.487407 | 4.106715 | 2.619308 | 9.57E-75 | 3.23E-73 |
| STX6 | 1.939819 | 2.984474 | 1.044655 | 9.91E-77 | 4.85E-75 |
| AGAP5 | 1.770763 | 0.220027 | -1.55074 | 9.07E-56 | 3.68E-55 |
| ZNF471 | 1.479027 | 0.381408 | -1.09762 | 3.19E-68 | 3.92E-67 |
| TRIOBP | 4.131533 | 1.899559 | -2.23197 | 8.70E-77 | 4.32E-75 |
| S100A13 | 4.906809 | 3.105287 | -1.80152 | 3.18E-63 | 2.26E-62 |
| PSMD6 | 4.2904 | 2.831524 | -1.45888 | 2.33E-60 | 1.28E-59 |
| MIR3131 | 0.219817 | 2.000414 | 1.780597 | 3.53E-37 | 7.81E-37 |
| GLUL | 8.401649 | 5.235917 | -3.16573 | 4.07E-73 | 1.02E-71 |
| GOLGA8B | 4.250566 | 1.321337 | -2.92923 | 1.71E-54 | 6.48E-54 |
| NEK9 | 3.866898 | 2.718001 | -1.1489 | 5.44E-59 | 2.70E-58 |
| KCTD12 | 2.991323 | 4.618112 | 1.626789 | 1.73E-41 | 4.22E-41 |
| CNTN3 | 1.151212 | 0.144526 | -1.00669 | 7.70E-72 | 1.57E-70 |
| ACTC1 | 2.109203 | 0.947395 | -1.16181 | 7.44E-21 | 1.18E-20 |
| TUBE1 | 3.00248 | 1.514136 | -1.48834 | 1.35E-61 | 8.23E-61 |
| RBM23 | 4.122547 | 2.556175 | -1.56637 | 9.10E-72 | 1.83E-70 |
| CFI | 2.104377 | 3.120803 | 1.016427 | 3.28E-28 | 6.00E-28 |
| ELOVL7 | 1.349776 | 2.527905 | 1.178129 | 4.68E-52 | 1.59E-51 |
| AL021068.2 | 0.295173 | 2.099266 | 1.804093 | 1.32E-60 | 7.37E-60 |
| STX16-NPEPL1 | 1.939011 | 0.209314 | -1.7297 | 2.14E-57 | 9.53E-57 |
| GAPDHP63 | 0.205824 | 1.269998 | 1.064174 | 5.53E-64 | 4.23E-63 |
| HSPA2 | 2.776847 | 1.719095 | -1.05775 | 6.90E-30 | 1.31E-29 |
| WASH2P | 2.866257 | 1.14915 | -1.71711 | 3.07E-66 | 2.96E-65 |
| MTPN | 3.860663 | 5.386915 | 1.526252 | 3.64E-68 | 4.43E-67 |
| TRAJ31 | 0.153504 | 1.239924 | 1.086421 | 1.99E-30 | 3.81E-30 |
| NPR2 | 2.724435 | 1.707791 | -1.01664 | 2.56E-44 | 6.73E-44 |
| B4GALT5 | 3.103121 | 5.222665 | 2.119544 | 1.25E-75 | 4.84E-74 |
| RF00002 | 2.139225 | 0.161393 | -1.97783 | 4.53E-65 | 3.89E-64 |
| SLC35C1 | 2.77503 | 4.123431 | 1.348402 | 1.79E-56 | 7.53E-56 |
| SLX1B | 1.899943 | 0.047705 | -1.85224 | 1.23E-61 | 7.49E-61 |
| PLIN5 | 3.986022 | 0.287444 | -3.69858 | 4.21E-77 | 2.26E-75 |
| WBP4 | 2.375105 | 3.402709 | 1.027604 | 1.74E-59 | 8.97E-59 |
| TXNRD2 | 3.450807 | 1.746469 | -1.70434 | 7.33E-66 | 6.81E-65 |
| FAM214A | 3.60278 | 2.069722 | -1.53306 | 4.19E-73 | 1.05E-71 |
| CTSS | 3.753946 | 5.514791 | 1.760845 | 4.62E-52 | 1.58E-51 |
| RF01882 | 1.762101 | 0.002008 | -1.76009 | 1.80E-78 | 1.35E-76 |
| PUS7 | 1.672555 | 2.803233 | 1.130678 | 7.02E-66 | 6.52E-65 |
| TMEM69 | 3.12789 | 4.15381 | 1.02592 | 1.68E-71 | 3.22E-70 |
| MGAT5 | 2.680452 | 3.816159 | 1.135707 | 8.45E-59 | 4.13E-58 |
| HSPB8 | 4.586408 | 2.79904 | -1.78737 | 4.18E-30 | 7.96E-30 |
| GAPDHP65 | 0.480166 | 2.244137 | 1.76397 | 3.70E-63 | 2.61E-62 |
| DENND3 | 3.35651 | 1.581235 | -1.77527 | 2.60E-62 | 1.68E-61 |
| MDFI | 1.623535 | 3.315629 | 1.692095 | 1.04E-55 | 4.21E-55 |
| NNMT | 5.17198 | 3.965377 | -1.2066 | 3.01E-23 | 5.00E-23 |
| MUC6 | 5.312765 | 3.387218 | -1.92555 | 7.88E-12 | 1.06E-11 |
| LEO1 | 2.480382 | 3.681282 | 1.200899 | 4.11E-73 | 1.03E-71 |
| SNRPGP2 | 1.575111 | 3.293802 | 1.718691 | 4.01E-67 | 4.26E-66 |
| GTSE1 | 0.908819 | 2.509031 | 1.600212 | 3.32E-63 | 2.35E-62 |
| BANF1P3 | 0.369548 | 2.067894 | 1.698346 | 5.34E-68 | 6.35E-67 |
| RANGRF | 3.814253 | 2.292455 | -1.5218 | 1.72E-62 | 1.13E-61 |
| RPL7P9 | 3.71045 | 4.719575 | 1.009126 | 7.53E-36 | 1.62E-35 |
| TMSB4XP6 | 2.877116 | 0.002008 | -2.87511 | 1.83E-78 | 1.36E-76 |
| IMPAD1 | 2.860756 | 4.464529 | 1.603772 | 3.35E-72 | 7.27E-71 |
| MIR25 | 0.177171 | 1.936056 | 1.758886 | 9.08E-75 | 3.09E-73 |
| SMC3 | 2.973929 | 4.521174 | 1.547245 | 1.72E-77 | 1.02E-75 |
| RIPK2 | 2.0607 | 3.508076 | 1.447376 | 4.02E-82 | 1.44E-79 |
| RNY4P10 | 0.199161 | 1.95579 | 1.75663 | 2.46E-73 | 6.43E-72 |
| MGST1 | 5.268795 | 3.729343 | -1.53945 | 1.25E-39 | 2.91E-39 |
| AC024940.3 | 0.507154 | 2.115248 | 1.608094 | 5.15E-61 | 2.98E-60 |
| AP001318.2 | 0.630595 | 2.066154 | 1.435559 | 9.63E-57 | 4.12E-56 |
| KRT8P45 | 0.765686 | 2.114907 | 1.349221 | 1.48E-49 | 4.58E-49 |
| NBDY | 3.333227 | 4.429157 | 1.09593 | 9.29E-42 | 2.28E-41 |
| HNF4G | 1.129221 | 3.288224 | 2.159003 | 9.67E-64 | 7.24E-63 |
| HS2ST1 | 1.858566 | 2.920557 | 1.061991 | 1.95E-78 | 1.43E-76 |
| FTL | 9.977783 | 11.97046 | 1.99268 | 4.40E-74 | 1.31E-72 |
| YTHDF3 | 3.04302 | 4.310676 | 1.267655 | 5.12E-69 | 6.91E-68 |
| PPT2 | 3.210709 | 2.085304 | -1.12541 | 8.67E-61 | 4.93E-60 |
| HLA-DMA | 4.091297 | 5.115402 | 1.024104 | 4.94E-21 | 7.87E-21 |
| AC010132.3 | 1.679756 | 0.018845 | -1.66091 | 5.48E-63 | 3.78E-62 |
| AC005288.1 | 2.256529 | 3.592895 | 1.336366 | 1.07E-77 | 6.65E-76 |
| WDFY1 | 2.814145 | 4.032101 | 1.217956 | 7.53E-79 | 8.64E-77 |
| SULT1C2 | 3.793137 | 2.113356 | -1.67978 | 1.59E-16 | 2.33E-16 |
| DST | 4.963689 | 2.23826 | -2.72543 | 6.43E-69 | 8.60E-68 |
| AKR7L | 2.679157 | 1.503545 | -1.17561 | 2.42E-16 | 3.53E-16 |
| DNHD1 | 1.947074 | 0.555377 | -1.3917 | 1.35E-60 | 7.56E-60 |
| XPR1 | 1.486596 | 3.04246 | 1.555863 | 4.52E-75 | 1.62E-73 |
| ACTG1 | 8.927256 | 10.76217 | 1.834916 | 7.39E-79 | 8.57E-77 |
| AC125494.1 | 2.382876 | 0.100157 | -2.28272 | 2.23E-56 | 9.35E-56 |
| FAM171A1 | 2.545359 | 3.706865 | 1.161506 | 5.37E-47 | 1.53E-46 |
| FAHD2A | 3.423968 | 1.656156 | -1.76781 | 1.17E-69 | 1.73E-68 |
| LGALS9C | 2.641172 | 0.686781 | -1.95439 | 3.72E-28 | 6.80E-28 |
| ABHD17A | 4.344625 | 3.234483 | -1.11014 | 6.61E-75 | 2.30E-73 |
| CDC5L | 2.239171 | 3.486927 | 1.247756 | 3.62E-74 | 1.09E-72 |
| MGME1 | 2.256126 | 3.649952 | 1.393826 | 5.76E-79 | 7.02E-77 |
| UPK3BL1 | 3.57266 | 0.646587 | -2.92607 | 4.15E-63 | 2.90E-62 |
| ARID5B | 2.472564 | 3.534969 | 1.062405 | 5.10E-45 | 1.37E-44 |
| RPS27P21 | 0.448138 | 3.016805 | 2.568667 | 1.93E-66 | 1.91E-65 |
| RFC3 | 1.588461 | 3.528851 | 1.940391 | 7.27E-84 | 1.18E-80 |
| SMARCC1 | 3.063195 | 4.374755 | 1.31156 | 5.43E-79 | 6.71E-77 |
| INO80B | 3.441396 | 1.620565 | -1.82083 | 5.52E-63 | 3.81E-62 |
| AC068831.4 | 2.138329 | 0.002008 | -2.13632 | 1.78E-78 | 1.35E-76 |
| ADA2 | 2.317288 | 3.466161 | 1.148873 | 1.88E-31 | 3.67E-31 |
| TM9SF4 | 3.692888 | 4.925535 | 1.232647 | 3.02E-78 | 2.11E-76 |
| SNORA71C | 0.261099 | 1.689699 | 1.4286 | 1.81E-63 | 1.32E-62 |
| AL513314.2 | 1.732721 | 0.271534 | -1.46119 | 1.65E-61 | 9.94E-61 |
| RBPMS | 5.693308 | 3.431309 | -2.262 | 1.62E-60 | 9.02E-60 |
| LRRC37A16P | 0.782981 | 1.831778 | 1.048796 | 2.52E-60 | 1.39E-59 |
| ZNF281 | 1.570796 | 2.79247 | 1.221674 | 1.44E-82 | 8.57E-80 |
| DPY19L2 | 1.531136 | 0.298291 | -1.23284 | 2.52E-61 | 1.50E-60 |
| ZNF493 | 1.988169 | 0.940894 | -1.04728 | 1.67E-65 | 1.50E-64 |
| DHRS4 | 3.733228 | 2.609874 | -1.12335 | 5.20E-68 | 6.19E-67 |
| PPIL1 | 2.860468 | 4.546451 | 1.685982 | 6.23E-81 | 1.49E-78 |
| NRG4 | 1.560908 | 0.353272 | -1.20764 | 5.50E-53 | 1.94E-52 |
| GKN2 | 6.118734 | 1.910082 | -4.20865 | 6.27E-36 | 1.35E-35 |
| HMGB1P6 | 4.500074 | 2.990662 | -1.50941 | 1.97E-35 | 4.19E-35 |
| ETFDH | 3.66492 | 2.56242 | -1.1025 | 4.93E-67 | 5.17E-66 |
| DES | 7.986125 | 5.385374 | -2.60075 | 7.79E-22 | 1.26E-21 |
| AC135048.1 | 1.249847 | 0.128905 | -1.12094 | 4.19E-72 | 8.87E-71 |
| POF1B | 1.619562 | 4.529327 | 2.909765 | 1.58E-63 | 1.16E-62 |
| COPS9 | 5.612285 | 3.936477 | -1.67581 | 6.05E-70 | 9.30E-69 |
| FOXQ1 | 2.914636 | 5.366631 | 2.451995 | 1.31E-46 | 3.69E-46 |
| ACTA2 | 8.033189 | 6.468905 | -1.56428 | 4.06E-25 | 7.01E-25 |
| ADH1B | 5.062728 | 1.45234 | -3.61039 | 2.08E-69 | 2.98E-68 |
| PWP2 | 1.407991 | 0.315301 | -1.09269 | 5.49E-59 | 2.72E-58 |
| HES1 | 3.984092 | 5.690347 | 1.706255 | 8.26E-64 | 6.22E-63 |
| ARL17A | 2.035541 | 0.308535 | -1.72701 | 4.44E-62 | 2.81E-61 |
| PRIMA1 | 2.895628 | 1.062382 | -1.83325 | 2.60E-51 | 8.58E-51 |
| MYH11 | 7.252013 | 4.474599 | -2.77741 | 1.68E-32 | 3.36E-32 |
| ZNF280D | 3.100926 | 1.553666 | -1.54726 | 1.48E-63 | 1.09E-62 |
| AC093673.1 | 1.814179 | 2.824944 | 1.010765 | 4.36E-43 | 1.11E-42 |
| TTTY14 | 1.475409 | 0.375993 | -1.09942 | 5.89E-14 | 8.25E-14 |
| EPHB2 | 0.635598 | 2.817784 | 2.182186 | 1.01E-72 | 2.38E-71 |
| GSTP1 | 7.2175 | 8.254548 | 1.037047 | 1.17E-45 | 3.20E-45 |
| TNFRSF1B | 2.978512 | 4.281838 | 1.303326 | 5.39E-51 | 1.76E-50 |
| RNU6-37P | 0.327338 | 1.90249 | 1.575153 | 5.10E-60 | 2.74E-59 |
| RPL41P1 | 0.503619 | 3.650619 | 3.146999 | 1.03E-66 | 1.05E-65 |
| NORAD | 4.84819 | 6.180806 | 1.332615 | 1.34E-71 | 2.61E-70 |
| PMF1-BGLAP | 1.755887 | 0.50305 | -1.25284 | 1.37E-71 | 2.67E-70 |
| AC044860.1 | 1.289336 | 0.120149 | -1.16919 | 1.30E-67 | 1.47E-66 |
| MSLN | 1.10426 | 4.349556 | 3.245296 | 3.42E-48 | 1.01E-47 |
| SNX4 | 2.978379 | 4.046859 | 1.068481 | 2.88E-67 | 3.11E-66 |
| ASPSCR1 | 3.44296 | 1.910283 | -1.53268 | 1.03E-50 | 3.32E-50 |
| CCNF | 0.867334 | 2.439672 | 1.572338 | 7.35E-73 | 1.78E-71 |
| NPIPP1 | 3.424483 | 1.628465 | -1.79602 | 1.16E-54 | 4.44E-54 |
| ZSCAN30 | 2.227331 | 1.227262 | -1.00007 | 2.09E-61 | 1.25E-60 |
| FNDC10 | 1.38727 | 2.895844 | 1.508575 | 1.22E-47 | 3.54E-47 |
| DEPDC1B | 0.64804 | 2.392005 | 1.743966 | 4.11E-74 | 1.23E-72 |
| GJB2 | 1.492737 | 4.31879 | 2.826053 | 5.64E-68 | 6.68E-67 |
| DHPS | 4.996183 | 3.730428 | -1.26576 | 3.22E-68 | 3.95E-67 |
| ING4 | 3.983116 | 2.668561 | -1.31456 | 1.26E-71 | 2.47E-70 |
| MAVS | 3.041482 | 4.270787 | 1.229304 | 5.15E-62 | 3.24E-61 |
| GPR89B | 2.463124 | 0.781984 | -1.68114 | 7.30E-54 | 2.69E-53 |
| FARSB | 2.88681 | 4.191276 | 1.304466 | 2.66E-73 | 6.92E-72 |
| EIF2AK1 | 4.022272 | 5.484675 | 1.462403 | 1.50E-82 | 8.57E-80 |
| PLP2 | 5.696515 | 7.537555 | 1.841039 | 5.40E-61 | 3.11E-60 |
| LCOR | 1.803733 | 2.861736 | 1.058003 | 3.94E-67 | 4.20E-66 |
| CU633906.1 | 1.447962 | 0.04469 | -1.40327 | 2.79E-63 | 2.00E-62 |
| CYP4F12 | 3.029423 | 1.607485 | -1.42194 | 2.43E-36 | 5.28E-36 |
| RCOR2 | 0.628661 | 1.696543 | 1.067882 | 1.77E-42 | 4.44E-42 |
| TGOLN2 | 4.470816 | 5.747252 | 1.276436 | 3.89E-72 | 8.31E-71 |
| EIF3C | 5.83004 | 1.735362 | -4.09468 | 2.28E-61 | 1.36E-60 |
| KLRK1 | 1.467818 | 0.138627 | -1.32919 | 9.60E-62 | 5.92E-61 |
| DAPK2 | 2.016205 | 0.781997 | -1.23421 | 2.19E-54 | 8.28E-54 |
| FLCN | 3.638892 | 1.937435 | -1.70146 | 1.88E-60 | 1.04E-59 |
| ANKRD37 | 2.682841 | 1.141171 | -1.54167 | 3.18E-65 | 2.78E-64 |
| NPHP1 | 1.434898 | 0.381854 | -1.05304 | 8.62E-65 | 7.23E-64 |
| CCL5 | 2.926917 | 4.639237 | 1.71232 | 1.49E-36 | 3.26E-36 |
| ATG101 | 3.834577 | 2.7425 | -1.09208 | 6.12E-58 | 2.81E-57 |
| EEA1 | 1.561178 | 2.657354 | 1.096176 | 1.60E-62 | 1.05E-61 |
| RNU6-1016P | 0.472739 | 3.013558 | 2.540819 | 7.01E-60 | 3.72E-59 |
| ATG16L2 | 3.512135 | 1.483481 | -2.02865 | 2.81E-61 | 1.66E-60 |
| POLD4 | 5.038095 | 3.22095 | -1.81715 | 1.75E-63 | 1.28E-62 |
| ZNF706 | 5.010438 | 3.739338 | -1.2711 | 1.43E-55 | 5.74E-55 |
| GCSHP5 | 2.29209 | 0.587254 | -1.70484 | 9.24E-58 | 4.18E-57 |
| CEP63 | 2.617986 | 1.482525 | -1.13546 | 1.83E-63 | 1.33E-62 |
| NOSTRIN | 3.474081 | 2.134818 | -1.33926 | 7.44E-42 | 1.84E-41 |
| PDHA1 | 5.927018 | 4.274383 | -1.65264 | 7.12E-59 | 3.50E-58 |
| ZMAT1 | 2.138729 | 0.588029 | -1.5507 | 4.97E-67 | 5.22E-66 |
| RPS17P2 | 0.237888 | 1.322085 | 1.084197 | 3.61E-63 | 2.54E-62 |
| XRCC2 | 0.37467 | 1.515003 | 1.140333 | 2.11E-75 | 7.94E-74 |
| FP236383.2 | 6.891853 | 1.762023 | -5.12983 | 2.38E-66 | 2.33E-65 |
| FAM111B | 0.738753 | 2.381282 | 1.642529 | 1.21E-68 | 1.57E-67 |
| MAGT1 | 3.226936 | 4.502625 | 1.275688 | 5.01E-69 | 6.77E-68 |
| RPS26P8 | 0.385849 | 1.558856 | 1.173008 | 2.48E-58 | 1.17E-57 |
| MTND4P12 | 1.267299 | 5.109643 | 3.842344 | 1.30E-66 | 1.31E-65 |
| ACTN2 | 1.535577 | 0.280563 | -1.25501 | 1.43E-53 | 5.19E-53 |
| COL4A6 | 2.09479 | 0.604872 | -1.48992 | 1.14E-47 | 3.30E-47 |
| FAM117B | 1.271594 | 2.498844 | 1.22725 | 2.81E-71 | 5.23E-70 |
| ADGRG7 | 0.301404 | 1.535513 | 1.234108 | 1.61E-41 | 3.93E-41 |
| CAB39L | 3.279028 | 1.868831 | -1.4102 | 2.05E-53 | 7.37E-53 |
| MMP14 | 4.630846 | 6.018966 | 1.38812 | 3.99E-55 | 1.56E-54 |
| PRAME | 0.153723 | 1.196191 | 1.042469 | 4.74E-11 | 6.26E-11 |
| RAB25 | 4.430303 | 6.249195 | 1.818892 | 7.26E-36 | 1.56E-35 |
| NPIPA8 | 1.483561 | 0.047085 | -1.43648 | 1.02E-47 | 2.96E-47 |
| MAP6 | 1.996009 | 0.69167 | -1.30434 | 1.36E-54 | 5.20E-54 |
| CYP51A1 | 3.20225 | 1.807971 | -1.39428 | 1.06E-51 | 3.56E-51 |
| NPIPA7 | 2.277313 | 0.01488 | -2.26243 | 6.46E-69 | 8.63E-68 |
| CD81 | 7.016394 | 5.588358 | -1.42804 | 6.00E-57 | 2.60E-56 |
| GOLPH3 | 4.466512 | 5.904065 | 1.437553 | 8.45E-70 | 1.28E-68 |
| LINC00894 | 1.729499 | 0.392833 | -1.33667 | 5.10E-53 | 1.80E-52 |
| AC007182.2 | 0.339118 | 1.547966 | 1.208848 | 1.83E-68 | 2.32E-67 |
| RNASEH2A | 2.311166 | 3.614885 | 1.303719 | 5.02E-60 | 2.69E-59 |
| RRM2 | 1.535696 | 4.195773 | 2.660077 | 1.03E-69 | 1.53E-68 |
| AP1AR | 2.234036 | 3.28519 | 1.051154 | 4.02E-71 | 7.32E-70 |
| SNHG7 | 4.263629 | 2.863576 | -1.40005 | 5.81E-55 | 2.26E-54 |
| MOB2 | 4.061725 | 2.737039 | -1.32469 | 1.07E-74 | 3.57E-73 |
| EIF4EBP2 | 3.896164 | 5.007706 | 1.111542 | 1.36E-53 | 4.93E-53 |
| CCDC159 | 4.231735 | 1.658722 | -2.57301 | 1.05E-78 | 1.13E-76 |
| C12orf75 | 3.878726 | 5.072174 | 1.193448 | 1.00E-25 | 1.75E-25 |
| SREK1 | 3.815159 | 2.429805 | -1.38535 | 1.75E-53 | 6.31E-53 |
| MST1 | 2.88234 | 0.985103 | -1.89724 | 2.30E-54 | 8.67E-54 |
| PTPRN | 1.58651 | 0.302418 | -1.28409 | 1.14E-66 | 1.16E-65 |
| LPCAT3 | 4.474846 | 3.210124 | -1.26472 | 4.25E-43 | 1.08E-42 |
| CDC42EP5 | 3.621101 | 5.777939 | 2.156838 | 2.48E-44 | 6.54E-44 |
| ATP6AP1L | 1.389534 | 0.351205 | -1.03833 | 7.68E-63 | 5.22E-62 |
| AC080013.6 | 0.617332 | 2.147316 | 1.529984 | 1.50E-62 | 9.88E-62 |
| LYRM9 | 2.367898 | 0.598198 | -1.7697 | 2.86E-72 | 6.28E-71 |
| PLBD1 | 2.691439 | 5.264427 | 2.572988 | 3.94E-68 | 4.78E-67 |
| RAB6A | 4.100698 | 5.165763 | 1.065065 | 2.52E-64 | 2.01E-63 |
| CLDN3 | 0.893303 | 6.151931 | 5.258627 | 2.10E-68 | 2.64E-67 |
| SARS2 | 3.166093 | 1.428873 | -1.73722 | 4.05E-53 | 1.44E-52 |
| ULBP3 | 0.452009 | 1.491898 | 1.039889 | 2.40E-50 | 7.63E-50 |
| Z84492.1 | 2.082877 | 0.697458 | -1.38542 | 5.73E-70 | 8.89E-69 |
| FERMT1 | 1.476139 | 4.467337 | 2.991198 | 3.42E-75 | 1.25E-73 |
| MTCYBP18 | 0.52783 | 2.517996 | 1.990165 | 8.30E-64 | 6.25E-63 |
| CDT1 | 1.06335 | 3.379367 | 2.316017 | 6.21E-73 | 1.52E-71 |
| GSTT2B | 2.792562 | 0.88175 | -1.91081 | 1.55E-54 | 5.89E-54 |
| ECHDC2 | 5.878122 | 1.986282 | -3.89184 | 2.28E-77 | 1.30E-75 |
| CFD | 6.170937 | 4.215124 | -1.95581 | 2.39E-43 | 6.13E-43 |
| HMOX2 | 4.934803 | 3.618495 | -1.31631 | 1.42E-50 | 4.54E-50 |
| PIK3R2 | 2.739689 | 0.442751 | -2.29694 | 4.61E-61 | 2.68E-60 |
| CDKN1C | 4.193853 | 2.913829 | -1.28002 | 9.89E-39 | 2.26E-38 |
| AGPAT5 | 2.158011 | 3.331875 | 1.173864 | 1.25E-64 | 1.03E-63 |
| FEZ1 | 2.562163 | 1.135533 | -1.42663 | 1.37E-50 | 4.39E-50 |
| FAM3D | 3.143489 | 4.153007 | 1.009518 | 1.00E-07 | 1.23E-07 |
| ADAM12 | 0.246039 | 1.471487 | 1.225448 | 3.11E-66 | 3.00E-65 |
| RPL21P119 | 0.517289 | 1.665245 | 1.147956 | 4.31E-57 | 1.89E-56 |
| SELENBP1 | 6.053217 | 4.134205 | -1.91901 | 2.06E-61 | 1.24E-60 |
| DMAP1 | 3.699496 | 2.489893 | -1.2096 | 4.43E-74 | 1.32E-72 |
| SLC30A6 | 2.103635 | 3.373133 | 1.269498 | 5.40E-79 | 6.71E-77 |
| MANEA | 1.549026 | 2.767608 | 1.218582 | 2.14E-60 | 1.18E-59 |
| MUS81 | 4.241129 | 2.755409 | -1.48572 | 2.80E-69 | 3.93E-68 |
| UBL5 | 6.683253 | 5.568518 | -1.11474 | 1.52E-62 | 1.00E-61 |
| FAM83B | 0.77208 | 2.540303 | 1.768222 | 2.26E-57 | 1.00E-56 |
| RAB10 | 4.310674 | 5.813599 | 1.502926 | 2.39E-72 | 5.34E-71 |
| INAVA | 2.549568 | 4.33258 | 1.783012 | 4.98E-43 | 1.26E-42 |
| FZD5 | 2.582423 | 3.973396 | 1.390972 | 1.03E-53 | 3.76E-53 |
| CD163 | 3.636145 | 2.413903 | -1.22224 | 8.51E-22 | 1.38E-21 |
| REG4 | 1.320896 | 5.096197 | 3.775301 | 1.41E-41 | 3.45E-41 |
| TUBA8 | 1.308944 | 0.105402 | -1.20354 | 6.77E-63 | 4.64E-62 |
| AC006128.1 | 2.441754 | 1.026459 | -1.4153 | 1.08E-50 | 3.48E-50 |
| ANPEP | 2.389789 | 4.686933 | 2.297144 | 3.82E-30 | 7.26E-30 |
| CLDN4 | 2.242938 | 6.406618 | 4.16368 | 1.66E-73 | 4.46E-72 |
| AL121761.1 | 0.819641 | 2.061703 | 1.242062 | 9.84E-18 | 1.48E-17 |
| DDX19B | 3.26262 | 2.003909 | -1.25871 | 7.82E-69 | 1.04E-67 |
| NCBP3 | 3.298918 | 2.169238 | -1.12968 | 7.82E-58 | 3.56E-57 |
| GTF2H3 | 2.143096 | 3.1488 | 1.005704 | 4.65E-75 | 1.66E-73 |
| D2HGDH | 3.608166 | 2.306606 | -1.30156 | 9.02E-52 | 3.04E-51 |
| SMIM31 | 0.443832 | 1.508917 | 1.065086 | 4.47E-25 | 7.70E-25 |
| NDC1 | 1.669101 | 3.678226 | 2.009124 | 1.04E-83 | 1.43E-80 |
| PABPC1L | 3.419248 | 2.322238 | -1.09701 | 1.11E-22 | 1.82E-22 |
| PABPN1 | 5.66182 | 4.313291 | -1.34853 | 3.34E-50 | 1.05E-49 |
| C11orf58 | 4.903768 | 3.721171 | -1.1826 | 2.59E-60 | 1.42E-59 |
| CES2 | 3.644476 | 4.750348 | 1.105871 | 1.12E-24 | 1.92E-24 |
| CGRRF1 | 2.510085 | 1.406405 | -1.10368 | 2.53E-71 | 4.75E-70 |
| EPB41L4A-AS1 | 4.613999 | 2.1596 | -2.4544 | 9.46E-73 | 2.24E-71 |
| RN7SL138P | 0.46777 | 1.558144 | 1.090374 | 5.88E-61 | 3.38E-60 |
| ENC1 | 2.444859 | 4.544601 | 2.099742 | 1.60E-71 | 3.09E-70 |
| AC138932.1 | 2.553173 | 0.813724 | -1.73945 | 9.89E-64 | 7.39E-63 |
| SLC34A2 | 0.385403 | 1.392493 | 1.007089 | 1.84E-12 | 2.50E-12 |
| MAML2 | 1.385349 | 2.503819 | 1.11847 | 7.36E-59 | 3.61E-58 |
| HSF4 | 4.070522 | 1.229476 | -2.84105 | 5.60E-59 | 2.77E-58 |
| DIP2B | 1.820323 | 3.141908 | 1.321585 | 5.51E-76 | 2.30E-74 |
| CCR1 | 1.008725 | 2.325801 | 1.317076 | 6.46E-52 | 2.19E-51 |
| CCDC107 | 4.643725 | 2.698436 | -1.94529 | 4.95E-75 | 1.75E-73 |
| LNX2 | 2.193225 | 3.379306 | 1.186081 | 3.56E-59 | 1.79E-58 |
| AFAP1 | 2.046464 | 3.049078 | 1.002613 | 1.46E-40 | 3.48E-40 |
| SORBS3 | 4.854901 | 3.811122 | -1.04378 | 7.89E-46 | 2.17E-45 |
| AL138785.1 | 0.463683 | 2.539056 | 2.075373 | 5.58E-59 | 2.76E-58 |
| ELF4 | 2.793884 | 4.353789 | 1.559905 | 8.56E-64 | 6.44E-63 |
| ELP2 | 3.690019 | 2.313833 | -1.37619 | 6.93E-58 | 3.17E-57 |
| KRT6B | 0.503379 | 1.700572 | 1.197193 | 6.58E-13 | 9.03E-13 |
| FADS3 | 3.474434 | 1.936663 | -1.53777 | 7.35E-53 | 2.58E-52 |
| AC025580.1 | 0.312598 | 1.380983 | 1.068385 | 4.54E-41 | 1.10E-40 |
| IGFBP3 | 3.977258 | 5.56924 | 1.591982 | 2.21E-48 | 6.58E-48 |
| GZMA | 1.589852 | 2.975786 | 1.385934 | 3.35E-35 | 7.10E-35 |
| FUCA1 | 3.73327 | 5.230499 | 1.497229 | 4.93E-58 | 2.27E-57 |
| ZNF667-AS1 | 2.57541 | 0.998811 | -1.5766 | 1.63E-60 | 9.06E-60 |
| ADCY5 | 2.861545 | 1.40679 | -1.45475 | 7.58E-32 | 1.50E-31 |
| NCAPG2 | 1.477049 | 2.529526 | 1.052477 | 8.23E-54 | 3.02E-53 |
| PLCXD2 | 1.92305 | 0.652433 | -1.27062 | 2.78E-25 | 4.83E-25 |
| CLDN5 | 3.855691 | 2.325302 | -1.53039 | 3.69E-47 | 1.06E-46 |
| HNF4A | 1.915656 | 4.30909 | 2.393434 | 4.08E-62 | 2.60E-61 |
| APBB1 | 3.511169 | 1.766486 | -1.74468 | 3.45E-61 | 2.02E-60 |
| SCNN1D | 1.737679 | 0.529728 | -1.20795 | 7.44E-67 | 7.70E-66 |
| AC139256.2 | 2.836075 | 0.370646 | -2.46543 | 6.88E-64 | 5.23E-63 |
| RPL7AP50 | 0.73276 | 2.51242 | 1.77966 | 6.06E-65 | 5.16E-64 |
| NECTIN1 | 1.840203 | 3.536066 | 1.695863 | 1.78E-66 | 1.77E-65 |
| PDK2 | 4.185386 | 2.846948 | -1.33844 | 3.43E-67 | 3.68E-66 |
| IGLV1-51 | 4.449349 | 5.985836 | 1.536487 | 1.62E-10 | 2.12E-10 |
| AC244669.1 | 2.59854 | 1.326501 | -1.27204 | 2.74E-56 | 1.14E-55 |
| RB1 | 2.631296 | 4.155961 | 1.524666 | 9.92E-70 | 1.48E-68 |
| EIF2B4 | 4.091914 | 2.811045 | -1.28087 | 2.37E-67 | 2.59E-66 |
| RNU7-40P | 0.313176 | 2.578947 | 2.265771 | 4.90E-70 | 7.67E-69 |
| ZNF410 | 3.142168 | 0.46449 | -2.67768 | 8.70E-73 | 2.08E-71 |
| MAN1A1 | 2.998163 | 4.401429 | 1.403265 | 2.77E-46 | 7.72E-46 |
| TMEM14B | 4.536771 | 3.465778 | -1.07099 | 3.89E-54 | 1.45E-53 |
| TK1 | 2.565015 | 5.299475 | 2.734461 | 5.59E-72 | 1.16E-70 |
| SP100 | 4.124677 | 2.978181 | -1.1465 | 5.10E-56 | 2.09E-55 |
| IGHV4-31 | 1.896338 | 4.071976 | 2.175637 | 1.01E-29 | 1.91E-29 |
| ZBTB16 | 2.878226 | 0.599362 | -2.27886 | 2.87E-69 | 4.04E-68 |
| GUK1 | 6.473821 | 5.177337 | -1.29648 | 1.37E-68 | 1.77E-67 |
| DDX5 | 7.213884 | 5.906236 | -1.30765 | 4.58E-56 | 1.89E-55 |
| HSPB2 | 1.600814 | 0.388619 | -1.2122 | 5.43E-64 | 4.17E-63 |
| GIPR | 2.043032 | 0.553426 | -1.48961 | 6.92E-30 | 1.31E-29 |
| CLK1 | 5.577265 | 3.675762 | -1.9015 | 2.40E-61 | 1.42E-60 |
| CYP2C9 | 2.297932 | 1.014684 | -1.28325 | 1.82E-17 | 2.73E-17 |
| NMB | 1.832355 | 2.986958 | 1.154603 | 1.03E-49 | 3.20E-49 |
| MTCO1P40 | 1.147217 | 4.880255 | 3.733038 | 2.81E-59 | 1.43E-58 |
| SYNPO2 | 4.725989 | 2.906619 | -1.81937 | 1.03E-27 | 1.86E-27 |
| ACSF3 | 2.995183 | 1.75062 | -1.24456 | 9.34E-64 | 6.99E-63 |
| NCAPD2 | 2.771558 | 4.521148 | 1.74959 | 2.38E-80 | 4.58E-78 |
| MIR3189 | 0.044001 | 1.623233 | 1.579232 | 2.22E-60 | 1.22E-59 |
| MUC5AC | 4.803816 | 3.422377 | -1.38144 | 2.72E-07 | 3.32E-07 |
| CCND2 | 2.590991 | 3.934939 | 1.343947 | 2.31E-25 | 4.01E-25 |
| TOM1L1 | 3.738605 | 2.691829 | -1.04678 | 4.20E-36 | 9.08E-36 |
| SLC31A2 | 2.31837 | 0.541606 | -1.77676 | 4.08E-71 | 7.41E-70 |
| FAM84A | 1.733478 | 2.735024 | 1.001546 | 7.41E-15 | 1.05E-14 |
| PGM2L1 | 0.834245 | 2.131823 | 1.297578 | 2.45E-80 | 4.67E-78 |
| PTOV1-AS2 | 3.019601 | 1.781128 | -1.23847 | 1.77E-46 | 4.96E-46 |
| SLC25A43 | 2.053739 | 3.563234 | 1.509494 | 1.29E-67 | 1.45E-66 |
| NACA | 8.006775 | 5.636954 | -2.36982 | 1.77E-63 | 1.29E-62 |
| ACSF2 | 4.033382 | 2.121899 | -1.91148 | 2.65E-53 | 9.50E-53 |
| INPP5J | 2.446528 | 1.428306 | -1.01822 | 3.09E-32 | 6.15E-32 |
| MRPL53 | 4.356179 | 1.718562 | -2.63762 | 1.56E-59 | 8.06E-59 |
| FKBP11 | 4.075374 | 3.007847 | -1.06753 | 1.11E-31 | 2.17E-31 |
| RPL5P34 | 1.126587 | 2.610126 | 1.483539 | 9.40E-55 | 3.62E-54 |
| PMS2P1 | 1.481771 | 2.947521 | 1.46575 | 7.23E-74 | 2.08E-72 |
| RGL3 | 3.572408 | 1.282576 | -2.28983 | 4.35E-43 | 1.11E-42 |
| AC006122.1 | 0.230342 | 1.263693 | 1.033351 | 4.08E-62 | 2.60E-61 |
| SNORD101 | 0.186262 | 1.811197 | 1.624935 | 1.09E-70 | 1.87E-69 |
| MAFB | 2.283435 | 3.667546 | 1.384112 | 1.48E-42 | 3.72E-42 |
| AC012615.3 | 1.663556 | 0.005155 | -1.6584 | 8.62E-76 | 3.46E-74 |
| CBLIF | 6.589674 | 1.115318 | -5.47436 | 6.56E-45 | 1.75E-44 |
| GMPR | 3.702778 | 1.756589 | -1.94619 | 5.97E-60 | 3.18E-59 |
| AC022018.1 | 0.200486 | 1.27216 | 1.071674 | 4.09E-65 | 3.53E-64 |
| COMMD6 | 6.040825 | 4.549984 | -1.49084 | 1.65E-64 | 1.34E-63 |
| AC113191.1 | 0.965044 | 3.185399 | 2.220355 | 8.70E-77 | 4.32E-75 |
| PDE4DIP | 3.779088 | 1.510541 | -2.26855 | 9.51E-68 | 1.09E-66 |
| FAM131A | 2.842664 | 1.702363 | -1.1403 | 1.13E-59 | 5.90E-59 |
| HCG25 | 2.92245 | 0.591799 | -2.33065 | 1.46E-54 | 5.56E-54 |
| AC008894.2 | 2.852461 | 0.080733 | -2.77173 | 2.86E-66 | 2.77E-65 |
| LMO3 | 1.479693 | 0.463129 | -1.01656 | 1.01E-38 | 2.31E-38 |
| BEX2 | 2.805923 | 1.281234 | -1.52469 | 4.06E-38 | 9.15E-38 |
| FGGY | 1.999027 | 0.932961 | -1.06607 | 7.61E-64 | 5.75E-63 |
| SOX4 | 2.377017 | 5.083404 | 2.706387 | 3.43E-82 | 1.37E-79 |
| FMC1-LUC7L2 | 2.376423 | 0.018889 | -2.35753 | 5.04E-67 | 5.28E-66 |
| AC078817.1 | 0.465927 | 2.131548 | 1.665621 | 4.04E-61 | 2.36E-60 |
| MOK | 2.521894 | 1.132901 | -1.38899 | 1.92E-60 | 1.06E-59 |
| OSGEP | 3.73257 | 2.484904 | -1.24767 | 2.01E-59 | 1.03E-58 |
| AL355816.2 | 1.917551 | 0.002008 | -1.91554 | 1.78E-78 | 1.35E-76 |
| VEGFA | 4.956028 | 3.658103 | -1.29793 | 4.32E-31 | 8.36E-31 |
| CBS | 2.950766 | 0.085993 | -2.86477 | 5.50E-73 | 1.35E-71 |
| PATL1 | 3.033362 | 4.787641 | 1.754279 | 2.28E-82 | 1.05E-79 |
| PIGM | 1.680974 | 2.802551 | 1.121577 | 1.33E-73 | 3.69E-72 |
| RNU4-62P | 0.42098 | 2.389949 | 1.96897 | 1.46E-59 | 7.55E-59 |
| CIP2A | 0.706038 | 2.002931 | 1.296893 | 8.18E-79 | 9.33E-77 |
| SNORA65 | 0.655588 | 1.891255 | 1.235667 | 7.32E-39 | 1.68E-38 |
| IGHV1-69D | 2.981465 | 4.778607 | 1.797142 | 6.55E-17 | 9.66E-17 |
| AC002044.1 | 0.159295 | 1.242147 | 1.082852 | 1.77E-67 | 1.96E-66 |
| GTF2IRD2 | 2.928025 | 0.365127 | -2.5629 | 1.43E-73 | 3.92E-72 |
| ARHGEF26 | 2.623589 | 1.314681 | -1.30891 | 4.18E-44 | 1.09E-43 |
| POLR1A | 1.810514 | 2.986657 | 1.176143 | 5.49E-78 | 3.68E-76 |
| OSR1 | 1.951773 | 0.63497 | -1.3168 | 5.72E-59 | 2.83E-58 |
| AC009065.5 | 1.261977 | 3.016806 | 1.75483 | 2.02E-54 | 7.64E-54 |
| ELK3 | 2.519498 | 4.082099 | 1.562601 | 1.10E-61 | 6.71E-61 |
| QSER1 | 1.685112 | 2.691427 | 1.006316 | 1.15E-66 | 1.17E-65 |
| AC091564.4 | 2.100075 | 0.002008 | -2.09807 | 1.78E-78 | 1.35E-76 |
| EME2 | 2.426566 | 1.297641 | -1.12893 | 8.84E-54 | 3.24E-53 |
| KLHDC4 | 3.25417 | 1.711739 | -1.54243 | 1.24E-48 | 3.72E-48 |
| TBC1D10A | 3.087472 | 1.553448 | -1.53402 | 5.08E-74 | 1.50E-72 |
| PTGDR2 | 1.928539 | 0.664577 | -1.26396 | 1.61E-37 | 3.57E-37 |
| ADAMTS10 | 2.413177 | 1.123439 | -1.28974 | 3.53E-50 | 1.11E-49 |
| AC106872.6 | 0.227115 | 1.451198 | 1.224083 | 2.99E-64 | 2.37E-63 |
| PRCD | 1.378642 | 0.159355 | -1.21929 | 1.67E-76 | 7.80E-75 |
| IGHV2-26 | 1.64746 | 3.13424 | 1.48678 | 5.81E-18 | 8.73E-18 |
| EEF1A2 | 3.003919 | 1.572726 | -1.43119 | 1.37E-32 | 2.76E-32 |
| GABRE | 2.073451 | 0.839297 | -1.23415 | 3.96E-39 | 9.15E-39 |
| SCARA5 | 3.347729 | 0.911171 | -2.43656 | 1.88E-70 | 3.11E-69 |
| TIAL1 | 4.7207 | 3.540909 | -1.17979 | 4.38E-57 | 1.92E-56 |
| SPTLC2 | 2.594418 | 3.736725 | 1.142307 | 1.15E-60 | 6.50E-60 |
| PLEK | 1.65346 | 2.938211 | 1.28475 | 3.29E-36 | 7.13E-36 |
| RPS6KA4 | 3.31412 | 4.393618 | 1.079498 | 2.84E-58 | 1.33E-57 |
| RARRES3 | 4.330878 | 5.372245 | 1.041367 | 1.20E-17 | 1.79E-17 |
| RCAN2 | 3.64822 | 2.609396 | -1.03882 | 4.28E-27 | 7.65E-27 |
| NDUFB8 | 6.073948 | 3.930174 | -2.14377 | 3.08E-70 | 4.97E-69 |
| RPS20P14 | 1.571596 | 3.076662 | 1.505066 | 2.00E-59 | 1.03E-58 |
| SLC26A6 | 4.113926 | 2.440529 | -1.6734 | 5.15E-38 | 1.16E-37 |
| MTCP1 | 2.736177 | 0.419971 | -2.31621 | 6.57E-56 | 2.68E-55 |
| RNF31 | 3.884127 | 2.650638 | -1.23349 | 3.68E-61 | 2.15E-60 |
| GLRX | 3.960616 | 2.512204 | -1.44841 | 9.52E-57 | 4.08E-56 |
| RPS15AP12 | 0.523019 | 1.912291 | 1.389272 | 2.55E-65 | 2.25E-64 |
| RAB26 | 2.273292 | 0.868174 | -1.40512 | 1.32E-24 | 2.26E-24 |
| HAVCR2 | 0.983119 | 2.074574 | 1.091455 | 6.82E-50 | 2.13E-49 |
| IGHM | 5.163682 | 6.447988 | 1.284306 | 3.84E-07 | 4.66E-07 |
| ASPM | 0.548746 | 2.255939 | 1.707193 | 4.47E-74 | 1.33E-72 |
| IRAK2 | 1.164384 | 2.725285 | 1.560901 | 1.02E-61 | 6.30E-61 |
| CASK | 2.551966 | 3.56354 | 1.011575 | 3.36E-62 | 2.15E-61 |
| BX679664.1 | 0.335502 | 1.770736 | 1.435234 | 6.05E-63 | 4.15E-62 |
| C11orf98 | 4.372306 | 2.625152 | -1.74715 | 1.52E-59 | 7.86E-59 |
| SERPINB5 | 0.680394 | 4.091713 | 3.411319 | 2.79E-64 | 2.22E-63 |
| ETV7 | 1.286091 | 2.887407 | 1.601317 | 2.98E-54 | 1.12E-53 |
| TSPAN7 | 3.230909 | 2.059188 | -1.17172 | 1.15E-34 | 2.41E-34 |
| COPS7B | 3.789609 | 2.64827 | -1.14134 | 2.85E-55 | 1.12E-54 |
| INTS11 | 5.330089 | 3.428741 | -1.90135 | 3.18E-64 | 2.51E-63 |
| AC073861.1 | 3.497573 | 5.647595 | 2.150022 | 9.21E-65 | 7.70E-64 |
| HLA-DQB1 | 2.965125 | 4.66998 | 1.704855 | 5.59E-31 | 1.08E-30 |
| PET100 | 4.206089 | 3.134583 | -1.07151 | 4.74E-48 | 1.39E-47 |
| INSYN1 | 1.630822 | 0.38305 | -1.24777 | 3.82E-64 | 2.99E-63 |
| CPVL | 2.289761 | 3.357625 | 1.067864 | 2.70E-25 | 4.69E-25 |
| PAQR4 | 2.283263 | 3.380832 | 1.097569 | 1.13E-38 | 2.58E-38 |
| KIAA0895L | 3.486893 | 1.766444 | -1.72045 | 1.46E-57 | 6.53E-57 |
| NUTM2D | 1.288594 | 0.183057 | -1.10554 | 2.00E-73 | 5.30E-72 |
| IGLV1-40 | 4.426387 | 6.385118 | 1.958731 | 4.62E-16 | 6.70E-16 |
| CAMK2N1 | 3.191828 | 5.603165 | 2.411337 | 5.01E-56 | 2.06E-55 |
| COPS5 | 4.020798 | 2.874389 | -1.14641 | 7.23E-55 | 2.80E-54 |
| CHKA | 4.443658 | 3.221607 | -1.22205 | 1.02E-41 | 2.50E-41 |
| AC073072.2 | 0.219346 | 1.258818 | 1.039472 | 6.02E-63 | 4.14E-62 |
| BAK1 | 3.138953 | 4.461031 | 1.322078 | 1.45E-56 | 6.14E-56 |
| AC138305.3 | 1.515933 | 3.06603 | 1.550097 | 3.95E-45 | 1.06E-44 |
| IGHV2-70 | 2.05817 | 3.189303 | 1.131133 | 5.68E-11 | 7.50E-11 |
| ZNF577 | 2.442074 | 0.811463 | -1.63061 | 1.19E-64 | 9.83E-64 |
| FAM228B | 2.363968 | 0.819311 | -1.54466 | 1.75E-71 | 3.34E-70 |
| UBE2L6 | 4.188982 | 5.325265 | 1.136283 | 4.16E-43 | 1.06E-42 |
| RPL5P4 | 0.739601 | 1.953516 | 1.213915 | 7.50E-54 | 2.76E-53 |
| ATP6V0C | 5.871277 | 4.409296 | -1.46198 | 1.18E-65 | 1.08E-64 |
| CDC20 | 1.742015 | 4.705636 | 2.963621 | 9.53E-72 | 1.91E-70 |
| PLEKHG1 | 1.250785 | 2.583429 | 1.332644 | 2.60E-68 | 3.22E-67 |
| HEXA | 4.405145 | 2.52241 | -1.88273 | 4.50E-55 | 1.76E-54 |
| HSPA13 | 2.031729 | 3.16725 | 1.135521 | 2.07E-56 | 8.71E-56 |
| SYBU | 3.380798 | 2.023407 | -1.35739 | 9.77E-44 | 2.53E-43 |
| HERC1 | 3.822478 | 2.292937 | -1.52954 | 3.54E-66 | 3.39E-65 |
| MGAT2 | 2.567637 | 1.538984 | -1.02865 | 5.15E-57 | 2.24E-56 |
| SMG1P1 | 1.781727 | 0.150176 | -1.63155 | 1.92E-59 | 9.91E-59 |
| DUSP22 | 3.865273 | 2.621669 | -1.2436 | 1.29E-72 | 3.00E-71 |
| TPTEP1 | 2.621049 | 0.404848 | -2.2162 | 1.39E-77 | 8.29E-76 |
| LRRC40 | 1.811804 | 2.845937 | 1.034133 | 1.08E-66 | 1.10E-65 |
| GSTM2 | 5.677047 | 0.793052 | -4.88399 | 9.13E-82 | 3.03E-79 |
| MIR6739 | 0.098261 | 1.256974 | 1.158713 | 2.49E-61 | 1.48E-60 |
| HAGH | 4.427085 | 2.373344 | -2.05374 | 5.17E-79 | 6.48E-77 |
| AZGP1 | 5.346106 | 3.225905 | -2.1202 | 1.43E-21 | 2.31E-21 |
| AC092868.1 | 0.438064 | 1.563599 | 1.125535 | 1.03E-40 | 2.47E-40 |
| GMDS-DT | 1.74926 | 0.426414 | -1.32285 | 3.42E-69 | 4.74E-68 |
| PSCA | 7.804102 | 4.17388 | -3.63022 | 4.95E-29 | 9.20E-29 |
| TCEA2 | 3.781035 | 2.101461 | -1.67957 | 7.04E-65 | 5.96E-64 |
| CTRB1 | 1.401479 | 0.129502 | -1.27198 | 2.86E-52 | 9.80E-52 |
| VXN | 1.703907 | 0.114676 | -1.58923 | 3.64E-77 | 1.97E-75 |
| SLC12A7 | 3.805552 | 4.908368 | 1.102816 | 2.02E-50 | 6.43E-50 |
| SERF1B | 3.143055 | 0.09601 | -3.04705 | 3.63E-74 | 1.10E-72 |
| SAMD9L | 1.770847 | 2.957922 | 1.187075 | 1.06E-37 | 2.36E-37 |
| MCM6 | 2.252775 | 3.927094 | 1.674319 | 2.49E-80 | 4.68E-78 |
| AC016734.1 | 0.676029 | 1.76732 | 1.091292 | 5.03E-66 | 4.75E-65 |
| NFATC2 | 1.69181 | 2.825463 | 1.133653 | 4.01E-51 | 1.31E-50 |
| TTI1 | 2.371887 | 3.426873 | 1.054986 | 2.08E-79 | 2.99E-77 |
| MAPK8IP3 | 4.293588 | 2.579637 | -1.71395 | 1.78E-55 | 7.09E-55 |
| FP565260.6 | 1.839458 | 0.458856 | -1.3806 | 5.61E-61 | 3.23E-60 |
| DTNA | 2.333856 | 0.604992 | -1.72886 | 6.57E-67 | 6.83E-66 |
| MALSU1 | 3.70631 | 2.606909 | -1.0994 | 2.90E-57 | 1.28E-56 |
| MBNL2 | 3.296564 | 4.614717 | 1.318153 | 1.38E-52 | 4.79E-52 |
| AP004608.1 | 1.527516 | 0.273312 | -1.2542 | 7.19E-36 | 1.54E-35 |
| PPP1R14BP3 | 1.290362 | 3.237849 | 1.947488 | 3.83E-71 | 7.01E-70 |
| AC003665.1 | 3.737923 | 2.591694 | -1.14623 | 3.49E-22 | 5.70E-22 |
| UBQLN2 | 2.806217 | 4.313136 | 1.506919 | 3.69E-62 | 2.36E-61 |
| DNAJC4 | 4.836376 | 3.611356 | -1.22502 | 1.57E-69 | 2.29E-68 |
| HSP90AB3P | 0.482489 | 1.818095 | 1.335606 | 9.32E-79 | 1.02E-76 |
| LRRC37A2 | 2.398811 | 0.802491 | -1.59632 | 8.68E-58 | 3.94E-57 |
| GPSM1 | 2.742554 | 1.599311 | -1.14324 | 1.06E-48 | 3.18E-48 |
| KIF4A | 0.644638 | 2.671402 | 2.026764 | 4.77E-77 | 2.54E-75 |
| SLC9A3R1 | 4.919424 | 6.14434 | 1.224916 | 9.02E-31 | 1.74E-30 |
| ANLN | 1.098235 | 3.590892 | 2.492657 | 1.64E-80 | 3.42E-78 |
| NOLC1 | 3.743232 | 5.225692 | 1.48246 | 1.25E-76 | 5.93E-75 |
| HIST1H2BH | 0.579466 | 1.954712 | 1.375245 | 1.07E-42 | 2.71E-42 |
| SYTL1 | 4.022797 | 2.37697 | -1.64583 | 1.56E-22 | 2.57E-22 |
| AC135983.2 | 1.857806 | 0.54435 | -1.31346 | 1.03E-62 | 6.89E-62 |
| TACC3 | 2.182696 | 3.362733 | 1.180037 | 1.05E-48 | 3.14E-48 |
| INTS3 | 4.093879 | 2.897211 | -1.19667 | 9.14E-55 | 3.52E-54 |
| ERMP1 | 2.324149 | 3.35305 | 1.028901 | 1.56E-46 | 4.38E-46 |
| CARMIL1 | 2.210415 | 3.358236 | 1.147821 | 1.61E-59 | 8.29E-59 |
| TWF1 | 3.734051 | 5.018111 | 1.28406 | 2.42E-71 | 4.55E-70 |
| MICALL2 | 4.502181 | 2.870938 | -1.63124 | 3.24E-48 | 9.61E-48 |
| C8orf88 | 2.212833 | 0.982784 | -1.23005 | 3.12E-37 | 6.91E-37 |
| CCL14 | 3.838801 | 0.399637 | -3.43916 | 9.23E-79 | 1.01E-76 |
| IL4I1 | 0.595778 | 2.270891 | 1.675113 | 1.92E-70 | 3.18E-69 |
| KIFC3 | 3.323747 | 2.011733 | -1.31201 | 5.63E-52 | 1.91E-51 |
| STUB1 | 5.184704 | 3.928559 | -1.25614 | 2.29E-76 | 1.04E-74 |
| RPS23 | 8.873707 | 6.42201 | -2.4517 | 9.27E-63 | 6.25E-62 |
| PLAUR | 2.998902 | 4.14846 | 1.149558 | 2.26E-29 | 4.23E-29 |
| SLFN5 | 1.764655 | 2.947394 | 1.182739 | 2.98E-51 | 9.81E-51 |
| PLCB3 | 3.017606 | 4.482185 | 1.464578 | 4.96E-60 | 2.66E-59 |
| MYL6B | 3.457806 | 2.365943 | -1.09186 | 3.12E-55 | 1.23E-54 |
| AL034379.1 | 0.614169 | 2.423906 | 1.809737 | 4.44E-63 | 3.10E-62 |
| HSPA1A | 5.925329 | 4.386351 | -1.53898 | 1.02E-26 | 1.81E-26 |
| MVB12A | 4.157872 | 3.120671 | -1.0372 | 1.58E-65 | 1.42E-64 |
| LYNX1 | 2.768668 | 1.454674 | -1.31399 | 4.52E-34 | 9.35E-34 |
| NDUFA13 | 6.033195 | 3.569089 | -2.46411 | 5.98E-68 | 7.06E-67 |
| NCOA4 | 4.743603 | 6.027984 | 1.284381 | 5.41E-53 | 1.91E-52 |
| INO80B-WBP1 | 2.322046 | 0.02538 | -2.29667 | 2.90E-65 | 2.54E-64 |
| GPR89A | 2.617884 | 0.987406 | -1.63048 | 1.89E-50 | 6.03E-50 |
| ADORA2A | 1.263092 | 0.139894 | -1.1232 | 1.40E-67 | 1.57E-66 |
| AKAP8L | 4.70669 | 2.746779 | -1.95991 | 2.48E-63 | 1.78E-62 |
| RPS19P3 | 0.76922 | 2.221853 | 1.452632 | 1.93E-66 | 1.91E-65 |
| KRT23 | 0.33518 | 1.842778 | 1.507599 | 1.54E-23 | 2.58E-23 |
| KPNA2P3 | 1.845006 | 0.002008 | -1.843 | 1.78E-78 | 1.35E-76 |
| LRCH4 | 4.082558 | 2.034641 | -2.04792 | 1.38E-62 | 9.12E-62 |
| PRR11 | 0.787462 | 3.171701 | 2.384238 | 2.78E-82 | 1.22E-79 |
| IFIT2 | 1.113501 | 2.311079 | 1.197578 | 2.20E-48 | 6.53E-48 |
| AC005392.2 | 0.427608 | 1.985376 | 1.557768 | 2.82E-44 | 7.41E-44 |
| SLC44A1 | 2.625995 | 4.067714 | 1.441719 | 3.04E-74 | 9.25E-73 |
| RRS1 | 3.115568 | 4.334686 | 1.219118 | 2.90E-62 | 1.86E-61 |
| RPS23P8 | 3.590517 | 4.855275 | 1.264758 | 9.74E-46 | 2.67E-45 |
| DDAH1 | 3.12031 | 4.785395 | 1.665086 | 4.00E-72 | 8.51E-71 |
| LDHD | 3.293586 | 2.187731 | -1.10585 | 2.59E-26 | 4.57E-26 |
| MIR22HG | 3.993886 | 2.527911 | -1.46598 | 3.18E-49 | 9.70E-49 |
| C2CD4D | 0.436531 | 1.457226 | 1.020695 | 1.50E-64 | 1.23E-63 |
| AC009630.3 | 1.411858 | 0.002008 | -1.40985 | 1.78E-78 | 1.35E-76 |
| IFNGR1 | 4.45824 | 5.472677 | 1.014436 | 3.23E-54 | 1.21E-53 |
| CTSB | 6.289034 | 7.398514 | 1.109481 | 2.67E-46 | 7.44E-46 |
| EIF2B5 | 4.350598 | 3.29965 | -1.05095 | 5.31E-51 | 1.73E-50 |
| DGKA | 3.561765 | 2.112 | -1.44977 | 5.99E-59 | 2.96E-58 |
| AC092597.1 | 0.412943 | 1.540685 | 1.127742 | 2.86E-62 | 1.84E-61 |
| CMTM6 | 3.251748 | 4.712797 | 1.461048 | 5.93E-80 | 9.83E-78 |
| NPIPA5 | 2.802487 | 0.813517 | -1.98897 | 2.05E-48 | 6.10E-48 |
| LGI4 | 3.153924 | 0.724271 | -2.42965 | 2.54E-76 | 1.13E-74 |
| CDX2 | 0.322449 | 2.688311 | 2.365862 | 3.19E-59 | 1.62E-58 |
| PDP1 | 1.994523 | 3.178411 | 1.183888 | 2.68E-73 | 6.98E-72 |
| SDC1 | 3.561026 | 6.141135 | 2.580109 | 9.81E-53 | 3.43E-52 |
| PFKM | 3.76729 | 2.691232 | -1.07606 | 1.87E-50 | 5.96E-50 |
| SLC30A1 | 2.043862 | 3.500216 | 1.456354 | 3.37E-65 | 2.93E-64 |
| KIAA0391 | 2.208131 | 0.748351 | -1.45978 | 4.87E-60 | 2.62E-59 |
| CCDC32 | 2.857339 | 1.423865 | -1.43347 | 1.31E-71 | 2.56E-70 |
| SST | 5.45951 | 1.563708 | -3.8958 | 4.77E-48 | 1.40E-47 |
| TCEAL4 | 5.492369 | 4.054678 | -1.43769 | 8.20E-57 | 3.53E-56 |
| TMOD3 | 2.513743 | 3.601461 | 1.087718 | 1.10E-67 | 1.25E-66 |
| AC005062.1 | 1.816077 | 0.305528 | -1.51055 | 2.76E-71 | 5.14E-70 |
| LINC02086 | 0.133834 | 1.209373 | 1.075539 | 1.40E-56 | 5.92E-56 |
| TM9SF2 | 4.678553 | 6.327831 | 1.649279 | 1.77E-77 | 1.03E-75 |
| ABRACL | 3.437951 | 4.840933 | 1.402982 | 3.61E-48 | 1.07E-47 |
| PKD1P1 | 2.011121 | 0.023801 | -1.98732 | 4.41E-69 | 6.01E-68 |
| IGLV3-10 | 3.149205 | 4.179285 | 1.03008 | 5.27E-07 | 6.39E-07 |
| PCSK9 | 0.680922 | 2.29733 | 1.616407 | 2.21E-40 | 5.26E-40 |
| C11orf52 | 2.443127 | 0.067893 | -2.37523 | 2.08E-67 | 2.29E-66 |
| HIST2H2AA3 | 3.640337 | 0.394561 | -3.24578 | 3.56E-59 | 1.79E-58 |
| SNRPB | 5.515602 | 7.786363 | 2.27076 | 3.64E-85 | 3.26E-81 |
| BATF | 1.708796 | 3.192834 | 1.484037 | 1.08E-53 | 3.93E-53 |
| MBNL3 | 1.35296 | 2.514097 | 1.161137 | 5.88E-54 | 2.17E-53 |
| HOXB-AS4 | 0.056199 | 1.195865 | 1.139666 | 3.79E-64 | 2.97E-63 |
| CUL3 | 3.932646 | 2.860805 | -1.07184 | 2.60E-59 | 1.32E-58 |
| SPSB3 | 4.272587 | 0.574483 | -3.6981 | 3.20E-70 | 5.16E-69 |
| CT83 | 0.081875 | 1.968021 | 1.886146 | 2.06E-26 | 3.64E-26 |
| PLEK2 | 2.067767 | 4.470037 | 2.40227 | 7.10E-56 | 2.89E-55 |
| IFI6 | 4.554972 | 8.109371 | 3.554399 | 8.94E-73 | 2.13E-71 |
| SRM | 4.749941 | 6.082448 | 1.332508 | 2.16E-72 | 4.85E-71 |
| CIART | 2.493148 | 1.130077 | -1.36307 | 2.40E-51 | 7.95E-51 |
| BRI3 | 6.258141 | 4.386775 | -1.87137 | 5.78E-56 | 2.37E-55 |
| ASMTL-AS1 | 2.513145 | 1.269571 | -1.24357 | 5.83E-44 | 1.52E-43 |
| ESRRB | 1.620292 | 0.098683 | -1.52161 | 1.97E-71 | 3.74E-70 |
| FBXL18 | 1.080776 | 2.137485 | 1.056709 | 8.18E-65 | 6.87E-64 |
| HKDC1 | 0.56671 | 3.023646 | 2.456936 | 3.15E-63 | 2.24E-62 |
| SNHG25 | 1.48302 | 3.870007 | 2.386987 | 1.24E-54 | 4.73E-54 |
| LRTOMT | 2.156418 | 1.141587 | -1.01483 | 1.76E-63 | 1.28E-62 |
| RNU6-1024P | 0.152016 | 1.216971 | 1.064954 | 2.11E-56 | 8.89E-56 |
| CU633967.1 | 1.915317 | 0.022722 | -1.8926 | 3.57E-68 | 4.35E-67 |
| AL627309.6 | 2.683735 | 1.251762 | -1.43197 | 3.29E-40 | 7.79E-40 |
| RPL35P1 | 0.781799 | 2.630136 | 1.848337 | 2.69E-66 | 2.62E-65 |
| AC012321.1 | 2.850153 | 0.002008 | -2.84815 | 1.78E-78 | 1.35E-76 |
| PTMAP4 | 0.596584 | 3.998992 | 3.402408 | 1.42E-71 | 2.76E-70 |
| ATP6V0D1 | 4.800974 | 3.673781 | -1.12719 | 3.54E-71 | 6.51E-70 |
| AC125611.3 | 3.663355 | 0.021147 | -3.64221 | 2.38E-68 | 2.96E-67 |
| ARPC4-TTLL3 | 2.073843 | 0.157074 | -1.91677 | 2.02E-62 | 1.32E-61 |
| TIMM44 | 4.001948 | 2.865981 | -1.13597 | 3.43E-60 | 1.86E-59 |
| SBNO1 | 1.83118 | 2.879606 | 1.048425 | 4.31E-75 | 1.55E-73 |
| WNT2 | 0.134904 | 1.476711 | 1.341807 | 8.66E-65 | 7.26E-64 |
| FANCF | 1.484976 | 2.64706 | 1.162085 | 1.62E-74 | 5.22E-73 |
| DIABLO | 3.590492 | 1.202651 | -2.38784 | 2.92E-63 | 2.08E-62 |
| TRO | 1.894046 | 0.552394 | -1.34165 | 1.97E-62 | 1.28E-61 |
| CSPG4P10 | 1.967072 | 0.703194 | -1.26388 | 6.51E-52 | 2.20E-51 |
| SSR4 | 6.818917 | 5.3807 | -1.43822 | 1.50E-55 | 6.01E-55 |
| MAGED4B | 2.355282 | 0.025281 | -2.33 | 7.61E-70 | 1.16E-68 |
| GNG12 | 3.94458 | 5.182911 | 1.23833 | 3.01E-44 | 7.91E-44 |
| PCDHGC3 | 3.532084 | 2.025536 | -1.50655 | 3.29E-48 | 9.75E-48 |
| TMEM220-AS1 | 1.692413 | 0.490397 | -1.20202 | 1.05E-70 | 1.80E-69 |
| AC097534.2 | 1.825434 | 0.550879 | -1.27455 | 3.46E-69 | 4.80E-68 |
| SGPL1 | 2.473421 | 3.830766 | 1.357345 | 9.04E-77 | 4.46E-75 |
| AC018557.1 | 1.97342 | 0.002008 | -1.97141 | 1.78E-78 | 1.35E-76 |
| CKAP4 | 4.296409 | 5.398518 | 1.102109 | 2.56E-53 | 9.17E-53 |
| BHLHA15 | 2.84161 | 1.785659 | -1.05595 | 2.70E-11 | 3.59E-11 |
| NPIPB5 | 5.014184 | 0.78719 | -4.22699 | 8.10E-55 | 3.12E-54 |
| SNHG21 | 1.77999 | 0.744031 | -1.03596 | 2.41E-60 | 1.33E-59 |
| SLC5A1 | 0.930098 | 2.8767 | 1.946602 | 2.02E-46 | 5.66E-46 |
| IGF2 | 2.251278 | 3.60498 | 1.353702 | 5.93E-18 | 8.91E-18 |
| THTPA | 2.608956 | 1.22745 | -1.38151 | 7.39E-77 | 3.74E-75 |
| CSRP1 | 6.939681 | 4.86109 | -2.07859 | 5.80E-52 | 1.97E-51 |
| HMGA1P3 | 0.167342 | 1.301747 | 1.134405 | 9.72E-71 | 1.68E-69 |
| NAA15 | 2.00729 | 3.051937 | 1.044647 | 2.15E-75 | 8.07E-74 |
| RNU6-1099P | 0.101524 | 1.130643 | 1.029119 | 4.81E-60 | 2.58E-59 |
| RGS12 | 2.706649 | 1.487307 | -1.21934 | 5.47E-58 | 2.52E-57 |
| MXD3 | 2.352966 | 1.28118 | -1.07179 | 2.54E-49 | 7.78E-49 |
| BAMBI | 1.265239 | 2.734225 | 1.468986 | 4.50E-34 | 9.32E-34 |
| TBRG1 | 3.584888 | 2.366686 | -1.2182 | 2.41E-64 | 1.93E-63 |
| FBXO9 | 4.623401 | 2.73917 | -1.88423 | 1.27E-74 | 4.14E-73 |
| ICK | 2.211554 | 3.248033 | 1.036479 | 7.75E-57 | 3.34E-56 |
| SYTL2 | 3.845189 | 2.61739 | -1.2278 | 9.93E-31 | 1.91E-30 |
| AC087393.2 | 1.958709 | 0.002008 | -1.9567 | 1.78E-78 | 1.35E-76 |
| SNORD19C | 0.105633 | 1.538802 | 1.433169 | 1.44E-65 | 1.30E-64 |
| CLK4 | 3.278337 | 1.641748 | -1.63659 | 2.14E-62 | 1.39E-61 |
| TFF3 | 2.532184 | 5.473213 | 2.941029 | 6.84E-39 | 1.57E-38 |
| AC009022.1 | 3.3579 | 1.297779 | -2.06012 | 2.85E-51 | 9.41E-51 |
| ACTBP11 | 0.243421 | 1.339147 | 1.095726 | 6.74E-61 | 3.86E-60 |
| NAP1L1 | 5.833192 | 4.018181 | -1.81501 | 1.14E-57 | 5.12E-57 |
| IGLV3-9 | 1.696633 | 2.941601 | 1.244968 | 1.09E-14 | 1.54E-14 |
| UBBP1 | 0.25049 | 1.527115 | 1.276625 | 8.19E-64 | 6.17E-63 |
| FSCN1 | 2.491187 | 4.575057 | 2.08387 | 1.32E-67 | 1.48E-66 |
| CTTN | 5.838663 | 4.804648 | -1.03402 | 4.28E-45 | 1.15E-44 |
| TEN1 | 2.898744 | 0.134867 | -2.76388 | 1.17E-66 | 1.19E-65 |
| ZNF385B | 2.319223 | 0.409376 | -1.90985 | 2.46E-68 | 3.06E-67 |
| BBIP1 | 2.83891 | 1.287597 | -1.55131 | 2.15E-65 | 1.91E-64 |
| SMAD4 | 3.421377 | 2.352469 | -1.06891 | 2.21E-66 | 2.18E-65 |
| NCOA7 | 2.903206 | 4.290488 | 1.387282 | 4.27E-60 | 2.30E-59 |
| SLC7A8 | 4.251597 | 3.011501 | -1.2401 | 2.67E-42 | 6.65E-42 |
| CRACR2B | 4.774852 | 3.348752 | -1.4261 | 3.32E-18 | 5.03E-18 |
| AC002059.2 | 0.154939 | 1.693745 | 1.538805 | 2.20E-71 | 4.15E-70 |
| LPAR5 | 1.79368 | 2.857826 | 1.064146 | 5.55E-33 | 1.12E-32 |
| HNRNPH1 | 6.85614 | 4.466311 | -2.38983 | 3.57E-52 | 1.22E-51 |
| AC007683.1 | 0.448306 | 1.807187 | 1.358881 | 1.51E-71 | 2.92E-70 |
| CBR3-AS1 | 1.731771 | 0.566539 | -1.16523 | 1.49E-58 | 7.16E-58 |
| AC016888.1 | 1.538003 | 2.601681 | 1.063678 | 1.36E-33 | 2.79E-33 |
| TMEM178A | 1.939764 | 0.475774 | -1.46399 | 2.42E-68 | 3.01E-67 |
| RN7SL674P | 0.313438 | 1.540697 | 1.227259 | 1.70E-51 | 5.66E-51 |
| MNDA | 1.256091 | 2.357136 | 1.101046 | 5.58E-36 | 1.20E-35 |
| SPTY2D1 | 1.882135 | 3.025666 | 1.14353 | 3.48E-69 | 4.81E-68 |
| ZDHHC5 | 3.613368 | 4.96154 | 1.348173 | 5.17E-74 | 1.53E-72 |
| CRYBG1 | 1.533778 | 3.361723 | 1.827945 | 2.68E-56 | 1.12E-55 |
| CD68 | 3.415635 | 1.52845 | -1.88718 | 9.36E-53 | 3.27E-52 |
| ANAPC15 | 3.944305 | 1.86996 | -2.07434 | 2.51E-73 | 6.55E-72 |
| RNA5SP82 | 0.136935 | 1.41556 | 1.278626 | 1.29E-59 | 6.70E-59 |
| MKI67 | 1.236296 | 4.286205 | 3.049909 | 3.79E-80 | 6.66E-78 |
| SLC25A32 | 2.361544 | 3.525868 | 1.164324 | 2.50E-77 | 1.40E-75 |
| CTSD | 7.043304 | 8.293551 | 1.250247 | 9.05E-55 | 3.48E-54 |
| NKG7 | 1.832809 | 3.529052 | 1.696243 | 1.85E-42 | 4.63E-42 |
| CCND1 | 3.399169 | 5.714385 | 2.315216 | 3.21E-69 | 4.47E-68 |
| SH3RF1 | 2.408089 | 3.602621 | 1.194533 | 1.10E-54 | 4.20E-54 |
| ACP6 | 2.694186 | 1.625516 | -1.06867 | 1.03E-46 | 2.91E-46 |
| PLXNB1 | 4.855609 | 3.698041 | -1.15757 | 4.32E-47 | 1.23E-46 |
| RPL12P4 | 2.057106 | 3.194817 | 1.137711 | 6.94E-42 | 1.71E-41 |
| ZNF217 | 2.361324 | 3.832634 | 1.47131 | 1.16E-73 | 3.24E-72 |
| CRYZL1 | 3.320981 | 1.831734 | -1.48925 | 1.60E-69 | 2.32E-68 |
| BBS1 | 3.068293 | 0.499603 | -2.56869 | 9.47E-69 | 1.25E-67 |
| GSAP | 2.739982 | 1.697744 | -1.04224 | 5.07E-49 | 1.54E-48 |
| AL365205.1 | 3.003571 | 1.374138 | -1.62943 | 1.85E-58 | 8.79E-58 |
| BMPER | 1.500222 | 0.301178 | -1.19904 | 5.00E-66 | 4.73E-65 |
| NOL12 | 2.850083 | 1.2461 | -1.60398 | 8.70E-59 | 4.25E-58 |
| ANAPC5 | 4.913785 | 3.281375 | -1.63241 | 1.35E-49 | 4.18E-49 |
| SLC27A2 | 0.693914 | 2.495637 | 1.801723 | 4.14E-61 | 2.42E-60 |
| CHKB-CPT1B | 1.611238 | 0.237226 | -1.37401 | 9.12E-58 | 4.13E-57 |
| POLR1B | 1.714306 | 2.850864 | 1.136559 | 2.50E-81 | 6.99E-79 |
| PPP1R3C | 2.895125 | 1.497133 | -1.39799 | 4.55E-41 | 1.10E-40 |
| AGR2 | 5.93864 | 8.352791 | 2.414151 | 4.37E-24 | 7.39E-24 |
| IGHV3-53 | 1.686431 | 3.311998 | 1.625567 | 8.21E-25 | 1.41E-24 |
| BROX | 2.817591 | 4.095894 | 1.278303 | 3.12E-77 | 1.71E-75 |
| CEACAM7 | 0.446252 | 2.344666 | 1.898414 | 2.65E-39 | 6.15E-39 |
| GSS | 3.695283 | 4.940189 | 1.244906 | 1.16E-65 | 1.06E-64 |
| SMIM27 | 2.357896 | 1.215304 | -1.14259 | 7.68E-71 | 1.34E-69 |
| COMMD3 | 3.960885 | 2.619929 | -1.34096 | 1.91E-56 | 8.07E-56 |
| AP000346.2 | 1.473546 | 0.383253 | -1.09029 | 3.76E-48 | 1.11E-47 |
| HLA-DPA1 | 4.17716 | 5.403477 | 1.226317 | 4.68E-23 | 7.75E-23 |
| CFAP44 | 2.146016 | 0.84761 | -1.29841 | 3.71E-49 | 1.13E-48 |
| CDC25C | 0.525903 | 1.778229 | 1.252326 | 2.94E-64 | 2.33E-63 |
| NPY | 1.591726 | 0.449559 | -1.14217 | 5.59E-47 | 1.59E-46 |
| AC133134.1 | 0.54468 | 2.740992 | 2.196312 | 7.26E-63 | 4.95E-62 |
| PADI2 | 0.836449 | 1.845868 | 1.009419 | 1.66E-31 | 3.26E-31 |
| MAD2L1 | 1.239798 | 2.684761 | 1.444963 | 4.46E-60 | 2.40E-59 |
| CALR | 6.765541 | 8.558837 | 1.793296 | 9.69E-80 | 1.48E-77 |
| PTPRM | 3.631719 | 2.242172 | -1.38955 | 5.65E-58 | 2.60E-57 |
| ZNF10 | 2.18907 | 1.021011 | -1.16806 | 4.77E-61 | 2.77E-60 |
| GAPDH | 8.590303 | 10.04404 | 1.453734 | 1.04E-69 | 1.54E-68 |
| OAZ2 | 4.428214 | 2.952395 | -1.47582 | 1.78E-71 | 3.40E-70 |
| RRBP1 | 5.344249 | 6.513614 | 1.169365 | 4.61E-42 | 1.14E-41 |
| ITFG2 | 3.546516 | 2.229068 | -1.31745 | 7.84E-64 | 5.92E-63 |
| MT2A | 7.439095 | 5.937706 | -1.50139 | 2.98E-43 | 7.62E-43 |
| AL355032.1 | 0.616887 | 1.954549 | 1.337662 | 1.23E-55 | 4.95E-55 |
| GET4 | 3.845999 | 1.232023 | -2.61398 | 1.72E-58 | 8.20E-58 |
| GUSBP11 | 2.811875 | 0.144374 | -2.6675 | 1.36E-62 | 8.98E-62 |
| NAALADL1 | 2.161426 | 0.953379 | -1.20805 | 2.74E-53 | 9.80E-53 |
| PPAT | 1.219985 | 2.257138 | 1.037154 | 1.84E-70 | 3.06E-69 |
| ARL6IP5 | 4.715105 | 5.727483 | 1.012378 | 5.76E-48 | 1.69E-47 |
| ARPP19 | 3.70583 | 4.872678 | 1.166848 | 1.81E-63 | 1.32E-62 |
| HELLS | 1.032266 | 2.071624 | 1.039358 | 2.70E-56 | 1.13E-55 |
| OSGIN1 | 2.581444 | 1.49311 | -1.08833 | 1.63E-43 | 4.21E-43 |
| HIST1H4I | 0.729627 | 3.217949 | 2.488321 | 7.26E-68 | 8.47E-67 |
| HIST1H4H | 1.06507 | 2.349735 | 1.284665 | 2.26E-45 | 6.14E-45 |
| PM20D2 | 2.158753 | 3.303889 | 1.145136 | 1.99E-54 | 7.52E-54 |
| YWHAQ | 5.767055 | 6.890949 | 1.123895 | 1.05E-65 | 9.70E-65 |
| TOP3B | 2.579655 | 0.117044 | -2.46261 | 2.32E-68 | 2.90E-67 |
| KIT | 2.786318 | 1.599313 | -1.187 | 1.24E-43 | 3.20E-43 |
| REV1 | 3.307757 | 2.15267 | -1.15509 | 3.00E-62 | 1.93E-61 |
| HNRNPF | 5.317267 | 6.559248 | 1.241981 | 1.36E-79 | 2.02E-77 |
| CCDC6 | 2.87635 | 4.258953 | 1.382603 | 2.19E-67 | 2.39E-66 |
| BCKDHA | 3.393731 | 1.175975 | -2.21776 | 3.09E-76 | 1.35E-74 |
| RN7SL396P | 0.214837 | 1.411998 | 1.197162 | 1.81E-62 | 1.19E-61 |
| NPEPPS | 4.3703 | 3.192883 | -1.17742 | 3.51E-59 | 1.77E-58 |
| CYP4X1 | 2.718157 | 1.62015 | -1.09801 | 1.51E-19 | 2.34E-19 |
| GLO1 | 4.357559 | 5.997793 | 1.640233 | 1.23E-68 | 1.59E-67 |
| GRIN2D | 0.342012 | 3.206315 | 2.864303 | 2.31E-81 | 6.56E-79 |
| ITGAE | 3.304105 | 1.882836 | -1.42127 | 3.46E-59 | 1.74E-58 |
| AL136126.1 | 0.296253 | 1.402926 | 1.106673 | 4.82E-64 | 3.73E-63 |
| KRT10 | 3.215945 | 4.419574 | 1.203629 | 2.79E-54 | 1.05E-53 |
| SPP1 | 2.042107 | 5.049254 | 3.007147 | 3.61E-56 | 1.50E-55 |
| IGHG3 | 3.657478 | 5.934454 | 2.276976 | 3.72E-26 | 6.54E-26 |
| MYEF2 | 2.966605 | 0.561502 | -2.4051 | 1.82E-70 | 3.02E-69 |
| SRSF11 | 5.820103 | 4.165466 | -1.65464 | 2.54E-51 | 8.39E-51 |
| PROSER1 | 2.87973 | 3.925268 | 1.045538 | 6.84E-71 | 1.20E-69 |
| STK25 | 5.374558 | 3.636843 | -1.73772 | 1.07E-64 | 8.84E-64 |
| RDH13 | 3.086487 | 2.039404 | -1.04708 | 2.12E-40 | 5.04E-40 |
| INO80E | 4.32396 | 2.796157 | -1.5278 | 3.49E-66 | 3.34E-65 |
| KRBOX1 | 1.149449 | 0.149018 | -1.00043 | 2.33E-70 | 3.82E-69 |
| NBPF15 | 2.664496 | 1.662634 | -1.00186 | 5.24E-48 | 1.54E-47 |
| FIGNL1 | 1.200053 | 2.221417 | 1.021365 | 1.98E-67 | 2.19E-66 |
| PRMT2 | 4.189696 | 2.883086 | -1.30661 | 4.13E-74 | 1.23E-72 |
| ANKRD13D | 3.676229 | 2.664905 | -1.01132 | 5.35E-50 | 1.68E-49 |
| AC125232.1 | 2.071304 | 0.996108 | -1.0752 | 4.80E-62 | 3.03E-61 |
| AC114546.3 | 1.816062 | 0.002008 | -1.81405 | 1.78E-78 | 1.35E-76 |
| CDC7 | 0.905111 | 2.034387 | 1.129276 | 1.35E-67 | 1.51E-66 |
| RAD51C | 2.82499 | 1.53392 | -1.29107 | 2.84E-63 | 2.03E-62 |
| RNU4ATAC18P | 0.218786 | 1.270241 | 1.051454 | 1.46E-53 | 5.31E-53 |
| PPP2R3B | 2.295093 | 1.130795 | -1.1643 | 1.08E-53 | 3.94E-53 |
| TRIM52 | 2.558627 | 1.299949 | -1.25868 | 2.07E-58 | 9.79E-58 |
| HERC3 | 3.745878 | 2.123083 | -1.62279 | 7.28E-77 | 3.70E-75 |
| HIST1H2AC | 2.94675 | 4.13654 | 1.18979 | 2.15E-32 | 4.29E-32 |
| CDC26 | 3.656861 | 2.641244 | -1.01562 | 5.12E-69 | 6.91E-68 |
| MACF1 | 4.732027 | 2.787951 | -1.94408 | 5.60E-59 | 2.77E-58 |
| CHI3L1 | 1.628503 | 3.188671 | 1.560168 | 7.43E-30 | 1.41E-29 |
| ITGA2 | 1.215839 | 3.630768 | 2.414929 | 6.75E-70 | 1.03E-68 |
| DNAJB2 | 4.582989 | 3.092441 | -1.49055 | 1.10E-70 | 1.89E-69 |
| PDZD8 | 2.159675 | 3.66893 | 1.509255 | 2.52E-72 | 5.60E-71 |
| CCNE1 | 1.085276 | 2.609286 | 1.52401 | 9.71E-60 | 5.09E-59 |
| AC000089.1 | 0.589983 | 2.084989 | 1.495006 | 1.70E-67 | 1.89E-66 |
| OTX1 | 0.312105 | 1.388371 | 1.076266 | 1.07E-64 | 8.88E-64 |
| GHRL | 5.381155 | 0.916309 | -4.46485 | 7.90E-49 | 2.38E-48 |
| SUN1 | 4.943971 | 3.615079 | -1.32889 | 1.44E-56 | 6.09E-56 |
| TOR1B | 2.7759 | 3.921092 | 1.145191 | 5.78E-70 | 8.95E-69 |
| HJURP | 0.850705 | 2.592524 | 1.741819 | 3.78E-67 | 4.04E-66 |
| AL139095.2 | 0.76148 | 2.29555 | 1.53407 | 2.16E-62 | 1.40E-61 |
| FP671120.5 | 1.120808 | 3.627954 | 2.507146 | 2.43E-53 | 8.72E-53 |
| FBXL8 | 2.965203 | 1.852971 | -1.11223 | 2.10E-38 | 4.77E-38 |
| SLC18B1 | 2.542441 | 3.646899 | 1.104458 | 5.07E-57 | 2.21E-56 |
| SFRP4 | 1.060219 | 3.777945 | 2.717726 | 5.56E-45 | 1.49E-44 |
| ADAM28 | 3.823705 | 2.259982 | -1.56372 | 6.95E-19 | 1.06E-18 |
| ZNHIT3 | 3.496228 | 2.027369 | -1.46886 | 8.66E-57 | 3.72E-56 |
| FGF13 | 1.454593 | 0.211111 | -1.24348 | 6.83E-74 | 1.98E-72 |
| AC090589.1 | 0.366478 | 1.513005 | 1.146527 | 1.00E-64 | 8.35E-64 |
| USF2 | 5.383885 | 4.380533 | -1.00335 | 1.29E-64 | 1.06E-63 |
| COX7A2L | 5.015136 | 3.683079 | -1.33206 | 4.37E-67 | 4.63E-66 |
| MYBL2 | 1.242388 | 5.067197 | 3.824809 | 3.21E-81 | 8.34E-79 |
| EZR | 5.857895 | 7.369524 | 1.511629 | 1.22E-38 | 2.79E-38 |
| CTTNBP2NL | 1.835856 | 2.945688 | 1.109832 | 7.78E-67 | 8.05E-66 |
| AC245595.1 | 2.768472 | 0.463641 | -2.30483 | 1.01E-73 | 2.83E-72 |
| ATP4A | 6.114671 | 0.544653 | -5.57002 | 3.24E-59 | 1.64E-58 |
| PKP3 | 3.145971 | 5.256891 | 2.11092 | 2.35E-53 | 8.45E-53 |
| PRKX | 1.564489 | 2.637224 | 1.072735 | 3.40E-59 | 1.72E-58 |
| ATP5MPL | 5.699398 | 4.399082 | -1.30032 | 1.25E-61 | 7.64E-61 |
| TUBB6 | 4.679457 | 3.554811 | -1.12465 | 1.95E-25 | 3.38E-25 |
| SMN2 | 2.705347 | 0.785707 | -1.91964 | 8.80E-50 | 2.74E-49 |
| PERP | 3.844635 | 6.096216 | 2.25158 | 3.73E-69 | 5.12E-68 |
| QTRT1 | 4.228815 | 3.185157 | -1.04366 | 1.44E-49 | 4.44E-49 |
| AC130304.1 | 2.491193 | 0.002008 | -2.48919 | 1.78E-78 | 1.35E-76 |
| AC107983.1 | 0.785945 | 3.069083 | 2.283137 | 3.15E-67 | 3.39E-66 |
| IGHV3-13 | 1.512959 | 2.591452 | 1.078493 | 6.47E-13 | 8.88E-13 |
| FRMD4A | 2.705033 | 1.307304 | -1.39773 | 1.15E-65 | 1.05E-64 |
| SRPK1 | 2.705284 | 4.142208 | 1.436923 | 2.98E-76 | 1.31E-74 |
| AMACR | 3.059366 | 1.397924 | -1.66144 | 2.28E-43 | 5.85E-43 |
| KHDRBS3 | 2.036106 | 0.92671 | -1.1094 | 2.50E-49 | 7.66E-49 |
| LIMS2 | 4.751918 | 1.962551 | -2.78937 | 6.00E-63 | 4.12E-62 |
| AP002884.1 | 2.084242 | 0.470441 | -1.6138 | 1.20E-77 | 7.31E-76 |
| RLIM | 2.092775 | 3.291498 | 1.198723 | 5.91E-70 | 9.10E-69 |
| ACBD6 | 3.345431 | 2.291727 | -1.0537 | 2.56E-51 | 8.45E-51 |
| CRYAB | 5.476898 | 2.525443 | -2.95145 | 6.97E-59 | 3.42E-58 |
| DOLK | 2.503121 | 3.504483 | 1.001362 | 1.21E-63 | 8.96E-63 |
| VIPR2 | 1.732092 | 0.405518 | -1.32657 | 2.36E-61 | 1.40E-60 |
| ALG13-AS1 | 0.142543 | 1.151936 | 1.009393 | 2.35E-62 | 1.52E-61 |
| IGHG1 | 6.044354 | 8.077426 | 2.033073 | 5.62E-19 | 8.62E-19 |
| ACIN1 | 5.25398 | 4.079211 | -1.17477 | 1.98E-54 | 7.47E-54 |
| PYGB | 4.536051 | 6.121557 | 1.585507 | 6.09E-52 | 2.07E-51 |
| IFIT3 | 2.128778 | 4.007067 | 1.878289 | 1.10E-58 | 5.32E-58 |
| LY6E | 4.8237 | 7.216253 | 2.392552 | 9.65E-70 | 1.44E-68 |
| RPP21 | 4.306853 | 2.770997 | -1.53586 | 8.29E-58 | 3.76E-57 |
| LOXL2 | 2.203639 | 3.697369 | 1.49373 | 7.40E-57 | 3.20E-56 |
| IFT43 | 3.392239 | 2.099937 | -1.2923 | 1.92E-69 | 2.76E-68 |
| HM13-IT1 | 0.645454 | 1.969211 | 1.323756 | 2.35E-70 | 3.85E-69 |
| ZBTB2 | 1.999552 | 3.18514 | 1.185589 | 7.54E-69 | 1.00E-67 |
| CDC42P6 | 0.969819 | 2.159909 | 1.190091 | 1.76E-54 | 6.66E-54 |
| RPL17-C18orf32 | 1.902561 | 0.036804 | -1.86576 | 6.59E-66 | 6.14E-65 |
| AKT1 | 5.01655 | 3.771195 | -1.24535 | 1.46E-66 | 1.46E-65 |
| SERGEF | 3.510913 | 1.830199 | -1.68071 | 9.63E-71 | 1.67E-69 |
| ATOH8 | 2.733613 | 0.66021 | -2.0734 | 4.75E-76 | 2.00E-74 |
| CRYZL2P-SEC16B | 1.316011 | 0.271555 | -1.04446 | 1.27E-55 | 5.11E-55 |
| NEIL1 | 3.785348 | 1.174021 | -2.61133 | 2.56E-69 | 3.63E-68 |
| HINFP | 2.994096 | 1.369652 | -1.62444 | 7.29E-62 | 4.54E-61 |
| HSPB6 | 5.850507 | 4.327448 | -1.52306 | 6.49E-14 | 9.07E-14 |
| AC110749.1 | 0.283753 | 1.348196 | 1.064443 | 3.04E-60 | 1.66E-59 |
| TPT1-AS1 | 2.993038 | 1.311455 | -1.68158 | 1.22E-58 | 5.89E-58 |
| MIR647 | 0.372205 | 3.628668 | 3.256462 | 1.75E-77 | 1.02E-75 |
| MXRA7 | 4.875245 | 3.057583 | -1.81766 | 1.55E-49 | 4.78E-49 |
| ZNF121 | 1.819606 | 2.866758 | 1.047152 | 2.55E-69 | 3.61E-68 |
| AC002398.2 | 1.684455 | 0.314305 | -1.37015 | 2.58E-38 | 5.84E-38 |
| CKS2 | 2.93208 | 6.302196 | 3.370116 | 9.33E-81 | 2.09E-78 |
| SERPINA1 | 4.780569 | 6.001127 | 1.220558 | 9.27E-10 | 1.19E-09 |
| AC099560.2 | 3.061231 | 4.090766 | 1.029535 | 1.91E-30 | 3.65E-30 |
| RN7SL1 | 5.013626 | 0.002008 | -5.01162 | 1.78E-78 | 1.35E-76 |
| MMGT1 | 2.741721 | 3.889883 | 1.148163 | 2.82E-63 | 2.01E-62 |
| DGKD | 4.949045 | 2.758483 | -2.19056 | 1.07E-56 | 4.56E-56 |
| ZSWIM4 | 1.493472 | 2.994145 | 1.500674 | 7.20E-75 | 2.49E-73 |
| AC004890.1 | 0.333462 | 1.435191 | 1.101728 | 2.90E-55 | 1.15E-54 |
| HMGA1P8 | 0.458683 | 1.772028 | 1.313345 | 2.43E-67 | 2.64E-66 |
| CHCHD2 | 6.904617 | 8.25533 | 1.350712 | 2.19E-77 | 1.25E-75 |
| CIB1 | 5.284958 | 6.613336 | 1.328378 | 1.94E-62 | 1.27E-61 |
| PYGM | 1.749705 | 0.553976 | -1.19573 | 3.40E-52 | 1.16E-51 |
| IGLV1-44 | 3.959236 | 5.533562 | 1.574326 | 6.39E-12 | 8.61E-12 |
| KIF2C | 1.02542 | 3.243085 | 2.217665 | 1.21E-76 | 5.77E-75 |
| PCCA | 3.689079 | 1.872138 | -1.81694 | 4.17E-72 | 8.84E-71 |
| AC018629.1 | 0.23831 | 1.474344 | 1.236034 | 5.86E-44 | 1.53E-43 |
| CD93 | 2.643665 | 3.760956 | 1.117291 | 3.61E-39 | 8.35E-39 |
| ZMPSTE24 | 3.26789 | 4.501283 | 1.233393 | 4.37E-72 | 9.19E-71 |
| FBP2 | 3.049084 | 0.797862 | -2.25122 | 2.09E-26 | 3.70E-26 |
| REP15 | 2.481068 | 0.614693 | -1.86637 | 3.53E-47 | 1.01E-46 |
| TSPAN4 | 3.769853 | 2.377169 | -1.39268 | 2.27E-57 | 1.01E-56 |
| MIR210HG | 2.298135 | 1.156866 | -1.14127 | 1.25E-41 | 3.06E-41 |
| RPL23AP18 | 0.282252 | 1.416677 | 1.134425 | 1.61E-66 | 1.61E-65 |
| PYURF | 3.900245 | 2.164205 | -1.73604 | 2.10E-62 | 1.37E-61 |
| CRK | 3.556656 | 4.735589 | 1.178933 | 5.28E-66 | 4.97E-65 |
| PABPC3 | 0.333581 | 1.494212 | 1.160631 | 3.80E-73 | 9.60E-72 |
| RNU7-41P | 0.291044 | 1.747151 | 1.456108 | 1.09E-39 | 2.55E-39 |
| PRR15L | 4.17238 | 5.798352 | 1.625972 | 6.22E-22 | 1.01E-21 |
| RNASEK | 6.202753 | 2.486552 | -3.7162 | 4.14E-71 | 7.51E-70 |
| HK2 | 2.33537 | 3.620915 | 1.285545 | 7.17E-34 | 1.48E-33 |
| MON2 | 3.24725 | 2.070062 | -1.17719 | 3.30E-61 | 1.94E-60 |
| RPL31 | 8.934235 | 6.357758 | -2.57648 | 3.20E-60 | 1.74E-59 |
| NBL1 | 5.3027 | 4.119235 | -1.18346 | 1.22E-47 | 3.54E-47 |
| RECQL4 | 1.657143 | 3.167374 | 1.510232 | 5.24E-57 | 2.28E-56 |
| SUV39H2 | 1.230805 | 2.313394 | 1.082588 | 5.54E-78 | 3.69E-76 |
| ATP6V1A | 2.83457 | 4.026251 | 1.191681 | 1.04E-72 | 2.46E-71 |
| GBA2 | 5.232404 | 3.86306 | -1.36934 | 3.00E-65 | 2.63E-64 |
| TFAP2A | 0.637995 | 1.845608 | 1.207613 | 8.84E-43 | 2.23E-42 |
| AC092718.4 | 1.354134 | 3.377523 | 2.023389 | 1.98E-77 | 1.14E-75 |
| C2orf69 | 1.698288 | 2.831001 | 1.132712 | 1.80E-67 | 2.00E-66 |
| SETD6 | 2.938186 | 1.606215 | -1.33197 | 4.85E-56 | 2.00E-55 |
| CREB3L1 | 4.334803 | 5.913678 | 1.578875 | 7.83E-26 | 1.37E-25 |
| GATA6-AS1 | 4.011658 | 1.244826 | -2.76683 | 1.30E-33 | 2.67E-33 |
| GALNT15 | 2.321917 | 0.750735 | -1.57118 | 2.11E-56 | 8.89E-56 |
| RPL41P5 | 0.974648 | 5.722362 | 4.747714 | 9.68E-56 | 3.92E-55 |
| SNORD12B | 0.127263 | 2.059033 | 1.931771 | 9.84E-79 | 1.06E-76 |
| AC129492.1 | 5.575114 | 3.105849 | -2.46926 | 7.99E-72 | 1.63E-70 |
| MT-CO1 | 13.30433 | 14.45521 | 1.150884 | 4.49E-55 | 1.75E-54 |
| LCN10 | 2.162019 | 0.047561 | -2.11446 | 5.99E-82 | 2.06E-79 |
| AL161787.1 | 1.264513 | 4.324637 | 3.060124 | 5.04E-61 | 2.92E-60 |
| DMKN | 3.752562 | 1.582181 | -2.17038 | 2.59E-44 | 6.80E-44 |
| LAMB3 | 3.195948 | 5.498813 | 2.302865 | 5.33E-50 | 1.67E-49 |
| EXOSC5 | 2.864726 | 4.086459 | 1.221733 | 1.26E-64 | 1.04E-63 |
| AC068888.1 | 2.365134 | 1.035301 | -1.32983 | 3.82E-61 | 2.24E-60 |
| IL18 | 2.373816 | 3.424075 | 1.05026 | 4.74E-37 | 1.04E-36 |
| RNU6-925P | 0.091271 | 1.208739 | 1.117468 | 1.67E-67 | 1.86E-66 |
| YBX3 | 6.114928 | 4.536324 | -1.5786 | 7.57E-58 | 3.45E-57 |
| H2AFJ | 5.356736 | 3.773145 | -1.58359 | 6.97E-70 | 1.06E-68 |
| AC012651.1 | 1.367443 | 0.231426 | -1.13602 | 4.41E-62 | 2.79E-61 |
| HABP2 | 1.299837 | 2.325025 | 1.025188 | 1.61E-10 | 2.11E-10 |
| SOWAHC | 1.822043 | 3.272265 | 1.450222 | 4.66E-56 | 1.92E-55 |
| NBPF26 | 1.862443 | 0.776951 | -1.08549 | 6.32E-53 | 2.22E-52 |
| MIR196A1 | 0.049449 | 1.233186 | 1.183736 | 8.76E-28 | 1.59E-27 |
| CRIP2 | 4.975121 | 3.740076 | -1.23504 | 4.14E-44 | 1.08E-43 |
| BRICD5 | 2.140378 | 0.8441 | -1.29628 | 2.27E-53 | 8.16E-53 |
| SHF | 1.894063 | 0.885414 | -1.00865 | 2.22E-54 | 8.37E-54 |
| AARSD1 | 3.672565 | 0.920969 | -2.7516 | 2.13E-49 | 6.54E-49 |
| TRAPPC2L | 4.498757 | 2.992248 | -1.50651 | 2.75E-63 | 1.97E-62 |
| CPXM1 | 0.902579 | 2.773733 | 1.871154 | 4.68E-66 | 4.45E-65 |
| AC073046.1 | 0.806483 | 2.230921 | 1.424438 | 2.97E-59 | 1.51E-58 |
| AC048382.2 | 1.619044 | 0.288893 | -1.33015 | 1.31E-60 | 7.34E-60 |
| NMI | 2.466655 | 3.686755 | 1.2201 | 2.63E-68 | 3.25E-67 |
| AC138035.1 | 2.057542 | 0.232776 | -1.82477 | 3.95E-63 | 2.77E-62 |
| PSMC1P5 | 3.179203 | 0.19319 | -2.98601 | 2.74E-49 | 8.39E-49 |
| SLC9A4 | 2.865546 | 0.931708 | -1.93384 | 8.71E-30 | 1.65E-29 |
| ANKZF1 | 4.312889 | 2.523796 | -1.78909 | 9.21E-50 | 2.86E-49 |
| LAD1 | 3.708432 | 6.170037 | 2.461604 | 1.56E-59 | 8.06E-59 |
| RNU6-387P | 0.080245 | 1.144564 | 1.064319 | 1.60E-64 | 1.30E-63 |
| MOGAT3 | 0.227273 | 1.57284 | 1.345567 | 1.07E-61 | 6.55E-61 |
| NRAS | 2.406845 | 4.303005 | 1.89616 | 2.47E-77 | 1.39E-75 |
| CA9 | 5.534473 | 2.973688 | -2.56078 | 7.75E-23 | 1.28E-22 |
| MRPL15 | 3.940354 | 5.243878 | 1.303524 | 2.15E-73 | 5.69E-72 |
| TPT1P6 | 0.294832 | 1.343476 | 1.048643 | 3.28E-60 | 1.79E-59 |
| ACSL1 | 4.263124 | 3.230305 | -1.03282 | 3.12E-49 | 9.53E-49 |
| COL4A5 | 3.395869 | 1.397502 | -1.99837 | 4.81E-58 | 2.22E-57 |
| MOCS1 | 2.729233 | 1.573469 | -1.15576 | 1.44E-60 | 8.04E-60 |
| SELENOI | 1.712094 | 3.151016 | 1.438922 | 1.03E-70 | 1.77E-69 |
| CXCR3 | 0.823717 | 2.106499 | 1.282782 | 1.60E-47 | 4.62E-47 |
| MAPK10 | 1.971732 | 0.516462 | -1.45527 | 2.69E-68 | 3.32E-67 |
| TNFRSF10A | 1.29988 | 2.955119 | 1.655239 | 3.34E-74 | 1.01E-72 |
| AGRN | 3.495458 | 5.195955 | 1.700497 | 1.20E-72 | 2.80E-71 |
| FXYD1 | 3.862303 | 0.426072 | -3.43623 | 2.00E-82 | 9.70E-80 |
| ARMCX7P | 1.405409 | 0.072287 | -1.33312 | 8.60E-50 | 2.68E-49 |
| AC073896.4 | 1.595624 | 2.726639 | 1.131014 | 1.02E-57 | 4.59E-57 |
| GALT | 3.922542 | 2.355055 | -1.56749 | 8.27E-71 | 1.44E-69 |
| ATP9B | 2.84838 | 1.432855 | -1.41552 | 8.66E-73 | 2.08E-71 |
| ACACB | 3.483515 | 1.537907 | -1.94561 | 9.53E-72 | 1.91E-70 |
| HPCAL1 | 4.53253 | 3.329236 | -1.20329 | 1.27E-40 | 3.02E-40 |
| ELF1 | 3.574273 | 4.750321 | 1.176048 | 4.53E-70 | 7.13E-69 |
| SNURF | 1.482136 | 0.243443 | -1.23869 | 1.52E-74 | 4.94E-73 |
| USP38 | 1.772256 | 2.952398 | 1.180142 | 5.11E-64 | 3.94E-63 |
| MIR5094 | 0.081728 | 1.194209 | 1.112481 | 2.29E-55 | 9.07E-55 |
| AC114801.3 | 0.468032 | 2.354107 | 1.886075 | 1.18E-55 | 4.74E-55 |
| MYL5 | 2.785872 | 1.315623 | -1.47025 | 1.73E-65 | 1.55E-64 |
| AC018645.2 | 1.783413 | 2.880454 | 1.097041 | 8.85E-60 | 4.65E-59 |
| SNORD62B | 0.26983 | 2.433088 | 2.163258 | 2.59E-72 | 5.73E-71 |
| THUMPD3-AS1 | 3.358821 | 1.501321 | -1.8575 | 3.66E-51 | 1.20E-50 |
| NQO2 | 3.522598 | 1.852073 | -1.67052 | 5.42E-59 | 2.69E-58 |
| PLA2G1B | 1.7028 | 0.385059 | -1.31774 | 2.41E-51 | 7.98E-51 |
| AC132217.1 | 2.528624 | 0.002008 | -2.52662 | 1.78E-78 | 1.35E-76 |
| KNL1 | 0.560173 | 1.943649 | 1.383476 | 1.95E-70 | 3.22E-69 |
| APBB3 | 3.769324 | 1.519251 | -2.25007 | 1.24E-61 | 7.55E-61 |
| SNORD14A | 0.230642 | 2.239854 | 2.009212 | 9.03E-73 | 2.15E-71 |
| CCNA2 | 1.372141 | 3.981232 | 2.609091 | 2.60E-77 | 1.46E-75 |
| PCNA | 4.088877 | 6.71514 | 2.626263 | 5.01E-84 | 9.97E-81 |
| KLF3 | 3.620927 | 4.772365 | 1.151439 | 6.79E-53 | 2.38E-52 |
| PAK2 | 2.992462 | 4.726101 | 1.733639 | 4.13E-79 | 5.36E-77 |
| CSAG1 | 0.093781 | 1.33212 | 1.238338 | 1.80E-18 | 2.74E-18 |
| UHRF1 | 0.819898 | 2.513219 | 1.693322 | 8.01E-71 | 1.40E-69 |
| IGKV3-11 | 5.468848 | 6.548925 | 1.080077 | 0.00023 | 0.00026 |
| PHKA2 | 3.266085 | 2.069203 | -1.19688 | 2.49E-52 | 8.56E-52 |
| RRP8 | 2.887819 | 1.740208 | -1.14761 | 1.36E-64 | 1.11E-63 |
| ROGDI | 4.258649 | 2.752559 | -1.50609 | 3.12E-77 | 1.71E-75 |
| MAOA | 4.70763 | 3.443366 | -1.26426 | 3.82E-38 | 8.62E-38 |
| GPD2 | 2.241072 | 3.346559 | 1.105487 | 3.99E-55 | 1.56E-54 |
| ATAD5 | 0.608361 | 1.630537 | 1.022176 | 2.09E-79 | 2.99E-77 |
| RPL10 | 9.815939 | 8.408547 | -1.40739 | 5.27E-57 | 2.29E-56 |
| PRR4 | 1.922869 | 0.62176 | -1.30111 | 3.07E-55 | 1.21E-54 |
| PRSS3 | 2.567757 | 5.085988 | 2.51823 | 1.64E-46 | 4.61E-46 |
| SMIM12 | 3.482459 | 2.282649 | -1.19981 | 1.20E-70 | 2.04E-69 |
| TRIP13 | 0.841926 | 2.804097 | 1.962171 | 2.84E-78 | 2.01E-76 |
| ENOSF1 | 4.276364 | 2.25575 | -2.02061 | 5.07E-61 | 2.93E-60 |
| ZFP36L2 | 5.434464 | 7.446231 | 2.011766 | 4.51E-72 | 9.45E-71 |
| JRK | 3.032474 | 1.663886 | -1.36859 | 1.48E-26 | 2.63E-26 |
| B3GNT7 | 2.699197 | 3.833269 | 1.134072 | 2.26E-16 | 3.30E-16 |
| ISG20 | 3.931746 | 2.382127 | -1.54962 | 1.85E-38 | 4.22E-38 |
| BCL10 | 2.343733 | 3.641177 | 1.297443 | 6.05E-56 | 2.47E-55 |
| EMC6 | 4.04762 | 2.414588 | -1.63303 | 3.50E-69 | 4.83E-68 |
| NPC1L1 | 0.720623 | 1.730881 | 1.010259 | 1.74E-06 | 2.08E-06 |
| ABCC5 | 3.95053 | 2.092418 | -1.85811 | 2.89E-63 | 2.06E-62 |
| GLTPD2 | 0.613954 | 1.994086 | 1.380132 | 5.02E-57 | 2.19E-56 |
| SMUG1 | 3.623509 | 2.578916 | -1.04459 | 2.14E-51 | 7.11E-51 |
| AC008443.1 | 2.021888 | 0.617473 | -1.40441 | 1.08E-58 | 5.22E-58 |
| LRRFIP2 | 3.752716 | 2.341469 | -1.41125 | 9.67E-71 | 1.68E-69 |
| MTND6P4 | 0.804456 | 2.837967 | 2.033511 | 1.47E-54 | 5.60E-54 |
| SLC38A11 | 1.939288 | 0.768931 | -1.17036 | 3.06E-33 | 6.22E-33 |
| LYN | 2.247718 | 4.110818 | 1.863099 | 2.18E-71 | 4.11E-70 |
| MTFR1L | 4.577516 | 2.697507 | -1.88001 | 1.85E-80 | 3.72E-78 |
| CASC9 | 0.240604 | 1.762858 | 1.522254 | 4.91E-42 | 1.22E-41 |
| CXorf40B | 3.584193 | 2.472243 | -1.11195 | 1.56E-64 | 1.27E-63 |
| AP2B1 | 4.346832 | 5.604436 | 1.257604 | 4.70E-69 | 6.37E-68 |
| TACR2 | 2.539598 | 1.528918 | -1.01068 | 1.14E-10 | 1.50E-10 |
| ZNFX1 | 2.558114 | 4.188259 | 1.630145 | 3.32E-76 | 1.44E-74 |
| LINC-PINT | 2.305559 | 1.135717 | -1.16984 | 1.18E-56 | 5.04E-56 |
| SRP9 | 5.472273 | 6.629113 | 1.156839 | 3.76E-73 | 9.51E-72 |
| NRARP | 2.236652 | 5.000786 | 2.764134 | 3.23E-72 | 7.02E-71 |
| PIGR | 4.617803 | 6.742582 | 2.124779 | 2.66E-15 | 3.81E-15 |
| ITGA7 | 4.300041 | 2.218969 | -2.08107 | 9.96E-49 | 3.00E-48 |
| AC107956.1 | 1.361016 | 2.727881 | 1.366866 | 2.32E-45 | 6.29E-45 |
| SMIM11B | 3.641598 | 0.023523 | -3.61807 | 3.16E-76 | 1.38E-74 |
| GPS2 | 4.468454 | 1.897957 | -2.5705 | 8.03E-67 | 8.29E-66 |
| CDH3 | 0.943182 | 2.871998 | 1.928815 | 6.51E-58 | 2.98E-57 |
| AC099850.3 | 0.476594 | 3.255533 | 2.778939 | 1.45E-82 | 8.57E-80 |
| SLC12A9-AS1 | 0.26082 | 1.373583 | 1.112763 | 3.66E-72 | 7.92E-71 |
| FAM189A2 | 3.347532 | 1.254026 | -2.09351 | 2.00E-65 | 1.78E-64 |
| PGC | 10.32159 | 5.769637 | -4.55195 | 6.43E-28 | 1.17E-27 |
| RHOD | 3.121173 | 4.162843 | 1.04167 | 1.11E-29 | 2.10E-29 |
| RHPN2 | 2.620459 | 4.425153 | 1.804694 | 3.70E-46 | 1.03E-45 |
| RPL27A | 8.698133 | 6.647742 | -2.05039 | 9.46E-63 | 6.37E-62 |
| FAM102B | 1.51075 | 2.845542 | 1.334792 | 2.55E-70 | 4.16E-69 |
| CSE1L | 3.449542 | 5.49046 | 2.040918 | 1.36E-83 | 1.56E-80 |
| PLS1 | 2.177603 | 4.876844 | 2.699241 | 1.77E-64 | 1.44E-63 |
| UBE2D1 | 2.322522 | 3.485342 | 1.16282 | 8.89E-70 | 1.34E-68 |
| WDR92 | 1.457462 | 0.375683 | -1.08178 | 3.04E-58 | 1.42E-57 |
| NDST2 | 2.90706 | 0.530053 | -2.37701 | 5.70E-65 | 4.86E-64 |
| RGS16 | 1.746193 | 2.795234 | 1.049041 | 5.30E-29 | 9.85E-29 |
| E2F8 | 1.153508 | 2.465497 | 1.311988 | 3.62E-47 | 1.04E-46 |
| E2F2 | 0.605046 | 1.81438 | 1.209334 | 6.05E-56 | 2.47E-55 |
| PI16 | 2.719555 | 1.137585 | -1.58197 | 4.43E-33 | 8.98E-33 |
| AC010326.3 | 1.59851 | 2.760209 | 1.1617 | 8.45E-57 | 3.63E-56 |
| HOXA13 | 0.109458 | 2.061033 | 1.951575 | 5.33E-65 | 4.57E-64 |
| KIF11 | 0.903084 | 3.47669 | 2.573606 | 1.08E-81 | 3.44E-79 |
| ZNF7 | 2.75982 | 1.667454 | -1.09237 | 6.14E-49 | 1.86E-48 |
| PMM1 | 4.655467 | 2.070703 | -2.58476 | 2.09E-80 | 4.16E-78 |
| COL11A1 | 0.264603 | 1.516734 | 1.252131 | 2.81E-30 | 5.36E-30 |
| RPS19P7 | 0.255472 | 1.547653 | 1.292181 | 4.26E-63 | 2.98E-62 |
| ATP5ME | 7.100693 | 5.898852 | -1.20184 | 6.97E-61 | 3.99E-60 |
| SNORA71A | 0.188779 | 1.363878 | 1.175099 | 5.08E-64 | 3.92E-63 |
| MAP3K1 | 1.773509 | 2.810287 | 1.036778 | 9.57E-66 | 8.83E-65 |
| DUSP14 | 2.032868 | 3.05676 | 1.023892 | 3.54E-44 | 9.29E-44 |
| TMEM60 | 2.837296 | 3.951362 | 1.114066 | 9.83E-70 | 1.47E-68 |
| POLL | 3.431416 | 2.312162 | -1.11925 | 3.29E-70 | 5.29E-69 |
| ZMAT5 | 3.050504 | 1.820823 | -1.22968 | 1.50E-72 | 3.44E-71 |
| RNA5-8SN3 | 6.534022 | 0.13607 | -6.39795 | 1.06E-64 | 8.81E-64 |
| SERINC2 | 4.312432 | 6.366859 | 2.054426 | 6.91E-41 | 1.66E-40 |
| CCDC86 | 2.961111 | 4.15835 | 1.197239 | 1.98E-68 | 2.49E-67 |
| PLEKHH3 | 3.982581 | 2.611717 | -1.37086 | 1.92E-68 | 2.42E-67 |
| NDUFA6-DT | 1.801571 | 0.38205 | -1.41952 | 8.88E-79 | 9.88E-77 |
| NOTCH3 | 2.615469 | 4.632913 | 2.017443 | 1.56E-63 | 1.14E-62 |
| COPA | 4.424341 | 5.763003 | 1.338662 | 1.13E-78 | 1.19E-76 |
| COL5A2 | 3.081895 | 4.861637 | 1.779741 | 9.64E-56 | 3.90E-55 |
| ADRM1 | 4.900971 | 6.013346 | 1.112375 | 2.41E-73 | 6.32E-72 |
| ATP13A4 | 2.014727 | 0.700322 | -1.3144 | 1.53E-34 | 3.19E-34 |
| CTC1 | 3.395407 | 2.189532 | -1.20587 | 1.02E-63 | 7.61E-63 |
| CZIB | 4.396732 | 2.892674 | -1.50406 | 8.50E-73 | 2.04E-71 |
| ANO7 | 2.625931 | 0.999772 | -1.62616 | 1.39E-26 | 2.46E-26 |
| RPL23P8 | 0.45865 | 1.786297 | 1.327647 | 5.53E-64 | 4.23E-63 |
| AC027796.3 | 1.334784 | 0.043703 | -1.29108 | 2.26E-65 | 2.01E-64 |
| COL3A1 | 5.529381 | 8.198852 | 2.669471 | 2.73E-61 | 1.61E-60 |
| CXCL17 | 5.687929 | 3.863333 | -1.8246 | 1.55E-11 | 2.07E-11 |
| PALM3 | 3.008581 | 1.597394 | -1.41119 | 4.07E-18 | 6.14E-18 |
| TGIF2 | 2.047938 | 3.240553 | 1.192615 | 1.03E-71 | 2.05E-70 |
| TTN-AS1 | 2.325028 | 0.194499 | -2.13053 | 9.78E-67 | 1.00E-65 |
| SEC11A | 5.037767 | 4.017409 | -1.02036 | 8.82E-49 | 2.66E-48 |
| RPS15AP38 | 0.728422 | 2.129638 | 1.401216 | 9.17E-61 | 5.21E-60 |
| PNPO | 2.449452 | 3.671733 | 1.222281 | 3.47E-71 | 6.40E-70 |
| PSMC4 | 4.548505 | 5.560651 | 1.012146 | 4.35E-72 | 9.16E-71 |
| ZWINT | 1.8585 | 4.138039 | 2.279539 | 2.03E-71 | 3.85E-70 |
| NBPF19 | 1.587885 | 0.362463 | -1.22542 | 1.58E-63 | 1.16E-62 |
| CANT1 | 3.820869 | 5.128582 | 1.307713 | 9.76E-56 | 3.95E-55 |
| LINC00963 | 3.454534 | 2.404959 | -1.04957 | 1.51E-53 | 5.48E-53 |
| AASS | 2.703441 | 0.957284 | -1.74616 | 1.88E-68 | 2.38E-67 |
| RN7SL3 | 0.568877 | 2.685578 | 2.116701 | 2.06E-58 | 9.76E-58 |
| FAM193B | 4.811974 | 2.429449 | -2.38252 | 7.21E-59 | 3.54E-58 |
| CEACAM6 | 1.210217 | 6.510499 | 5.300281 | 1.40E-66 | 1.41E-65 |
| AC008764.4 | 1.263134 | 0.121799 | -1.14134 | 7.22E-38 | 1.62E-37 |
| ISG15 | 3.544304 | 5.981787 | 2.437483 | 3.61E-63 | 2.54E-62 |
| AC023794.4 | 2.332796 | 0.579141 | -1.75365 | 1.87E-73 | 4.99E-72 |
| RPL13AP7 | 0.736466 | 1.979976 | 1.243509 | 6.38E-61 | 3.66E-60 |
| AL669983.1 | 0.321665 | 1.785629 | 1.463964 | 1.95E-64 | 1.57E-63 |
| EXO1 | 0.472094 | 2.004319 | 1.532225 | 3.09E-75 | 1.14E-73 |
| GTF2IRD2B | 3.398646 | 0.966198 | -2.43245 | 6.16E-70 | 9.46E-69 |
| MAPK12 | 2.083014 | 0.7425 | -1.34051 | 8.99E-54 | 3.29E-53 |
| IDH3A | 3.580023 | 2.428144 | -1.15188 | 4.76E-59 | 2.37E-58 |
| AC016735.1 | 0.306807 | 2.182999 | 1.876192 | 2.54E-69 | 3.60E-68 |
| CRYBB2P1 | 2.625905 | 1.319069 | -1.30684 | 4.87E-56 | 2.00E-55 |
| RALGAPA1 | 2.839281 | 1.675935 | -1.16335 | 1.80E-64 | 1.45E-63 |
| RPL10P15 | 0.631844 | 2.212514 | 1.58067 | 2.89E-63 | 2.06E-62 |
| MIR3609 | 0.545947 | 3.610116 | 3.064169 | 2.03E-63 | 1.47E-62 |
| PGM5 | 3.777644 | 1.621374 | -2.15627 | 5.07E-43 | 1.29E-42 |
| SMOC2 | 4.679833 | 3.622502 | -1.05733 | 1.95E-09 | 2.50E-09 |
| PSPHP1 | 0.38676 | 2.07928 | 1.69252 | 8.43E-16 | 1.22E-15 |
| AP1S1 | 3.750515 | 5.001942 | 1.251426 | 4.87E-58 | 2.25E-57 |
| AC092809.3 | 0.431534 | 1.698578 | 1.267044 | 5.24E-64 | 4.03E-63 |
| SNORD100 | 0.183195 | 1.92992 | 1.746725 | 4.86E-72 | 1.01E-70 |
| TM7SF2 | 3.799968 | 2.455364 | -1.3446 | 2.61E-32 | 5.20E-32 |
| MAT2A | 6.354866 | 4.996349 | -1.35852 | 3.75E-56 | 1.55E-55 |
| EFNA3 | 0.916203 | 2.414067 | 1.497863 | 6.09E-52 | 2.07E-51 |
| TP53I13 | 4.193458 | 3.158604 | -1.03485 | 3.16E-49 | 9.63E-49 |
| RIN2 | 2.597877 | 3.834474 | 1.236596 | 7.75E-69 | 1.03E-67 |
| ABCD1 | 2.180588 | 3.592143 | 1.411555 | 8.93E-59 | 4.35E-58 |
| ATL3 | 3.10534 | 4.147891 | 1.042551 | 9.01E-45 | 2.40E-44 |
| AP003396.5 | 1.515645 | 0.002008 | -1.51364 | 1.78E-78 | 1.35E-76 |
| JAK1 | 4.160169 | 5.196673 | 1.036504 | 2.78E-56 | 1.16E-55 |
| IGKV1-6 | 2.832901 | 3.987488 | 1.154588 | 6.66E-10 | 8.60E-10 |
| IGKV1-27 | 3.166438 | 4.323425 | 1.156987 | 1.16E-08 | 1.47E-08 |
| PCBP2 | 7.020792 | 4.915537 | -2.10525 | 4.62E-56 | 1.90E-55 |
| PAPSS2 | 2.050863 | 3.320503 | 1.269639 | 7.93E-50 | 2.47E-49 |
| FOXN2 | 1.507493 | 2.882461 | 1.374968 | 3.87E-80 | 6.73E-78 |
| KRT8 | 6.869742 | 8.712176 | 1.842434 | 6.71E-39 | 1.54E-38 |
| ADRA2A | 1.215717 | 2.751534 | 1.535817 | 5.15E-23 | 8.52E-23 |
| CYBB | 1.735835 | 3.644736 | 1.908901 | 4.05E-58 | 1.88E-57 |
| AC090543.2 | 0.364455 | 1.850181 | 1.485726 | 3.67E-67 | 3.92E-66 |
| ERVK13-1 | 2.324626 | 0.873472 | -1.45115 | 6.66E-62 | 4.17E-61 |
| COL12A1 | 2.754697 | 4.390708 | 1.636011 | 1.03E-42 | 2.59E-42 |
| ACSL4 | 2.725958 | 4.035514 | 1.309555 | 3.49E-61 | 2.05E-60 |
| ETV4 | 0.974622 | 3.51277 | 2.538148 | 1.84E-72 | 4.16E-71 |
| IGHJ3P | 0.525537 | 2.704262 | 2.178725 | 1.43E-41 | 3.49E-41 |
| AC011462.1 | 3.520288 | 5.012414 | 1.492126 | 4.30E-51 | 1.41E-50 |
| RNU4-47P | 0.295693 | 1.437053 | 1.14136 | 7.44E-58 | 3.39E-57 |
| RTEL1 | 2.168865 | 0.381152 | -1.78771 | 6.51E-57 | 2.82E-56 |
| AP002784.2 | 0.696438 | 1.806035 | 1.109597 | 1.25E-60 | 7.01E-60 |
| SKIL | 2.2642 | 3.831044 | 1.566844 | 7.05E-73 | 1.71E-71 |
| DNTTIP1 | 3.606224 | 4.764118 | 1.157894 | 2.57E-66 | 2.50E-65 |
| NFYA | 2.366585 | 3.481461 | 1.114876 | 1.28E-76 | 6.08E-75 |
| AC011603.2 | 3.007057 | 0.166681 | -2.84038 | 2.28E-54 | 8.58E-54 |
| PRUNE2 | 3.7019 | 1.848631 | -1.85327 | 1.78E-32 | 3.56E-32 |
| AC079329.1 | 1.482986 | 0.002008 | -1.48098 | 1.78E-78 | 1.35E-76 |
| CITED4 | 2.873409 | 3.97391 | 1.100502 | 7.39E-27 | 1.32E-26 |
| SCAF1 | 3.534828 | 4.849065 | 1.314237 | 4.15E-63 | 2.90E-62 |
| ARL6IP4 | 5.609454 | 0.995685 | -4.61377 | 5.46E-67 | 5.71E-66 |
| PABPC1 | 7.628338 | 9.218726 | 1.590388 | 6.74E-77 | 3.46E-75 |
| RPL35P5 | 1.407795 | 3.351524 | 1.943729 | 2.81E-56 | 1.18E-55 |
| VDAC1P8 | 1.747004 | 0.63808 | -1.10892 | 1.20E-48 | 3.61E-48 |
| P3H2 | 2.774316 | 1.736338 | -1.03798 | 2.57E-28 | 4.71E-28 |
| ARGLU1 | 5.808537 | 4.16509 | -1.64345 | 4.57E-55 | 1.78E-54 |
| CPA2 | 4.034482 | 0.66618 | -3.3683 | 3.51E-49 | 1.07E-48 |
| IER5L | 2.215967 | 3.836277 | 1.62031 | 2.05E-60 | 1.13E-59 |
| H2AFX | 3.314066 | 5.766732 | 2.452666 | 1.10E-76 | 5.34E-75 |
| RPS27L | 5.639147 | 3.205783 | -2.43336 | 2.80E-65 | 2.45E-64 |
| LSM3 | 2.622526 | 3.911018 | 1.288492 | 3.62E-59 | 1.82E-58 |
| GRASP | 3.106583 | 2.009213 | -1.09737 | 4.34E-50 | 1.37E-49 |
| ZGPAT | 3.069918 | 1.84408 | -1.22584 | 2.55E-58 | 1.20E-57 |
| GARS-DT | 3.635571 | 0.885256 | -2.75032 | 6.87E-54 | 2.53E-53 |
| EHF | 2.694392 | 4.507506 | 1.813114 | 1.07E-41 | 2.63E-41 |
| ECHS1 | 5.191083 | 6.210666 | 1.019583 | 3.67E-58 | 1.71E-57 |
| EDEM2 | 3.004747 | 4.509538 | 1.504792 | 7.70E-72 | 1.57E-70 |
| SNORD19 | 0.094415 | 1.098552 | 1.004137 | 1.24E-49 | 3.85E-49 |
| GTF2IP23 | 0.303951 | 1.484185 | 1.180234 | 1.52E-72 | 3.49E-71 |
| POLE4 | 4.377418 | 3.101231 | -1.27619 | 8.74E-60 | 4.60E-59 |
| UCA1 | 0.281171 | 2.087223 | 1.806052 | 2.26E-50 | 7.18E-50 |
| RPL22P1 | 5.173811 | 3.472125 | -1.70169 | 2.51E-57 | 1.11E-56 |
| CCDC183 | 1.529767 | 0.455492 | -1.07427 | 4.15E-56 | 1.72E-55 |
| RPL23 | 8.248302 | 6.754819 | -1.49348 | 2.30E-56 | 9.64E-56 |
| ZNF677 | 1.898045 | 0.419304 | -1.47874 | 2.06E-73 | 5.47E-72 |
| PPP1R12C | 5.549277 | 3.379303 | -2.16997 | 1.92E-69 | 2.76E-68 |
| FAM47E-STBD1 | 1.465974 | 0.03074 | -1.43523 | 9.37E-73 | 2.23E-71 |
| POLDIP2 | 4.789009 | 5.798198 | 1.00919 | 2.90E-71 | 5.38E-70 |
| COQ6 | 3.176291 | 1.142004 | -2.03429 | 1.24E-74 | 4.07E-73 |
| GUCY2C | 0.393436 | 2.502097 | 2.108661 | 5.60E-59 | 2.77E-58 |
| LY96 | 2.057476 | 3.530255 | 1.472779 | 2.96E-46 | 8.24E-46 |
| HSPB1P2 | 0.420807 | 1.470559 | 1.049752 | 1.34E-55 | 5.38E-55 |
| KLK11 | 4.421854 | 2.105461 | -2.31639 | 9.80E-26 | 1.71E-25 |
| ZNF296 | 1.297324 | 2.636765 | 1.339441 | 3.15E-54 | 1.18E-53 |
| FXYD5 | 4.705089 | 5.813436 | 1.108347 | 8.56E-43 | 2.16E-42 |
| CDK10 | 4.864536 | 2.885659 | -1.97888 | 4.40E-54 | 1.63E-53 |
| EPHA2 | 2.819504 | 4.977249 | 2.157744 | 5.22E-56 | 2.14E-55 |
| RPL37P2 | 1.376755 | 5.646206 | 4.269451 | 2.77E-66 | 2.68E-65 |
| IRF2BP1 | 3.166598 | 4.234278 | 1.06768 | 3.61E-49 | 1.10E-48 |
| ASPH | 4.602961 | 3.510998 | -1.09196 | 4.64E-42 | 1.15E-41 |
| MT-RNR2 | 11.78373 | 13.39024 | 1.606517 | 2.43E-54 | 9.12E-54 |
| S100A14 | 4.118109 | 5.980575 | 1.862466 | 1.27E-23 | 2.13E-23 |
| ZNF185 | 1.647663 | 2.696591 | 1.048928 | 4.94E-33 | 1.00E-32 |
| IGFBP7 | 6.358769 | 8.043789 | 1.68502 | 2.87E-42 | 7.15E-42 |
| LINC02542 | 0.294184 | 1.345742 | 1.051558 | 5.28E-53 | 1.87E-52 |
| OAS3 | 1.959332 | 4.473263 | 2.513931 | 1.12E-73 | 3.14E-72 |
| PIK3AP1 | 0.982732 | 3.259391 | 2.276659 | 5.07E-79 | 6.40E-77 |
| NEDD1 | 1.709671 | 2.793418 | 1.083748 | 2.28E-76 | 1.04E-74 |
| CXorf40A | 3.050745 | 1.5822 | -1.46855 | 1.17E-72 | 2.74E-71 |
| ZBTB24 | 1.270119 | 2.303304 | 1.033186 | 2.22E-76 | 1.02E-74 |
| WSB1 | 5.106256 | 3.441331 | -1.66492 | 3.54E-55 | 1.39E-54 |
| OAS2 | 2.123387 | 4.144938 | 2.02155 | 1.14E-60 | 6.42E-60 |
| CDRT4 | 1.998337 | 0.192337 | -1.806 | 3.97E-63 | 2.79E-62 |
| ST7L | 2.286378 | 1.247256 | -1.03912 | 6.77E-61 | 3.88E-60 |
| CAPN8 | 4.576826 | 3.118661 | -1.45817 | 7.91E-14 | 1.10E-13 |
| NMU | 0.742902 | 2.683882 | 1.94098 | 5.29E-47 | 1.51E-46 |
| AC093162.2 | 0.684305 | 2.244874 | 1.560569 | 2.80E-70 | 4.54E-69 |
| PPT1 | 3.965493 | 5.368936 | 1.403442 | 3.74E-69 | 5.14E-68 |
| LYPD6B | 3.23365 | 1.790805 | -1.44285 | 9.12E-18 | 1.37E-17 |
| MYOF | 3.237637 | 4.428125 | 1.190489 | 9.51E-35 | 2.00E-34 |
| NAT9 | 3.667582 | 2.503547 | -1.16404 | 1.36E-48 | 4.05E-48 |
| OSBPL3 | 2.061974 | 3.128182 | 1.066208 | 1.72E-52 | 5.96E-52 |
| CDC25B | 3.723042 | 4.892223 | 1.169181 | 1.95E-38 | 4.43E-38 |
| AC016596.2 | 0.635734 | 4.121321 | 3.485587 | 7.04E-65 | 5.96E-64 |
| SNORD69 | 0.184359 | 1.703282 | 1.518923 | 1.15E-68 | 1.49E-67 |
| CES1 | 2.996014 | 1.77123 | -1.22478 | 1.43E-21 | 2.30E-21 |
| SMAD2 | 3.405658 | 1.637282 | -1.76838 | 7.50E-66 | 6.95E-65 |
| LY6H | 2.14786 | 0.363532 | -1.78433 | 1.15E-74 | 3.78E-73 |
| MCTS2P | 0.963663 | 2.021909 | 1.058246 | 3.43E-65 | 2.98E-64 |
| RPS15AP11 | 0.440039 | 1.680732 | 1.240694 | 1.38E-61 | 8.40E-61 |
| HBA1 | 3.562088 | 1.254002 | -2.30809 | 6.21E-46 | 1.71E-45 |
| RGN | 2.629216 | 0.996734 | -1.63248 | 3.78E-58 | 1.76E-57 |
| MAP4K3-DT | 2.815273 | 0.926284 | -1.88899 | 8.67E-76 | 3.47E-74 |
| CPA1 | 1.57554 | 0.149496 | -1.42604 | 5.66E-67 | 5.91E-66 |
| RBM15 | 1.412273 | 2.485131 | 1.072858 | 1.50E-75 | 5.73E-74 |
| BLOC1S5-TXNDC5 | 2.248625 | 0.090549 | -2.15808 | 8.46E-76 | 3.41E-74 |
| HIP1 | 1.766696 | 2.791818 | 1.025122 | 3.08E-58 | 1.44E-57 |
| SCGB2A1 | 2.005955 | 0.999615 | -1.00634 | 3.34E-13 | 4.60E-13 |
| IGKV1-39 | 1.591197 | 2.707561 | 1.116363 | 1.70E-16 | 2.49E-16 |
| ADAR | 4.509002 | 5.787268 | 1.278266 | 2.60E-81 | 7.05E-79 |
| PRAP1 | 0.780059 | 3.70711 | 2.927051 | 6.53E-52 | 2.21E-51 |
| SETD1A | 2.438366 | 3.447631 | 1.009265 | 4.94E-66 | 4.67E-65 |
| PSMB2 | 3.983745 | 5.336264 | 1.352519 | 2.31E-80 | 4.49E-78 |
| TSPAN13 | 3.855786 | 5.848667 | 1.992882 | 3.10E-61 | 1.82E-60 |
| PRKAG2 | 3.607172 | 2.17266 | -1.43451 | 1.38E-73 | 3.82E-72 |
| RPS2P46 | 2.702952 | 4.340752 | 1.6378 | 1.89E-62 | 1.24E-61 |
| AC009245.1 | 0.551992 | 1.705314 | 1.153322 | 1.08E-60 | 6.08E-60 |
| SCARNA7 | 0.800599 | 2.872823 | 2.072225 | 7.32E-58 | 3.34E-57 |
| ENY2 | 4.63026 | 3.157278 | -1.47298 | 8.38E-57 | 3.60E-56 |
| PABPC4 | 5.995715 | 4.892812 | -1.1029 | 1.69E-46 | 4.75E-46 |
| RNU5F-1 | 0.175359 | 1.230257 | 1.054897 | 7.59E-55 | 2.93E-54 |
| ZNF276 | 3.245015 | 1.881249 | -1.36377 | 1.77E-55 | 7.06E-55 |
| ACSM3 | 3.631086 | 1.421598 | -2.20949 | 1.12E-44 | 2.97E-44 |
| SLC26A9 | 3.121054 | 1.305553 | -1.8155 | 2.28E-22 | 3.74E-22 |
| ABCA6 | 2.008302 | 0.366571 | -1.64173 | 1.09E-72 | 2.57E-71 |
| ABHD2 | 3.741932 | 5.172889 | 1.430957 | 1.71E-43 | 4.41E-43 |
| MTMR3 | 3.066098 | 0.751586 | -2.31451 | 1.12E-74 | 3.73E-73 |
| IFITM9P | 0.135499 | 1.17815 | 1.042652 | 7.99E-75 | 2.75E-73 |
| COL4A1 | 4.057642 | 6.196536 | 2.138894 | 3.06E-56 | 1.27E-55 |
| AC008264.2 | 1.801336 | 0.052291 | -1.74905 | 4.29E-64 | 3.34E-63 |
| RPS9 | 8.619004 | 7.060531 | -1.55847 | 1.79E-63 | 1.31E-62 |
| GMIP | 2.549696 | 3.683901 | 1.134205 | 1.93E-51 | 6.41E-51 |
| ILF2 | 5.159924 | 6.31828 | 1.158357 | 9.83E-79 | 1.06E-76 |
| ST6GAL1 | 2.516448 | 3.694712 | 1.178264 | 1.19E-36 | 2.60E-36 |
| MIR4263 | 0.195764 | 1.481432 | 1.285668 | 1.61E-60 | 8.95E-60 |
| WDR59 | 3.689265 | 2.218157 | -1.47111 | 7.31E-61 | 4.17E-60 |
| ZNF444 | 3.759252 | 2.739465 | -1.01979 | 6.92E-53 | 2.43E-52 |
| PPP1R1A | 2.709081 | 0.61527 | -2.09381 | 2.63E-56 | 1.10E-55 |
| TMEM116 | 2.400701 | 0.878756 | -1.52195 | 1.08E-71 | 2.14E-70 |
| TOMM5 | 4.445683 | 2.436768 | -2.00892 | 3.49E-58 | 1.63E-57 |
| EIF2D | 3.915461 | 2.479973 | -1.43549 | 6.48E-73 | 1.58E-71 |
| PGAM5 | 2.757251 | 3.960865 | 1.203614 | 2.83E-71 | 5.27E-70 |
| DTL | 0.607187 | 2.554496 | 1.947309 | 1.57E-80 | 3.30E-78 |
| FBXW7 | 2.726321 | 1.692501 | -1.03382 | 3.77E-71 | 6.92E-70 |
| KCNK1 | 2.59614 | 3.751333 | 1.155193 | 1.21E-24 | 2.07E-24 |
| ILK | 5.539806 | 2.03965 | -3.50016 | 4.33E-76 | 1.85E-74 |
| ACTA2-AS1 | 2.801095 | 0.985777 | -1.81532 | 1.74E-50 | 5.55E-50 |
| TIPRL | 2.653512 | 3.982231 | 1.328718 | 2.50E-72 | 5.55E-71 |
| ADA | 3.984333 | 2.455755 | -1.52858 | 4.28E-38 | 9.65E-38 |
| ACAD11 | 2.481708 | 0.10471 | -2.377 | 6.47E-79 | 7.73E-77 |
| AC004890.2 | 2.334395 | 0.311032 | -2.02336 | 9.59E-60 | 5.03E-59 |
| ATP1B1 | 6.178076 | 7.519805 | 1.341729 | 5.29E-25 | 9.11E-25 |
| HEMK1 | 2.819795 | 0.819204 | -2.00059 | 8.45E-70 | 1.28E-68 |
| SPAG7 | 4.518136 | 3.307526 | -1.21061 | 2.74E-78 | 1.97E-76 |
| ITGB8 | 1.122611 | 2.248233 | 1.125622 | 4.62E-50 | 1.45E-49 |
| VPS28 | 6.029602 | 4.62702 | -1.40258 | 1.59E-67 | 1.77E-66 |
| ZNF226 | 2.563458 | 1.411399 | -1.15206 | 2.93E-60 | 1.60E-59 |
| NR2F2-AS1 | 1.733617 | 0.248104 | -1.48551 | 5.77E-74 | 1.70E-72 |
| AK1 | 4.475314 | 2.182869 | -2.29244 | 2.25E-66 | 2.21E-65 |
| AL139099.3 | 0.172732 | 1.282017 | 1.109286 | 1.76E-66 | 1.74E-65 |
| AC079922.1 | 0.539563 | 2.369299 | 1.829736 | 7.68E-70 | 1.17E-68 |
| HSP90AA1 | 6.571504 | 8.078208 | 1.506704 | 3.20E-66 | 3.07E-65 |
| CD36 | 3.631566 | 1.214835 | -2.41673 | 1.97E-74 | 6.21E-73 |
| EEF1A1P12 | 0.568814 | 1.998158 | 1.429344 | 1.43E-59 | 7.43E-59 |
| STAG3L5P-PVRIG2P-PILRB | 4.105201 | 0.603018 | -3.50218 | 2.42E-50 | 7.69E-50 |
| SYK | 2.074536 | 3.747256 | 1.672719 | 2.78E-59 | 1.41E-58 |
| PLXNB3 | 2.571608 | 1.043718 | -1.52789 | 5.76E-43 | 1.46E-42 |
| TMEM102 | 1.690451 | 2.864496 | 1.174045 | 1.26E-47 | 3.66E-47 |
| KIF5B | 4.050516 | 5.804297 | 1.753781 | 2.32E-74 | 7.21E-73 |
| UBE2C | 1.984862 | 5.667292 | 3.68243 | 8.69E-78 | 5.48E-76 |
| UHRF2 | 3.162642 | 2.152381 | -1.01026 | 7.99E-53 | 2.80E-52 |
| SELPLG | 1.95558 | 3.2993 | 1.34372 | 2.83E-45 | 7.65E-45 |
| XRN2 | 3.59739 | 5.259898 | 1.662508 | 5.62E-84 | 1.01E-80 |
| MARS2 | 1.406448 | 2.588603 | 1.182155 | 2.16E-69 | 3.08E-68 |
| CDCA5 | 1.039751 | 2.931148 | 1.891396 | 2.16E-70 | 3.56E-69 |
| SNHG29 | 7.040466 | 5.474004 | -1.56646 | 2.25E-57 | 1.00E-56 |
| NOTCH2NLA | 1.818345 | 0.125535 | -1.69281 | 2.80E-68 | 3.45E-67 |
| SPSB1 | 2.876455 | 3.902006 | 1.025551 | 5.82E-34 | 1.20E-33 |
| ARHGAP39 | 1.07501 | 2.504681 | 1.429671 | 8.11E-75 | 2.78E-73 |
| CLDN2 | 0.722922 | 3.466667 | 2.743746 | 3.43E-50 | 1.08E-49 |
| PLA2G2A | 4.180606 | 5.301066 | 1.12046 | 6.96E-05 | 8.01E-05 |
| VPS51 | 5.399269 | 4.315921 | -1.08335 | 1.99E-67 | 2.20E-66 |
| ABRAXAS2 | 2.132107 | 3.300583 | 1.168475 | 6.52E-69 | 8.71E-68 |
| PGA4 | 9.716168 | 0.086139 | -9.63003 | 5.47E-76 | 2.29E-74 |
| TMEM209 | 1.714837 | 2.926198 | 1.211361 | 7.15E-77 | 3.64E-75 |
| FDCSP | 0.665472 | 2.633855 | 1.968383 | 4.69E-33 | 9.49E-33 |
| EFNA2 | 0.334175 | 2.624554 | 2.290378 | 1.26E-65 | 1.15E-64 |
| MIR135A1 | 0.191454 | 1.528496 | 1.337043 | 1.95E-58 | 9.27E-58 |
| MIR7111 | 0.12187 | 1.123632 | 1.001762 | 3.91E-48 | 1.15E-47 |
| RPL15P3 | 2.561975 | 4.493922 | 1.931947 | 1.82E-60 | 1.01E-59 |
| TGFB1I1 | 4.585805 | 3.397437 | -1.18837 | 2.04E-24 | 3.47E-24 |
| BHLHE40 | 4.211417 | 5.677751 | 1.466334 | 2.71E-38 | 6.14E-38 |
| COX4I1 | 7.502157 | 5.922724 | -1.57943 | 4.08E-69 | 5.57E-68 |
| CENPW | 1.887467 | 3.900067 | 2.012599 | 1.61E-72 | 3.69E-71 |
| SNHG12 | 3.724748 | 2.134213 | -1.59053 | 1.31E-48 | 3.91E-48 |
| AC008147.3 | 0.214479 | 1.537607 | 1.323129 | 1.27E-71 | 2.49E-70 |
| MARCKSL1 | 4.64715 | 7.702878 | 3.055728 | 3.52E-83 | 3.01E-80 |
| CLASRP | 5.09466 | 2.98783 | -2.10683 | 7.42E-65 | 6.25E-64 |
| SMARCD3 | 4.02493 | 1.567708 | -2.45722 | 1.14E-73 | 3.18E-72 |
| HIST2H2BB | 1.968731 | 0.408135 | -1.5606 | 3.73E-54 | 1.39E-53 |
| GRIPAP1 | 3.875596 | 2.864169 | -1.01143 | 5.63E-55 | 2.19E-54 |
| FAM162A | 4.976396 | 3.850184 | -1.12621 | 1.37E-63 | 1.02E-62 |
| SH3GL2 | 1.760025 | 0.150259 | -1.60977 | 5.90E-77 | 3.07E-75 |
| UBALD2 | 4.556002 | 5.587436 | 1.031434 | 2.48E-43 | 6.35E-43 |
| RPL7P23 | 0.555312 | 1.852003 | 1.296691 | 4.15E-66 | 3.96E-65 |
| CKMT2 | 2.836273 | 0.549608 | -2.28666 | 1.11E-76 | 5.38E-75 |
| ADM | 3.842664 | 2.541994 | -1.30067 | 4.88E-38 | 1.10E-37 |
| FOXD2 | 0.284398 | 1.495194 | 1.210796 | 8.54E-75 | 2.93E-73 |
| KDM4C | 2.766494 | 1.327942 | -1.43855 | 3.74E-63 | 2.63E-62 |
| BORCS8 | 2.98744 | 1.617472 | -1.36997 | 1.10E-67 | 1.25E-66 |
| CAPRIN2 | 2.956573 | 1.710098 | -1.24648 | 1.70E-48 | 5.06E-48 |
| MRPL14 | 3.860451 | 5.427495 | 1.567044 | 3.95E-71 | 7.21E-70 |
| AL592114.1 | 0.41528 | 1.477521 | 1.062241 | 5.40E-62 | 3.39E-61 |
| TMEM250 | 3.185253 | 4.228994 | 1.043741 | 1.91E-55 | 7.61E-55 |
| ZC3HAV1L | 0.996038 | 2.628815 | 1.632777 | 1.45E-73 | 3.96E-72 |
| DAG1 | 3.962688 | 5.487357 | 1.52467 | 4.15E-70 | 6.57E-69 |
| FBXO17 | 2.363093 | 1.133768 | -1.22932 | 3.00E-46 | 8.33E-46 |
| ARL16 | 3.784738 | 2.756064 | -1.02867 | 2.30E-58 | 1.08E-57 |
| GPT | 3.009824 | 1.572777 | -1.43705 | 4.39E-28 | 8.02E-28 |
| ANAPC4 | 3.067516 | 2.001092 | -1.06642 | 1.13E-55 | 4.54E-55 |
| IL22RA1 | 1.08909 | 3.270781 | 2.181691 | 4.63E-55 | 1.81E-54 |
| TMX1 | 2.70637 | 3.883398 | 1.177028 | 7.13E-74 | 2.06E-72 |
| GOLGA2P10 | 3.360723 | 1.963009 | -1.39771 | 2.02E-32 | 4.03E-32 |
| RARRES2 | 5.642174 | 4.41521 | -1.22696 | 1.29E-32 | 2.60E-32 |
| CABLES2 | 1.613603 | 2.678326 | 1.064724 | 3.49E-64 | 2.74E-63 |
| PSD | 3.127754 | 1.119585 | -2.00817 | 3.92E-48 | 1.16E-47 |
| IGHG2 | 4.055841 | 6.918432 | 2.862591 | 4.13E-36 | 8.93E-36 |
| DNAJC7 | 4.289558 | 2.927934 | -1.36162 | 8.33E-51 | 2.70E-50 |
| FUZ | 2.980897 | 1.696194 | -1.2847 | 8.66E-57 | 3.72E-56 |
| KLK6 | 0.27956 | 2.727081 | 2.447521 | 1.56E-50 | 4.98E-50 |
| KPNA2 | 3.090779 | 5.459748 | 2.368969 | 1.78E-82 | 8.85E-80 |
| PPP1R12B | 3.991929 | 2.799106 | -1.19282 | 4.58E-26 | 8.05E-26 |
| RRM2B | 1.924359 | 3.053552 | 1.129193 | 6.72E-71 | 1.18E-69 |
| ARL6IP1 | 4.47702 | 6.217301 | 1.740281 | 2.99E-77 | 1.65E-75 |
| PDGFRB | 3.504587 | 4.670744 | 1.166157 | 1.66E-32 | 3.33E-32 |
| AC018738.1 | 1.243942 | 5.293748 | 4.049805 | 1.79E-57 | 7.97E-57 |
| PLAC9 | 3.212155 | 1.969112 | -1.24304 | 2.03E-43 | 5.21E-43 |
| MDH1 | 5.973677 | 4.407698 | -1.56598 | 7.81E-72 | 1.59E-70 |
| BRCA1 | 1.063792 | 2.089774 | 1.025982 | 2.62E-64 | 2.09E-63 |
| KLF5 | 3.555895 | 6.619219 | 3.063325 | 8.55E-67 | 8.80E-66 |
| ICAM3 | 2.458188 | 0.817146 | -1.64104 | 1.55E-57 | 6.95E-57 |
| VANGL1 | 1.375375 | 2.56292 | 1.187545 | 1.17E-71 | 2.30E-70 |
| ARHGEF18 | 3.058551 | 1.263555 | -1.795 | 9.73E-69 | 1.27E-67 |
| MFAP5 | 3.369342 | 2.293001 | -1.07634 | 1.24E-19 | 1.93E-19 |
| AL627309.5 | 1.525003 | 0.045062 | -1.47994 | 5.83E-70 | 9.01E-69 |
| IDO1 | 0.757727 | 2.583211 | 1.825483 | 2.80E-47 | 8.03E-47 |
| GINS1 | 0.684027 | 2.582249 | 1.898222 | 1.16E-78 | 1.21E-76 |
| C2CD4D-AS1 | 0.913561 | 1.921861 | 1.0083 | 1.34E-29 | 2.51E-29 |
| AMT | 3.701738 | 1.349027 | -2.35271 | 1.13E-76 | 5.47E-75 |
| ABO | 1.623689 | 2.668384 | 1.044696 | 2.60E-21 | 4.16E-21 |
| UFD1 | 4.045012 | 2.885391 | -1.15962 | 3.43E-58 | 1.60E-57 |
| MXRA5 | 1.607667 | 4.642569 | 3.034902 | 5.54E-77 | 2.90E-75 |
| ITGAV | 3.186683 | 4.434276 | 1.247593 | 3.80E-55 | 1.49E-54 |
| AC064799.1 | 1.079338 | 2.519313 | 1.439975 | 2.53E-55 | 1.00E-54 |
| ONECUT3 | 0.339539 | 1.54254 | 1.203002 | 2.23E-17 | 3.32E-17 |
| NEK6 | 2.699785 | 4.330201 | 1.630416 | 4.21E-70 | 6.65E-69 |
| CD55 | 3.644127 | 5.867644 | 2.223517 | 1.25E-51 | 4.19E-51 |
| RITA1 | 2.447312 | 3.621859 | 1.174547 | 9.84E-64 | 7.36E-63 |
| IZUMO4 | 2.199185 | 0.451635 | -1.74755 | 1.45E-73 | 3.96E-72 |
| YES1 | 2.785717 | 4.009527 | 1.223809 | 1.94E-65 | 1.73E-64 |
| RPS18P12 | 0.717832 | 3.276879 | 2.559047 | 4.34E-64 | 3.38E-63 |
| FAM110A | 1.947791 | 3.150602 | 1.202811 | 2.41E-52 | 8.30E-52 |
| CCL4L2 | 0.905193 | 2.203939 | 1.298746 | 2.46E-41 | 5.99E-41 |
| SMARCE1 | 4.324374 | 2.814631 | -1.50974 | 6.18E-56 | 2.52E-55 |
| KLHL23 | 1.671133 | 2.798548 | 1.127415 | 2.28E-39 | 5.30E-39 |
| RWDD3 | 2.799929 | 1.428915 | -1.37101 | 3.29E-58 | 1.54E-57 |
| CHPF | 4.157319 | 5.978832 | 1.821513 | 4.54E-71 | 8.17E-70 |
| ZBED1 | 3.031863 | 4.032892 | 1.001029 | 2.63E-61 | 1.56E-60 |
| FIP1L1 | 3.551654 | 2.395512 | -1.15614 | 1.27E-58 | 6.13E-58 |
| AC005912.1 | 3.176257 | 6.663444 | 3.487186 | 2.44E-69 | 3.47E-68 |
| C5orf15 | 3.420456 | 4.966164 | 1.545708 | 1.57E-69 | 2.29E-68 |
| PREP | 3.157428 | 4.256373 | 1.098945 | 4.73E-67 | 4.99E-66 |
| NRAV | 1.585097 | 2.691825 | 1.106727 | 1.05E-61 | 6.45E-61 |
| S100A10 | 6.266119 | 8.609165 | 2.343046 | 1.27E-69 | 1.86E-68 |
| UBE2B | 5.074253 | 3.988859 | -1.08539 | 1.42E-64 | 1.17E-63 |
| SPAST | 1.525897 | 2.812308 | 1.286411 | 8.42E-79 | 9.54E-77 |
| MIR590 | 0.202928 | 1.613405 | 1.410476 | 3.42E-62 | 2.19E-61 |
| MTCO2P12 | 2.132209 | 5.169974 | 3.037765 | 1.43E-61 | 8.65E-61 |
| SILC1 | 1.348643 | 0.064993 | -1.28365 | 4.66E-56 | 1.92E-55 |
| UNKL | 2.661306 | 1.409941 | -1.25137 | 1.27E-61 | 7.73E-61 |
| CNKSR3 | 2.195739 | 1.175775 | -1.01996 | 3.60E-56 | 1.49E-55 |
| AL136380.1 | 0.64936 | 2.850924 | 2.201564 | 6.30E-64 | 4.81E-63 |
| CNN1 | 6.27615 | 4.352661 | -1.92349 | 8.90E-21 | 1.41E-20 |
| OBSCN | 1.994946 | 0.735636 | -1.25931 | 9.19E-60 | 4.83E-59 |
| DNAJC17 | 3.144268 | 1.486576 | -1.65769 | 9.19E-71 | 1.60E-69 |
| SMC2 | 1.45424 | 3.113625 | 1.659385 | 1.28E-81 | 3.88E-79 |
| MELK | 0.890202 | 3.241887 | 2.351685 | 1.37E-77 | 8.20E-76 |
| PRPH | 1.525503 | 0.174722 | -1.35078 | 9.69E-67 | 9.92E-66 |
| C3orf18 | 2.53608 | 0.926917 | -1.60916 | 3.94E-72 | 8.42E-71 |
| SIK1B | 3.411991 | 1.593151 | -1.81884 | 1.22E-50 | 3.93E-50 |
| MVD | 4.025771 | 2.8734 | -1.15237 | 1.55E-52 | 5.36E-52 |
| FLNA | 8.263817 | 6.96487 | -1.29895 | 2.77E-16 | 4.04E-16 |
| GSK3B | 2.40533 | 3.641476 | 1.236146 | 4.62E-71 | 8.31E-70 |
| STAG3L3 | 2.769128 | 0.738729 | -2.0304 | 4.56E-53 | 1.61E-52 |
| REXO2 | 5.057041 | 3.478596 | -1.57845 | 2.20E-64 | 1.76E-63 |
| ERGIC1 | 5.307984 | 4.037295 | -1.27069 | 8.94E-67 | 9.18E-66 |
| C19orf33 | 4.47864 | 6.278902 | 1.800261 | 3.91E-28 | 7.13E-28 |
| AC018868.1 | 0.50397 | 2.226374 | 1.722404 | 1.06E-67 | 1.20E-66 |
| WDR61 | 3.916056 | 2.444833 | -1.47122 | 2.02E-62 | 1.32E-61 |
| LUM | 4.737224 | 7.11794 | 2.380716 | 1.67E-46 | 4.68E-46 |
| HOXC9 | 0.110959 | 1.432259 | 1.3213 | 2.47E-69 | 3.52E-68 |
| GMPPB | 3.317165 | 2.276555 | -1.04061 | 9.63E-49 | 2.90E-48 |
| DNM2 | 5.499321 | 4.474668 | -1.02465 | 4.65E-40 | 1.10E-39 |
| HACD2 | 2.657939 | 4.157159 | 1.49922 | 2.82E-69 | 3.97E-68 |
| CHRD | 2.605532 | 1.044718 | -1.56081 | 5.95E-60 | 3.17E-59 |
| CHPT1 | 4.523998 | 3.457965 | -1.06603 | 3.63E-54 | 1.35E-53 |
| DCTN2 | 5.70548 | 4.579085 | -1.12639 | 2.61E-77 | 1.46E-75 |
| DHRS7 | 5.201999 | 3.847276 | -1.35472 | 4.29E-63 | 3.00E-62 |
| TRIM15 | 0.399658 | 2.873719 | 2.474061 | 5.51E-71 | 9.80E-70 |
| IQGAP3 | 1.150703 | 3.446586 | 2.295883 | 1.06E-70 | 1.82E-69 |
| GSTM1 | 2.888239 | 1.537463 | -1.35078 | 1.42E-20 | 2.24E-20 |
| ADAMTS1 | 4.323542 | 2.846308 | -1.47723 | 1.28E-34 | 2.69E-34 |
| SFR1 | 1.603968 | 2.63554 | 1.031572 | 1.50E-67 | 1.67E-66 |
| ADIRF | 4.520262 | 0.002607 | -4.51765 | 1.93E-77 | 1.11E-75 |
| CGNL1 | 2.740726 | 1.214625 | -1.5261 | 7.60E-58 | 3.46E-57 |
| GNB3 | 1.483718 | 0.339482 | -1.14424 | 1.46E-65 | 1.32E-64 |
| RNU4-40P | 0.256202 | 1.396148 | 1.139946 | 1.27E-54 | 4.86E-54 |
| CDHR5 | 1.625518 | 4.214865 | 2.589346 | 1.98E-54 | 7.47E-54 |
| WDR73 | 2.798675 | 0.737304 | -2.06137 | 8.46E-56 | 3.43E-55 |
| AP003037.1 | 2.759828 | 0.029985 | -2.72984 | 6.22E-74 | 1.82E-72 |
| CAPN10 | 3.217214 | 1.850041 | -1.36717 | 1.08E-52 | 3.77E-52 |
| POMK | 0.748215 | 2.502341 | 1.754126 | 3.89E-72 | 8.31E-71 |
| LATS1 | 1.942335 | 2.965573 | 1.023239 | 1.34E-66 | 1.35E-65 |
| LINC01786 | 1.397142 | 0.234959 | -1.16218 | 7.29E-65 | 6.16E-64 |
| FOXM1 | 1.424548 | 3.977407 | 2.552859 | 2.66E-76 | 1.18E-74 |
| MTLN | 4.303824 | 2.880058 | -1.42377 | 5.24E-64 | 4.03E-63 |
| F2R | 1.794736 | 4.409174 | 2.614438 | 9.58E-83 | 6.80E-80 |
| NUFIP2 | 2.711262 | 3.801663 | 1.090401 | 3.80E-79 | 5.08E-77 |
| RPL18AP3 | 4.897731 | 6.377722 | 1.47999 | 5.17E-59 | 2.57E-58 |
| TRAC | 2.684299 | 4.009985 | 1.325685 | 7.27E-31 | 1.40E-30 |
| SERINC5 | 2.087224 | 3.617926 | 1.530703 | 9.28E-74 | 2.63E-72 |
| ZDHHC9 | 3.204451 | 4.678842 | 1.474391 | 1.03E-73 | 2.91E-72 |
| SDS | 0.682479 | 2.257074 | 1.574595 | 6.82E-59 | 3.36E-58 |
| AC091390.4 | 1.839868 | 0.643927 | -1.19594 | 2.29E-54 | 8.64E-54 |
| GPER1 | 3.436301 | 0.916754 | -2.51955 | 1.78E-70 | 2.96E-69 |
| MTND5P11 | 0.544723 | 1.844926 | 1.300202 | 2.78E-56 | 1.16E-55 |
| TYMS | 2.19274 | 4.024389 | 1.831649 | 1.44E-63 | 1.06E-62 |
| N4BP2L2 | 4.357809 | 2.504477 | -1.85333 | 2.02E-57 | 9.00E-57 |
| DERL2 | 4.122787 | 2.679689 | -1.4431 | 5.17E-64 | 3.99E-63 |
| CD52 | 3.194955 | 4.389008 | 1.194052 | 2.00E-24 | 3.41E-24 |
| PDE1B | 1.915937 | 0.588235 | -1.3277 | 7.21E-56 | 2.94E-55 |
| TMEM161B | 2.782392 | 1.371156 | -1.41124 | 1.55E-64 | 1.26E-63 |
| AC093525.6 | 1.460327 | 0.097778 | -1.36255 | 1.59E-64 | 1.29E-63 |
| TCF25 | 5.458817 | 3.902596 | -1.55622 | 3.82E-73 | 9.63E-72 |
| AL157935.1 | 1.792651 | 0.521488 | -1.27116 | 2.12E-39 | 4.94E-39 |
| UCP2 | 3.995647 | 5.256177 | 1.26053 | 2.71E-42 | 6.77E-42 |
| AC245060.2 | 0.538237 | 1.601548 | 1.063311 | 2.06E-63 | 1.49E-62 |
| HERC2P2 | 3.859321 | 1.904287 | -1.95503 | 8.71E-55 | 3.35E-54 |
| PDLIM2 | 3.449551 | 1.51501 | -1.93454 | 3.76E-69 | 5.16E-68 |
| C4orf46 | 1.129035 | 2.225902 | 1.096867 | 3.76E-76 | 1.62E-74 |
| SIGMAR1 | 4.478828 | 6.009721 | 1.530892 | 1.06E-71 | 2.09E-70 |
| VAMP7 | 3.032334 | 4.345923 | 1.313588 | 2.49E-73 | 6.50E-72 |
| BGN | 4.135551 | 7.670463 | 3.534911 | 6.12E-77 | 3.18E-75 |
| FOXP3 | 0.401079 | 1.87929 | 1.478212 | 2.76E-74 | 8.53E-73 |
| DTX3L | 2.552531 | 4.611233 | 2.058702 | 1.31E-83 | 1.56E-80 |
| OSGIN2 | 1.698577 | 2.819461 | 1.120885 | 1.37E-66 | 1.38E-65 |
| BAIAP3 | 1.765122 | 0.697177 | -1.06795 | 2.59E-51 | 8.55E-51 |
| CMPK2 | 1.421154 | 2.544691 | 1.123536 | 5.96E-42 | 1.48E-41 |
| ABCA9 | 1.722638 | 0.405556 | -1.31708 | 2.06E-61 | 1.24E-60 |
| CBLC | 2.535573 | 5.064739 | 2.529166 | 9.67E-64 | 7.24E-63 |
| FOXC1 | 1.039917 | 2.91154 | 1.871624 | 1.21E-62 | 8.03E-62 |
| HMMR | 0.838069 | 2.81065 | 1.972582 | 6.30E-73 | 1.54E-71 |
| DNASE1 | 3.059771 | 1.54917 | -1.5106 | 7.93E-45 | 2.12E-44 |
| HSPB1P1 | 0.739531 | 2.8981 | 2.158568 | 7.38E-60 | 3.90E-59 |
| SIDT2 | 4.993242 | 3.1328 | -1.86044 | 2.79E-72 | 6.16E-71 |
| PDZRN4 | 1.546821 | 0.512365 | -1.03446 | 1.60E-32 | 3.20E-32 |
| MMP3 | 1.256149 | 2.715901 | 1.459753 | 5.33E-14 | 7.46E-14 |
| ANK3 | 2.347448 | 1.128112 | -1.21934 | 1.11E-68 | 1.45E-67 |
| SCNN1G | 1.767018 | 0.252507 | -1.51451 | 2.21E-53 | 7.94E-53 |
| AS3MT | 1.333308 | 0.140993 | -1.19232 | 2.61E-76 | 1.16E-74 |
| AL049873.1 | 1.316187 | 2.839076 | 1.522889 | 3.65E-58 | 1.70E-57 |
| TROAP | 1.346845 | 2.808587 | 1.461741 | 5.42E-46 | 1.50E-45 |
| NDUFA10 | 5.354327 | 3.449623 | -1.9047 | 2.96E-70 | 4.78E-69 |
| CPS1 | 0.752516 | 2.219492 | 1.466977 | 1.03E-08 | 1.30E-08 |
| NDUFC2 | 5.47485 | 4.195005 | -1.27984 | 2.06E-55 | 8.17E-55 |
| GKN1 | 8.128938 | 2.766845 | -5.36209 | 2.73E-38 | 6.19E-38 |
| ITGB6 | 1.695445 | 2.741845 | 1.0464 | 1.60E-19 | 2.47E-19 |
| RPS6KB2 | 4.309955 | 3.281178 | -1.02878 | 3.24E-60 | 1.77E-59 |
| ECT2 | 1.710509 | 4.290137 | 2.579628 | 9.87E-83 | 6.80E-80 |
| ALB | 1.794955 | 0.530129 | -1.26483 | 2.33E-47 | 6.70E-47 |
| ZIC2 | 0.131767 | 1.166713 | 1.034946 | 1.10E-21 | 1.77E-21 |
| ANKLE2 | 3.580821 | 2.499603 | -1.08122 | 1.12E-45 | 3.08E-45 |
| VKORC1L1 | 2.386607 | 3.809339 | 1.422732 | 6.10E-78 | 4.02E-76 |
| BRD9 | 3.309538 | 2.273385 | -1.03615 | 4.26E-47 | 1.21E-46 |
| TFDP1 | 3.735392 | 5.149702 | 1.41431 | 1.78E-79 | 2.60E-77 |
| RNA5-8SN2 | 6.551884 | 0.295405 | -6.25648 | 6.90E-62 | 4.31E-61 |
| ISLR | 3.247484 | 4.635167 | 1.387683 | 2.89E-21 | 4.62E-21 |
| CYHR1 | 4.270562 | 2.619557 | -1.65101 | 1.13E-58 | 5.48E-58 |
| MYLK | 5.01173 | 3.166467 | -1.84526 | 3.27E-30 | 6.23E-30 |
| ARHGDIG | 2.992731 | 0.614577 | -2.37815 | 6.28E-66 | 5.86E-65 |
| WRAP73 | 2.979984 | 1.827446 | -1.15254 | 1.26E-54 | 4.80E-54 |
| TMEM273 | 2.187004 | 1.185496 | -1.00151 | 1.33E-50 | 4.28E-50 |
| RPS7P11 | 1.082704 | 2.537542 | 1.454838 | 2.97E-64 | 2.35E-63 |
| ITCH | 2.672069 | 3.748668 | 1.076598 | 7.49E-68 | 8.71E-67 |
| AC108488.1 | 2.393426 | 0.856774 | -1.53665 | 1.44E-64 | 1.18E-63 |
| CENPT | 4.319761 | 2.30392 | -2.01584 | 2.99E-64 | 2.37E-63 |
| AGAP6 | 2.934896 | 1.870838 | -1.06406 | 6.97E-47 | 1.98E-46 |
| NHEJ1 | 2.037914 | 0.544461 | -1.49345 | 8.09E-63 | 5.49E-62 |
| TTC17 | 4.274505 | 2.99711 | -1.2774 | 2.42E-50 | 7.69E-50 |
| MEST | 1.661006 | 3.613818 | 1.952811 | 1.57E-73 | 4.25E-72 |
| HAND2-AS1 | 2.316411 | 0.523766 | -1.79265 | 1.56E-50 | 4.98E-50 |
| UBE2G2 | 4.365974 | 3.214133 | -1.15184 | 9.06E-64 | 6.79E-63 |
| AC016700.2 | 0.289427 | 1.818495 | 1.529067 | 1.88E-63 | 1.36E-62 |
| C19orf71 | 1.18956 | 2.2559 | 1.06634 | 1.98E-54 | 7.47E-54 |
| BNIP3 | 3.466829 | 1.987911 | -1.47892 | 3.00E-54 | 1.12E-53 |
| PGAM1 | 5.544809 | 4.535568 | -1.00924 | 2.89E-62 | 1.86E-61 |
| MIR186 | 0.175091 | 1.426898 | 1.251807 | 3.89E-56 | 1.61E-55 |
| PODXL | 2.735569 | 4.155215 | 1.419646 | 6.30E-71 | 1.11E-69 |
| ATP6V0E2 | 3.665084 | 2.649435 | -1.01565 | 7.84E-41 | 1.88E-40 |
| RPL4P4 | 1.989257 | 3.658134 | 1.668877 | 2.30E-61 | 1.37E-60 |
| WDR13 | 5.431655 | 3.087368 | -2.34429 | 3.55E-78 | 2.45E-76 |
| IL2RB | 1.166663 | 2.770261 | 1.603598 | 8.81E-54 | 3.23E-53 |
| MED13 | 2.23133 | 3.355735 | 1.124405 | 2.05E-68 | 2.57E-67 |
| STK36 | 3.669025 | 2.241898 | -1.42713 | 7.84E-50 | 2.44E-49 |
| SLC26A10 | 1.925032 | 0.068493 | -1.85654 | 1.35E-76 | 6.32E-75 |
| KDELR1 | 5.744337 | 7.260945 | 1.516607 | 3.59E-81 | 9.20E-79 |
| NUP155 | 1.708089 | 2.997978 | 1.289889 | 8.79E-81 | 1.99E-78 |
| CU633904.1 | 1.71352 | 0.012987 | -1.70053 | 8.29E-72 | 1.68E-70 |
| DDHD2 | 3.213236 | 2.109759 | -1.10348 | 2.01E-60 | 1.11E-59 |
| C4A | 3.892486 | 1.571205 | -2.32128 | 7.43E-50 | 2.32E-49 |
| IL11RA | 3.412011 | 0.916904 | -2.49511 | 6.25E-79 | 7.57E-77 |
| GLE1 | 2.618751 | 3.886516 | 1.267764 | 8.11E-75 | 2.78E-73 |
| CEP112 | 1.702021 | 0.679091 | -1.02293 | 1.10E-57 | 4.96E-57 |
| ILKAP | 3.780179 | 2.339338 | -1.44084 | 1.37E-61 | 8.34E-61 |
| FUT3 | 1.769067 | 3.543391 | 1.774324 | 1.74E-27 | 3.14E-27 |
| TCF20 | 2.363535 | 3.707995 | 1.344461 | 6.90E-75 | 2.39E-73 |
| CNPY3 | 4.047178 | 5.448872 | 1.401693 | 5.30E-74 | 1.56E-72 |
| SH3BGRL2 | 3.531422 | 4.557348 | 1.025926 | 1.42E-31 | 2.78E-31 |
| ABCA8 | 2.955411 | 0.73652 | -2.21889 | 2.16E-70 | 3.56E-69 |
| MTO1 | 2.706243 | 1.507946 | -1.1983 | 4.87E-66 | 4.62E-65 |
| ZNF207 | 4.765495 | 3.721474 | -1.04402 | 1.05E-47 | 3.05E-47 |
| MRPL44 | 2.999458 | 4.006374 | 1.006916 | 1.65E-64 | 1.34E-63 |
| AC103702.2 | 0.199739 | 2.801902 | 2.602163 | 6.46E-74 | 1.88E-72 |
| PFKP | 3.521161 | 4.739221 | 1.218059 | 7.78E-46 | 2.14E-45 |
| CPT1B | 2.815446 | 0.557812 | -2.25763 | 2.93E-51 | 9.66E-51 |
| AL139100.1 | 0.386212 | 1.681728 | 1.295516 | 3.06E-60 | 1.67E-59 |
| YBX1P2 | 0.425144 | 1.626063 | 1.200918 | 8.89E-62 | 5.50E-61 |
| TXNP6 | 0.457848 | 1.511347 | 1.053499 | 1.39E-58 | 6.68E-58 |
| CDA | 1.474451 | 3.308289 | 1.833838 | 9.13E-48 | 2.66E-47 |
| STOML1 | 2.277714 | 1.000324 | -1.27739 | 3.02E-67 | 3.27E-66 |
| GATD3B | 3.863418 | 0.84671 | -3.01671 | 3.91E-70 | 6.21E-69 |
| VAV2 | 2.26886 | 3.830966 | 1.562107 | 7.06E-78 | 4.56E-76 |
| ACER3 | 1.531613 | 2.885884 | 1.354271 | 4.13E-72 | 8.77E-71 |
| EVI2B | 2.154378 | 3.628016 | 1.473638 | 7.70E-41 | 1.85E-40 |
| MTCO1P22 | 0.193845 | 1.329888 | 1.136044 | 3.97E-65 | 3.43E-64 |
| PURB | 2.391962 | 3.469236 | 1.077274 | 1.42E-65 | 1.29E-64 |
| RNU1-28P | 1.676805 | 0.198926 | -1.47788 | 1.07E-61 | 6.55E-61 |
| SRMS | 0.429702 | 1.812267 | 1.382564 | 1.13E-52 | 3.93E-52 |
| DUT | 4.695262 | 3.655499 | -1.03976 | 1.78E-50 | 5.70E-50 |
| KLHDC8A | 1.540234 | 0.480756 | -1.05948 | 1.12E-47 | 3.24E-47 |
| TSPOAP1 | 3.442098 | 0.738491 | -2.70361 | 8.15E-73 | 1.96E-71 |
| ACTG2 | 7.810161 | 5.212188 | -2.59797 | 1.83E-29 | 3.44E-29 |
| BBOX1-AS1 | 0.382216 | 1.673078 | 1.290862 | 2.42E-55 | 9.58E-55 |
| RPS10P3 | 0.398523 | 1.943512 | 1.544988 | 2.04E-58 | 9.64E-58 |
| MTCO2P22 | 0.351813 | 2.360691 | 2.008878 | 2.43E-65 | 2.15E-64 |
| PLEKHJ1 | 4.974916 | 3.278656 | -1.69626 | 2.55E-57 | 1.13E-56 |
| PPDPF | 7.391185 | 9.458393 | 2.067208 | 3.30E-71 | 6.09E-70 |
| CAPN13 | 3.202455 | 1.048302 | -2.15415 | 2.16E-27 | 3.88E-27 |
| MTRNR2L2 | 0.437244 | 2.463723 | 2.026479 | 8.79E-58 | 3.98E-57 |
| AC004475.1 | 3.496415 | 2.29948 | -1.19694 | 9.60E-69 | 1.26E-67 |
| GALNT5 | 1.143457 | 2.323214 | 1.179757 | 1.99E-25 | 3.46E-25 |
| RPS3AP47 | 0.648714 | 2.514691 | 1.865978 | 4.87E-58 | 2.25E-57 |
| LINC00261 | 3.278637 | 1.551467 | -1.72717 | 4.32E-17 | 6.41E-17 |
| SMIM5 | 2.03498 | 0.638764 | -1.39622 | 3.75E-67 | 4.01E-66 |
| RNF43 | 1.516949 | 2.958849 | 1.4419 | 3.47E-43 | 8.85E-43 |
| SH3YL1 | 4.327798 | 2.520285 | -1.80751 | 6.32E-40 | 1.49E-39 |
| C6orf222 | 1.119293 | 2.738937 | 1.619644 | 2.97E-37 | 6.58E-37 |
| PSMC6 | 4.416814 | 3.209014 | -1.2078 | 2.10E-54 | 7.92E-54 |
| AC091057.3 | 1.473825 | 0.085412 | -1.38841 | 2.89E-61 | 1.70E-60 |
| BX248409.1 | 0.213514 | 1.237399 | 1.023885 | 2.59E-63 | 1.86E-62 |
| LGALS9B | 2.269096 | 0.778048 | -1.49105 | 4.51E-21 | 7.19E-21 |
| PNCK | 2.153919 | 0.590734 | -1.56319 | 2.20E-38 | 5.00E-38 |
| RNU1-2 | 1.665385 | 0.169763 | -1.49562 | 1.29E-61 | 7.86E-61 |
| DMD | 2.716156 | 1.098463 | -1.61769 | 1.06E-57 | 4.80E-57 |
| PAICS | 2.979123 | 4.283205 | 1.304082 | 1.51E-75 | 5.74E-74 |
| RPS29P3 | 0.456451 | 2.10768 | 1.651229 | 4.89E-58 | 2.26E-57 |
| EEF1A1 | 10.7005 | 9.596574 | -1.10393 | 8.13E-47 | 2.30E-46 |
| NPR1 | 2.302064 | 1.258606 | -1.04346 | 4.32E-41 | 1.04E-40 |
| GBP3 | 2.588844 | 3.802221 | 1.213376 | 2.61E-31 | 5.09E-31 |
| AC093752.1 | 2.53786 | 1.094504 | -1.44336 | 5.77E-59 | 2.85E-58 |
| RPL18 | 8.756868 | 7.550967 | -1.2059 | 7.45E-55 | 2.88E-54 |
| TEX264 | 4.163149 | 3.152623 | -1.01053 | 8.73E-57 | 3.75E-56 |
| ADO | 2.058581 | 3.553751 | 1.49517 | 2.96E-75 | 1.10E-73 |
| BRD8 | 3.930461 | 2.82322 | -1.10724 | 2.83E-55 | 1.12E-54 |
| ATP6V1F | 5.639617 | 6.651181 | 1.011564 | 2.67E-66 | 2.60E-65 |
| RBPMS2 | 3.937731 | 2.343823 | -1.59391 | 7.81E-32 | 1.54E-31 |
| BARX1 | 4.960133 | 3.241917 | -1.71822 | 5.12E-26 | 8.98E-26 |
| AL162413.1 | 0.05953 | 1.544934 | 1.485404 | 7.21E-20 | 1.12E-19 |
| GOLGA6L9 | 1.95326 | 0.608525 | -1.34474 | 5.63E-58 | 2.59E-57 |
| SH3BGRL3 | 5.993774 | 7.789233 | 1.795459 | 1.43E-69 | 2.10E-68 |
| MTRNR2L9 | 0.30231 | 1.669152 | 1.366843 | 9.67E-59 | 4.69E-58 |
| RBM34 | 3.422577 | 0.672066 | -2.75051 | 2.81E-51 | 9.27E-51 |
| BHLHE41 | 1.814684 | 3.281398 | 1.466714 | 1.48E-42 | 3.73E-42 |
| EVA1C | 3.465261 | 1.977711 | -1.48755 | 7.44E-60 | 3.94E-59 |
| MTHFD1L | 1.688325 | 2.889537 | 1.201212 | 6.15E-60 | 3.28E-59 |
| ZNF367 | 0.751655 | 2.412259 | 1.660605 | 3.33E-82 | 1.37E-79 |
| PSMD9 | 3.912782 | 1.587332 | -2.32545 | 4.42E-68 | 5.32E-67 |
| YJEFN3 | 1.770847 | 0.649244 | -1.1216 | 1.86E-48 | 5.53E-48 |
| USP32P1 | 2.063359 | 0.124171 | -1.93919 | 3.83E-60 | 2.07E-59 |
| LRRC27 | 1.853635 | 0.67772 | -1.17592 | 2.67E-67 | 2.89E-66 |
| NECTIN2 | 4.892511 | 5.905531 | 1.01302 | 4.15E-44 | 1.09E-43 |
| OVOL1 | 0.911434 | 2.257766 | 1.346331 | 2.21E-41 | 5.38E-41 |
| RAB24 | 3.540536 | 1.890983 | -1.64955 | 7.65E-71 | 1.34E-69 |
| GOLGA6L5P | 2.223955 | 0.125132 | -2.09882 | 1.33E-67 | 1.49E-66 |
| TMEM25 | 2.867771 | 1.497865 | -1.36991 | 3.55E-65 | 3.08E-64 |
| AP001052.1 | 0.099805 | 1.450343 | 1.350538 | 2.00E-71 | 3.80E-70 |
| RN7SL368P | 0.321134 | 1.559088 | 1.237954 | 3.03E-62 | 1.94E-61 |
| CRYBG2 | 0.712531 | 1.958514 | 1.245983 | 9.72E-45 | 2.59E-44 |
| UBE2R2 | 3.519103 | 4.797115 | 1.278012 | 5.71E-61 | 3.28E-60 |
| RNA5-8SN1 | 6.530249 | 0.094466 | -6.43578 | 3.89E-70 | 6.20E-69 |
| ERICH1 | 2.744768 | 1.730458 | -1.01431 | 6.47E-53 | 2.28E-52 |
| TOMM7 | 7.149835 | 5.853408 | -1.29643 | 6.62E-63 | 4.54E-62 |
| E2F7 | 0.327127 | 1.405666 | 1.078539 | 5.03E-68 | 6.01E-67 |
| AC025857.2 | 0.938962 | 2.669791 | 1.730829 | 7.05E-73 | 1.71E-71 |
| PLA2G4B | 2.076656 | 0.100561 | -1.9761 | 1.20E-65 | 1.09E-64 |
| AC010976.2 | 1.786369 | 0.437163 | -1.34921 | 1.13E-57 | 5.10E-57 |
| CISD2 | 2.627424 | 3.722658 | 1.095233 | 2.57E-68 | 3.18E-67 |
| SPG7 | 4.466125 | 2.518745 | -1.94738 | 2.58E-59 | 1.32E-58 |
| AC026401.3 | 1.663711 | 3.049325 | 1.385614 | 3.92E-57 | 1.72E-56 |
| UTP14C | 2.004449 | 3.048907 | 1.044458 | 5.20E-69 | 7.00E-68 |
| FBLN1 | 6.081055 | 4.205344 | -1.87571 | 1.61E-36 | 3.51E-36 |
| ENTPD2 | 1.908934 | 3.152613 | 1.243679 | 5.67E-32 | 1.12E-31 |
| SGPP1 | 1.955711 | 2.957861 | 1.00215 | 2.53E-50 | 8.02E-50 |
| HLA-DQB2 | 0.80328 | 2.260181 | 1.456901 | 1.90E-37 | 4.22E-37 |
| TIMM21 | 3.345845 | 2.006197 | -1.33965 | 2.92E-65 | 2.56E-64 |
| TRIB3 | 1.799241 | 2.988979 | 1.189738 | 2.25E-26 | 3.98E-26 |
| TM2D1 | 3.647381 | 2.362615 | -1.28477 | 2.58E-63 | 1.85E-62 |
| TSC22D3 | 6.680944 | 4.040673 | -2.64027 | 1.91E-72 | 4.30E-71 |
| SLC12A4 | 3.607187 | 2.580286 | -1.0269 | 1.00E-46 | 2.84E-46 |
| PCDH17 | 0.81351 | 1.863721 | 1.050211 | 6.81E-60 | 3.61E-59 |
| PTMAP8 | 0.173725 | 1.189482 | 1.015758 | 2.88E-73 | 7.43E-72 |
| AC240565.2 | 1.456652 | 0.250456 | -1.2062 | 9.30E-63 | 6.26E-62 |
| SNHG5 | 6.67676 | 4.68469 | -1.99207 | 2.99E-56 | 1.25E-55 |
| AL049839.2 | 4.437432 | 0.21743 | -4.22 | 5.93E-64 | 4.53E-63 |
| KANK1 | 3.310814 | 2.174409 | -1.1364 | 6.85E-52 | 2.32E-51 |
| CDKN3 | 1.403355 | 3.251383 | 1.848028 | 6.80E-65 | 5.76E-64 |
| RPS15P5 | 0.245986 | 1.308875 | 1.06289 | 4.28E-62 | 2.71E-61 |
| BAIAP2L2 | 1.81745 | 4.727297 | 2.909847 | 1.84E-67 | 2.04E-66 |
| JMJD7 | 2.058966 | 0.196558 | -1.86241 | 1.09E-63 | 8.10E-63 |
| ARF6 | 3.794963 | 5.34748 | 1.552517 | 3.73E-65 | 3.23E-64 |
| AL121845.3 | 1.28287 | 0.19109 | -1.09178 | 6.00E-53 | 2.11E-52 |
| CHAF1A | 1.778186 | 2.890413 | 1.112227 | 1.17E-63 | 8.66E-63 |
| SLC27A5 | 2.114627 | 0.747861 | -1.36677 | 9.17E-61 | 5.21E-60 |
| UTP25 | 1.256983 | 2.26865 | 1.011666 | 3.61E-82 | 1.37E-79 |
| ELL3 | 1.682639 | 0.681874 | -1.00077 | 3.81E-54 | 1.42E-53 |
| VNN1 | 0.901177 | 3.101467 | 2.20029 | 1.69E-48 | 5.04E-48 |
| CD14 | 3.341788 | 4.985723 | 1.643935 | 1.75E-44 | 4.62E-44 |
| MTRNR2L1 | 0.306473 | 1.411942 | 1.105468 | 1.28E-57 | 5.76E-57 |
| AC239859.6 | 2.04512 | 1.029002 | -1.01612 | 7.90E-40 | 1.86E-39 |
| ALG13 | 3.55708 | 2.426305 | -1.13077 | 2.02E-62 | 1.32E-61 |
| MTG1 | 3.359671 | 1.523659 | -1.83601 | 3.19E-51 | 1.05E-50 |
| SERPINE1 | 2.785039 | 4.635648 | 1.850608 | 9.91E-38 | 2.21E-37 |
| SEC23B | 3.736599 | 4.763205 | 1.026606 | 7.35E-47 | 2.08E-46 |
| CENPU | 1.268908 | 2.746854 | 1.477946 | 6.20E-60 | 3.30E-59 |
| GSN | 7.149282 | 5.194709 | -1.95457 | 6.31E-66 | 5.89E-65 |
| AC112491.1 | 3.146 | 4.977116 | 1.831116 | 1.92E-39 | 4.48E-39 |
| LENG8 | 6.097677 | 4.509298 | -1.58838 | 1.70E-51 | 5.64E-51 |
| SCAND2P | 1.578903 | 0.418555 | -1.16035 | 8.00E-67 | 8.26E-66 |
| RPL18A | 8.945108 | 7.924346 | -1.02076 | 8.56E-54 | 3.14E-53 |
| AOX1 | 2.393962 | 0.842329 | -1.55163 | 2.03E-50 | 6.45E-50 |
| SNRPFP1 | 0.198785 | 1.364222 | 1.165437 | 1.58E-70 | 2.66E-69 |
| TTC7B | 3.123325 | 0.705892 | -2.41743 | 1.72E-68 | 2.19E-67 |
| FAM57A | 2.858398 | 3.99342 | 1.135022 | 5.96E-51 | 1.94E-50 |
| RPS27P29 | 0.3527 | 1.882732 | 1.530032 | 8.92E-66 | 8.24E-65 |
| CASQ2 | 2.155506 | 0.929107 | -1.2264 | 1.31E-32 | 2.64E-32 |
| PTTG1 | 2.039387 | 4.034337 | 1.99495 | 1.33E-57 | 5.98E-57 |
| IGHV3-49 | 2.378574 | 4.230307 | 1.851733 | 6.92E-21 | 1.10E-20 |
| MARVELD2 | 1.835333 | 3.094415 | 1.259082 | 5.74E-50 | 1.80E-49 |
| IGHV1-46 | 2.112298 | 4.431372 | 2.319074 | 2.48E-32 | 4.95E-32 |
| IGKV1OR2-108 | 0.712646 | 2.066612 | 1.353966 | 6.29E-48 | 1.84E-47 |
| DGLUCY | 4.381238 | 2.56121 | -1.82003 | 1.21E-75 | 4.72E-74 |
| CARNMT1 | 1.65029 | 2.697979 | 1.047689 | 1.46E-69 | 2.13E-68 |
| DHRS1 | 3.516355 | 2.201961 | -1.31439 | 3.19E-69 | 4.46E-68 |
| CCDC85C | 2.899072 | 1.756591 | -1.14248 | 3.68E-66 | 3.52E-65 |
| RPL3P2 | 0.630667 | 1.665229 | 1.034561 | 4.53E-50 | 1.42E-49 |
| SCHIP1 | 1.309958 | 0.160395 | -1.14956 | 1.07E-67 | 1.22E-66 |
| ASIC3 | 1.609413 | 0.483664 | -1.12575 | 7.21E-59 | 3.54E-58 |
| RBM39 | 6.279308 | 4.385847 | -1.89346 | 1.23E-50 | 3.96E-50 |
| OSBPL9 | 4.192197 | 3.128721 | -1.06348 | 1.90E-56 | 8.01E-56 |
| PRAG1 | 1.625742 | 3.731419 | 2.105678 | 6.22E-74 | 1.82E-72 |
| CD79A | 2.14588 | 3.544357 | 1.398477 | 7.82E-20 | 1.22E-19 |
| HENMT1 | 1.168831 | 2.458086 | 1.289255 | 6.88E-58 | 3.14E-57 |
| CBWD2 | 3.073825 | 1.24659 | -1.82724 | 2.16E-55 | 8.58E-55 |
| CLIC3 | 2.02411 | 3.108283 | 1.084172 | 2.73E-25 | 4.72E-25 |
| LIN54 | 1.248942 | 2.252887 | 1.003945 | 5.66E-71 | 1.00E-69 |
| P3H4 | 2.069539 | 3.4454 | 1.375861 | 6.22E-67 | 6.48E-66 |
| EEF1DP1 | 0.533397 | 1.742143 | 1.208746 | 1.07E-58 | 5.17E-58 |
| ARPIN-AP3S2 | 2.044746 | 0.107173 | -1.93757 | 2.77E-73 | 7.20E-72 |
| AC093840.1 | 0.215266 | 1.220572 | 1.005306 | 5.02E-63 | 3.47E-62 |
| AC026403.1 | 4.019326 | 5.482066 | 1.462741 | 4.99E-43 | 1.27E-42 |
| AC234031.1 | 2.633571 | 0.036002 | -2.59757 | 1.29E-65 | 1.18E-64 |
| LARGE1 | 2.962589 | 1.925627 | -1.03696 | 1.38E-64 | 1.13E-63 |
| AL358781.1 | 2.116323 | 0.002008 | -2.11432 | 1.78E-78 | 1.35E-76 |
| POMP | 4.64476 | 6.465229 | 1.820468 | 7.83E-80 | 1.25E-77 |
| AC127502.2 | 1.812729 | 0.687966 | -1.12476 | 6.41E-58 | 2.93E-57 |
| GGA1 | 4.064029 | 2.875448 | -1.18858 | 2.37E-61 | 1.41E-60 |
| TUBBP1 | 0.416569 | 1.464312 | 1.047743 | 6.31E-72 | 1.30E-70 |
| SH2B3 | 1.99229 | 3.569385 | 1.577095 | 6.42E-73 | 1.57E-71 |
| MROH6 | 2.053696 | 3.386969 | 1.333272 | 4.23E-36 | 9.14E-36 |
| ZC3HAV1 | 2.425359 | 3.65121 | 1.225852 | 2.68E-61 | 1.58E-60 |
| GAK | 4.951423 | 3.41397 | -1.53745 | 2.59E-61 | 1.53E-60 |
| TCF19 | 1.833453 | 3.199262 | 1.365809 | 4.54E-62 | 2.87E-61 |
| LINC00342 | 3.184227 | 1.726929 | -1.4573 | 4.00E-38 | 9.03E-38 |
| FRK | 1.193253 | 2.317338 | 1.124086 | 2.67E-47 | 7.65E-47 |
| DPH1 | 4.128153 | 2.202466 | -1.92569 | 1.33E-66 | 1.34E-65 |
| SMG1P5 | 1.554364 | 0.353932 | -1.20043 | 4.09E-63 | 2.87E-62 |
| IGHV5-51 | 3.480976 | 6.114816 | 2.633839 | 4.12E-29 | 7.68E-29 |
| MTERF4 | 3.405359 | 1.53503 | -1.87033 | 6.83E-74 | 1.98E-72 |
| SNORD17 | 0.966863 | 3.469206 | 2.502343 | 3.85E-61 | 2.25E-60 |
| RASA4B | 2.509199 | 0.037055 | -2.47214 | 1.25E-77 | 7.59E-76 |
| PCSK7 | 4.191061 | 1.922085 | -2.26898 | 3.68E-72 | 7.94E-71 |
| TPD52L1 | 3.972972 | 1.22383 | -2.74914 | 6.90E-67 | 7.16E-66 |
| RAD51 | 0.855802 | 2.254994 | 1.399191 | 3.66E-69 | 5.04E-68 |
| AC079140.2 | 1.144952 | 4.3907 | 3.245748 | 4.13E-61 | 2.41E-60 |
| GRB7 | 2.899727 | 4.09988 | 1.200153 | 1.23E-10 | 1.61E-10 |
| FHL1 | 6.418377 | 3.353638 | -3.06474 | 4.52E-58 | 2.09E-57 |
| BOLA2B | 3.661818 | 0.603622 | -3.0582 | 4.18E-59 | 2.09E-58 |
| MAGEA6 | 0.022403 | 1.298404 | 1.276001 | 1.03E-06 | 1.23E-06 |
| TMEM59 | 6.574067 | 5.028088 | -1.54598 | 3.68E-70 | 5.89E-69 |
| RNU1-3 | 1.642382 | 0.034697 | -1.60768 | 4.27E-71 | 7.75E-70 |
| CHRDL1 | 2.535949 | 1.423768 | -1.11218 | 1.57E-20 | 2.48E-20 |
| OLFML2B | 1.432542 | 3.559494 | 2.126951 | 9.96E-63 | 6.70E-62 |
| RPL6P27 | 3.061509 | 4.593177 | 1.531668 | 5.28E-59 | 2.62E-58 |
| CST2 | 0.241289 | 1.988741 | 1.747453 | 2.20E-64 | 1.76E-63 |
| SERP2 | 2.267858 | 0.789511 | -1.47835 | 2.42E-71 | 4.55E-70 |
| IGFLR1 | 2.144089 | 0.901863 | -1.24223 | 1.48E-51 | 4.94E-51 |
| CSNK1D | 5.218754 | 3.814398 | -1.40436 | 9.32E-61 | 5.29E-60 |
| ACBD4 | 3.038654 | 1.961306 | -1.07735 | 1.16E-70 | 1.99E-69 |
| SPRED2 | 2.70096 | 3.85297 | 1.15201 | 1.12E-61 | 6.82E-61 |
| NBPF1 | 2.600495 | 1.334583 | -1.26591 | 9.14E-64 | 6.85E-63 |
| APOL1 | 3.664314 | 5.410261 | 1.745947 | 6.35E-47 | 1.80E-46 |
| RHOV | 0.819616 | 1.88608 | 1.066464 | 1.48E-28 | 2.72E-28 |
| RAB22A | 1.993268 | 3.291264 | 1.297995 | 5.03E-76 | 2.11E-74 |
| CHIA | 4.052982 | 0.095341 | -3.95764 | 2.72E-62 | 1.75E-61 |
| AC024293.1 | 4.278855 | 5.417264 | 1.138409 | 5.31E-45 | 1.42E-44 |
| TACC2 | 3.921539 | 2.675522 | -1.24602 | 3.07E-56 | 1.28E-55 |
| GDI2 | 5.201605 | 6.213518 | 1.011913 | 3.47E-67 | 3.73E-66 |
| MIR6859-1 | 0.216781 | 1.552726 | 1.335945 | 2.05E-50 | 6.53E-50 |
| RPL36A | 7.759453 | 4.734819 | -3.02463 | 4.60E-61 | 2.67E-60 |
| TRPV6 | 1.742731 | 0.316778 | -1.42595 | 1.73E-49 | 5.33E-49 |
| AC103810.3 | 0.421463 | 3.3444 | 2.922938 | 2.30E-72 | 5.14E-71 |
| LIPF | 9.480437 | 2.724897 | -6.75554 | 3.93E-46 | 1.09E-45 |
| TMEM258 | 5.751683 | 4.687981 | -1.0637 | 3.38E-54 | 1.26E-53 |
| IGKV3D-15 | 0.973836 | 2.033261 | 1.059425 | 4.49E-27 | 8.02E-27 |
| AC020898.1 | 0.647277 | 1.915668 | 1.26839 | 3.57E-64 | 2.80E-63 |
| ST5 | 4.899028 | 2.633437 | -2.26559 | 6.60E-76 | 2.71E-74 |
| CCDC130 | 4.329922 | 3.086947 | -1.24297 | 8.63E-64 | 6.49E-63 |
| PLK4 | 0.846818 | 2.08185 | 1.235032 | 4.00E-64 | 3.12E-63 |
| VSIG1 | 4.106439 | 3.077701 | -1.02874 | 5.01E-06 | 5.93E-06 |
| MRPL27 | 4.869043 | 3.654413 | -1.21463 | 5.10E-55 | 1.99E-54 |
| NCLN | 3.830768 | 5.099006 | 1.268238 | 9.74E-70 | 1.46E-68 |
| PDE9A | 2.512588 | 1.081925 | -1.43066 | 1.23E-52 | 4.27E-52 |
| NBN | 2.447025 | 3.752101 | 1.305076 | 1.82E-71 | 3.46E-70 |
| SNHG9 | 3.995881 | 2.688782 | -1.3071 | 1.74E-47 | 5.02E-47 |
| TWSG1 | 2.733933 | 3.983419 | 1.249486 | 2.45E-46 | 6.85E-46 |
| FENDRR | 3.514282 | 1.62135 | -1.89293 | 2.07E-57 | 9.23E-57 |
| SPOCK2 | 1.929923 | 3.009816 | 1.079893 | 1.15E-27 | 2.08E-27 |
| IGKV1-16 | 3.064542 | 4.532415 | 1.467873 | 3.58E-12 | 4.84E-12 |
| WDR6 | 5.475818 | 4.161223 | -1.3146 | 1.98E-60 | 1.09E-59 |
| PTK6 | 2.477406 | 3.777613 | 1.300207 | 2.21E-27 | 3.97E-27 |
| TIMELESS | 1.905293 | 3.111473 | 1.20618 | 3.87E-62 | 2.47E-61 |
| LYRM4 | 2.824893 | 1.643512 | -1.18138 | 4.33E-62 | 2.74E-61 |
| TRIB1 | 3.782071 | 4.890774 | 1.108703 | 1.54E-34 | 3.23E-34 |
| LEPR | 2.396051 | 1.068354 | -1.3277 | 1.38E-52 | 4.79E-52 |
| MAN2C1 | 5.64612 | 2.37188 | -3.27424 | 1.25E-68 | 1.61E-67 |
| SLC25A29 | 3.589368 | 2.434151 | -1.15522 | 4.61E-48 | 1.36E-47 |
| C9orf64 | 2.129882 | 3.142389 | 1.012507 | 8.14E-61 | 4.63E-60 |
| AGMAT | 0.642206 | 3.362594 | 2.720388 | 1.55E-73 | 4.21E-72 |
| XBP1 | 5.458915 | 0.002008 | -5.45691 | 1.78E-78 | 1.35E-76 |
| MTHFS | 3.210649 | 1.818278 | -1.39237 | 3.73E-56 | 1.55E-55 |
| MYO19 | 3.676092 | 2.453547 | -1.22255 | 6.65E-42 | 1.64E-41 |
| CCDC189 | 1.435856 | 0.349392 | -1.08646 | 1.30E-58 | 6.24E-58 |
| HIST2H4A | 1.597802 | 0.256323 | -1.34148 | 3.68E-58 | 1.71E-57 |
| KIF23 | 1.202991 | 2.494639 | 1.291648 | 4.29E-57 | 1.88E-56 |
| RPL10P8 | 0.180273 | 1.186019 | 1.005746 | 7.47E-66 | 6.93E-65 |
| SAMD5 | 1.092821 | 2.560982 | 1.468161 | 3.41E-43 | 8.69E-43 |
| RFXANK | 4.545317 | 3.476389 | -1.06893 | 1.67E-61 | 1.01E-60 |
| AC008026.1 | 0.843328 | 1.912215 | 1.068888 | 4.13E-53 | 1.46E-52 |
| RNF128 | 2.620034 | 4.125578 | 1.505543 | 1.36E-34 | 2.85E-34 |
| SUZ12 | 2.435537 | 3.439018 | 1.00348 | 6.47E-79 | 7.73E-77 |
| HOXA10 | 0.22133 | 2.012009 | 1.790679 | 5.58E-51 | 1.82E-50 |
| ICMT | 3.202629 | 4.697332 | 1.494704 | 5.12E-72 | 1.06E-70 |
| ECI2 | 4.983859 | 2.747645 | -2.23621 | 2.78E-71 | 5.18E-70 |
| RPS7 | 7.907458 | 6.421967 | -1.48549 | 2.55E-57 | 1.13E-56 |
| OAS1 | 3.017542 | 4.347027 | 1.329485 | 1.77E-29 | 3.31E-29 |
| MUC2 | 0.426997 | 1.932573 | 1.505576 | 1.85E-14 | 2.62E-14 |
| CFC1 | 1.524706 | 0.049445 | -1.47526 | 2.57E-58 | 1.21E-57 |
| STT3B | 4.157851 | 5.319807 | 1.161955 | 6.70E-74 | 1.95E-72 |
| SRSF5 | 6.835764 | 4.496522 | -2.33924 | 9.36E-74 | 2.65E-72 |
| FCGR2C | 1.666984 | 0.647556 | -1.01943 | 1.92E-42 | 4.80E-42 |
| FAM13A-AS1 | 1.590253 | 0.39189 | -1.19836 | 2.77E-62 | 1.78E-61 |
| C1QA | 4.646016 | 6.166472 | 1.520456 | 6.11E-33 | 1.23E-32 |
| EPHB4 | 3.412929 | 4.418078 | 1.005149 | 8.91E-54 | 3.26E-53 |
| RPS4Y1 | 4.301398 | 3.126252 | -1.17515 | 2.32E-14 | 3.27E-14 |
| AL583722.2 | 3.130787 | 0.002008 | -3.12878 | 1.78E-78 | 1.35E-76 |
| ANAPC2 | 4.135741 | 3.121752 | -1.01399 | 5.38E-56 | 2.20E-55 |
| C7 | 4.924189 | 2.260102 | -2.66409 | 1.09E-40 | 2.62E-40 |
| PRKCI | 3.110915 | 4.266759 | 1.155844 | 1.07E-54 | 4.12E-54 |
| CNNM4 | 2.110594 | 3.613488 | 1.502894 | 4.83E-63 | 3.35E-62 |
| CAMTA1 | 4.394629 | 2.189216 | -2.20541 | 1.13E-70 | 1.92E-69 |
| U2AF1 | 3.858528 | 0.194259 | -3.66427 | 7.73E-61 | 4.41E-60 |
| INTS5 | 2.509704 | 3.595532 | 1.085828 | 4.00E-64 | 3.12E-63 |
| PRDM1 | 1.88556 | 3.19549 | 1.309931 | 1.18E-41 | 2.89E-41 |
| AC006449.7 | 3.222695 | 0.002008 | -3.22069 | 1.78E-78 | 1.35E-76 |
| AC015922.2 | 1.25101 | 2.416565 | 1.165555 | 5.16E-33 | 1.04E-32 |
| CTRB2 | 1.443957 | 0.091451 | -1.35251 | 2.34E-59 | 1.19E-58 |
| PODXL2 | 2.654506 | 4.152258 | 1.497752 | 9.12E-39 | 2.09E-38 |
| FMC1 | 3.422791 | 2.349716 | -1.07308 | 1.02E-56 | 4.37E-56 |
| FEZF1-AS1 | 0.068153 | 1.188622 | 1.12047 | 2.97E-54 | 1.11E-53 |
| TIMP3 | 5.056722 | 2.198118 | -2.8586 | 8.86E-67 | 9.11E-66 |
| SSBP2 | 2.941308 | 1.094656 | -1.84665 | 4.75E-71 | 8.51E-70 |
| DBNL | 4.521746 | 3.432456 | -1.08929 | 5.04E-67 | 5.28E-66 |
| PPP1R9B | 3.367492 | 4.55383 | 1.186338 | 9.96E-73 | 2.36E-71 |
| FTH1P4 | 0.295021 | 1.449078 | 1.154057 | 1.92E-68 | 2.42E-67 |
| LDB3 | 1.958022 | 0.733544 | -1.22448 | 3.07E-45 | 8.28E-45 |
| SDC4 | 5.480296 | 6.99715 | 1.516854 | 7.19E-57 | 3.11E-56 |
| PRR13 | 6.276376 | 5.052823 | -1.22355 | 4.36E-51 | 1.43E-50 |
| IGSF6 | 0.819301 | 2.344061 | 1.524761 | 1.04E-69 | 1.54E-68 |
| SMS | 4.020506 | 5.44931 | 1.428804 | 2.82E-78 | 2.01E-76 |
| ITGB4 | 4.86978 | 6.263511 | 1.39373 | 1.86E-40 | 4.43E-40 |
| METTL3 | 3.794724 | 2.390596 | -1.40413 | 4.41E-55 | 1.72E-54 |
| AC006011.1 | 1.541689 | 0.002008 | -1.53968 | 1.78E-78 | 1.35E-76 |
| GNS | 3.825122 | 5.136681 | 1.311559 | 4.29E-70 | 6.76E-69 |
| LINC01006 | 2.432433 | 1.362611 | -1.06982 | 6.72E-39 | 1.54E-38 |
| SLIRP | 5.494228 | 4.058567 | -1.43566 | 1.20E-59 | 6.27E-59 |
| RNU6-1280P | 0.149589 | 1.288225 | 1.138635 | 1.61E-54 | 6.10E-54 |
| LAMA2 | 3.021774 | 1.63427 | -1.3875 | 5.55E-47 | 1.58E-46 |
| YWHAZP3 | 0.59025 | 1.713211 | 1.122961 | 1.00E-63 | 7.48E-63 |
| MTDH | 3.478409 | 5.475115 | 1.996706 | 1.29E-82 | 8.57E-80 |
| AC244154.1 | 1.944576 | 0.66458 | -1.28 | 1.50E-60 | 8.35E-60 |
| CRKL | 3.012046 | 4.279334 | 1.267288 | 1.08E-69 | 1.60E-68 |
| LTC4S | 1.44893 | 0.08029 | -1.36864 | 6.71E-81 | 1.58E-78 |
| NUSAP1 | 1.678321 | 4.273137 | 2.594816 | 8.96E-76 | 3.57E-74 |
| PPAN | 3.192001 | 1.670762 | -1.52124 | 2.16E-49 | 6.64E-49 |
| KLK1 | 1.032869 | 2.340673 | 1.307805 | 3.87E-22 | 6.31E-22 |
| MYOT | 1.325489 | 0.262146 | -1.06334 | 2.25E-71 | 4.24E-70 |
| THY1 | 3.165983 | 4.316373 | 1.15039 | 1.54E-24 | 2.63E-24 |
| TRBJ2-3 | 0.349819 | 1.635361 | 1.285542 | 1.63E-42 | 4.10E-42 |
| AC018521.5 | 1.928051 | 0.669678 | -1.25837 | 6.32E-76 | 2.61E-74 |
| GPRASP1 | 1.986439 | 0.883959 | -1.10248 | 1.51E-44 | 3.99E-44 |
| PRPSAP1 | 3.894842 | 2.755211 | -1.13963 | 1.91E-55 | 7.61E-55 |
| HSPE1P2 | 0.433479 | 1.494646 | 1.061168 | 3.70E-66 | 3.53E-65 |
| GBF1 | 3.47802 | 4.550387 | 1.072367 | 5.21E-62 | 3.28E-61 |
| CDH17 | 0.652913 | 4.58758 | 3.934666 | 7.20E-62 | 4.49E-61 |
| DPP4 | 1.075457 | 2.789615 | 1.714158 | 4.64E-45 | 1.25E-44 |
| HOXB7 | 1.696719 | 3.654021 | 1.957302 | 3.08E-51 | 1.02E-50 |
| TFF2 | 7.275631 | 4.93777 | -2.33786 | 2.97E-13 | 4.10E-13 |
| HNRNPUL2-BSCL2 | 2.797354 | 0.958111 | -1.83924 | 4.06E-66 | 3.87E-65 |
| CCDC57 | 3.723105 | 1.612857 | -2.11025 | 3.26E-54 | 1.22E-53 |
| ZNRF2P1 | 0.822973 | 3.014567 | 2.191594 | 6.42E-60 | 3.41E-59 |
| PHYHD1 | 3.962938 | 1.335996 | -2.62694 | 1.21E-75 | 4.72E-74 |
| LCP1 | 2.754016 | 4.564195 | 1.810179 | 5.24E-53 | 1.85E-52 |
| AFAP1-AS1 | 0.311351 | 1.869271 | 1.55792 | 8.20E-15 | 1.16E-14 |
| MET | 1.725912 | 4.23557 | 2.509658 | 8.65E-80 | 1.37E-77 |
| AL353691.2 | 0.319516 | 1.559701 | 1.240185 | 5.89E-51 | 1.92E-50 |
| PLBD2 | 2.883924 | 4.656584 | 1.77266 | 7.05E-78 | 4.56E-76 |
| ZNF385A | 2.732907 | 3.750335 | 1.017428 | 1.06E-34 | 2.22E-34 |
| C5orf66-AS1 | 1.963064 | 0.653864 | -1.3092 | 2.00E-53 | 7.21E-53 |
| SNORA66 | 0.383549 | 1.476636 | 1.093087 | 7.75E-49 | 2.34E-48 |
| TM2D3 | 3.602523 | 1.817234 | -1.78529 | 6.90E-62 | 4.31E-61 |
| THAP3 | 2.878005 | 1.588158 | -1.28985 | 2.62E-70 | 4.27E-69 |
| PI4KAP1 | 2.996631 | 0.911768 | -2.08486 | 3.17E-58 | 1.48E-57 |
| IGHV4-59 | 3.384078 | 5.254581 | 1.870503 | 3.69E-17 | 5.48E-17 |
| MIR429 | 0.278354 | 2.599845 | 2.321491 | 7.46E-68 | 8.68E-67 |
| AC124067.4 | 0.107103 | 1.293146 | 1.186043 | 2.25E-49 | 6.92E-49 |
| CHFR | 2.995993 | 1.149897 | -1.8461 | 1.25E-70 | 2.13E-69 |
| SERPINB9 | 1.621736 | 3.296475 | 1.67474 | 1.86E-62 | 1.22E-61 |
| AC087343.1 | 0.444877 | 1.610561 | 1.165684 | 1.08E-59 | 5.66E-59 |
| SEC16B | 1.75652 | 0.119166 | -1.63735 | 4.85E-66 | 4.60E-65 |
| BOLA3 | 3.801788 | 2.681949 | -1.11984 | 9.36E-53 | 3.27E-52 |
| AC016542.2 | 0.208874 | 1.263209 | 1.054335 | 4.26E-62 | 2.70E-61 |
| SELENOM | 6.021929 | 4.239501 | -1.78243 | 2.77E-45 | 7.48E-45 |
| PLSCR3 | 3.037094 | 0.37583 | -2.66126 | 1.78E-62 | 1.17E-61 |
| ATP11A | 2.011261 | 3.705544 | 1.694283 | 1.73E-78 | 1.35E-76 |
| DCXR | 6.088523 | 4.903107 | -1.18542 | 3.40E-40 | 8.06E-40 |
| ZNF775 | 3.180994 | 1.926663 | -1.25433 | 1.48E-60 | 8.25E-60 |
| SIAH2 | 3.260376 | 4.265251 | 1.004875 | 4.15E-58 | 1.93E-57 |
| FAM107A | 3.568952 | 1.115368 | -2.45358 | 1.32E-73 | 3.66E-72 |
| RBM6 | 4.997125 | 3.074076 | -1.92305 | 6.01E-61 | 3.45E-60 |
| AC004466.1 | 1.292637 | 0.145772 | -1.14686 | 7.86E-60 | 4.15E-59 |
| UFL1 | 2.783408 | 3.876028 | 1.092619 | 1.43E-61 | 8.68E-61 |
| LAMP3 | 0.51006 | 2.490664 | 1.980605 | 1.22E-75 | 4.74E-74 |
| EEF1A1P9 | 0.585831 | 2.143799 | 1.557968 | 3.49E-60 | 1.89E-59 |
| AL009174.1 | 1.495853 | 2.967721 | 1.471868 | 1.76E-56 | 7.42E-56 |
| IL33 | 3.725249 | 2.600383 | -1.12487 | 5.65E-22 | 9.17E-22 |
| TFB2M | 2.399298 | 3.547986 | 1.148688 | 1.64E-72 | 3.75E-71 |
| NDUFAF5 | 2.854081 | 1.362591 | -1.49149 | 5.94E-59 | 2.93E-58 |
| MT1E | 6.952016 | 5.176887 | -1.77513 | 2.03E-36 | 4.42E-36 |
| BATF2 | 1.660365 | 2.928357 | 1.267992 | 3.75E-43 | 9.57E-43 |
| GAS6 | 5.247303 | 4.148562 | -1.09874 | 6.23E-43 | 1.58E-42 |
| SKP2 | 1.555144 | 3.341265 | 1.786121 | 3.47E-79 | 4.70E-77 |
| GUCA2B | 1.967167 | 0.520708 | -1.44646 | 4.95E-24 | 8.36E-24 |
| AP001453.2 | 0.793275 | 1.854884 | 1.061609 | 1.59E-40 | 3.79E-40 |
| HEXD | 4.115279 | 2.454052 | -1.66123 | 6.30E-57 | 2.73E-56 |
| RF01955 | 1.612451 | 0.002008 | -1.61044 | 1.89E-78 | 1.40E-76 |
| RPS6KA3 | 2.465067 | 3.745903 | 1.280836 | 1.28E-66 | 1.29E-65 |
| CTSZ | 5.267662 | 6.609724 | 1.342062 | 9.66E-63 | 6.50E-62 |
| FAM222B | 2.382874 | 3.87954 | 1.496666 | 5.91E-70 | 9.10E-69 |
| ZNF692 | 4.13993 | 2.46663 | -1.6733 | 3.43E-50 | 1.08E-49 |
| RNU6-611P | 0.307926 | 2.138704 | 1.830778 | 8.67E-64 | 6.51E-63 |
| THNSL2 | 2.869045 | 1.260695 | -1.60835 | 5.93E-56 | 2.43E-55 |
| WASL | 3.288496 | 4.687991 | 1.399495 | 6.02E-59 | 2.97E-58 |
| GPX1P1 | 1.366564 | 2.680957 | 1.314393 | 2.82E-29 | 5.27E-29 |
| RPL10P6 | 1.363093 | 3.977728 | 2.614635 | 4.40E-56 | 1.81E-55 |
| LAPTM4B | 3.90179 | 6.021341 | 2.119551 | 2.90E-68 | 3.57E-67 |
| SLC25A26 | 2.955041 | 1.73306 | -1.22198 | 6.96E-67 | 7.22E-66 |
| FUT6 | 0.496424 | 1.924506 | 1.428082 | 3.18E-31 | 6.18E-31 |
| PI4KAP2 | 3.060485 | 0.90551 | -2.15498 | 1.23E-59 | 6.40E-59 |
| SHC2 | 3.861437 | 1.987938 | -1.8735 | 3.62E-63 | 2.55E-62 |
| NOP14 | 2.951507 | 4.012247 | 1.06074 | 4.11E-73 | 1.03E-71 |
| ALPG | 0.034145 | 1.274717 | 1.240573 | 1.95E-62 | 1.27E-61 |
| U2AF1L4 | 3.716765 | 1.674136 | -2.04263 | 1.66E-73 | 4.45E-72 |
| PRPF6 | 4.129016 | 5.415268 | 1.286253 | 6.07E-75 | 2.12E-73 |
| AC020913.3 | 0.137128 | 1.295483 | 1.158356 | 3.67E-68 | 4.47E-67 |
| TTC6 | 1.437661 | 0.256875 | -1.18079 | 2.37E-25 | 4.12E-25 |
| RAPGEF3 | 3.31017 | 0.890792 | -2.41938 | 1.71E-74 | 5.45E-73 |
| TTLL7 | 2.0335 | 0.837051 | -1.19645 | 1.12E-53 | 4.07E-53 |
| TCEAL2 | 2.821974 | 0.945209 | -1.87676 | 2.35E-45 | 6.36E-45 |
| MRPS23 | 3.687028 | 2.582987 | -1.10404 | 1.59E-48 | 4.74E-48 |
| KCNJ16 | 2.194828 | 0.264981 | -1.92985 | 1.38E-54 | 5.26E-54 |
| SNORA5C | 0.395227 | 1.720636 | 1.325409 | 1.34E-53 | 4.85E-53 |
| CCNB2 | 1.335378 | 3.785305 | 2.449926 | 4.11E-74 | 1.23E-72 |
| CXXC1 | 4.887545 | 3.206889 | -1.68066 | 6.63E-68 | 7.77E-67 |
| RAC1 | 5.923975 | 7.533021 | 1.609046 | 1.39E-78 | 1.35E-76 |
| YPEL3 | 5.31656 | 3.973237 | -1.34332 | 5.46E-67 | 5.71E-66 |
| NARF | 4.108139 | 2.418994 | -1.68914 | 3.85E-61 | 2.25E-60 |
| SH2B1 | 4.315883 | 2.667769 | -1.64811 | 3.75E-72 | 8.05E-71 |
| PLEKHS1 | 0.606286 | 2.837049 | 2.230763 | 2.73E-57 | 1.20E-56 |
| AC021074.1 | 0.364889 | 1.780651 | 1.415762 | 1.93E-65 | 1.72E-64 |
| IL13RA1 | 3.776527 | 5.253062 | 1.476535 | 9.35E-60 | 4.91E-59 |
| C17orf49 | 3.560602 | 0.587411 | -2.97319 | 2.99E-66 | 2.89E-65 |
| UBXN1 | 5.675657 | 4.655756 | -1.0199 | 4.55E-70 | 7.15E-69 |
| CCDC9B | 3.17182 | 1.507435 | -1.66439 | 6.43E-49 | 1.95E-48 |
| PDE4C | 2.671611 | 0.898429 | -1.77318 | 1.65E-59 | 8.54E-59 |
| TMEM220 | 2.705732 | 1.385265 | -1.32047 | 2.13E-67 | 2.33E-66 |
| DKK1 | 0.68492 | 1.960724 | 1.275804 | 0.000135 | 0.000154 |
| SEC31B | 2.515423 | 0.595732 | -1.91969 | 2.27E-63 | 1.64E-62 |
| MT1H | 3.173836 | 1.986653 | -1.18718 | 2.64E-10 | 3.44E-10 |
| SLX1B-SULT1A4 | 1.581564 | 0.012877 | -1.56869 | 6.96E-62 | 4.34E-61 |
| ARL4C | 1.980444 | 3.645869 | 1.665425 | 1.66E-58 | 7.92E-58 |
| ARHGAP11A | 0.897885 | 2.791049 | 1.893164 | 1.06E-77 | 6.61E-76 |
| ATAD2 | 1.656305 | 3.341808 | 1.685504 | 2.78E-76 | 1.22E-74 |
| AC020659.1 | 1.5688 | 0.193811 | -1.37499 | 4.94E-61 | 2.86E-60 |
| SULT2A1 | 1.621643 | 0.449403 | -1.17224 | 4.22E-33 | 8.55E-33 |
| LINC00092 | 1.316622 | 0.283291 | -1.03333 | 1.86E-74 | 5.86E-73 |
| CDK1 | 1.513742 | 3.708827 | 2.195084 | 1.48E-69 | 2.16E-68 |
| RPRD2 | 2.530466 | 3.820463 | 1.289997 | 5.67E-65 | 4.84E-64 |
| ACYP2 | 2.57863 | 1.450786 | -1.12784 | 1.21E-73 | 3.36E-72 |
| MEMO1 | 2.944754 | 1.063257 | -1.8815 | 3.18E-64 | 2.51E-63 |
| KPNA4 | 2.663179 | 3.977359 | 1.31418 | 9.06E-67 | 9.29E-66 |
| AP1G2 | 4.969487 | 2.848349 | -2.12114 | 1.06E-49 | 3.28E-49 |
| EIF3L | 6.877792 | 5.076054 | -1.80174 | 4.11E-72 | 8.73E-71 |
| FUCA2 | 3.755296 | 5.032091 | 1.276795 | 1.14E-76 | 5.51E-75 |
| CXCL11 | 0.404636 | 2.433024 | 2.028388 | 3.65E-67 | 3.90E-66 |
| ZNF710-AS1 | 3.26783 | 1.771118 | -1.49671 | 3.64E-53 | 1.29E-52 |
| NUBP2 | 4.20148 | 3.101016 | -1.10046 | 3.68E-65 | 3.19E-64 |
| P4HTM | 3.691104 | 2.438256 | -1.25285 | 3.56E-59 | 1.79E-58 |
| HEATR1 | 1.972892 | 3.043797 | 1.070905 | 4.56E-73 | 1.13E-71 |
| TOMM34 | 3.005209 | 4.965971 | 1.960762 | 4.18E-84 | 9.36E-81 |
| NPIPA1 | 4.627297 | 0.528961 | -4.09834 | 1.43E-59 | 7.43E-59 |
| MPRIP | 5.375399 | 3.506093 | -1.86931 | 2.75E-65 | 2.41E-64 |
| RPL21P28 | 1.036907 | 3.343152 | 2.306246 | 8.67E-61 | 4.93E-60 |
| MRPL23 | 4.970316 | 3.384463 | -1.58585 | 1.91E-63 | 1.39E-62 |
| FAM200B | 3.420631 | 2.088965 | -1.33167 | 2.65E-70 | 4.31E-69 |
| CYP4B1 | 2.151281 | 0.421264 | -1.73002 | 1.63E-64 | 1.32E-63 |
| PMAIP1 | 0.97183 | 2.485854 | 1.514024 | 2.86E-59 | 1.45E-58 |
| GINS2 | 0.799812 | 2.440409 | 1.640597 | 1.08E-67 | 1.23E-66 |
| DHRS12 | 3.004567 | 1.721074 | -1.28349 | 8.06E-74 | 2.30E-72 |
| HDAC6 | 4.129537 | 2.389804 | -1.73973 | 1.81E-59 | 9.35E-59 |
| PTP4A3 | 4.826976 | 3.70028 | -1.1267 | 4.54E-30 | 8.64E-30 |
| RPAIN | 3.388147 | 1.740901 | -1.64725 | 1.36E-62 | 9.01E-62 |
| AL390195.1 | 1.408039 | 0.284454 | -1.12358 | 9.04E-60 | 4.75E-59 |
| CDCA8 | 1.320301 | 3.77743 | 2.45713 | 5.89E-77 | 3.07E-75 |
| FO393411.1 | 0.91021 | 3.142309 | 2.232099 | 1.02E-60 | 5.74E-60 |
| GOSR2 | 3.578042 | 2.106016 | -1.47203 | 8.52E-62 | 5.27E-61 |
| RPL37P6 | 0.403809 | 2.115993 | 1.712184 | 3.66E-66 | 3.51E-65 |
| PSMB8 | 4.706962 | 5.860601 | 1.15364 | 3.16E-45 | 8.52E-45 |
| ATP4B | 6.074107 | 0.761452 | -5.31265 | 1.32E-53 | 4.80E-53 |
| MRPS33 | 4.068514 | 2.523265 | -1.54525 | 1.23E-62 | 8.16E-62 |
| PIMREG | 0.657976 | 1.799583 | 1.141607 | 3.21E-56 | 1.33E-55 |
| DCK | 2.099959 | 3.730513 | 1.630554 | 9.76E-76 | 3.88E-74 |
| ZNF146 | 3.38323 | 4.595225 | 1.211995 | 4.80E-80 | 8.19E-78 |
| HSD17B4 | 5.394588 | 3.5712 | -1.82339 | 1.75E-69 | 2.53E-68 |
| AC133550.1 | 1.896472 | 0.008478 | -1.88799 | 9.73E-73 | 2.30E-71 |
| AC023906.1 | 0.265548 | 1.349233 | 1.083684 | 2.02E-58 | 9.56E-58 |
| CFAP410 | 3.471327 | 1.676921 | -1.79441 | 9.13E-76 | 3.64E-74 |
| FAXDC2 | 3.094848 | 1.531773 | -1.56308 | 2.55E-54 | 9.58E-54 |
| AGAP2-AS1 | 1.525729 | 2.663716 | 1.137987 | 3.47E-54 | 1.29E-53 |
| VWA5B2 | 1.69201 | 0.352889 | -1.33912 | 3.20E-53 | 1.14E-52 |
| KLC4 | 3.753109 | 2.628691 | -1.12442 | 8.02E-51 | 2.60E-50 |
| S100B | 2.661095 | 1.444638 | -1.21646 | 7.18E-31 | 1.39E-30 |
| PTPMT1 | 3.923825 | 2.863142 | -1.06068 | 3.46E-57 | 1.52E-56 |
| PCLAF | 1.646081 | 3.191656 | 1.545575 | 1.37E-45 | 3.73E-45 |
| VHL | 2.754102 | 3.885397 | 1.131295 | 2.59E-72 | 5.73E-71 |
| ACBD5 | 2.444508 | 3.842992 | 1.398484 | 8.65E-72 | 1.75E-70 |
| PARD6B | 1.581768 | 2.817725 | 1.235956 | 2.32E-46 | 6.48E-46 |
| KIAA2013 | 3.972907 | 5.178341 | 1.205434 | 5.17E-65 | 4.43E-64 |
| FRMD1 | 2.203837 | 0.237261 | -1.96658 | 8.56E-51 | 2.77E-50 |
| PXMP2 | 4.823036 | 3.323133 | -1.4999 | 2.69E-42 | 6.72E-42 |
| AC074143.1 | 3.847167 | 2.5058 | -1.34137 | 4.91E-58 | 2.27E-57 |
| HMGCL | 3.977365 | 2.755879 | -1.22149 | 1.13E-67 | 1.28E-66 |
| AL162231.1 | 1.470867 | 0.360248 | -1.11062 | 1.53E-66 | 1.53E-65 |
| SPAG16 | 2.719939 | 0.940666 | -1.77927 | 2.75E-77 | 1.53E-75 |
| PTGER3 | 2.836741 | 0.783243 | -2.0535 | 2.66E-69 | 3.75E-68 |
| MYO1D | 3.625677 | 4.736168 | 1.110491 | 3.92E-52 | 1.34E-51 |
| ZNF564 | 1.341812 | 0.315412 | -1.0264 | 1.72E-63 | 1.26E-62 |
| SHROOM3 | 2.81895 | 3.911076 | 1.092126 | 1.03E-30 | 1.99E-30 |
| MSL1 | 4.700044 | 3.691441 | -1.0086 | 2.17E-44 | 5.73E-44 |
| NACA3P | 0.751737 | 1.985901 | 1.234164 | 9.39E-60 | 4.93E-59 |
| DHRS4L2 | 3.438508 | 2.380777 | -1.05773 | 1.04E-61 | 6.37E-61 |
| HLA-A | 7.194702 | 8.745894 | 1.551193 | 8.01E-60 | 4.22E-59 |
| DARS2 | 2.110182 | 3.880765 | 1.770582 | 1.85E-81 | 5.36E-79 |
| ESCO2 | 0.562111 | 1.576167 | 1.014055 | 3.30E-56 | 1.37E-55 |
| NEAT1 | 8.273063 | 4.194633 | -4.07843 | 9.71E-56 | 3.93E-55 |
| SLC27A4 | 3.196609 | 4.412452 | 1.215843 | 7.50E-60 | 3.97E-59 |
| ZNF333 | 2.113032 | 0.83684 | -1.27619 | 5.48E-64 | 4.20E-63 |
| NAIP | 1.844693 | 0.164248 | -1.68045 | 9.75E-65 | 8.14E-64 |
| KIAA1217 | 2.701176 | 3.731972 | 1.030797 | 1.27E-54 | 4.86E-54 |
| GSTM3 | 5.359322 | 3.042933 | -2.31639 | 3.60E-66 | 3.45E-65 |
| CD24P4 | 0.684708 | 2.672984 | 1.988275 | 3.25E-58 | 1.52E-57 |
| ZC3H13 | 2.683058 | 4.00786 | 1.324802 | 6.22E-66 | 5.82E-65 |
| LRFN4 | 2.250194 | 3.901288 | 1.651094 | 9.82E-68 | 1.13E-66 |
| SLK | 3.063635 | 4.114202 | 1.050567 | 6.70E-57 | 2.90E-56 |
| PTAFR | 1.205685 | 2.781833 | 1.576148 | 8.66E-62 | 5.36E-61 |
| MSH5 | 2.170088 | 0.9851 | -1.18499 | 1.26E-39 | 2.94E-39 |
| FER1L4 | 3.914768 | 1.622003 | -2.29276 | 4.06E-31 | 7.88E-31 |
| ALDH5A1 | 1.589547 | 2.753229 | 1.163683 | 8.46E-50 | 2.64E-49 |
| HEYL | 1.755802 | 3.087077 | 1.331275 | 2.76E-43 | 7.06E-43 |
| CARMN | 2.402325 | 0.690895 | -1.71143 | 4.68E-49 | 1.42E-48 |
| CBX4 | 2.775597 | 4.309218 | 1.533621 | 1.55E-74 | 5.03E-73 |
| NDUFA5 | 4.545142 | 2.985643 | -1.5595 | 9.42E-69 | 1.24E-67 |
| CKAP2 | 1.296906 | 3.60361 | 2.306704 | 1.58E-82 | 8.57E-80 |
| NDUFB2 | 6.022316 | 3.76419 | -2.25813 | 4.92E-69 | 6.66E-68 |
| SH3GLB2 | 5.571915 | 3.599321 | -1.97259 | 1.34E-74 | 4.37E-73 |
| NOP53 | 7.150444 | 5.45944 | -1.691 | 1.70E-74 | 5.45E-73 |
| EZH1 | 3.584603 | 2.391873 | -1.19273 | 5.31E-64 | 4.08E-63 |
| MT-TM | 0.223312 | 1.416167 | 1.192856 | 6.11E-37 | 1.35E-36 |
| TM9SF1 | 3.422574 | 1.855477 | -1.5671 | 1.31E-59 | 6.78E-59 |
| C17orf53 | 0.616099 | 1.75399 | 1.137891 | 1.42E-68 | 1.83E-67 |
| IGLV3-19 | 3.972856 | 5.781867 | 1.809011 | 8.18E-14 | 1.14E-13 |
| LINC02210 | 2.644816 | 1.081878 | -1.56294 | 3.32E-63 | 2.35E-62 |
| CXCL1 | 1.849447 | 5.11528 | 3.265834 | 3.22E-62 | 2.06E-61 |
| CLDN1 | 0.77258 | 4.408073 | 3.635493 | 4.81E-78 | 3.25E-76 |
| AC016739.1 | 3.225104 | 5.064666 | 1.839562 | 1.73E-56 | 7.33E-56 |
| TNFSF12 | 4.268921 | 3.174703 | -1.09422 | 1.05E-34 | 2.20E-34 |
| HLA-DRA | 5.992166 | 9.081999 | 3.089833 | 2.37E-64 | 1.89E-63 |
| TRBJ2-7 | 0.215358 | 1.380256 | 1.164898 | 5.09E-35 | 1.08E-34 |
| LINC02193 | 1.690861 | 0.065276 | -1.62558 | 6.91E-71 | 1.21E-69 |
| C15orf40 | 2.640536 | 1.115072 | -1.52546 | 1.89E-66 | 1.88E-65 |
| CDCA7 | 1.327212 | 4.026362 | 2.699149 | 4.13E-75 | 1.49E-73 |
| SCD | 3.126723 | 5.418493 | 2.291771 | 4.24E-58 | 1.97E-57 |
| AC098583.1 | 0.343439 | 1.848426 | 1.504988 | 9.13E-65 | 7.64E-64 |
| MEA1 | 4.449448 | 5.590557 | 1.141109 | 3.24E-65 | 2.83E-64 |
| LMNB1 | 1.765388 | 4.160163 | 2.394774 | 4.02E-76 | 1.73E-74 |
| CCL25 | 0.522033 | 1.927854 | 1.405821 | 4.33E-36 | 9.34E-36 |
| SELENOW | 6.612543 | 4.819344 | -1.7932 | 2.20E-68 | 2.75E-67 |
| PPIP5K1 | 2.705514 | 1.644004 | -1.06151 | 3.87E-57 | 1.70E-56 |
| WBP1 | 4.891029 | 2.188608 | -2.70242 | 1.50E-79 | 2.20E-77 |
| AC023886.2 | 0.217839 | 1.350655 | 1.132816 | 5.46E-69 | 7.36E-68 |
| HDC | 3.136756 | 0.441216 | -2.69554 | 6.80E-75 | 2.36E-73 |
| SCRN2 | 3.755491 | 2.741229 | -1.01426 | 2.38E-63 | 1.71E-62 |
| BEND5 | 1.799177 | 0.627786 | -1.17139 | 3.37E-60 | 1.83E-59 |
| RPL34 | 8.726957 | 6.756314 | -1.97064 | 2.89E-63 | 2.07E-62 |
| LIPA | 3.205026 | 4.517747 | 1.312721 | 1.67E-59 | 8.64E-59 |
| HIF3A | 3.879028 | 1.060301 | -2.81873 | 1.23E-61 | 7.52E-61 |
| EXD3 | 2.944443 | 1.425077 | -1.51937 | 4.66E-64 | 3.61E-63 |
| RBM28 | 2.899742 | 1.745335 | -1.15441 | 1.69E-47 | 4.89E-47 |
| TBC1D14 | 4.128862 | 2.482748 | -1.64611 | 1.42E-63 | 1.05E-62 |
| IFT172 | 3.345934 | 2.228233 | -1.1177 | 1.03E-56 | 4.42E-56 |
| FNDC5 | 2.300531 | 0.557311 | -1.74322 | 7.91E-68 | 9.17E-67 |
| CXCL16 | 3.723709 | 6.151527 | 2.427818 | 7.66E-78 | 4.92E-76 |
| FUT2 | 2.92636 | 4.294579 | 1.368219 | 4.21E-26 | 7.40E-26 |
| ZNRF3 | 1.331322 | 0.293341 | -1.03798 | 1.42E-60 | 7.95E-60 |
| SNORA26 | 0.644206 | 2.033909 | 1.389703 | 2.81E-43 | 7.18E-43 |
| C6orf223 | 0.146402 | 1.590474 | 1.444072 | 1.64E-58 | 7.83E-58 |
| GCHFR | 2.549629 | 1.491352 | -1.05828 | 5.62E-42 | 1.39E-41 |
| VTI1B | 4.884271 | 3.597202 | -1.28707 | 1.00E-74 | 3.38E-73 |
| CHCHD2P2 | 0.276022 | 1.671118 | 1.395095 | 1.29E-65 | 1.18E-64 |
| ALKAL2 | 2.34714 | 0.234476 | -2.11266 | 8.20E-78 | 5.23E-76 |
| LRRC37B | 2.193031 | 1.186233 | -1.0068 | 1.01E-55 | 4.09E-55 |
| RPS26P47 | 0.496574 | 2.328787 | 1.832213 | 2.77E-63 | 1.98E-62 |
| F2RL1 | 2.532654 | 4.550088 | 2.017434 | 2.25E-44 | 5.94E-44 |
| FGFR1 | 3.930199 | 2.303337 | -1.62686 | 1.08E-39 | 2.54E-39 |
| CKAP2L | 0.478789 | 1.984997 | 1.506208 | 1.36E-75 | 5.25E-74 |
| ACCS | 3.38323 | 1.344637 | -2.03859 | 1.18E-62 | 7.86E-62 |
| C1GALT1 | 2.402066 | 3.497776 | 1.09571 | 3.34E-52 | 1.15E-51 |
| PCDH1 | 3.61879 | 4.852167 | 1.233377 | 2.67E-42 | 6.68E-42 |
| CFTR | 0.688974 | 2.351546 | 1.662572 | 2.00E-35 | 4.24E-35 |
| ALG1L6P | 0.219218 | 1.326646 | 1.107428 | 1.03E-65 | 9.50E-65 |
| LRRC8D | 1.9253 | 3.099421 | 1.174121 | 2.76E-76 | 1.22E-74 |
| CLSPN | 0.422632 | 1.978932 | 1.5563 | 6.32E-80 | 1.04E-77 |
| PRR13P5 | 1.030124 | 2.817883 | 1.787759 | 2.32E-62 | 1.50E-61 |
| FAM204A | 3.388934 | 1.399674 | -1.98926 | 2.12E-67 | 2.32E-66 |
| SLC39A5 | 0.712656 | 2.705869 | 1.993214 | 5.56E-39 | 1.28E-38 |
| SERPINA3 | 3.650621 | 0.45075 | -3.19987 | 5.95E-60 | 3.17E-59 |
| RNASE6 | 2.204721 | 3.762385 | 1.557664 | 6.51E-45 | 1.74E-44 |
| BST2 | 4.655266 | 6.545579 | 1.890313 | 4.46E-42 | 1.11E-41 |
| CSTB | 4.781094 | 6.263165 | 1.482072 | 1.54E-61 | 9.31E-61 |
| SPARC | 6.323227 | 7.664993 | 1.341765 | 5.34E-34 | 1.10E-33 |
| EVL | 4.447709 | 2.189743 | -2.25797 | 1.29E-68 | 1.67E-67 |
| PGM5-AS1 | 2.158423 | 0.586656 | -1.57177 | 1.82E-44 | 4.82E-44 |
| PDK4 | 5.50152 | 3.428443 | -2.07308 | 2.56E-39 | 5.94E-39 |
| STRN | 1.879357 | 3.181377 | 1.302019 | 1.64E-66 | 1.64E-65 |
| AL365181.3 | 0.632787 | 2.412275 | 1.779488 | 3.26E-53 | 1.16E-52 |
| IGHV1-69 | 1.730525 | 3.738345 | 2.007821 | 6.25E-26 | 1.10E-25 |
| KLHDC2 | 4.58217 | 2.614113 | -1.96806 | 4.59E-76 | 1.95E-74 |
| ENGASE | 3.920663 | 2.234177 | -1.68649 | 2.54E-49 | 7.78E-49 |
| PIGL | 3.304467 | 1.465673 | -1.83879 | 1.45E-54 | 5.54E-54 |
| IFT20 | 4.230252 | 2.600816 | -1.62944 | 2.32E-66 | 2.27E-65 |
| RHBDD2 | 4.652681 | 5.673452 | 1.02077 | 2.63E-62 | 1.69E-61 |
| SAP30BP | 4.603175 | 2.75137 | -1.85181 | 5.90E-68 | 6.98E-67 |
| MYB | 0.611117 | 2.390102 | 1.778984 | 1.33E-64 | 1.09E-63 |
| 4-Sep | 2.461428 | 0.843097 | -1.61833 | 1.15E-63 | 8.52E-63 |
| MUSTN1 | 2.390801 | 0.039509 | -2.35129 | 2.28E-76 | 1.04E-74 |
| PHF11 | 4.042727 | 2.997652 | -1.04507 | 4.45E-56 | 1.84E-55 |
| TMEM45B | 2.846404 | 5.064208 | 2.217804 | 6.79E-47 | 1.93E-46 |
| CLIC1 | 6.618127 | 7.957875 | 1.339748 | 3.94E-70 | 6.25E-69 |
| WTIP | 2.053315 | 0.722432 | -1.33088 | 2.49E-64 | 1.98E-63 |
| RAD21 | 4.351322 | 5.448978 | 1.097656 | 8.85E-60 | 4.65E-59 |
| NDUFA7 | 4.475584 | 2.424834 | -2.05075 | 7.79E-65 | 6.56E-64 |
| CD83 | 1.243672 | 2.526463 | 1.28279 | 5.17E-57 | 2.25E-56 |
| CYP2S1 | 3.557365 | 5.937522 | 2.380157 | 1.43E-38 | 3.27E-38 |
| GTF2H2 | 1.602429 | 0.376001 | -1.22643 | 3.44E-60 | 1.87E-59 |
| ERO1A | 3.280973 | 4.654479 | 1.373506 | 3.57E-65 | 3.10E-64 |
| TIMM23 | 3.957075 | 5.428423 | 1.471348 | 5.91E-76 | 2.46E-74 |
| TRIM56 | 2.620426 | 3.703359 | 1.082933 | 1.50E-57 | 6.72E-57 |
| APLP1 | 3.692701 | 1.418485 | -2.27422 | 4.15E-53 | 1.47E-52 |
| AGPS | 1.730459 | 3.212662 | 1.482203 | 3.27E-78 | 2.28E-76 |
| COBLL1 | 3.254797 | 2.020882 | -1.23392 | 1.07E-28 | 1.97E-28 |
| RBM5 | 5.360271 | 2.863772 | -2.4965 | 7.92E-63 | 5.38E-62 |
| AC060780.1 | 1.20683 | 2.307777 | 1.100947 | 1.81E-66 | 1.80E-65 |
| LRMDA | 2.613084 | 0.730802 | -1.88228 | 5.60E-65 | 4.78E-64 |
| ATF6 | 2.457471 | 3.640861 | 1.18339 | 2.33E-71 | 4.39E-70 |
| SLC25A3 | 7.134625 | 5.494435 | -1.64019 | 5.41E-70 | 8.44E-69 |
| CCZ1B | 3.509242 | 1.142385 | -2.36686 | 3.78E-52 | 1.29E-51 |
| AC034102.4 | 1.663348 | 0.002008 | -1.66134 | 1.78E-78 | 1.35E-76 |
| AC018475.1 | 0.548252 | 1.878967 | 1.330715 | 9.10E-57 | 3.90E-56 |
| IRF8 | 2.2397 | 3.370556 | 1.130856 | 4.62E-34 | 9.55E-34 |
| RN7SL5P | 0.342086 | 2.144195 | 1.802109 | 1.65E-62 | 1.09E-61 |
| UBA5 | 3.482159 | 2.408228 | -1.07393 | 4.43E-59 | 2.21E-58 |
| AL031587.5 | 2.975413 | 0.741003 | -2.23441 | 4.64E-76 | 1.96E-74 |
| DDC | 0.998151 | 2.905937 | 1.907786 | 1.73E-38 | 3.94E-38 |
| B4GALT1 | 4.185418 | 5.424662 | 1.239244 | 1.15E-58 | 5.57E-58 |
| GPRC5A | 3.074084 | 5.970581 | 2.896496 | 6.78E-60 | 3.60E-59 |
| AC093495.1 | 1.741501 | 0.363959 | -1.37754 | 1.60E-59 | 8.26E-59 |
| MCU | 3.368813 | 4.67272 | 1.303907 | 1.34E-42 | 3.37E-42 |
| MARK3 | 4.601112 | 2.978877 | -1.62223 | 4.24E-58 | 1.97E-57 |
| ULBP2 | 0.358512 | 1.410412 | 1.0519 | 6.01E-43 | 1.52E-42 |
| SLC39A8 | 1.209672 | 2.322403 | 1.112731 | 1.87E-51 | 6.21E-51 |
| AC115618.2 | 1.556577 | 3.447006 | 1.890429 | 3.82E-64 | 2.99E-63 |
| CYFIP2 | 3.525746 | 2.086615 | -1.43913 | 5.76E-56 | 2.36E-55 |
| YTHDF1 | 3.488447 | 4.890586 | 1.402139 | 4.42E-83 | 3.60E-80 |
| AC006329.1 | 0.799329 | 1.872869 | 1.07354 | 1.38E-32 | 2.76E-32 |
| NR2F1-AS1 | 2.136345 | 0.482556 | -1.65379 | 9.01E-69 | 1.19E-67 |
| SRP19 | 4.226985 | 2.383214 | -1.84377 | 5.87E-60 | 3.13E-59 |
| CCKBR | 3.224336 | 0.389688 | -2.83465 | 3.77E-68 | 4.59E-67 |
| AL358232.1 | 0.067064 | 1.169535 | 1.102472 | 2.84E-74 | 8.72E-73 |
| TSPYL2 | 4.934884 | 2.098181 | -2.8367 | 8.30E-70 | 1.26E-68 |
| PAM16 | 3.594923 | 2.03375 | -1.56117 | 1.57E-48 | 4.69E-48 |
| TRIM50 | 3.234631 | 0.23951 | -2.99512 | 7.78E-62 | 4.83E-61 |
| KIF3B | 2.742336 | 4.259231 | 1.516894 | 1.07E-74 | 3.57E-73 |
| SH3BP1 | 1.981801 | 3.25857 | 1.276769 | 7.18E-49 | 2.17E-48 |
| IGHJ2 | 0.484301 | 1.978764 | 1.494463 | 4.02E-29 | 7.49E-29 |
| HIST2H2AA4 | 2.724904 | 0.365394 | -2.35951 | 1.01E-50 | 3.25E-50 |
| ATXN3 | 2.226856 | 0.959608 | -1.26725 | 4.58E-63 | 3.19E-62 |
| TM4SF20 | 0.413729 | 2.329673 | 1.915943 | 1.50E-38 | 3.41E-38 |
| CBR1 | 5.134093 | 4.022282 | -1.11181 | 3.67E-40 | 8.68E-40 |
| C1QTNF3 | 2.635623 | 1.241788 | -1.39384 | 3.09E-49 | 9.43E-49 |
| HCFC1 | 3.495607 | 4.718869 | 1.223262 | 1.48E-70 | 2.49E-69 |
| TMEM198B | 3.635016 | 2.353232 | -1.28178 | 1.60E-56 | 6.76E-56 |
| ETV6 | 2.329032 | 3.762683 | 1.433651 | 2.79E-74 | 8.58E-73 |
| TNFSF9 | 0.482644 | 1.960559 | 1.477916 | 8.88E-52 | 2.99E-51 |
| MDK | 4.386883 | 6.978985 | 2.592102 | 4.57E-72 | 9.57E-71 |
| HLA-E | 6.79901 | 8.244547 | 1.445537 | 1.81E-65 | 1.62E-64 |
| LSR | 4.595463 | 6.94656 | 2.351097 | 8.19E-68 | 9.49E-67 |
| ARCN1 | 4.853625 | 5.945904 | 1.092279 | 5.19E-71 | 9.25E-70 |
| PDIA2 | 4.517303 | 1.398409 | -3.11889 | 3.58E-40 | 8.48E-40 |
| NXT1 | 2.976151 | 4.444925 | 1.468774 | 5.04E-70 | 7.86E-69 |
| RNU1-4 | 1.639922 | 0.032524 | -1.6074 | 2.85E-72 | 6.26E-71 |
| DDX24 | 5.016026 | 3.996804 | -1.01922 | 4.62E-62 | 2.92E-61 |
| AL606489.1 | 0.46727 | 2.381 | 1.91373 | 6.14E-66 | 5.75E-65 |
| MUC17 | 0.427916 | 3.085125 | 2.65721 | 4.44E-54 | 1.65E-53 |
| RPS7P1 | 3.119636 | 4.811025 | 1.691388 | 5.49E-61 | 3.17E-60 |
| MRPL57 | 3.151469 | 4.698837 | 1.547368 | 6.80E-65 | 5.76E-64 |
| CYREN | 3.312252 | 2.304621 | -1.00763 | 3.64E-60 | 1.97E-59 |
| NBPF12 | 3.372364 | 1.180792 | -2.19157 | 6.38E-59 | 3.14E-58 |
| FOXRED2 | 1.632983 | 3.420685 | 1.787702 | 1.58E-74 | 5.10E-73 |
| AC026271.1 | 0.5734 | 1.810588 | 1.237188 | 1.51E-76 | 7.07E-75 |
| LIMD1-AS1 | 2.002902 | 0.075579 | -1.92732 | 1.30E-66 | 1.31E-65 |
| AC021218.1 | 0.745969 | 2.150754 | 1.404785 | 3.40E-36 | 7.37E-36 |
| APCDD1 | 3.533711 | 2.334212 | -1.1995 | 5.94E-38 | 1.34E-37 |
| SMG1P3 | 1.740755 | 0.714972 | -1.02578 | 8.10E-55 | 3.12E-54 |
| PLEKHG6 | 1.851481 | 3.246523 | 1.395042 | 3.14E-39 | 7.28E-39 |
| MDP1 | 2.677339 | 0.746835 | -1.9305 | 3.32E-73 | 8.48E-72 |
| HDGF | 5.598201 | 6.878816 | 1.280615 | 2.81E-74 | 8.64E-73 |
| EGLN3 | 3.372834 | 2.14197 | -1.23086 | 3.00E-49 | 9.18E-49 |
| DTX3 | 3.955997 | 2.125054 | -1.83094 | 1.13E-60 | 6.40E-60 |
| TAZ | 4.054761 | 2.639844 | -1.41492 | 1.61E-53 | 5.83E-53 |
| AREG | 2.55763 | 3.636591 | 1.078962 | 1.17E-15 | 1.68E-15 |
| CNPY2 | 4.855482 | 3.191927 | -1.66356 | 1.07E-48 | 3.20E-48 |
| AC015674.1 | 2.283772 | 0.002008 | -2.28176 | 1.78E-78 | 1.35E-76 |
| NPIPA3 | 1.866101 | 0.170386 | -1.69571 | 2.66E-63 | 1.91E-62 |
| IST1 | 5.344004 | 3.786549 | -1.55745 | 5.48E-64 | 4.20E-63 |
| PCGF3 | 4.070455 | 3.067485 | -1.00297 | 8.94E-51 | 2.89E-50 |
| MTND4P35 | 0.29901 | 2.072241 | 1.77323 | 1.40E-66 | 1.41E-65 |
| YBX1 | 7.245615 | 8.441329 | 1.195715 | 7.38E-69 | 9.82E-68 |
| ITGA6 | 3.929644 | 5.558781 | 1.629137 | 6.79E-57 | 2.94E-56 |
| ANGPTL2 | 3.255287 | 4.263375 | 1.008088 | 3.37E-16 | 4.91E-16 |
| TRBC1 | 1.737766 | 0.697804 | -1.03996 | 9.16E-41 | 2.20E-40 |
| FAM13A | 3.269615 | 1.470159 | -1.79946 | 4.93E-75 | 1.74E-73 |
| NPEPL1 | 3.895869 | 1.438695 | -2.45717 | 1.42E-56 | 6.04E-56 |
| IGHV4-28 | 1.584859 | 2.920648 | 1.335788 | 1.36E-20 | 2.14E-20 |
| CGN | 3.115372 | 4.129176 | 1.013804 | 1.55E-13 | 2.16E-13 |
| SNF8 | 4.963363 | 3.381005 | -1.58236 | 7.02E-62 | 4.38E-61 |
| AGT | 1.698314 | 3.968404 | 2.27009 | 7.83E-53 | 2.75E-52 |
| IMMP2L | 2.442577 | 1.372697 | -1.06988 | 1.59E-64 | 1.29E-63 |
| CLEC3B | 3.934326 | 1.913501 | -2.02083 | 9.39E-60 | 4.93E-59 |
| SULF1 | 1.840794 | 4.328381 | 2.487587 | 3.96E-60 | 2.14E-59 |
| SDHAP1 | 3.438154 | 1.909297 | -1.52886 | 2.63E-56 | 1.10E-55 |
| GPR183 | 2.048639 | 3.152747 | 1.104107 | 6.08E-28 | 1.11E-27 |
| STARD9 | 1.832905 | 0.597043 | -1.23586 | 7.24E-56 | 2.95E-55 |
| KPNA3 | 2.613911 | 4.168519 | 1.554608 | 3.50E-70 | 5.62E-69 |
| C3AR1 | 1.601972 | 2.911579 | 1.309607 | 2.59E-42 | 6.47E-42 |
| ASXL2 | 1.62996 | 2.743176 | 1.113216 | 9.52E-70 | 1.43E-68 |
| RPL10P3 | 0.421367 | 2.022035 | 1.600668 | 3.56E-59 | 1.79E-58 |
| LBX2-AS1 | 1.923053 | 3.166838 | 1.243785 | 3.35E-51 | 1.10E-50 |
| CDH1 | 3.963053 | 6.051699 | 2.088646 | 1.57E-49 | 4.83E-49 |
| POFUT1 | 2.65969 | 4.274887 | 1.615197 | 1.19E-76 | 5.71E-75 |
| KIFC1 | 1.165655 | 3.261464 | 2.095809 | 1.14E-68 | 1.48E-67 |
| ZFYVE21 | 4.165391 | 2.233119 | -1.93227 | 1.69E-73 | 4.54E-72 |
| FAM177A1 | 3.948004 | 2.941899 | -1.0061 | 1.63E-58 | 7.80E-58 |
| TRIM31-AS1 | 0.191811 | 1.521151 | 1.32934 | 9.29E-73 | 2.21E-71 |
| AC099518.5 | 2.16402 | 0.002008 | -2.16201 | 1.78E-78 | 1.35E-76 |
| AC008481.1 | 0.333879 | 1.350218 | 1.01634 | 1.00E-66 | 1.03E-65 |
| AC005831.1 | 0.732005 | 1.859404 | 1.127398 | 1.10E-46 | 3.10E-46 |
| RHOA | 6.790444 | 7.853581 | 1.063136 | 1.03E-66 | 1.05E-65 |
| LINC01106 | 2.374527 | 0.918931 | -1.4556 | 6.90E-29 | 1.28E-28 |
| FNDC1 | 0.418021 | 2.938451 | 2.52043 | 3.23E-68 | 3.96E-67 |
| PNO1 | 2.287328 | 3.451251 | 1.163922 | 2.29E-72 | 5.13E-71 |
| IGF2BP2 | 2.239239 | 3.758004 | 1.518766 | 5.06E-51 | 1.65E-50 |
| SKA3 | 0.614922 | 2.913329 | 2.298407 | 1.22E-80 | 2.64E-78 |
| COL1A2 | 5.384911 | 7.326713 | 1.941803 | 9.22E-42 | 2.27E-41 |
| AC008581.1 | 2.736174 | 0.002008 | -2.73417 | 1.78E-78 | 1.35E-76 |
| AC012181.1 | 1.647759 | 0.620252 | -1.02751 | 2.67E-51 | 8.82E-51 |
| TRMT10C | 2.6532 | 3.722578 | 1.069378 | 1.66E-68 | 2.12E-67 |
| ZC3H3 | 2.714321 | 4.04651 | 1.332188 | 2.71E-76 | 1.20E-74 |
| ACBD3 | 3.171368 | 4.854443 | 1.683074 | 1.33E-75 | 5.15E-74 |
| ID3 | 5.615602 | 4.530423 | -1.08518 | 2.82E-47 | 8.09E-47 |
| COL5A1 | 3.66383 | 4.96755 | 1.30372 | 3.97E-29 | 7.40E-29 |
| SUB1 | 5.340171 | 4.178262 | -1.16191 | 2.83E-56 | 1.18E-55 |
| NUF2 | 0.804295 | 2.450268 | 1.645974 | 4.32E-68 | 5.21E-67 |
| HLA-DQA1 | 2.273959 | 3.979094 | 1.705135 | 9.69E-37 | 2.12E-36 |
| GNB5 | 2.381396 | 1.248408 | -1.13299 | 4.37E-62 | 2.77E-61 |
| CARTPT | 1.5863 | 0.537296 | -1.049 | 4.00E-27 | 7.16E-27 |
| VASH1-AS1 | 2.122337 | 0.722264 | -1.40007 | 2.75E-54 | 1.03E-53 |
| C1GALT1C1 | 2.894239 | 4.478472 | 1.584234 | 5.05E-77 | 2.68E-75 |
| PDIA3P1 | 1.756656 | 3.042912 | 1.286256 | 3.02E-71 | 5.60E-70 |
| SHB | 2.513194 | 3.549063 | 1.035869 | 3.09E-54 | 1.16E-53 |
| TFRC | 3.333802 | 4.811274 | 1.477473 | 6.29E-53 | 2.22E-52 |
| AL590666.2 | 0.712503 | 2.966568 | 2.254066 | 1.90E-61 | 1.14E-60 |
| MYH9 | 5.96025 | 7.477803 | 1.517552 | 2.44E-68 | 3.03E-67 |
| AC104534.1 | 0.432213 | 1.751913 | 1.3197 | 4.55E-45 | 1.22E-44 |
| RTEL1-TNFRSF6B | 1.823007 | 0.750061 | -1.07295 | 2.26E-42 | 5.64E-42 |
| VRK3 | 3.425206 | 2.268848 | -1.15636 | 4.33E-62 | 2.74E-61 |
| LYVE1 | 2.867538 | 1.079944 | -1.78759 | 4.11E-54 | 1.53E-53 |
| BHMT2 | 2.143694 | 0.672444 | -1.47125 | 6.00E-56 | 2.45E-55 |
| GNA13 | 2.882066 | 4.613562 | 1.731497 | 1.57E-65 | 1.41E-64 |
| C16orf89 | 4.620979 | 1.152969 | -3.46801 | 1.36E-70 | 2.30E-69 |
| SLC16A1 | 2.973046 | 4.096758 | 1.123713 | 1.11E-38 | 2.53E-38 |
| MTCO1P12 | 5.814611 | 7.499318 | 1.684707 | 1.18E-35 | 2.53E-35 |
| KIF20B | 0.85159 | 2.615724 | 1.764133 | 1.68E-82 | 8.57E-80 |
| GPR146 | 2.756771 | 0.446396 | -2.31038 | 8.63E-79 | 9.72E-77 |
| LIME1 | 2.531861 | 0.956844 | -1.57502 | 1.37E-54 | 5.22E-54 |
| LIPE-AS1 | 1.442882 | 0.421374 | -1.02151 | 8.09E-66 | 7.48E-65 |
| CPB1 | 1.646864 | 0.113302 | -1.53356 | 1.17E-76 | 5.63E-75 |
| NKX6-3 | 0.484533 | 1.554184 | 1.069651 | 2.54E-06 | 3.03E-06 |
| AC079250.1 | 1.194744 | 3.155464 | 1.96072 | 1.13E-60 | 6.40E-60 |
| ARIH2 | 4.28363 | 3.070011 | -1.21362 | 2.23E-63 | 1.61E-62 |
| TUBB4B | 6.081131 | 7.70065 | 1.619519 | 1.06E-70 | 1.83E-69 |
| MRPL52 | 5.238346 | 3.615638 | -1.62271 | 4.98E-61 | 2.88E-60 |
| CEP55 | 0.903762 | 3.56045 | 2.656688 | 2.78E-81 | 7.33E-79 |
| SRSF10 | 4.587501 | 3.521961 | -1.06554 | 5.98E-44 | 1.56E-43 |
| APOPT1 | 3.747026 | 2.048337 | -1.69869 | 2.38E-70 | 3.90E-69 |
| TGS1 | 1.949279 | 3.322893 | 1.373614 | 7.58E-83 | 5.90E-80 |
| CMC1 | 3.40167 | 1.311773 | -2.0899 | 7.64E-59 | 3.74E-58 |
| MUC13 | 2.028776 | 6.793694 | 4.764917 | 3.44E-68 | 4.21E-67 |
| AC008735.2 | 2.947801 | 1.830858 | -1.11694 | 1.11E-40 | 2.64E-40 |
| PPOX | 3.177392 | 1.652324 | -1.52507 | 4.41E-63 | 3.07E-62 |
| DPT | 4.542217 | 1.927223 | -2.61499 | 1.27E-53 | 4.61E-53 |
| MASTL | 1.452427 | 2.699701 | 1.247274 | 9.19E-79 | 1.01E-76 |
| RNU7-181P | 0.074434 | 1.214544 | 1.140109 | 3.30E-53 | 1.18E-52 |
| SEMA3B-AS1 | 2.208087 | 0.911861 | -1.29623 | 1.49E-36 | 3.26E-36 |
| SLC4A2 | 5.773205 | 4.117691 | -1.65551 | 6.10E-56 | 2.49E-55 |
| ETFB | 4.905734 | 3.273609 | -1.63212 | 5.27E-68 | 6.27E-67 |
| AL132712.2 | 0.164976 | 1.165779 | 1.000804 | 7.28E-57 | 3.15E-56 |
| AC007991.2 | 0.042879 | 1.08965 | 1.046772 | 1.59E-33 | 3.26E-33 |
| RPS15AP17 | 0.324591 | 1.562546 | 1.237955 | 4.97E-58 | 2.29E-57 |
| PRODH | 1.773845 | 0.574803 | -1.19904 | 5.39E-44 | 1.40E-43 |
| HTRA3 | 3.004515 | 4.05068 | 1.046165 | 2.46E-21 | 3.95E-21 |
| RABGGTB | 4.294075 | 3.115112 | -1.17896 | 1.33E-47 | 3.86E-47 |
| DEK | 4.034631 | 5.469327 | 1.434696 | 7.84E-76 | 3.18E-74 |
| TM9SF3 | 4.609296 | 5.759781 | 1.150485 | 1.14E-62 | 7.64E-62 |
| LINC02595 | 0.12176 | 1.336903 | 1.215142 | 3.61E-73 | 9.14E-72 |
| HERC2 | 3.634201 | 2.369347 | -1.26485 | 3.12E-61 | 1.84E-60 |
| LTBP2 | 2.250664 | 3.755209 | 1.504545 | 1.29E-43 | 3.34E-43 |
| SDHC | 5.29832 | 2.808725 | -2.4896 | 2.83E-73 | 7.32E-72 |
| PTGR1 | 5.008889 | 3.252043 | -1.75685 | 3.55E-51 | 1.17E-50 |
| RDH5 | 2.559921 | 0.805606 | -1.75431 | 1.66E-72 | 3.79E-71 |
| ABCB6 | 2.909033 | 0.905662 | -2.00337 | 6.41E-58 | 2.93E-57 |
| TMEM147-AS1 | 2.024371 | 1.009647 | -1.01472 | 9.61E-47 | 2.72E-46 |
| LYPLA1 | 3.645011 | 4.685527 | 1.040516 | 5.76E-55 | 2.24E-54 |
| CXCL3 | 2.217796 | 3.594702 | 1.376906 | 3.55E-22 | 5.79E-22 |
| CCKAR | 1.964895 | 0.338495 | -1.6264 | 2.82E-53 | 1.01E-52 |
| AL157935.2 | 2.015087 | 0.170981 | -1.84411 | 5.33E-66 | 5.02E-65 |
| MMP12 | 0.509993 | 3.891272 | 3.38128 | 1.13E-70 | 1.92E-69 |
| SNCG | 3.039343 | 1.843138 | -1.19621 | 1.67E-30 | 3.19E-30 |
| TOMM6 | 5.040417 | 0.002008 | -5.03841 | 1.78E-78 | 1.35E-76 |
| MAPRE1 | 4.261848 | 5.851903 | 1.590055 | 8.65E-73 | 2.08E-71 |
| RPS29P11 | 0.218137 | 1.312609 | 1.094472 | 4.51E-60 | 2.43E-59 |
| RPS26P11 | 0.236362 | 1.241133 | 1.004771 | 8.60E-59 | 4.19E-58 |
| ZSWIM1 | 1.961975 | 3.352153 | 1.390178 | 4.06E-73 | 1.02E-71 |
| GALM | 2.429857 | 4.227304 | 1.797447 | 4.89E-67 | 5.13E-66 |
| GCNT3 | 1.810975 | 3.715933 | 1.904958 | 3.31E-32 | 6.59E-32 |
| HOPX | 3.300113 | 1.909392 | -1.39072 | 7.81E-32 | 1.54E-31 |
| PXN-AS1 | 2.131149 | 1.031238 | -1.09991 | 1.01E-60 | 5.70E-60 |
| SF3B1 | 6.22488 | 5.150835 | -1.07404 | 7.40E-49 | 2.23E-48 |
| IGHV4-34 | 2.859885 | 5.19998 | 2.340094 | 2.30E-27 | 4.14E-27 |
| KCNMA1 | 2.838245 | 1.281612 | -1.55663 | 4.28E-33 | 8.68E-33 |
| MT1M | 4.72174 | 1.516184 | -3.20556 | 9.99E-71 | 1.73E-69 |
| BUB1 | 0.788527 | 2.896937 | 2.10841 | 1.69E-76 | 7.85E-75 |
| CXCL10 | 1.026261 | 4.197312 | 3.171051 | 1.70E-69 | 2.47E-68 |
| STK4 | 2.138078 | 3.43654 | 1.298462 | 3.76E-78 | 2.59E-76 |
| CCDC14 | 3.296687 | 2.129821 | -1.16687 | 2.15E-43 | 5.51E-43 |
| IGFBP2 | 6.934019 | 4.591625 | -2.34239 | 1.86E-50 | 5.94E-50 |
| MANEAL | 1.27774 | 2.630548 | 1.352808 | 8.30E-50 | 2.59E-49 |
| SLC4A11 | 0.833906 | 2.326955 | 1.493049 | 8.50E-48 | 2.48E-47 |
| NOMO2 | 4.566397 | 1.976179 | -2.59022 | 3.70E-60 | 2.00E-59 |
| TYROBP | 4.24839 | 5.949258 | 1.700867 | 2.82E-46 | 7.86E-46 |
| C12orf57 | 6.152126 | 4.653542 | -1.49858 | 7.10E-65 | 6.00E-64 |
| PDSS1 | 1.640866 | 3.122433 | 1.481567 | 1.04E-67 | 1.18E-66 |
| NBPF10 | 1.262899 | 0.13126 | -1.13164 | 5.30E-67 | 5.54E-66 |
| GIMAP5 | 2.361029 | 0.393643 | -1.96739 | 2.89E-70 | 4.68E-69 |
| AC011498.4 | 1.878602 | 0.096286 | -1.78232 | 1.12E-74 | 3.73E-73 |
| C4B | 3.832082 | 1.561817 | -2.27027 | 6.00E-49 | 1.82E-48 |
| GNAO1 | 2.306316 | 1.118329 | -1.18799 | 5.09E-29 | 9.46E-29 |
| MYZAP | 3.121655 | 0.984414 | -2.13724 | 1.42E-78 | 1.35E-76 |
| MYO5B | 2.447288 | 3.517654 | 1.070366 | 3.80E-25 | 6.56E-25 |
| GDF15 | 3.720452 | 5.000932 | 1.280479 | 1.70E-16 | 2.48E-16 |
| ZNF433-AS1 | 1.894057 | 0.604061 | -1.29 | 2.04E-63 | 1.48E-62 |
| AL049555.1 | 0.937288 | 2.647382 | 1.710094 | 3.78E-58 | 1.76E-57 |
| IFT122 | 2.851371 | 1.765611 | -1.08576 | 3.92E-53 | 1.39E-52 |
| MYH14 | 3.208174 | 5.615795 | 2.407621 | 1.24E-62 | 8.22E-62 |
| UBE2T | 1.626237 | 4.426244 | 2.800007 | 2.91E-80 | 5.31E-78 |
| DIO3OS | 2.653871 | 0.376314 | -2.27756 | 4.39E-74 | 1.31E-72 |
| PPP1R14D | 0.709565 | 2.788886 | 2.079322 | 1.15E-53 | 4.20E-53 |
| FAM174B | 3.351612 | 2.268802 | -1.08281 | 1.43E-26 | 2.53E-26 |
| APTR | 3.024411 | 1.933105 | -1.09131 | 1.51E-50 | 4.83E-50 |
| IFRD2 | 5.091605 | 3.855903 | -1.2357 | 7.51E-56 | 3.05E-55 |
| ATP5MC3 | 6.476715 | 4.738262 | -1.73845 | 3.26E-67 | 3.51E-66 |
| TNS2 | 5.303746 | 3.324321 | -1.97943 | 4.70E-64 | 3.64E-63 |
| WDR86 | 1.965031 | 0.551934 | -1.4131 | 5.37E-60 | 2.87E-59 |
| TRAPPC5 | 3.912462 | 0.744202 | -3.16826 | 5.83E-70 | 9.01E-69 |
| KRT8P3 | 1.087203 | 3.312241 | 2.225038 | 1.65E-60 | 9.17E-60 |
| S100A11 | 7.511711 | 10.2857 | 2.773987 | 4.36E-78 | 2.96E-76 |
| PINK1 | 4.578528 | 2.721306 | -1.85722 | 1.12E-81 | 3.47E-79 |
| EFS | 2.41861 | 1.388225 | -1.03038 | 4.01E-37 | 8.87E-37 |
| ATP8B1 | 3.524152 | 4.538707 | 1.014555 | 1.93E-24 | 3.28E-24 |
| IL1RL1 | 2.564265 | 0.514826 | -2.04944 | 5.61E-61 | 3.23E-60 |
| FUT9 | 1.539131 | 0.49445 | -1.04468 | 4.56E-26 | 8.01E-26 |
| FABP1 | 1.070996 | 2.365926 | 1.29493 | 0.000334 | 0.000377 |
| JDP2 | 3.207144 | 2.105466 | -1.10168 | 2.67E-61 | 1.58E-60 |
| TBC1D3L | 2.29262 | 0.19382 | -2.0988 | 5.60E-46 | 1.55E-45 |
| LAPTM5 | 3.912254 | 5.798735 | 1.886481 | 7.17E-55 | 2.78E-54 |
| MIR621 | 0.539881 | 3.939082 | 3.399201 | 3.73E-72 | 8.03E-71 |
| HOOK2 | 4.336264 | 2.816494 | -1.51977 | 3.38E-50 | 1.07E-49 |
| OLR1 | 0.570461 | 1.759617 | 1.189155 | 1.19E-45 | 3.25E-45 |
| AHR | 2.807518 | 5.031553 | 2.224036 | 5.33E-77 | 2.80E-75 |
| RABL2B | 3.114641 | 1.678616 | -1.43602 | 8.57E-55 | 3.30E-54 |
| ANK2 | 1.897205 | 0.742504 | -1.1547 | 3.45E-45 | 9.30E-45 |
| ANKS3 | 2.741024 | 1.262088 | -1.47894 | 4.38E-55 | 1.71E-54 |
| RNF215 | 2.183431 | 0.703834 | -1.4796 | 2.12E-60 | 1.17E-59 |
| GPA33 | 0.604469 | 2.716014 | 2.111545 | 4.07E-46 | 1.13E-45 |
| GNL1 | 4.077806 | 2.689204 | -1.3886 | 1.56E-66 | 1.56E-65 |
| GPR137B | 2.142954 | 3.601382 | 1.458428 | 7.65E-69 | 1.01E-67 |
| TRUB1 | 1.819728 | 3.202583 | 1.382855 | 4.96E-77 | 2.64E-75 |
| SPC25 | 0.868567 | 2.596209 | 1.727642 | 2.21E-70 | 3.64E-69 |
| ETNPPL | 1.848177 | 0.110842 | -1.73733 | 6.94E-64 | 5.27E-63 |
| CLYBL | 2.132311 | 1.123248 | -1.00906 | 3.74E-59 | 1.88E-58 |
| CEMP1 | 1.454504 | 0.002008 | -1.4525 | 1.78E-78 | 1.35E-76 |
| AC011472.3 | 2.914623 | 0.002008 | -2.91262 | 1.78E-78 | 1.35E-76 |
| LUZP6 | 2.188741 | 0.002008 | -2.18673 | 1.78E-78 | 1.35E-76 |
| INHBA | 0.575101 | 3.012859 | 2.437759 | 2.27E-78 | 1.65E-76 |
| HDAC10 | 3.696355 | 1.653787 | -2.04257 | 4.63E-53 | 1.64E-52 |
| RPL23AP12 | 0.193096 | 1.200604 | 1.007508 | 2.35E-62 | 1.52E-61 |
| SOX12 | 1.805199 | 3.059811 | 1.254612 | 2.26E-54 | 8.51E-54 |
| POC1A | 1.183751 | 2.59717 | 1.413419 | 2.81E-60 | 1.54E-59 |
| SLC7A6OS | 2.282597 | 1.054573 | -1.22802 | 9.50E-65 | 7.94E-64 |
| KDELR2 | 5.231066 | 6.807624 | 1.576559 | 2.67E-77 | 1.49E-75 |
| RBL1 | 0.885838 | 2.262616 | 1.376779 | 1.64E-82 | 8.57E-80 |
| IAH1 | 3.89864 | 2.398023 | -1.50062 | 1.63E-64 | 1.33E-63 |
| DUOX1 | 3.883433 | 1.31436 | -2.56907 | 2.79E-36 | 6.05E-36 |
| CCDC66 | 2.543003 | 1.205299 | -1.3377 | 5.00E-60 | 2.68E-59 |
| ATP9A | 2.906152 | 4.399305 | 1.493153 | 1.63E-61 | 9.82E-61 |
| CCDC146 | 2.574147 | 1.227317 | -1.34683 | 1.31E-55 | 5.28E-55 |
| IGHV3-33 | 3.603225 | 5.126744 | 1.52352 | 4.61E-11 | 6.10E-11 |
| TRAM2 | 2.357227 | 3.502999 | 1.145772 | 1.22E-55 | 4.92E-55 |
| C9orf3 | 4.22809 | 2.112032 | -2.11606 | 4.80E-67 | 5.05E-66 |
| GPR162 | 1.766278 | 0.512023 | -1.25426 | 2.75E-68 | 3.39E-67 |
| SIX5 | 3.203263 | 2.144999 | -1.05826 | 3.32E-54 | 1.24E-53 |
| IGKV2-28 | 4.215981 | 1.627416 | -2.58857 | 1.53E-26 | 2.71E-26 |
| ALDH18A1 | 3.725895 | 5.22221 | 1.496315 | 4.00E-73 | 1.01E-71 |
| TRMU | 3.442569 | 1.845694 | -1.59688 | 1.02E-48 | 3.05E-48 |
| HAUS4 | 3.772914 | 2.743655 | -1.02926 | 3.34E-63 | 2.36E-62 |
| TMEM134 | 4.681518 | 2.516076 | -2.16544 | 6.94E-70 | 1.06E-68 |
| ERCC1 | 4.244721 | 3.120851 | -1.12387 | 2.39E-68 | 2.97E-67 |
| JMJD6 | 3.731981 | 2.417543 | -1.31444 | 9.06E-64 | 6.79E-63 |
| PDZRN3 | 3.50174 | 2.172783 | -1.32896 | 7.84E-29 | 1.45E-28 |
| BEND7 | 2.639603 | 1.539606 | -1.1 | 3.90E-58 | 1.81E-57 |
| HOXC11 | 0.047677 | 1.453143 | 1.405465 | 3.38E-73 | 8.62E-72 |
| CHAMP1 | 2.463364 | 3.665899 | 1.202535 | 2.42E-72 | 5.38E-71 |
| TMX2P1 | 1.460996 | 2.510586 | 1.04959 | 7.47E-60 | 3.95E-59 |
| LINC02585 | 0.165919 | 1.169055 | 1.003136 | 6.84E-68 | 8.02E-67 |
| AC069218.1 | 0.21297 | 1.34466 | 1.13169 | 1.18E-67 | 1.33E-66 |
| ELL2 | 4.147491 | 2.818915 | -1.32858 | 3.05E-59 | 1.54E-58 |
| C16orf45 | 3.257704 | 2.181546 | -1.07616 | 7.42E-32 | 1.46E-31 |
| GFPT1 | 3.046019 | 4.666307 | 1.620289 | 4.44E-66 | 4.22E-65 |
| KCNIP3 | 1.771043 | 0.498962 | -1.27208 | 5.92E-67 | 6.17E-66 |
| CARNS1 | 1.984504 | 0.618893 | -1.36561 | 3.11E-55 | 1.23E-54 |
| AOC1 | 1.074014 | 4.880602 | 3.806588 | 3.08E-66 | 2.97E-65 |
| ASS1 | 4.697362 | 5.776288 | 1.078926 | 5.71E-28 | 1.04E-27 |
| ZBTB7A | 3.241293 | 5.045874 | 1.80458 | 4.85E-56 | 2.00E-55 |
| PFN1 | 7.598464 | 9.081467 | 1.483003 | 6.61E-72 | 1.35E-70 |
| HCP5 | 2.432678 | 4.269514 | 1.836836 | 2.14E-58 | 1.01E-57 |
| LGALS3BP | 6.566523 | 8.35843 | 1.791907 | 1.29E-71 | 2.52E-70 |
| AC003965.2 | 0.129544 | 1.395022 | 1.265478 | 6.25E-66 | 5.84E-65 |
| ITGB1BP1 | 4.120114 | 2.650343 | -1.46977 | 1.98E-61 | 1.19E-60 |
| SEPT7P2 | 2.598688 | 1.452184 | -1.1465 | 5.21E-57 | 2.27E-56 |
| SNRPN | 4.95298 | 2.263779 | -2.6892 | 1.18E-79 | 1.77E-77 |
| AC025259.3 | 1.752246 | 0.534189 | -1.21806 | 5.44E-33 | 1.10E-32 |
| GABBR1 | 3.709631 | 1.418287 | -2.29134 | 1.68E-64 | 1.36E-63 |
| LINC00667 | 3.229335 | 2.093683 | -1.13565 | 1.65E-71 | 3.16E-70 |
| DGCR6 | 1.690746 | 0.392269 | -1.29848 | 1.79E-64 | 1.45E-63 |
| MFGE8 | 5.346352 | 4.082563 | -1.26379 | 1.09E-33 | 2.24E-33 |
| RPL26 | 8.852025 | 6.633546 | -2.21848 | 1.29E-59 | 6.73E-59 |
| FZD2 | 0.841073 | 2.402779 | 1.561706 | 3.67E-68 | 4.47E-67 |
| GRHL2 | 1.794108 | 2.931274 | 1.137166 | 2.28E-43 | 5.85E-43 |
| SCLY | 1.724476 | 0.526703 | -1.19777 | 4.21E-48 | 1.24E-47 |
| ARF4 | 5.602326 | 6.703177 | 1.100851 | 3.45E-69 | 4.77E-68 |
| RNH1 | 5.324256 | 4.053061 | -1.27119 | 1.79E-68 | 2.27E-67 |
| FTLP14 | 0.993309 | 2.113049 | 1.11974 | 1.93E-38 | 4.39E-38 |
| LGALS3 | 6.472122 | 7.490975 | 1.018853 | 4.01E-36 | 8.68E-36 |
| AL445363.3 | 2.734058 | 0.108947 | -2.62511 | 1.06E-64 | 8.78E-64 |
| TMEM123 | 4.613283 | 6.174354 | 1.561071 | 1.05E-76 | 5.12E-75 |
| AL122020.1 | 0.813868 | 2.723853 | 1.909985 | 1.04E-59 | 5.45E-59 |
| MEG3 | 4.076954 | 0.940284 | -3.13667 | 5.23E-66 | 4.93E-65 |
| FTH1P3 | 0.426202 | 1.71647 | 1.290268 | 3.21E-73 | 8.23E-72 |
| AC007849.1 | 0.208804 | 1.220081 | 1.011277 | 4.35E-51 | 1.42E-50 |
| ATP5PD | 6.578195 | 4.79234 | -1.78586 | 3.74E-69 | 5.14E-68 |
| PKIB | 2.946875 | 1.292282 | -1.65459 | 4.95E-27 | 8.84E-27 |
| NEGR1 | 1.855066 | 0.812515 | -1.04255 | 1.57E-43 | 4.04E-43 |
| MIR331 | 0.14555 | 1.234919 | 1.08937 | 4.03E-60 | 2.18E-59 |
| CDKN2B | 1.596142 | 2.597925 | 1.001782 | 1.81E-30 | 3.46E-30 |
| VEGFD | 1.641612 | 0.35597 | -1.28564 | 9.44E-67 | 9.66E-66 |
| CAPZA1 | 4.199964 | 5.34572 | 1.145756 | 4.43E-70 | 6.98E-69 |
| SRSF9 | 5.172988 | 4.080161 | -1.09283 | 2.65E-54 | 9.95E-54 |
| CERS6-AS1 | 2.837217 | 0.055333 | -2.78188 | 1.52E-68 | 1.94E-67 |
| SYAP1 | 2.734684 | 3.994768 | 1.260084 | 4.78E-62 | 3.02E-61 |
| B3GNT2 | 2.62658 | 4.025681 | 1.399101 | 4.92E-66 | 4.65E-65 |
| AES | 7.206938 | 5.769471 | -1.43747 | 1.05E-74 | 3.53E-73 |
| FBXO3 | 3.118519 | 1.96202 | -1.1565 | 4.80E-71 | 8.58E-70 |
| CERS4 | 3.648137 | 1.851279 | -1.79686 | 2.89E-55 | 1.14E-54 |
| MAGIX | 2.465433 | 1.114146 | -1.35129 | 8.13E-35 | 1.71E-34 |
| NRM | 2.358931 | 3.739931 | 1.381 | 1.08E-66 | 1.10E-65 |
| GGH | 1.951633 | 3.574854 | 1.623221 | 1.20E-50 | 3.85E-50 |
| HASPIN | 0.588387 | 1.811336 | 1.222949 | 7.96E-67 | 8.22E-66 |
| SFN | 3.845616 | 6.333586 | 2.48797 | 1.08E-38 | 2.46E-38 |
| KIF20A | 0.791009 | 2.98209 | 2.191082 | 7.08E-77 | 3.62E-75 |
| RUFY3 | 3.489269 | 1.933594 | -1.55567 | 2.98E-65 | 2.60E-64 |
| AC025423.2 | 1.657283 | 0.002008 | -1.65528 | 1.78E-78 | 1.35E-76 |
| AHSA2P | 4.525564 | 2.094695 | -2.43087 | 1.10E-51 | 3.70E-51 |
| KDF1 | 2.240321 | 3.556597 | 1.316277 | 5.88E-40 | 1.38E-39 |
| MRNIP | 3.559991 | 1.37803 | -2.18196 | 3.16E-57 | 1.39E-56 |
| ICAM2 | 2.956194 | 1.691039 | -1.26515 | 4.47E-59 | 2.23E-58 |
| AC011495.1 | 0.681064 | 2.109367 | 1.428303 | 1.48E-58 | 7.11E-58 |
| AC092683.1 | 2.873552 | 0.101692 | -2.77186 | 2.00E-60 | 1.10E-59 |
| NOA1 | 2.719524 | 3.83705 | 1.117526 | 8.68E-58 | 3.94E-57 |
| AC131235.1 | 1.016512 | 2.82432 | 1.807808 | 1.90E-65 | 1.70E-64 |
| CD4 | 2.09146 | 3.588662 | 1.497202 | 4.05E-50 | 1.28E-49 |
| KDELC2 | 2.407853 | 3.481635 | 1.073782 | 1.72E-45 | 4.69E-45 |
| GOLGA8N | 1.979727 | 0.100866 | -1.87886 | 3.45E-75 | 1.26E-73 |
| AL355472.1 | 0.81437 | 2.841505 | 2.027135 | 2.55E-60 | 1.40E-59 |
| NABP2 | 3.447591 | 4.472421 | 1.024831 | 4.88E-70 | 7.64E-69 |
| VPS9D1-AS1 | 1.126037 | 2.689343 | 1.563306 | 7.77E-59 | 3.80E-58 |
| SYT13 | 1.322901 | 2.876211 | 1.55331 | 3.48E-28 | 6.37E-28 |
| VCAN | 2.310962 | 3.732674 | 1.421712 | 2.40E-38 | 5.45E-38 |
| FOXK1 | 2.069312 | 3.116527 | 1.047215 | 6.94E-55 | 2.69E-54 |
| POU2F1 | 1.291118 | 2.413932 | 1.122815 | 4.05E-79 | 5.30E-77 |
| FTH1P12 | 0.385504 | 1.595064 | 1.20956 | 1.89E-65 | 1.69E-64 |
| MIRLET7D | 0.213323 | 1.325832 | 1.112509 | 1.07E-51 | 3.60E-51 |
| HYPK | 2.017366 | 0.473986 | -1.54338 | 3.16E-56 | 1.31E-55 |
| CYB5D1 | 2.194318 | 0.538318 | -1.656 | 2.75E-70 | 4.46E-69 |
| FGFR1OP | 2.03107 | 0.989161 | -1.04191 | 2.17E-49 | 6.67E-49 |
| SLX1A-SULT1A3 | 2.40639 | 0.071526 | -2.33486 | 1.37E-58 | 6.57E-58 |
| SGCA | 3.016829 | 1.180246 | -1.83658 | 8.83E-48 | 2.57E-47 |
| RRP7BP | 2.600876 | 1.175325 | -1.42555 | 6.44E-54 | 2.38E-53 |
| IL16 | 2.200069 | 1.099699 | -1.10037 | 9.04E-51 | 2.92E-50 |
| BAG4 | 1.481089 | 2.743097 | 1.262007 | 4.05E-77 | 2.18E-75 |
| MCM10 | 0.383916 | 1.667535 | 1.283619 | 2.29E-70 | 3.75E-69 |
| TRBV28 | 0.567243 | 2.709795 | 2.142551 | 4.98E-63 | 3.45E-62 |
| TSPAN31 | 3.794001 | 2.673085 | -1.12092 | 2.15E-65 | 1.91E-64 |
| L3HYPDH | 2.910071 | 1.417643 | -1.49243 | 2.30E-54 | 8.67E-54 |
| CYP3A5 | 4.325393 | 3.130458 | -1.19494 | 1.53E-12 | 2.08E-12 |
| CXCL6 | 0.3479 | 1.499165 | 1.151266 | 3.44E-33 | 6.99E-33 |
| SLC22A17 | 3.324324 | 1.597085 | -1.72724 | 1.02E-59 | 5.34E-59 |
| HPRT1 | 3.382896 | 4.700479 | 1.317583 | 3.01E-74 | 9.18E-73 |
| TXNL1 | 4.174585 | 2.737887 | -1.4367 | 1.52E-74 | 4.94E-73 |
| TMEM176B | 5.13024 | 6.394021 | 1.263781 | 7.27E-35 | 1.53E-34 |
| FJX1 | 0.945394 | 1.951033 | 1.005638 | 7.41E-55 | 2.86E-54 |
| MIB2 | 4.18464 | 2.746779 | -1.43786 | 3.33E-54 | 1.24E-53 |
| NME4 | 5.176134 | 3.856027 | -1.32011 | 4.02E-43 | 1.02E-42 |
| RPL9P9 | 7.887735 | 6.382016 | -1.50572 | 4.48E-44 | 1.17E-43 |
| HIST1H3H | 0.799966 | 1.878915 | 1.078949 | 2.45E-43 | 6.28E-43 |
| PILRB | 3.850831 | 1.118997 | -2.73183 | 5.96E-48 | 1.75E-47 |
| AC040162.2 | 0.189744 | 1.290147 | 1.100404 | 2.02E-64 | 1.63E-63 |
| PRELID3B | 3.488816 | 5.562133 | 2.073317 | 2.38E-78 | 1.73E-76 |
| SLC5A5 | 2.572267 | 0.914482 | -1.65779 | 1.61E-19 | 2.49E-19 |
| FAM106A | 1.617233 | 0.108383 | -1.50885 | 2.52E-69 | 3.57E-68 |
| CARS2 | 4.014365 | 2.460307 | -1.55406 | 1.07E-57 | 4.82E-57 |
| RRM1 | 3.14049 | 4.236281 | 1.095791 | 4.54E-66 | 4.31E-65 |
| PDCD6 | 4.680639 | 3.422269 | -1.25837 | 3.71E-53 | 1.32E-52 |
| ARHGAP24 | 2.713873 | 1.406994 | -1.30688 | 4.77E-66 | 4.52E-65 |
| NDUFV1 | 6.042014 | 4.78488 | -1.25713 | 7.91E-64 | 5.97E-63 |
| LRP11 | 2.801882 | 4.189203 | 1.387321 | 1.58E-73 | 4.26E-72 |
| ANKHD1-EIF4EBP3 | 2.539394 | 0.483694 | -2.0557 | 2.04E-67 | 2.24E-66 |
| HBA2 | 4.381793 | 3.155011 | -1.22678 | 2.69E-13 | 3.72E-13 |
| FDX2 | 2.836432 | 1.483502 | -1.35293 | 6.57E-59 | 3.23E-58 |
| MRPL33 | 5.770387 | 4.301606 | -1.46878 | 4.91E-67 | 5.15E-66 |
| ARHGAP32 | 2.017822 | 3.136028 | 1.118206 | 5.79E-53 | 2.04E-52 |
| PNISR | 5.211539 | 3.288777 | -1.92276 | 3.59E-60 | 1.95E-59 |
| RNASEH2C | 4.059091 | 3.004896 | -1.05419 | 1.21E-60 | 6.82E-60 |
| PSMB3 | 5.480867 | 7.093062 | 1.612195 | 3.60E-82 | 1.37E-79 |
| SLC39A6 | 2.781891 | 3.989633 | 1.207742 | 3.64E-69 | 5.02E-68 |
| FTH1 | 10.17437 | 8.769808 | -1.40456 | 2.65E-56 | 1.11E-55 |
| TMSB10P1 | 0.266847 | 2.190664 | 1.923818 | 3.49E-70 | 5.60E-69 |
| GTF2I | 4.774186 | 3.235152 | -1.53903 | 1.95E-58 | 9.27E-58 |
| ZNF331 | 2.748298 | 1.471426 | -1.27687 | 2.08E-44 | 5.49E-44 |
| ESPL1 | 0.824469 | 2.168731 | 1.344262 | 1.23E-56 | 5.22E-56 |
| RPS19 | 8.840409 | 7.258578 | -1.58183 | 1.29E-49 | 4.00E-49 |
| IGKV3D-20 | 1.776036 | 3.27605 | 1.500015 | 5.22E-20 | 8.15E-20 |
| ZDHHC11B | 2.367164 | 1.309916 | -1.05725 | 1.97E-31 | 3.84E-31 |
| PPP1R3E | 2.906712 | 1.42089 | -1.48582 | 4.28E-65 | 3.68E-64 |
| ZFPL1 | 3.797013 | 2.072821 | -1.72419 | 6.81E-70 | 1.04E-68 |
| RPS24P8 | 0.620588 | 1.898862 | 1.278273 | 1.57E-65 | 1.41E-64 |
| KDELR3 | 2.691158 | 5.096332 | 2.405174 | 3.08E-69 | 4.30E-68 |
| AKTIP | 3.205167 | 2.141246 | -1.06392 | 8.82E-67 | 9.07E-66 |
| GBGT1 | 3.08965 | 1.362986 | -1.72666 | 7.19E-60 | 3.81E-59 |
| RNU6-125P | 0.183095 | 1.657551 | 1.474455 | 4.22E-62 | 2.68E-61 |
| MT-TF | 0.563933 | 2.682145 | 2.118212 | 1.38E-52 | 4.79E-52 |
| CTSF | 4.595445 | 2.866225 | -1.72922 | 1.42E-57 | 6.38E-57 |
| ALDH7A1 | 3.886138 | 2.656015 | -1.23012 | 1.36E-58 | 6.52E-58 |
| NPIPB13 | 1.554361 | 0.328916 | -1.22545 | 1.04E-57 | 4.70E-57 |
| THEM6 | 2.960164 | 4.795655 | 1.835491 | 2.05E-71 | 3.88E-70 |
| NXPH4 | 0.250407 | 1.273831 | 1.023424 | 2.19E-41 | 5.34E-41 |
| ABHD11-AS1 | 0.806474 | 2.359351 | 1.552877 | 1.91E-52 | 6.59E-52 |
| AL138478.1 | 2.961262 | 0.002008 | -2.95925 | 1.78E-78 | 1.35E-76 |
| SYNM | 4.2835 | 2.626631 | -1.65687 | 8.88E-22 | 1.44E-21 |
| TMEM138 | 3.723676 | 2.695188 | -1.02849 | 2.55E-46 | 7.13E-46 |
| RF02119 | 0.159227 | 1.382537 | 1.22331 | 1.70E-53 | 6.14E-53 |
| PAIP2B | 2.211459 | 0.905887 | -1.30557 | 1.72E-64 | 1.39E-63 |
| TMEM104 | 1.87233 | 2.891623 | 1.019293 | 2.24E-68 | 2.80E-67 |
| SECISBP2 | 3.723152 | 2.268742 | -1.45441 | 1.49E-60 | 8.32E-60 |
| CSAD | 4.010046 | 1.84748 | -2.16257 | 2.83E-58 | 1.33E-57 |
| CALCOCO1 | 5.536215 | 2.989344 | -2.54687 | 3.30E-75 | 1.21E-73 |
| COL10A1 | 0.174036 | 2.836952 | 2.662916 | 3.01E-69 | 4.21E-68 |
| STX8 | 4.192851 | 2.575846 | -1.617 | 7.44E-76 | 3.04E-74 |
| FANCI | 1.623054 | 2.79823 | 1.175176 | 2.01E-55 | 7.99E-55 |
| STK19 | 3.38377 | 1.511732 | -1.87204 | 1.62E-67 | 1.80E-66 |
| TRIM3 | 3.455476 | 1.561302 | -1.89417 | 2.00E-72 | 4.49E-71 |
| XAF1 | 3.260099 | 1.885038 | -1.37506 | 3.01E-41 | 7.29E-41 |
| TAF1D | 4.735094 | 3.211815 | -1.52328 | 5.90E-46 | 1.63E-45 |
| GOLGA2P7 | 3.389145 | 0.425303 | -2.96384 | 1.49E-66 | 1.49E-65 |
| AC093484.3 | 3.910237 | 0.002008 | -3.90823 | 1.78E-78 | 1.35E-76 |
| CDK5RAP3 | 5.929874 | 3.591747 | -2.33813 | 2.22E-50 | 7.07E-50 |
| AC004453.1 | 2.043229 | 4.437796 | 2.394567 | 4.52E-59 | 2.26E-58 |
| ACADVL | 7.661778 | 5.186446 | -2.47533 | 4.64E-73 | 1.15E-71 |
| QPCT | 1.021776 | 2.058384 | 1.036608 | 2.94E-33 | 5.99E-33 |
| RPL17 | 8.621104 | 5.062631 | -3.55847 | 1.22E-65 | 1.11E-64 |
| PTH1R | 1.76624 | 0.354023 | -1.41222 | 4.29E-76 | 1.84E-74 |
| WDR34 | 3.774253 | 4.82981 | 1.055557 | 1.91E-53 | 6.87E-53 |
| AGTR1 | 1.573646 | 0.492356 | -1.08129 | 3.45E-48 | 1.02E-47 |
| LONRF2 | 1.470647 | 0.461974 | -1.00867 | 1.96E-55 | 7.80E-55 |
| DYRK2 | 2.090992 | 3.246281 | 1.15529 | 9.89E-72 | 1.97E-70 |
| RPS29P17 | 0.195535 | 1.238942 | 1.043407 | 1.69E-62 | 1.11E-61 |
| AFF3 | 1.637083 | 0.432084 | -1.205 | 3.52E-67 | 3.77E-66 |
| MCUR1 | 2.150051 | 3.318339 | 1.168288 | 4.70E-69 | 6.37E-68 |
| NT5E | 2.169534 | 3.549175 | 1.379641 | 2.16E-37 | 4.79E-37 |
| COL28A1 | 2.184563 | 0.726168 | -1.45839 | 7.10E-57 | 3.07E-56 |
| VAPA | 5.353453 | 3.951581 | -1.40187 | 5.86E-70 | 9.05E-69 |
| NPIPA9 | 4.962751 | 0.039514 | -4.92324 | 9.04E-62 | 5.58E-61 |
| MAL2 | 3.867595 | 6.697759 | 2.830164 | 1.24E-65 | 1.13E-64 |
| THOC1 | 3.380034 | 1.915538 | -1.4645 | 1.42E-49 | 4.38E-49 |
| GIHCG | 2.153347 | 0.94819 | -1.20516 | 1.34E-57 | 6.00E-57 |
| RUNX3 | 1.319319 | 2.724207 | 1.404887 | 1.78E-53 | 6.43E-53 |
| PSMA3-AS1 | 3.773164 | 2.317209 | -1.45595 | 6.70E-55 | 2.60E-54 |
| HTD2 | 1.297339 | 0.067921 | -1.22942 | 3.11E-67 | 3.35E-66 |
| MRPL46 | 3.126982 | 1.309429 | -1.81755 | 1.08E-68 | 1.41E-67 |
| ABI1 | 3.403036 | 4.488258 | 1.085222 | 2.43E-67 | 2.64E-66 |
| PRPF40B | 3.087768 | 0.776666 | -2.3111 | 9.12E-66 | 8.42E-65 |
| ANGPTL1 | 2.586686 | 1.12487 | -1.46182 | 2.09E-34 | 4.36E-34 |
| BLOC1S1 | 5.782085 | 4.137308 | -1.64478 | 5.07E-71 | 9.05E-70 |
| MEIS2 | 2.740445 | 1.47973 | -1.26072 | 8.95E-49 | 2.70E-48 |
| SQSTM1 | 6.458657 | 5.327412 | -1.13125 | 1.34E-51 | 4.49E-51 |
| RIPOR3 | 2.184334 | 0.742439 | -1.4419 | 7.20E-65 | 6.08E-64 |
| SNRNP70 | 6.533658 | 4.883972 | -1.64969 | 3.37E-49 | 1.03E-48 |
| DYNC2H1 | 1.754236 | 0.5798 | -1.17444 | 1.07E-66 | 1.09E-65 |
| ARHGAP10 | 3.227638 | 1.332066 | -1.89557 | 5.06E-68 | 6.03E-67 |
| APOE | 4.070531 | 6.529467 | 2.458935 | 5.89E-58 | 2.71E-57 |
| C15orf39 | 2.553846 | 3.749487 | 1.195641 | 1.23E-62 | 8.19E-62 |
| MTRNR2L8 | 0.891644 | 2.694663 | 1.803019 | 9.81E-54 | 3.59E-53 |
| FCN3 | 1.664708 | 0.530915 | -1.13379 | 1.98E-44 | 5.22E-44 |
| AL117382.1 | 0.364893 | 1.520758 | 1.155866 | 9.44E-49 | 2.84E-48 |
| AC025459.1 | 0.317415 | 1.335038 | 1.017623 | 7.08E-51 | 2.29E-50 |
| LGALS4 | 4.441172 | 7.162738 | 2.721567 | 1.66E-39 | 3.87E-39 |
| AL359076.1 | 0.81556 | 1.885087 | 1.069526 | 4.13E-55 | 1.62E-54 |
| ZNF488 | 0.447335 | 1.534629 | 1.087294 | 1.70E-47 | 4.91E-47 |
| RNASE4 | 4.502488 | 1.399812 | -3.10268 | 7.75E-71 | 1.36E-69 |
| CDK18 | 3.937087 | 2.71512 | -1.22197 | 1.46E-32 | 2.93E-32 |
| MIS18A | 1.752935 | 3.158274 | 1.405339 | 3.18E-72 | 6.94E-71 |
| SOCS1 | 2.293334 | 3.518883 | 1.225549 | 9.03E-44 | 2.34E-43 |
| LINC01128 | 2.246091 | 1.061107 | -1.18498 | 1.03E-65 | 9.50E-65 |
| CXCL5 | 1.080868 | 3.951276 | 2.870407 | 6.95E-35 | 1.46E-34 |
| RPL10AP2 | 0.463347 | 1.518008 | 1.054661 | 1.10E-58 | 5.30E-58 |
| ZSCAN18 | 3.9338 | 1.046506 | -2.88729 | 2.05E-76 | 9.46E-75 |
| MTND6P3 | 0.420915 | 1.679748 | 1.258833 | 1.04E-51 | 3.49E-51 |
| RNU1-1 | 1.647303 | 0.079064 | -1.56824 | 2.48E-63 | 1.78E-62 |
| NET1 | 4.359932 | 5.396903 | 1.036971 | 1.39E-48 | 4.16E-48 |
| KCNJ15 | 2.188737 | 0.704585 | -1.48415 | 2.39E-39 | 5.56E-39 |
| SPRY4 | 2.18501 | 3.312672 | 1.127662 | 1.94E-57 | 8.65E-57 |
| PDLIM3 | 4.560815 | 2.898467 | -1.66235 | 7.54E-30 | 1.43E-29 |
| UBE2V1 | 4.416807 | 2.002711 | -2.4141 | 7.71E-55 | 2.98E-54 |
| DSCC1 | 0.975144 | 2.510669 | 1.535525 | 3.38E-78 | 2.35E-76 |
| CHMP1B-AS1 | 1.683806 | 0.002008 | -1.6818 | 1.78E-78 | 1.35E-76 |
| RIMKLB | 2.956439 | 1.071988 | -1.88445 | 1.51E-61 | 9.15E-61 |
| PPP1R16A | 4.508682 | 3.070787 | -1.43789 | 1.97E-46 | 5.52E-46 |
| IKBKE | 1.621706 | 2.899678 | 1.277973 | 8.34E-57 | 3.59E-56 |
| EXOC7 | 4.084411 | 2.985512 | -1.0989 | 4.35E-73 | 1.08E-71 |
| SGO2 | 0.621202 | 1.706282 | 1.08508 | 4.01E-71 | 7.30E-70 |
| CLN3 | 3.95636 | 2.623847 | -1.33251 | 2.79E-56 | 1.17E-55 |
| KLF15 | 3.450262 | 1.014078 | -2.43618 | 4.66E-72 | 9.74E-71 |
| TMEM110-MUSTN1 | 1.28195 | 0.094107 | -1.18784 | 1.31E-68 | 1.69E-67 |
| ETS2 | 4.64864 | 5.741475 | 1.092835 | 5.81E-28 | 1.06E-27 |
| AC027644.3 | 2.285427 | 1.106572 | -1.17885 | 2.69E-58 | 1.27E-57 |
| CDC25A | 0.619652 | 1.837264 | 1.217611 | 2.27E-63 | 1.64E-62 |
| MORF4L1 | 5.961512 | 4.750488 | -1.21102 | 1.89E-58 | 8.97E-58 |
| GADD45B | 5.546972 | 3.890966 | -1.65601 | 2.10E-49 | 6.45E-49 |
| SAT2 | 5.41455 | 3.877898 | -1.53665 | 1.19E-74 | 3.91E-73 |
| CLRN3 | 0.486268 | 3.62738 | 3.141113 | 2.35E-64 | 1.88E-63 |
| FAR2P2 | 1.351346 | 0.131775 | -1.21957 | 1.26E-72 | 2.95E-71 |
| HBP1 | 3.979602 | 2.903197 | -1.0764 | 4.90E-68 | 5.86E-67 |
| HIST3H2A | 1.867482 | 3.113752 | 1.24627 | 5.79E-35 | 1.22E-34 |
| HIST1H2BK | 3.968944 | 5.775278 | 1.806334 | 2.65E-56 | 1.11E-55 |
| AC009244.1 | 2.394255 | 1.046583 | -1.34767 | 2.29E-40 | 5.46E-40 |
| LIN7C | 2.246307 | 3.633353 | 1.387046 | 3.21E-69 | 4.47E-68 |
| MIR570 | 0.394164 | 3.146175 | 2.752011 | 5.80E-71 | 1.03E-69 |
| MT3 | 1.286608 | 0.107043 | -1.17956 | 4.05E-75 | 1.46E-73 |
| TMSB4XP8 | 2.277342 | 5.728947 | 3.451604 | 1.32E-65 | 1.20E-64 |
| LOX | 1.468235 | 2.983408 | 1.515174 | 1.36E-57 | 6.10E-57 |
| FEZ2 | 3.30687 | 2.251871 | -1.055 | 1.40E-65 | 1.26E-64 |
| MIR3685 | 0.269465 | 2.705325 | 2.43586 | 1.97E-69 | 2.82E-68 |
| HIST1H1E | 0.811834 | 2.430764 | 1.61893 | 3.68E-51 | 1.21E-50 |
| ADAMTSL1 | 1.544475 | 0.539434 | -1.00504 | 3.04E-66 | 2.94E-65 |
| DCN | 6.781977 | 4.483404 | -2.29857 | 3.19E-54 | 1.19E-53 |
| STAU1 | 4.314016 | 5.717624 | 1.403608 | 1.07E-79 | 1.61E-77 |
| MAPK6 | 2.386227 | 3.726923 | 1.340696 | 1.27E-62 | 8.44E-62 |
| NUMA1 | 5.59201 | 4.02895 | -1.56306 | 1.12E-61 | 6.85E-61 |
| CPT1A | 3.537897 | 4.656217 | 1.11832 | 6.80E-50 | 2.12E-49 |
| GGNBP2 | 4.175679 | 2.957495 | -1.21818 | 1.10E-56 | 4.69E-56 |
| BAZ1B | 3.141171 | 4.505 | 1.363829 | 9.76E-77 | 4.79E-75 |
| LMF1 | 2.811187 | 1.348035 | -1.46315 | 3.33E-72 | 7.25E-71 |
| UQCC2 | 4.097425 | 2.555588 | -1.54184 | 4.70E-59 | 2.34E-58 |
| TRAM1 | 4.64676 | 6.281135 | 1.634375 | 2.17E-79 | 3.08E-77 |
| PTGDS | 4.716499 | 3.653993 | -1.06251 | 7.36E-15 | 1.05E-14 |
| KIF18B | 0.789427 | 2.758787 | 1.96936 | 4.09E-72 | 8.71E-71 |
| RPS11P5 | 1.308396 | 3.373344 | 2.064948 | 2.66E-63 | 1.91E-62 |
| SNORA33 | 0.808533 | 2.882896 | 2.074363 | 5.93E-49 | 1.80E-48 |
| ZFAND2B | 4.130734 | 2.847447 | -1.28329 | 2.79E-73 | 7.22E-72 |
| RAP2B | 2.069796 | 3.676442 | 1.606645 | 2.26E-61 | 1.35E-60 |
| CLK3 | 4.253937 | 1.652884 | -2.60105 | 1.15E-71 | 2.27E-70 |
| UHMK1 | 2.523118 | 4.039946 | 1.516828 | 1.35E-80 | 2.88E-78 |
| APLNR | 1.299702 | 3.422264 | 2.122561 | 6.49E-63 | 4.45E-62 |
| CYB5R1 | 5.039079 | 3.045761 | -1.99332 | 1.28E-75 | 4.95E-74 |
| OR2A9P | 1.343366 | 0.109604 | -1.23376 | 3.95E-63 | 2.77E-62 |
| CUL9 | 3.424328 | 2.077068 | -1.34726 | 4.54E-59 | 2.26E-58 |
| RGS2 | 5.286043 | 4.17671 | -1.10933 | 3.10E-24 | 5.26E-24 |
| RPL35A | 8.293047 | 7.079464 | -1.21358 | 1.48E-56 | 6.27E-56 |
| IGHV3-48 | 1.905183 | 4.211099 | 2.305916 | 2.35E-33 | 4.79E-33 |
| VLDLR | 2.215443 | 0.915783 | -1.29966 | 7.63E-60 | 4.03E-59 |
| KIF14 | 0.305531 | 1.635174 | 1.329643 | 1.25E-77 | 7.58E-76 |
| CD59 | 6.694775 | 5.600553 | -1.09422 | 1.14E-54 | 4.35E-54 |
| LIFR | 3.110085 | 1.332472 | -1.77761 | 9.39E-67 | 9.63E-66 |
| BRCC3 | 2.352264 | 3.775037 | 1.422773 | 1.68E-71 | 3.22E-70 |
| RALA | 3.109314 | 4.395612 | 1.286298 | 2.12E-64 | 1.70E-63 |
| CEBPB | 4.569361 | 6.087794 | 1.518434 | 2.94E-47 | 8.42E-47 |
| RPL13AP25 | 1.1366 | 4.873142 | 3.736542 | 1.12E-62 | 7.45E-62 |
| CEMIP | 0.602446 | 2.749028 | 2.146582 | 3.28E-69 | 4.56E-68 |
| YWHAG | 4.079203 | 5.769533 | 1.69033 | 1.88E-77 | 1.09E-75 |
| ZWILCH | 1.394109 | 2.753348 | 1.359239 | 8.90E-80 | 1.37E-77 |
| HOMER2 | 3.629783 | 0.768338 | -2.86145 | 1.01E-66 | 1.03E-65 |
| AP000763.2 | 0.925492 | 4.09938 | 3.173888 | 9.82E-61 | 5.56E-60 |
| PIGG | 3.722215 | 2.120574 | -1.60164 | 1.03E-53 | 3.76E-53 |
| RPL11P3 | 0.611387 | 1.926484 | 1.315098 | 6.09E-68 | 7.18E-67 |
| ATP10B | 0.938103 | 2.649587 | 1.711484 | 5.18E-36 | 1.11E-35 |
| B3GNT3 | 3.400769 | 5.428431 | 2.027662 | 2.04E-45 | 5.55E-45 |
| MUC3A | 0.840607 | 4.025791 | 3.185184 | 5.37E-60 | 2.87E-59 |
| ADAMTS13 | 1.854854 | 0.631443 | -1.22341 | 3.32E-64 | 2.62E-63 |
| AC010442.1 | 4.80981 | 3.290489 | -1.51932 | 2.60E-24 | 4.42E-24 |
| MGP | 6.67722 | 5.659526 | -1.01769 | 1.55E-08 | 1.95E-08 |
| TICAM1 | 3.113427 | 4.25782 | 1.144393 | 6.33E-49 | 1.92E-48 |
| SIPA1L3 | 2.225512 | 3.97734 | 1.751828 | 1.08E-69 | 1.60E-68 |
| TRAPPC4 | 4.255576 | 2.904917 | -1.35066 | 8.29E-58 | 3.76E-57 |
| NCOA2 | 2.150946 | 3.238189 | 1.087242 | 1.05E-63 | 7.83E-63 |
| GRHPR | 4.76766 | 3.253357 | -1.5143 | 3.18E-75 | 1.17E-73 |
| USP15 | 3.698214 | 1.954674 | -1.74354 | 2.06E-59 | 1.06E-58 |
| AC139491.2 | 1.610232 | 0.254053 | -1.35618 | 6.98E-61 | 3.99E-60 |
| CDX1 | 0.437539 | 3.156128 | 2.718588 | 1.71E-47 | 4.94E-47 |
| BCL2A1 | 1.107086 | 2.786826 | 1.67974 | 1.29E-52 | 4.49E-52 |
| NCOA3 | 2.521526 | 3.742899 | 1.221372 | 4.54E-68 | 5.46E-67 |
| COMMD8 | 2.470682 | 3.606833 | 1.136151 | 2.30E-63 | 1.66E-62 |
| HMOX1 | 2.743285 | 3.816321 | 1.073036 | 3.84E-25 | 6.63E-25 |
| EIF3M | 5.870925 | 4.207591 | -1.66333 | 1.66E-53 | 6.02E-53 |
| SCN7A | 1.592214 | 0.402584 | -1.18963 | 2.83E-54 | 1.06E-53 |
| MAGI2-AS3 | 2.45367 | 0.869307 | -1.58436 | 2.37E-60 | 1.30E-59 |
| TRIM74 | 1.6835 | 0.103317 | -1.58018 | 5.14E-71 | 9.17E-70 |
| CADM3 | 2.215539 | 0.619242 | -1.5963 | 2.70E-59 | 1.37E-58 |
| PIGT | 5.080085 | 6.873252 | 1.793167 | 4.25E-76 | 1.83E-74 |
| NR4A1 | 5.683452 | 3.802208 | -1.88124 | 2.29E-31 | 4.48E-31 |
| MRPL50 | 2.34307 | 3.543434 | 1.200365 | 4.50E-73 | 1.12E-71 |
| MICB | 0.90989 | 2.19992 | 1.29003 | 1.27E-60 | 7.13E-60 |
| PKD1 | 4.785153 | 2.632007 | -2.15315 | 1.71E-60 | 9.48E-60 |
| RAB17 | 3.668292 | 2.2126 | -1.45569 | 1.55E-18 | 2.36E-18 |
| EIF2S2 | 4.431538 | 5.721543 | 1.290005 | 6.00E-76 | 2.49E-74 |
| PRR34-AS1 | 2.935309 | 1.808636 | -1.12667 | 8.30E-52 | 2.80E-51 |
| BCL6 | 4.39003 | 3.156802 | -1.23323 | 5.52E-34 | 1.14E-33 |
| PSMG4 | 2.931779 | 1.292444 | -1.63934 | 3.59E-59 | 1.81E-58 |
| RBBP9 | 1.853067 | 3.443921 | 1.590854 | 1.19E-66 | 1.21E-65 |
| RMND5B | 3.60919 | 2.51727 | -1.09192 | 6.93E-75 | 2.40E-73 |
| C1S | 6.11131 | 5.079667 | -1.03164 | 2.69E-19 | 4.15E-19 |
| CCL15 | 0.285598 | 1.868647 | 1.583049 | 1.40E-59 | 7.25E-59 |
| AF127577.1 | 1.665283 | 0.002008 | -1.66328 | 1.78E-78 | 1.35E-76 |
| ITIH4 | 2.125954 | 0.112092 | -2.01386 | 5.59E-68 | 6.63E-67 |
| RASA4 | 3.09909 | 0.116788 | -2.9823 | 4.48E-76 | 1.91E-74 |
| TRAPPC12 | 4.124402 | 2.5497 | -1.5747 | 4.13E-74 | 1.23E-72 |
| SSR2 | 6.205951 | 5.166101 | -1.03985 | 8.88E-54 | 3.25E-53 |
| FLII | 6.030042 | 4.807807 | -1.22223 | 5.44E-66 | 5.12E-65 |
| AC087741.1 | 1.962656 | 0.740016 | -1.22264 | 3.98E-45 | 1.07E-44 |
| RSRP1 | 4.940986 | 2.666201 | -2.27478 | 8.92E-53 | 3.12E-52 |
| FAM157C | 1.405824 | 0.190337 | -1.21549 | 8.38E-63 | 5.67E-62 |
| SLC46A1 | 2.399694 | 0.996359 | -1.40334 | 1.30E-63 | 9.60E-63 |
| PLEKHO1 | 4.273243 | 2.72296 | -1.55028 | 3.52E-33 | 7.15E-33 |
| AL450306.1 | 1.618821 | 0.149749 | -1.46907 | 4.96E-60 | 2.66E-59 |
| RASGRP2 | 3.062898 | 0.971662 | -2.09124 | 9.70E-66 | 8.94E-65 |
| CCPG1 | 3.78194 | 1.955232 | -1.82671 | 5.36E-63 | 3.70E-62 |
| AC015813.1 | 2.430158 | 1.167492 | -1.26267 | 1.92E-48 | 5.72E-48 |
| RNF217 | 1.854947 | 0.84643 | -1.00852 | 1.69E-58 | 8.05E-58 |
| MST1L | 2.652309 | 0.796286 | -1.85602 | 2.31E-48 | 6.85E-48 |
| CCZ1 | 3.495106 | 1.077179 | -2.41793 | 1.30E-53 | 4.74E-53 |
| RNLS | 1.816203 | 0.517979 | -1.29822 | 4.20E-68 | 5.07E-67 |
| EIF3CL | 3.504564 | 0.762926 | -2.74164 | 2.01E-58 | 9.53E-58 |
| MSH2 | 1.785066 | 3.070844 | 1.285778 | 1.18E-78 | 1.21E-76 |
| SEC22C | 3.481393 | 2.226168 | -1.25523 | 4.62E-74 | 1.37E-72 |
| HOXB9 | 0.214257 | 2.467193 | 2.252935 | 1.15E-64 | 9.50E-64 |
| AL136454.1 | 1.71505 | 3.623841 | 1.908791 | 2.15E-59 | 1.10E-58 |
| SERPINH1 | 4.33153 | 5.525951 | 1.194421 | 5.52E-41 | 1.33E-40 |
| PDK3 | 1.596 | 2.62968 | 1.03368 | 2.77E-40 | 6.59E-40 |
| ACKR1 | 4.092517 | 2.632706 | -1.45981 | 6.83E-24 | 1.15E-23 |
| RABGEF1 | 2.683047 | 1.034062 | -1.64898 | 3.93E-71 | 7.18E-70 |
| UROS | 3.537738 | 2.429273 | -1.10846 | 5.24E-64 | 4.03E-63 |
| ELMOD3 | 3.090942 | 1.59848 | -1.49246 | 4.19E-73 | 1.05E-71 |
| PTBP3 | 2.724529 | 4.925114 | 2.200585 | 5.73E-78 | 3.79E-76 |
| TCTN1 | 2.751355 | 1.344698 | -1.40666 | 6.62E-66 | 6.17E-65 |
| DTWD1 | 2.91741 | 1.121605 | -1.79581 | 3.28E-65 | 2.86E-64 |
| ZNF92 | 1.279602 | 2.414234 | 1.134632 | 7.18E-73 | 1.74E-71 |
| COX20 | 3.591736 | 1.222748 | -2.36899 | 8.54E-68 | 9.87E-67 |
| RNU6-516P | 0.246433 | 1.730474 | 1.484041 | 1.15E-62 | 7.67E-62 |
| ATP5MC2 | 7.175134 | 5.614362 | -1.56077 | 3.88E-67 | 4.14E-66 |
| RPL30P4 | 0.358505 | 1.966986 | 1.608481 | 1.67E-65 | 1.50E-64 |
| ABI3BP | 3.289432 | 1.787886 | -1.50155 | 1.59E-24 | 2.71E-24 |
| EPN1 | 5.930788 | 4.045858 | -1.88493 | 1.50E-62 | 9.88E-62 |
| GATAD2B | 2.210504 | 3.272502 | 1.061998 | 1.91E-66 | 1.89E-65 |
| MRPS34 | 4.962425 | 5.989978 | 1.027553 | 4.51E-57 | 1.97E-56 |
| EFHD2 | 5.017682 | 6.532933 | 1.51525 | 1.23E-62 | 8.16E-62 |
| NUPR1 | 5.470236 | 2.822429 | -2.64781 | 1.37E-67 | 1.54E-66 |
| IGKV1-5 | 4.287426 | 6.810527 | 2.523101 | 2.20E-24 | 3.74E-24 |
| KRTCAP2 | 5.897693 | 3.120566 | -2.77713 | 5.95E-57 | 2.58E-56 |
| ATP5MC1 | 5.963139 | 4.876686 | -1.08645 | 1.01E-50 | 3.25E-50 |
| ABTB1 | 4.143057 | 2.601447 | -1.54161 | 4.89E-64 | 3.78E-63 |
| PEBP4 | 1.833406 | 0.190082 | -1.64332 | 1.28E-78 | 1.30E-76 |
| TRIM14 | 2.086521 | 3.927294 | 1.840773 | 2.76E-68 | 3.40E-67 |
| MSTO1 | 3.406105 | 1.820844 | -1.58526 | 3.01E-48 | 8.91E-48 |
| AC006027.1 | 1.679749 | 0.002008 | -1.67774 | 1.78E-78 | 1.35E-76 |
| GOLGA8A | 4.414309 | 2.029656 | -2.38465 | 3.90E-51 | 1.28E-50 |
| PDZK1IP1 | 3.901619 | 5.652222 | 1.750602 | 2.96E-20 | 4.63E-20 |
| RAB19 | 0.533024 | 1.829964 | 1.29694 | 1.51E-61 | 9.15E-61 |
| MTATP8P1 | 0.901516 | 3.980945 | 3.079429 | 3.31E-65 | 2.88E-64 |
| RPN1 | 5.201126 | 6.614183 | 1.413057 | 1.18E-80 | 2.58E-78 |
| COQ10A | 2.579621 | 1.316096 | -1.26352 | 6.20E-66 | 5.80E-65 |
| NTPCR | 3.227716 | 1.459732 | -1.76798 | 8.62E-65 | 7.23E-64 |
| CORO2A | 1.72794 | 3.713616 | 1.985676 | 1.85E-57 | 8.27E-57 |
| TNFAIP1 | 3.548258 | 4.587056 | 1.038798 | 1.05E-71 | 2.08E-70 |
| FIS1 | 6.084912 | 5.060687 | -1.02422 | 3.09E-67 | 3.34E-66 |
| TLCD1 | 1.99486 | 3.451087 | 1.456227 | 4.97E-46 | 1.38E-45 |
| SGSM2 | 4.378966 | 2.418975 | -1.95999 | 5.52E-70 | 8.59E-69 |
| TGFB3 | 2.979815 | 1.885059 | -1.09476 | 6.93E-35 | 1.46E-34 |
| AGAP3 | 4.19541 | 2.80496 | -1.39045 | 4.57E-58 | 2.12E-57 |
| AL356653.1 | 0.386723 | 1.586907 | 1.200185 | 9.67E-72 | 1.93E-70 |
| EMC4 | 4.85159 | 3.789664 | -1.06193 | 4.56E-68 | 5.48E-67 |
| MIA2 | 3.261539 | 0.957667 | -2.30387 | 2.67E-62 | 1.72E-61 |
| AL163636.2 | 3.17434 | 0.120078 | -3.05426 | 2.74E-74 | 8.47E-73 |
| EEF1B2P3 | 2.319166 | 3.43409 | 1.114924 | 4.70E-51 | 1.54E-50 |
| MARS | 4.994797 | 3.843699 | -1.1511 | 4.25E-53 | 1.50E-52 |
| SNORD63B | 0.220911 | 2.066033 | 1.845122 | 2.13E-66 | 2.10E-65 |
| BRI3BP | 1.655555 | 3.716782 | 2.061227 | 1.50E-73 | 4.08E-72 |
| ICAM1 | 2.859482 | 4.348954 | 1.489471 | 3.93E-44 | 1.03E-43 |
| SNORD6 | 0.176444 | 2.173528 | 1.997084 | 4.49E-70 | 7.07E-69 |
| SIVA1 | 4.955984 | 3.318342 | -1.63764 | 2.81E-66 | 2.72E-65 |
| SNHG1 | 4.803688 | 3.510043 | -1.29365 | 7.11E-43 | 1.80E-42 |
| PTBP2 | 2.626384 | 1.199323 | -1.42706 | 2.22E-59 | 1.14E-58 |
| TPMT | 2.34989 | 4.050541 | 1.700651 | 6.12E-69 | 8.22E-68 |
| MCF2L | 3.414554 | 1.605559 | -1.809 | 1.68E-51 | 5.60E-51 |
| RSAD1 | 3.922552 | 2.704775 | -1.21778 | 6.09E-58 | 2.80E-57 |
| CROCC | 3.218419 | 1.591758 | -1.62666 | 1.32E-61 | 8.03E-61 |
| RPL35P2 | 0.645068 | 2.186568 | 1.541499 | 2.12E-62 | 1.38E-61 |
| GSTA1 | 4.232827 | 3.139077 | -1.09375 | 1.68E-07 | 2.06E-07 |
| SMIM11A | 2.187343 | 0.022002 | -2.16534 | 7.55E-76 | 3.08E-74 |
| KRT19 | 5.873601 | 8.506384 | 2.632783 | 7.02E-38 | 1.57E-37 |
| SLC6A14 | 0.511969 | 2.693545 | 2.181577 | 1.40E-53 | 5.07E-53 |
| MEIS1 | 3.303437 | 1.488638 | -1.8148 | 1.41E-50 | 4.51E-50 |
| CXADR | 2.328646 | 3.626366 | 1.29772 | 5.16E-34 | 1.07E-33 |
| RBM17 | 4.851982 | 3.72099 | -1.13099 | 2.69E-48 | 7.99E-48 |
| SRGN | 4.625654 | 6.34856 | 1.722906 | 2.06E-44 | 5.45E-44 |
| ILDR1 | 0.319537 | 2.225035 | 1.905497 | 1.75E-77 | 1.02E-75 |
| LIN37 | 2.413989 | 0.738686 | -1.6753 | 2.18E-73 | 5.76E-72 |
| OSBPL8 | 2.156666 | 3.274182 | 1.117516 | 2.11E-63 | 1.53E-62 |
| NR2F6 | 4.136334 | 5.18683 | 1.050496 | 3.84E-42 | 9.54E-42 |
| TRBJ2-2P | 0.324636 | 1.433721 | 1.109084 | 4.32E-30 | 8.22E-30 |
| ANKEF1 | 1.045039 | 2.192622 | 1.147583 | 1.53E-65 | 1.38E-64 |
| URB2 | 1.191634 | 2.257331 | 1.065697 | 1.96E-71 | 3.72E-70 |
| AC093724.1 | 0.523376 | 1.680609 | 1.157233 | 3.97E-77 | 2.14E-75 |
| CDK3 | 2.074026 | 0.190796 | -1.88323 | 5.46E-56 | 2.24E-55 |
| AC020978.5 | 2.372291 | 0.002441 | -2.36985 | 1.75E-77 | 1.02E-75 |
| SYNJ2BP-COX16 | 1.491818 | 0.162501 | -1.32932 | 7.18E-72 | 1.47E-70 |
| AP001324.1 | 2.480687 | 5.076853 | 2.596165 | 2.15E-61 | 1.29E-60 |
| YBX1P1 | 1.375523 | 2.530637 | 1.155114 | 1.43E-60 | 8.01E-60 |
| TMEM238 | 1.997058 | 5.383363 | 3.386305 | 5.80E-66 | 5.44E-65 |
| UNC93B1 | 3.371179 | 4.799532 | 1.428354 | 1.99E-62 | 1.30E-61 |
| MYD88 | 3.507388 | 4.809464 | 1.302076 | 8.63E-60 | 4.54E-59 |
| SYNGR1 | 2.912261 | 1.190995 | -1.72127 | 2.33E-66 | 2.28E-65 |
| BPIFB1 | 2.323038 | 3.396466 | 1.073428 | 0.000131 | 0.00015 |
| TMCO3 | 3.729462 | 2.567779 | -1.16168 | 1.07E-59 | 5.61E-59 |
| NIT2 | 3.575799 | 2.356846 | -1.21895 | 4.95E-50 | 1.55E-49 |
| ACSS1 | 4.314041 | 3.146998 | -1.16704 | 5.88E-35 | 1.24E-34 |
| MBD3 | 4.123441 | 2.810603 | -1.31284 | 5.36E-64 | 4.12E-63 |
| OLFM1 | 2.443089 | 1.160077 | -1.28301 | 9.40E-47 | 2.66E-46 |
| CCNL2 | 5.828749 | 3.603937 | -2.22481 | 3.35E-51 | 1.10E-50 |
| PACS2 | 4.129212 | 2.903192 | -1.22602 | 2.59E-61 | 1.53E-60 |
| AC092651.2 | 0.370502 | 1.773443 | 1.402941 | 1.62E-45 | 4.41E-45 |
| NCAM1 | 3.09374 | 0.611912 | -2.48183 | 4.11E-73 | 1.03E-71 |
| LTBP4 | 5.970396 | 4.774832 | -1.19556 | 4.19E-41 | 1.01E-40 |
| ESRRG | 2.328053 | 0.266545 | -2.06151 | 4.39E-65 | 3.78E-64 |
| RNU1-27P | 1.675992 | 0.270646 | -1.40535 | 1.74E-58 | 8.27E-58 |
| MCM2 | 1.596744 | 3.85405 | 2.257306 | 2.65E-80 | 4.89E-78 |
| COMP | 0.840836 | 1.948787 | 1.107951 | 5.82E-13 | 7.99E-13 |
| MTERF2 | 3.005081 | 1.500886 | -1.50419 | 1.37E-66 | 1.38E-65 |
| APOL6 | 1.914603 | 4.155674 | 2.241071 | 8.33E-70 | 1.26E-68 |
| PGRMC1 | 4.845607 | 6.157323 | 1.311716 | 3.52E-67 | 3.77E-66 |
| AC124068.1 | 3.92754 | 0.002008 | -3.92553 | 1.78E-78 | 1.35E-76 |
| TMSB4XP4 | 1.790843 | 4.181951 | 2.391108 | 1.31E-59 | 6.81E-59 |
| ARFIP2 | 4.643673 | 3.483837 | -1.15984 | 1.80E-57 | 8.03E-57 |
| ZNF888 | 0.540073 | 1.764188 | 1.224115 | 2.38E-66 | 2.33E-65 |
| RPS4XP11 | 0.416367 | 1.490312 | 1.073946 | 2.86E-61 | 1.68E-60 |
| DNAJA1 | 4.782216 | 5.971773 | 1.189557 | 7.84E-62 | 4.87E-61 |
| MIR4653 | 0.209829 | 2.132107 | 1.922278 | 1.29E-68 | 1.67E-67 |
| SMYD3 | 2.036356 | 0.87817 | -1.15819 | 1.60E-53 | 5.81E-53 |
| TLE2 | 3.793335 | 2.670303 | -1.12303 | 6.94E-37 | 1.53E-36 |
| CUTALP | 2.975073 | 1.909709 | -1.06536 | 2.62E-42 | 6.54E-42 |
| VCPIP1 | 1.335422 | 2.470607 | 1.135184 | 3.78E-79 | 5.08E-77 |
| TUT1 | 2.832181 | 1.657846 | -1.17434 | 3.98E-51 | 1.30E-50 |
| LARP6 | 3.167741 | 1.153735 | -2.01401 | 5.75E-72 | 1.19E-70 |
| TPM2 | 7.679433 | 5.256731 | -2.4227 | 3.03E-43 | 7.73E-43 |
| KIF18A | 0.404011 | 1.880601 | 1.47659 | 2.37E-77 | 1.35E-75 |
| STIMATE | 2.225686 | 0.516915 | -1.70877 | 5.59E-67 | 5.83E-66 |
| TXN | 6.500211 | 7.952336 | 1.452126 | 1.84E-58 | 8.75E-58 |
| GPAT4 | 4.261718 | 3.197145 | -1.06457 | 3.70E-48 | 1.09E-47 |
| SMN1 | 2.838762 | 1.119207 | -1.71956 | 3.32E-53 | 1.18E-52 |
| VKORC1 | 4.844792 | 3.471573 | -1.37322 | 5.08E-62 | 3.20E-61 |
| DCTN3 | 4.84983 | 3.287906 | -1.56192 | 4.98E-78 | 3.35E-76 |
| PSMC1 | 4.754265 | 2.561484 | -2.19278 | 1.77E-63 | 1.29E-62 |
| STXBP2 | 4.86128 | 3.264475 | -1.59681 | 3.27E-28 | 5.98E-28 |
| PLA2G7 | 0.551044 | 2.855455 | 2.30441 | 1.22E-78 | 1.25E-76 |
| TXNDC12 | 3.598674 | 4.893835 | 1.29516 | 2.97E-80 | 5.31E-78 |
| MIR3671 | 0.233315 | 1.622359 | 1.389045 | 3.77E-59 | 1.89E-58 |
| GTF2H4 | 2.97321 | 0.667358 | -2.30585 | 4.52E-55 | 1.76E-54 |
| WASH7P | 2.580273 | 0.686 | -1.89427 | 1.71E-56 | 7.25E-56 |
| STK33 | 1.920861 | 0.521229 | -1.39963 | 6.31E-68 | 7.43E-67 |
| MPI | 3.335321 | 2.235711 | -1.09961 | 9.35E-75 | 3.17E-73 |
| FASTKD5 | 2.203306 | 3.267662 | 1.064355 | 1.17E-67 | 1.32E-66 |
| CRELD1 | 4.154156 | 2.824358 | -1.3298 | 5.12E-66 | 4.83E-65 |
| GJB3 | 0.983872 | 3.155032 | 2.17116 | 2.93E-53 | 1.05E-52 |
| HIP1R | 4.865068 | 3.628434 | -1.23663 | 3.76E-16 | 5.47E-16 |
| CHKB | 4.39968 | 1.059857 | -3.33982 | 5.21E-57 | 2.27E-56 |
| AL356535.1 | 0.366815 | 2.122781 | 1.755965 | 6.98E-63 | 4.77E-62 |
| LINC01578 | 4.595897 | 2.992028 | -1.60387 | 1.24E-60 | 6.93E-60 |
| ANO1 | 2.905835 | 4.025268 | 1.119433 | 4.26E-23 | 7.07E-23 |
| MIR559 | 0.070927 | 1.353546 | 1.28262 | 6.20E-56 | 2.53E-55 |
| ESPN | 1.131598 | 2.492094 | 1.360497 | 9.55E-31 | 1.84E-30 |
| LINC00869 | 2.610163 | 0.691235 | -1.91893 | 3.77E-64 | 2.96E-63 |
| AGER | 2.228676 | 1.189594 | -1.03908 | 2.69E-55 | 1.06E-54 |
| FRY | 3.009215 | 1.193654 | -1.81556 | 4.76E-67 | 5.01E-66 |
| AC016026.1 | 1.976044 | 0.059021 | -1.91702 | 1.72E-72 | 3.90E-71 |
| SCNN1B | 2.93646 | 0.517479 | -2.41898 | 3.22E-69 | 4.49E-68 |
| OGFOD2 | 2.616304 | 0.46043 | -2.15587 | 2.60E-66 | 2.53E-65 |
| OSTF1 | 3.63443 | 4.729975 | 1.095544 | 9.27E-59 | 4.51E-58 |
| SNHG14 | 3.339887 | 0.412694 | -2.92719 | 8.90E-80 | 1.37E-77 |
| NDUFS7 | 5.170656 | 2.776127 | -2.39453 | 1.06E-78 | 1.13E-76 |
| ADAM33 | 3.939994 | 1.03914 | -2.90085 | 5.22E-68 | 6.22E-67 |
| OR2A1-AS1 | 2.039245 | 0.664589 | -1.37466 | 1.13E-58 | 5.46E-58 |
| DCLRE1B | 0.956676 | 2.191196 | 1.23452 | 4.56E-81 | 1.14E-78 |
| BTBD1 | 3.208688 | 4.331458 | 1.122769 | 5.17E-73 | 1.28E-71 |
| MSH5-SAPCD1 | 1.533069 | 0.191089 | -1.34198 | 3.92E-54 | 1.46E-53 |
| HIST1H4C | 0.451719 | 1.946261 | 1.494542 | 1.92E-61 | 1.15E-60 |
| SORBS1 | 5.139633 | 3.118337 | -2.0213 | 8.85E-42 | 2.18E-41 |
| VMA21 | 2.056802 | 3.641601 | 1.584799 | 2.51E-80 | 4.68E-78 |
| AL080243.2 | 1.143301 | 2.597759 | 1.454458 | 1.12E-59 | 5.82E-59 |
| AL008729.2 | 1.498268 | 0.086869 | -1.4114 | 1.86E-62 | 1.22E-61 |
| EFNB2 | 2.797672 | 4.491089 | 1.693417 | 1.43E-63 | 1.05E-62 |
| CRABP2 | 1.762119 | 3.20848 | 1.446361 | 5.28E-25 | 9.09E-25 |
| RPS3AP26 | 2.216522 | 3.465482 | 1.248961 | 1.47E-51 | 4.92E-51 |
| DMBT1 | 0.8342 | 4.29767 | 3.46347 | 1.07E-45 | 2.94E-45 |
| FDPS | 5.44894 | 4.340028 | -1.10891 | 1.35E-52 | 4.70E-52 |
| THBS3 | 3.529774 | 2.449566 | -1.08021 | 2.95E-49 | 9.01E-49 |
| SNORA70 | 1.554037 | 0.002008 | -1.55203 | 1.90E-78 | 1.40E-76 |
| CKB | 8.122909 | 4.652936 | -3.46997 | 1.79E-69 | 2.58E-68 |
| HMGA1P2 | 0.758165 | 1.768291 | 1.010127 | 1.40E-63 | 1.03E-62 |
| CBWD6 | 1.9617 | 0.267956 | -1.69374 | 6.55E-53 | 2.30E-52 |
| ADHFE1 | 3.945558 | 0.457161 | -3.4884 | 1.12E-81 | 3.47E-79 |
| HLA-C | 7.309074 | 8.82925 | 1.520176 | 1.30E-58 | 6.27E-58 |
| ITGA8 | 2.604603 | 1.454466 | -1.15014 | 9.55E-33 | 1.92E-32 |
| IL17RB | 1.823523 | 3.073498 | 1.249975 | 9.22E-47 | 2.61E-46 |
| OLFM4 | 1.794583 | 6.582083 | 4.7875 | 1.95E-44 | 5.16E-44 |
| ACADL | 1.769939 | 0.114889 | -1.65505 | 9.84E-82 | 3.20E-79 |
| MPPE1 | 3.529383 | 1.472357 | -2.05703 | 2.61E-65 | 2.30E-64 |
| CDC45 | 0.924304 | 2.529152 | 1.604848 | 4.01E-64 | 3.13E-63 |
| WASF2 | 4.293879 | 5.370535 | 1.076656 | 8.54E-53 | 2.99E-52 |
| ROBO3 | 2.244507 | 0.601492 | -1.64302 | 6.20E-65 | 5.27E-64 |
| OST4 | 6.773395 | 8.15227 | 1.378875 | 1.78E-62 | 1.17E-61 |
| RNU6-437P | 0.144266 | 1.265687 | 1.121421 | 5.62E-39 | 1.29E-38 |
| COX8A | 7.505667 | 8.700799 | 1.195133 | 2.69E-61 | 1.59E-60 |
| AC133435.1 | 0.371976 | 1.504652 | 1.132676 | 1.96E-69 | 2.81E-68 |
| MAP3K12 | 2.704761 | 1.197981 | -1.50678 | 5.73E-61 | 3.30E-60 |
| CEACAM1 | 2.065477 | 3.992171 | 1.926695 | 1.27E-50 | 4.07E-50 |
| DNAH14 | 1.309799 | 0.303384 | -1.00641 | 2.08E-52 | 7.19E-52 |
| NDRG2 | 5.636609 | 2.798818 | -2.83779 | 3.41E-82 | 1.37E-79 |
| CDCA4 | 1.607188 | 3.32172 | 1.714532 | 1.98E-75 | 7.46E-74 |
| POLH | 1.558723 | 2.800661 | 1.241939 | 1.91E-69 | 2.74E-68 |
| MALAT1 | 6.624304 | 8.327028 | 1.702723 | 5.80E-36 | 1.25E-35 |
| HKR1 | 3.285079 | 2.167773 | -1.11731 | 2.36E-55 | 9.36E-55 |
| LCLAT1 | 1.318841 | 2.35194 | 1.033099 | 1.81E-74 | 5.75E-73 |
| LPGAT1 | 2.047018 | 3.478401 | 1.431383 | 2.97E-72 | 6.51E-71 |
| KRT7 | 2.293203 | 3.995994 | 1.702791 | 3.07E-16 | 4.47E-16 |
| RTP4 | 1.617423 | 3.010151 | 1.392728 | 9.11E-51 | 2.94E-50 |
| MARCKS | 3.979903 | 5.497616 | 1.517713 | 4.80E-59 | 2.39E-58 |
| AL033519.2 | 0.991034 | 3.170986 | 2.179952 | 4.69E-60 | 2.52E-59 |
| COMMD3-BMI1 | 2.225447 | 0.01651 | -2.20894 | 2.49E-68 | 3.09E-67 |
| SNORA77 | 0.421298 | 1.737927 | 1.316629 | 1.32E-50 | 4.23E-50 |
| CHRAC1 | 3.104843 | 4.41569 | 1.310847 | 2.33E-72 | 5.21E-71 |
| OR7E14P | 1.731254 | 2.949547 | 1.218293 | 1.14E-36 | 2.50E-36 |
| MYADM | 4.827881 | 6.123317 | 1.295437 | 3.29E-29 | 6.15E-29 |
| IER5 | 2.144012 | 3.238117 | 1.094106 | 2.22E-45 | 6.03E-45 |
| MRPS24 | 5.177605 | 2.401073 | -2.77653 | 3.99E-67 | 4.25E-66 |
| KCNE2 | 5.13185 | 1.016093 | -4.11576 | 8.64E-53 | 3.03E-52 |
| RN7SL4P | 0.561511 | 2.677497 | 2.115986 | 3.56E-59 | 1.79E-58 |
| RGMA | 3.894836 | 1.589867 | -2.30497 | 4.93E-47 | 1.40E-46 |
| PPIAP22 | 4.291555 | 5.419381 | 1.127827 | 2.13E-62 | 1.39E-61 |
| MAPKAPK5 | 3.08343 | 1.987314 | -1.09612 | 3.13E-50 | 9.89E-50 |
| RPL24P4 | 2.438069 | 3.875431 | 1.437363 | 3.21E-55 | 1.26E-54 |
| FUNDC2 | 5.008776 | 2.405941 | -2.60283 | 7.47E-74 | 2.14E-72 |
| TMED7 | 3.656248 | 4.786534 | 1.130285 | 5.43E-64 | 4.17E-63 |
| PNPLA7 | 2.962793 | 0.741431 | -2.22136 | 1.42E-73 | 3.89E-72 |
| AC091153.1 | 0.660233 | 2.038451 | 1.378217 | 3.99E-67 | 4.25E-66 |
| LY6G5B | 2.664945 | 0.702518 | -1.96243 | 8.91E-54 | 3.26E-53 |
| ZNF585A | 2.161976 | 0.801105 | -1.36087 | 2.20E-67 | 2.40E-66 |
| AC037198.2 | 1.893345 | 0.598752 | -1.29459 | 2.47E-36 | 5.37E-36 |
| ATE1 | 2.045517 | 3.339527 | 1.294011 | 1.97E-66 | 1.95E-65 |
| BIRC5 | 1.238034 | 3.265955 | 2.02792 | 8.94E-64 | 6.71E-63 |
| USP9Y | 1.699282 | 0.698937 | -1.00034 | 2.25E-13 | 3.11E-13 |
| EPOR | 2.614133 | 1.314032 | -1.3001 | 3.67E-46 | 1.02E-45 |
| GORASP1 | 4.389243 | 3.10865 | -1.28059 | 7.71E-70 | 1.17E-68 |
| LZTR1 | 4.023399 | 2.850971 | -1.17243 | 2.21E-57 | 9.80E-57 |
| ALKBH6 | 2.787006 | 0.90018 | -1.88683 | 5.31E-58 | 2.45E-57 |
| RPS7P3 | 0.492606 | 1.851018 | 1.358412 | 4.12E-64 | 3.21E-63 |
| CMC4 | 2.069035 | 0.057007 | -2.01203 | 1.18E-76 | 5.64E-75 |
| CRLF1 | 2.187074 | 0.816318 | -1.37076 | 1.34E-50 | 4.29E-50 |
| ITGA9-AS1 | 1.390551 | 0.376866 | -1.01369 | 4.39E-71 | 7.94E-70 |
| ZNF703 | 3.218573 | 5.509677 | 2.291104 | 1.33E-53 | 4.83E-53 |
| LGR5 | 0.62139 | 1.989494 | 1.368105 | 3.03E-30 | 5.79E-30 |
| APLN | 0.619077 | 1.738645 | 1.119567 | 2.85E-38 | 6.45E-38 |
| STAB1 | 4.421381 | 2.743775 | -1.67761 | 5.49E-54 | 2.03E-53 |
| TUBB | 6.110316 | 7.982298 | 1.871982 | 1.21E-84 | 3.62E-81 |
| AC006483.1 | 0.215449 | 1.765659 | 1.550209 | 8.70E-73 | 2.08E-71 |
| AC114491.1 | 1.147084 | 2.197692 | 1.050609 | 8.22E-46 | 2.26E-45 |
| NFE2L3 | 2.218988 | 4.135383 | 1.916395 | 1.30E-76 | 6.16E-75 |
| PAAF1 | 2.809817 | 1.619092 | -1.19072 | 3.85E-65 | 3.32E-64 |
| SURF4 | 5.289509 | 6.708604 | 1.419095 | 1.91E-74 | 6.01E-73 |
| FKBP1A | 5.791311 | 6.945306 | 1.153995 | 2.82E-78 | 2.01E-76 |
| COX17P1 | 0.306812 | 1.874979 | 1.568167 | 2.62E-65 | 2.31E-64 |
| MND1 | 0.739612 | 1.906976 | 1.167364 | 9.09E-61 | 5.16E-60 |
| CRIPT | 2.164427 | 3.311994 | 1.147568 | 1.17E-63 | 8.70E-63 |
| RPS20P2 | 0.236315 | 1.251804 | 1.015489 | 2.70E-59 | 1.37E-58 |
| ACY1 | 3.076983 | 1.708599 | -1.36838 | 7.68E-48 | 2.24E-47 |
| CCDC12 | 4.012155 | 2.702919 | -1.30924 | 1.18E-64 | 9.75E-64 |
| USP1 | 2.637367 | 3.781943 | 1.144575 | 1.05E-77 | 6.60E-76 |
| ADAMTS2 | 2.043711 | 3.161659 | 1.117947 | 5.81E-29 | 1.08E-28 |
| SOCS2 | 3.377203 | 1.663838 | -1.71337 | 5.77E-72 | 1.19E-70 |
| TICRR | 0.606021 | 1.640273 | 1.034252 | 8.19E-56 | 3.33E-55 |
| LRRC8B | 1.205273 | 2.530662 | 1.32539 | 2.88E-78 | 2.03E-76 |
| SETD4 | 2.494787 | 1.30928 | -1.18551 | 1.84E-61 | 1.11E-60 |
| HOXC8 | 0.105994 | 1.11544 | 1.009446 | 4.44E-64 | 3.45E-63 |
| DNAJC15 | 2.433312 | 3.512082 | 1.07877 | 2.34E-53 | 8.38E-53 |
| CEACAM5 | 1.978714 | 6.681932 | 4.703218 | 1.32E-53 | 4.80E-53 |
| RPL32P29 | 2.853295 | 0.002008 | -2.85129 | 1.78E-78 | 1.35E-76 |
| LINC01123 | 1.925384 | 0.77773 | -1.14765 | 2.51E-22 | 4.11E-22 |
| PAQR8 | 2.662325 | 3.7006 | 1.038275 | 1.66E-26 | 2.93E-26 |
| MRPL3 | 4.204681 | 5.435562 | 1.230881 | 6.64E-82 | 2.25E-79 |
| DAAM2 | 3.086012 | 1.807154 | -1.27886 | 2.93E-40 | 6.96E-40 |
| GOLIM4 | 3.198352 | 4.533307 | 1.334955 | 1.79E-64 | 1.45E-63 |
| WNT5A | 1.11931 | 2.412769 | 1.293459 | 5.18E-58 | 2.39E-57 |
| PSMA6 | 4.737713 | 1.75888 | -2.97883 | 4.62E-63 | 3.21E-62 |
| FBXL14 | 2.30134 | 3.755411 | 1.454071 | 1.77E-60 | 9.84E-60 |
| STK35 | 2.366261 | 3.507199 | 1.140938 | 6.25E-72 | 1.29E-70 |
| PANX1 | 2.014225 | 3.236428 | 1.222203 | 4.53E-67 | 4.79E-66 |
| GTF2H2B | 1.515147 | 0.235558 | -1.27959 | 3.15E-61 | 1.85E-60 |
| S100A16 | 5.431412 | 6.929506 | 1.498094 | 3.50E-45 | 9.43E-45 |
| IGHV4-39 | 3.307662 | 5.949805 | 2.642143 | 1.05E-27 | 1.90E-27 |
| DEXI | 3.583148 | 1.346288 | -2.23686 | 3.16E-73 | 8.14E-72 |
| CMPK1 | 4.682311 | 6.126007 | 1.443696 | 8.00E-71 | 1.40E-69 |
| CASTOR2 | 0.960655 | 2.263068 | 1.302413 | 1.13E-55 | 4.57E-55 |
| UBXN6 | 5.182252 | 3.625639 | -1.55661 | 4.99E-80 | 8.44E-78 |
| NPDC1 | 5.79581 | 4.764103 | -1.03171 | 1.83E-36 | 3.98E-36 |
| MTND3P25 | 0.355136 | 1.898318 | 1.543182 | 4.55E-57 | 1.99E-56 |
| SPRED1 | 1.823699 | 3.026393 | 1.202694 | 3.48E-69 | 4.81E-68 |
| YDJC | 2.95615 | 4.449634 | 1.493484 | 3.93E-71 | 7.18E-70 |
| SLC1A5 | 4.309576 | 5.432747 | 1.123171 | 6.63E-42 | 1.64E-41 |
| DENR | 3.22774 | 4.428808 | 1.201068 | 4.74E-79 | 6.06E-77 |
| SPEG | 3.809747 | 0.918641 | -2.89111 | 4.10E-62 | 2.61E-61 |
| MRPL47 | 3.587033 | 4.913527 | 1.326494 | 4.81E-79 | 6.11E-77 |
| AC099789.1 | 0.262973 | 1.673993 | 1.41102 | 1.19E-61 | 7.24E-61 |
| H1FX-AS1 | 1.407418 | 0.28087 | -1.12655 | 4.94E-68 | 5.91E-67 |
| ADD1 | 5.554939 | 4.124607 | -1.43033 | 7.71E-70 | 1.17E-68 |
| STK16 | 3.712139 | 2.666146 | -1.04599 | 6.43E-69 | 8.60E-68 |
| TNFSF15 | 0.731674 | 1.868825 | 1.137151 | 9.00E-59 | 4.38E-58 |
| SGSM3 | 5.733767 | 3.807214 | -1.92655 | 1.84E-63 | 1.34E-62 |
| MT1G | 6.656977 | 4.633039 | -2.02394 | 3.42E-22 | 5.58E-22 |
| MPEG1 | 1.773497 | 3.367028 | 1.593531 | 4.89E-43 | 1.24E-42 |
| AL355312.3 | 0.642906 | 1.932202 | 1.289296 | 1.62E-58 | 7.74E-58 |
| C1QC | 4.490076 | 6.697114 | 2.207038 | 1.32E-51 | 4.42E-51 |
| RPL21P16 | 7.431609 | 4.143061 | -3.28855 | 2.17E-62 | 1.41E-61 |
| TGFA | 1.231445 | 2.44162 | 1.210175 | 9.10E-41 | 2.18E-40 |
| DM1-AS | 1.847517 | 0.821206 | -1.02631 | 2.90E-60 | 1.59E-59 |
| RNF19B | 2.763189 | 4.42007 | 1.656881 | 1.37E-65 | 1.24E-64 |
| RPL26P6 | 0.928982 | 2.651497 | 1.722515 | 1.94E-59 | 9.99E-59 |
| HMGB3 | 2.250627 | 3.611535 | 1.360908 | 4.45E-56 | 1.84E-55 |
| OIP5 | 0.707059 | 2.262159 | 1.5551 | 9.47E-70 | 1.42E-68 |
| RPS3 | 8.731501 | 7.656838 | -1.07466 | 5.23E-49 | 1.59E-48 |
| NAA10 | 4.732359 | 3.013993 | -1.71837 | 3.24E-50 | 1.02E-49 |
| EPSTI1 | 1.531773 | 3.333609 | 1.801836 | 4.56E-64 | 3.54E-63 |
| NXT2 | 1.504677 | 2.869373 | 1.364695 | 8.89E-72 | 1.79E-70 |
| AC026464.4 | 3.180392 | 0.023354 | -3.15704 | 6.31E-67 | 6.56E-66 |
| GALNT1 | 3.027167 | 4.418823 | 1.391656 | 3.75E-65 | 3.24E-64 |
| ZNF526 | 1.415635 | 2.497081 | 1.081446 | 9.62E-72 | 1.93E-70 |
| ISOC1 | 2.907589 | 4.08909 | 1.181501 | 9.71E-55 | 3.73E-54 |
| FAM20A | 3.136684 | 1.21277 | -1.92391 | 1.83E-58 | 8.72E-58 |
| CCL24 | 0.792105 | 2.216156 | 1.424051 | 1.79E-35 | 3.82E-35 |
| RPL39P36 | 0.339745 | 1.404712 | 1.064967 | 3.26E-53 | 1.16E-52 |
| ANXA13 | 0.642882 | 2.152359 | 1.509478 | 1.12E-28 | 2.07E-28 |
| GPR153 | 1.526696 | 2.83857 | 1.311874 | 6.96E-41 | 1.67E-40 |
| CR2 | 0.436781 | 1.465034 | 1.028253 | 1.65E-17 | 2.47E-17 |
| CYB5A | 5.700786 | 2.731497 | -2.96929 | 1.28E-73 | 3.54E-72 |
| KRT18 | 5.76765 | 8.205275 | 2.437625 | 1.18E-50 | 3.80E-50 |
| DCUN1D4 | 3.259851 | 2.170854 | -1.089 | 2.42E-63 | 1.74E-62 |
| SH3KBP1 | 2.334095 | 3.892542 | 1.558447 | 6.34E-72 | 1.30E-70 |
| CCDC149 | 2.623206 | 1.229111 | -1.3941 | 9.26E-70 | 1.39E-68 |
| SNX1 | 4.599323 | 3.356539 | -1.24278 | 1.44E-75 | 5.52E-74 |
| RNU6-8 | 0.52946 | 2.837614 | 2.308153 | 1.25E-59 | 6.51E-59 |
| RPS26P3 | 0.377932 | 1.570009 | 1.192077 | 4.71E-61 | 2.73E-60 |
| PSAT1 | 2.810583 | 4.072809 | 1.262226 | 2.74E-30 | 5.24E-30 |
| MTCH2 | 3.949681 | 5.070859 | 1.121178 | 6.15E-69 | 8.24E-68 |
| CYP2C8 | 1.707945 | 0.250196 | -1.45775 | 1.89E-48 | 5.62E-48 |
| SMIM19 | 4.022618 | 2.697854 | -1.32476 | 1.56E-65 | 1.41E-64 |
| LRRC75B | 3.142984 | 2.019361 | -1.12362 | 2.35E-51 | 7.77E-51 |
| RN7SL128P | 0.268 | 1.466617 | 1.198616 | 1.89E-56 | 7.98E-56 |
| ANXA10 | 5.134545 | 3.708515 | -1.42603 | 4.20E-10 | 5.44E-10 |
| SINHCAF | 2.733124 | 3.849608 | 1.116483 | 1.70E-58 | 8.11E-58 |
| PYCARD | 3.074086 | 4.478809 | 1.404724 | 1.48E-52 | 5.14E-52 |
| FGG | 1.569859 | 0.365316 | -1.20454 | 1.23E-47 | 3.57E-47 |
| ARIH1 | 3.501475 | 2.257931 | -1.24354 | 1.60E-58 | 7.64E-58 |
| AL355312.4 | 0.723165 | 2.186086 | 1.462921 | 1.63E-37 | 3.62E-37 |
| CDH19 | 1.338189 | 0.336845 | -1.00134 | 1.06E-52 | 3.71E-52 |
| MT-TL1 | 0.510424 | 2.77834 | 2.267916 | 2.75E-57 | 1.21E-56 |
| CNBD2 | 1.216611 | 0.120417 | -1.09619 | 5.28E-57 | 2.29E-56 |
| COL2A1 | 1.864241 | 0.328742 | -1.5355 | 7.57E-39 | 1.74E-38 |
| CLN6 | 2.943403 | 4.059039 | 1.115636 | 1.60E-68 | 2.04E-67 |
| CD74 | 7.673431 | 9.822315 | 2.148884 | 7.97E-55 | 3.08E-54 |
| OLFM2 | 1.219961 | 2.340972 | 1.121011 | 6.00E-38 | 1.35E-37 |
| MT-RNR1 | 9.949306 | 11.12265 | 1.173343 | 2.25E-33 | 4.59E-33 |
| PIM2 | 2.935491 | 3.938967 | 1.003476 | 1.60E-25 | 2.79E-25 |
| CCDC88B | 2.762682 | 1.653944 | -1.10874 | 2.24E-44 | 5.90E-44 |
| TTC14 | 3.369208 | 1.889895 | -1.47931 | 5.39E-59 | 2.68E-58 |
| ZNF224 | 2.589347 | 1.134283 | -1.45506 | 2.96E-50 | 9.37E-50 |
| TWISTNB | 1.82944 | 3.350407 | 1.520967 | 8.24E-78 | 5.23E-76 |
| RPSAP19 | 0.428334 | 1.83881 | 1.410476 | 3.27E-69 | 4.54E-68 |
| TKFC | 3.88682 | 2.672232 | -1.21459 | 2.48E-46 | 6.92E-46 |
| PLLP | 4.173681 | 2.578679 | -1.595 | 3.42E-26 | 6.02E-26 |
| IGHA1 | 8.313594 | 9.368937 | 1.055344 | 0.017683 | 0.018903 |
| PABPC1P3 | 0.782589 | 2.040791 | 1.258203 | 5.12E-66 | 4.83E-65 |
| ATP5MDP1 | 0.398915 | 1.591027 | 1.192112 | 1.40E-48 | 4.19E-48 |
| ARMCX4 | 2.197597 | 0.555041 | -1.64256 | 2.36E-68 | 2.94E-67 |
| ODC1 | 4.345632 | 5.683348 | 1.337716 | 1.14E-47 | 3.30E-47 |
| SLC38A1 | 3.143019 | 4.196212 | 1.053193 | 8.61E-28 | 1.56E-27 |
| PTPRS | 3.427478 | 2.236388 | -1.19109 | 6.65E-37 | 1.47E-36 |
| DNAJC3 | 3.085915 | 4.390672 | 1.304756 | 6.36E-72 | 1.31E-70 |
| SLC52A2 | 3.595799 | 4.810728 | 1.214929 | 6.95E-63 | 4.75E-62 |
| LINC00663 | 1.397307 | 0.322878 | -1.07443 | 5.88E-71 | 1.04E-69 |
| SLC2A11 | 2.381401 | 0.746692 | -1.63471 | 2.65E-66 | 2.58E-65 |
| CREG1 | 4.755311 | 6.003211 | 1.2479 | 1.38E-68 | 1.77E-67 |
| AC005523.2 | 2.1107 | 0.002008 | -2.10869 | 1.78E-78 | 1.35E-76 |
| SEM1 | 5.129575 | 3.21714 | -1.91244 | 4.37E-51 | 1.43E-50 |
| TMC5 | 2.961499 | 4.725022 | 1.763523 | 6.16E-32 | 1.22E-31 |
| IGHV4-55 | 1.17517 | 2.275345 | 1.100174 | 1.28E-22 | 2.10E-22 |
| DNAJC25-GNG10 | 1.772331 | 0.012471 | -1.75986 | 2.75E-70 | 4.46E-69 |
| AC146944.4 | 2.00688 | 0.064045 | -1.94284 | 2.24E-69 | 3.19E-68 |
| PDE2A | 2.886824 | 1.043475 | -1.84335 | 1.51E-70 | 2.54E-69 |
| NOTCH1 | 2.477957 | 4.022346 | 1.544389 | 7.29E-55 | 2.82E-54 |
| IGHV3-23 | 4.163561 | 6.708169 | 2.544608 | 1.27E-25 | 2.22E-25 |
| ABTB2 | 1.319714 | 2.551198 | 1.231484 | 4.26E-53 | 1.51E-52 |
| AL049872.1 | 0.253279 | 1.604367 | 1.351087 | 9.23E-72 | 1.86E-70 |
| PUM1 | 3.427899 | 4.719876 | 1.291977 | 2.50E-76 | 1.12E-74 |
| FERMT2 | 3.948915 | 2.726021 | -1.22289 | 8.25E-28 | 1.50E-27 |
| FUT4 | 1.704454 | 3.263624 | 1.55917 | 4.68E-62 | 2.96E-61 |
| RBM47 | 3.496461 | 4.865818 | 1.369357 | 1.25E-32 | 2.52E-32 |
| NKTR | 3.937909 | 2.257388 | -1.68052 | 1.23E-53 | 4.49E-53 |
| COL1A1 | 5.506874 | 8.529548 | 3.022674 | 1.28E-69 | 1.88E-68 |
| PHC1 | 3.246566 | 1.395593 | -1.85097 | 2.98E-70 | 4.82E-69 |
| LINC01082 | 2.063536 | 1.036508 | -1.02703 | 2.07E-36 | 4.51E-36 |
| PDHB | 4.394148 | 3.331755 | -1.06239 | 1.39E-70 | 2.35E-69 |
| DCAF8 | 5.193766 | 2.977303 | -2.21646 | 4.82E-62 | 3.04E-61 |
| C15orf48 | 3.130699 | 5.005997 | 1.875298 | 2.14E-25 | 3.71E-25 |
| ZC3H14 | 3.366118 | 1.74286 | -1.62326 | 3.22E-60 | 1.75E-59 |
| CENPM | 1.423487 | 2.913874 | 1.490387 | 8.25E-47 | 2.33E-46 |
| SLX1A | 3.452535 | 0.053008 | -3.39953 | 3.25E-65 | 2.84E-64 |
| IER3IP1 | 2.611558 | 3.763149 | 1.151592 | 3.19E-66 | 3.07E-65 |
| AC005944.1 | 2.356807 | 0.002008 | -2.3548 | 1.80E-78 | 1.35E-76 |
| EFNB1 | 2.954396 | 4.729777 | 1.775381 | 4.26E-63 | 2.98E-62 |
| SPAG5 | 1.706682 | 2.923029 | 1.216347 | 2.82E-47 | 8.09E-47 |
| PFDN5 | 7.409132 | 5.468291 | -1.94084 | 1.96E-68 | 2.47E-67 |
| ITPRIPL2 | 2.174477 | 3.359823 | 1.185346 | 3.49E-60 | 1.89E-59 |
| PPIAP31 | 0.830924 | 1.98027 | 1.149346 | 1.21E-66 | 1.23E-65 |
| AGAP9 | 2.689652 | 1.267635 | -1.42202 | 1.06E-49 | 3.29E-49 |
| ANKRD29 | 2.453389 | 1.051707 | -1.40168 | 7.39E-62 | 4.60E-61 |
| EBF4 | 3.102978 | 1.957077 | -1.1459 | 2.11E-39 | 4.91E-39 |
| PTRH1 | 2.362709 | 0.065871 | -2.29684 | 1.22E-67 | 1.37E-66 |
| SELENOP | 5.711869 | 3.506753 | -2.20512 | 1.23E-64 | 1.02E-63 |
| TMSB10 | 8.434994 | 11.16056 | 2.725566 | 8.83E-86 | 1.58E-81 |
| DIO2 | 1.182654 | 2.665226 | 1.482571 | 5.68E-58 | 2.61E-57 |
| FGD6 | 1.030073 | 2.456512 | 1.426439 | 2.20E-68 | 2.75E-67 |
| AC105052.3 | 2.024827 | 0.353474 | -1.67135 | 4.83E-51 | 1.58E-50 |
| AP003555.3 | 0.488087 | 1.799821 | 1.311734 | 4.24E-42 | 1.05E-41 |
| ADAMDEC1 | 0.914582 | 2.40405 | 1.489468 | 5.16E-44 | 1.35E-43 |
| ASF1B | 1.266883 | 3.883073 | 2.616191 | 1.03E-75 | 4.08E-74 |
| MT-ATP8 | 13.52186 | 12.26609 | -1.25577 | 3.45E-48 | 1.02E-47 |
| NDUFV2 | 5.345843 | 2.996359 | -2.34948 | 3.75E-63 | 2.64E-62 |
| LINC00857 | 0.890594 | 2.052679 | 1.162085 | 4.63E-53 | 1.64E-52 |
| ENOPH1 | 3.04364 | 4.557761 | 1.514121 | 1.00E-80 | 2.21E-78 |
| S100A1 | 2.659272 | 0.768866 | -1.89041 | 2.05E-64 | 1.65E-63 |
| PPP1R15B | 3.4649 | 4.490044 | 1.025145 | 2.44E-55 | 9.66E-55 |
| MROH7 | 1.521145 | 0.120695 | -1.40045 | 8.22E-72 | 1.67E-70 |
| SP2 | 2.808782 | 4.238778 | 1.429995 | 2.92E-60 | 1.60E-59 |
| AP1M1 | 3.466503 | 2.086364 | -1.38014 | 2.44E-71 | 4.59E-70 |
| SENP3 | 3.964431 | 2.458408 | -1.50602 | 1.38E-62 | 9.12E-62 |
| AC246787.1 | 1.167015 | 3.940625 | 2.77361 | 2.48E-64 | 1.98E-63 |
| CYP2B6 | 0.174639 | 1.69982 | 1.525181 | 1.39E-54 | 5.30E-54 |
| SMIM7 | 4.647164 | 2.914059 | -1.73311 | 2.73E-71 | 5.10E-70 |
| FTH1P2 | 1.958885 | 3.142151 | 1.183266 | 1.23E-50 | 3.95E-50 |
| RAD23B | 4.049169 | 5.537491 | 1.488322 | 3.24E-70 | 5.22E-69 |
| RPS29P5 | 0.628219 | 2.745931 | 2.117712 | 4.13E-60 | 2.23E-59 |
| HBB | 4.583721 | 3.502569 | -1.08115 | 7.50E-10 | 9.68E-10 |
| RPS14 | 8.27857 | 7.244277 | -1.03429 | 2.76E-57 | 1.22E-56 |
| PGA3 | 10.65706 | 0.994929 | -9.66213 | 1.07E-68 | 1.39E-67 |
| AL603825.1 | 0.3556 | 2.269779 | 1.914179 | 4.03E-62 | 2.56E-61 |
| CA11 | 3.006329 | 1.824987 | -1.18134 | 6.25E-59 | 3.08E-58 |
| GNMT | 2.724394 | 0.424243 | -2.30015 | 4.35E-63 | 3.04E-62 |
| ADH7 | 1.590494 | 0.255069 | -1.33543 | 2.24E-50 | 7.13E-50 |
| RNA5SP283 | 0.319285 | 1.854147 | 1.534862 | 9.13E-57 | 3.92E-56 |
| GSTZ1 | 2.890645 | 1.372081 | -1.51856 | 3.15E-60 | 1.72E-59 |
| POP4 | 4.164423 | 2.710474 | -1.45395 | 2.67E-52 | 9.17E-52 |
| ARMC1 | 2.680598 | 3.809711 | 1.129113 | 1.72E-68 | 2.19E-67 |
| WBP2 | 5.747391 | 4.632786 | -1.11461 | 9.42E-73 | 2.24E-71 |
| NEDD8 | 5.656529 | 3.746348 | -1.91018 | 1.80E-64 | 1.45E-63 |
| SRP14P2 | 0.353831 | 1.463631 | 1.1098 | 8.59E-57 | 3.69E-56 |
| RNF130 | 4.406465 | 2.532906 | -1.87356 | 1.02E-67 | 1.16E-66 |
| TM4SF1 | 5.106246 | 6.128866 | 1.022621 | 1.55E-22 | 2.55E-22 |
| EWSR1 | 5.719359 | 4.61725 | -1.10211 | 1.48E-50 | 4.74E-50 |
| EEF2 | 8.313697 | 9.587969 | 1.274272 | 6.29E-64 | 4.80E-63 |
| AL732372.2 | 3.147227 | 0.136363 | -3.01086 | 2.74E-65 | 2.41E-64 |
| LMNB2 | 2.646183 | 4.501311 | 1.855128 | 2.97E-80 | 5.31E-78 |
| FRAT2 | 2.444592 | 3.74999 | 1.305397 | 4.26E-56 | 1.76E-55 |
| PHLDB1 | 4.158161 | 1.773654 | -2.38451 | 1.05E-61 | 6.45E-61 |
| RNF186 | 0.433753 | 2.175781 | 1.742028 | 1.22E-49 | 3.79E-49 |
| EIF3G | 6.313463 | 5.100542 | -1.21292 | 7.24E-67 | 7.51E-66 |
| LTBP3 | 5.008712 | 3.709564 | -1.29915 | 1.63E-43 | 4.20E-43 |
| AL354892.2 | 0.663812 | 2.134307 | 1.470496 | 2.53E-65 | 2.23E-64 |
| C2orf74 | 2.808278 | 0.671901 | -2.13638 | 5.29E-72 | 1.10E-70 |
| METTL17 | 4.60991 | 2.588293 | -2.02162 | 1.43E-57 | 6.40E-57 |
| AC074212.1 | 2.138398 | 0.443144 | -1.69525 | 8.58E-65 | 7.20E-64 |
| APOBEC3B | 1.049588 | 2.355843 | 1.306255 | 2.85E-40 | 6.77E-40 |
| PSAPL1 | 2.33516 | 1.011484 | -1.32368 | 6.90E-18 | 1.04E-17 |
| CYP2AB1P | 1.969874 | 0.157669 | -1.8122 | 1.34E-35 | 2.85E-35 |
| IGKV1D-39 | 4.730162 | 2.499798 | -2.23036 | 1.29E-21 | 2.07E-21 |
| SRPX | 3.983031 | 2.235839 | -1.74719 | 3.06E-46 | 8.52E-46 |
| LEF1 | 1.050089 | 2.111292 | 1.061203 | 7.19E-57 | 3.11E-56 |
| SELENON | 4.313093 | 5.782082 | 1.468989 | 1.77E-70 | 2.95E-69 |
| MCM4 | 2.367302 | 4.201733 | 1.83443 | 5.85E-74 | 1.72E-72 |
| HGH1 | 2.808232 | 4.180383 | 1.372151 | 1.22E-73 | 3.40E-72 |
| IHH | 2.260487 | 3.39866 | 1.138173 | 3.61E-15 | 5.16E-15 |
| TBL1XR1 | 3.126082 | 4.143894 | 1.017812 | 4.45E-72 | 9.35E-71 |
| ERCC3 | 3.627779 | 2.604284 | -1.0235 | 5.49E-58 | 2.53E-57 |
| FAM149A | 2.849655 | 1.297736 | -1.55192 | 8.76E-69 | 1.16E-67 |
| CYB561A3 | 3.302608 | 2.109064 | -1.19354 | 3.23E-59 | 1.63E-58 |
| FAHD2CP | 2.751867 | 1.105692 | -1.64617 | 3.85E-72 | 8.25E-71 |
| KIF15 | 0.69334 | 1.902819 | 1.209479 | 1.92E-63 | 1.39E-62 |
| BOC | 2.844589 | 1.356683 | -1.48791 | 7.95E-38 | 1.78E-37 |
| RANP1 | 0.479336 | 1.64865 | 1.169315 | 1.62E-68 | 2.06E-67 |
| VAMP1 | 2.564204 | 1.561075 | -1.00313 | 1.48E-53 | 5.35E-53 |
| TAP1 | 3.775417 | 5.482048 | 1.706632 | 1.03E-60 | 5.84E-60 |
| CDCP1 | 2.11323 | 4.16128 | 2.04805 | 1.45E-59 | 7.52E-59 |
| MIR635 | 0.315762 | 2.194236 | 1.878474 | 2.59E-66 | 2.52E-65 |
| CST1 | 0.118277 | 4.866717 | 4.74844 | 5.70E-85 | 3.40E-81 |
| AC090498.1 | 8.41113 | 3.422615 | -4.98852 | 1.07E-67 | 1.22E-66 |
| PRKAR2A | 2.685553 | 4.106689 | 1.421136 | 1.99E-63 | 1.44E-62 |
| ARHGEF37 | 3.288879 | 1.928857 | -1.36002 | 1.48E-57 | 6.64E-57 |
| TPGS1 | 2.642306 | 1.629846 | -1.01246 | 1.98E-64 | 1.59E-63 |
| RPL26P19 | 2.025899 | 3.620716 | 1.594818 | 2.14E-52 | 7.38E-52 |
| TRIM29 | 0.966371 | 2.50468 | 1.538308 | 1.61E-27 | 2.90E-27 |
| RPL10AP6 | 1.710485 | 4.258289 | 2.547803 | 1.60E-60 | 8.92E-60 |
| MIR4664 | 0.313292 | 3.081555 | 2.768263 | 5.89E-76 | 2.45E-74 |
| PIK3C2G | 2.345076 | 1.253175 | -1.0919 | 3.19E-19 | 4.91E-19 |
| FTH1P8 | 1.524361 | 2.925339 | 1.400978 | 8.14E-57 | 3.51E-56 |
| PRRG4 | 1.266915 | 3.089572 | 1.822657 | 1.07E-64 | 8.84E-64 |
| GCKR | 2.059441 | 0.614867 | -1.44457 | 4.64E-40 | 1.09E-39 |
| SLC7A5 | 3.741788 | 4.925717 | 1.183929 | 9.25E-28 | 1.67E-27 |
| CD2AP | 2.808167 | 4.530047 | 1.72188 | 1.05E-68 | 1.37E-67 |
| SARNP | 3.903662 | 0.692412 | -3.21125 | 1.97E-57 | 8.75E-57 |
| TAGLN | 8.862706 | 5.808427 | -3.05428 | 3.32E-48 | 9.82E-48 |
| SAFB | 4.594293 | 3.516119 | -1.07817 | 2.75E-52 | 9.43E-52 |
| HLA-DRB5 | 3.085632 | 5.807098 | 2.721466 | 6.43E-43 | 1.63E-42 |
| PIN1 | 4.571961 | 3.262269 | -1.30969 | 4.64E-76 | 1.96E-74 |
| IGHV3-74 | 2.613223 | 4.668401 | 2.055178 | 2.50E-24 | 4.25E-24 |
| FTH1P20 | 1.481068 | 3.004053 | 1.522985 | 1.43E-61 | 8.68E-61 |
| SULT1A1 | 3.136226 | 1.346608 | -1.78962 | 9.23E-59 | 4.49E-58 |
| RHNO1 | 2.408343 | 3.74253 | 1.334187 | 8.73E-72 | 1.76E-70 |
| AC009084.3 | 2.359001 | 0.002008 | -2.35699 | 1.78E-78 | 1.35E-76 |
| ERMARD | 2.969301 | 1.953231 | -1.01607 | 1.00E-48 | 3.01E-48 |
| CPNE3 | 3.353995 | 4.396121 | 1.042127 | 2.88E-63 | 2.06E-62 |
| CLPS | 2.015049 | 0.344412 | -1.67064 | 6.41E-50 | 2.00E-49 |
| PRC1 | 2.332411 | 3.353566 | 1.021155 | 6.65E-36 | 1.43E-35 |
| SLC29A2 | 2.240455 | 3.776746 | 1.536291 | 4.48E-59 | 2.24E-58 |
| SPIN2B | 2.292009 | 1.050372 | -1.24164 | 6.51E-73 | 1.59E-71 |
| SNORD14E | 0.104103 | 1.560036 | 1.455933 | 4.02E-65 | 3.47E-64 |
| TLNRD1 | 2.363499 | 3.88788 | 1.524381 | 1.01E-69 | 1.50E-68 |
| IL2RA | 0.574658 | 1.637165 | 1.062507 | 5.67E-52 | 1.93E-51 |
| SUGP2 | 4.080144 | 2.761144 | -1.319 | 6.34E-53 | 2.23E-52 |
| PRR7 | 0.983497 | 3.179693 | 2.196196 | 7.57E-80 | 1.22E-77 |
| AC113398.1 | 0.367467 | 2.275392 | 1.907926 | 1.97E-64 | 1.59E-63 |
| CEBPG | 2.910541 | 3.918804 | 1.008263 | 8.86E-58 | 4.02E-57 |
| ARFRP1 | 3.49905 | 2.490188 | -1.00886 | 7.49E-53 | 2.63E-52 |
| CMAHP | 2.703365 | 1.271059 | -1.43231 | 5.42E-61 | 3.13E-60 |
| SLC12A2 | 3.268962 | 4.309529 | 1.040568 | 9.17E-26 | 1.60E-25 |
| PAN2 | 4.543899 | 2.466238 | -2.07766 | 5.42E-56 | 2.22E-55 |
| CCL20 | 1.26909 | 4.36871 | 3.099619 | 3.40E-54 | 1.27E-53 |
| AC016876.2 | 1.709516 | 0.695541 | -1.01397 | 4.21E-56 | 1.74E-55 |
| ARL5B | 1.82661 | 3.350787 | 1.524178 | 4.23E-67 | 4.49E-66 |
| DSG2 | 2.784216 | 5.828011 | 3.043795 | 8.41E-72 | 1.70E-70 |
| TEKT4P2 | 2.032647 | 0.887893 | -1.14475 | 1.78E-52 | 6.17E-52 |
| SNORD104 | 0.480628 | 4.052099 | 3.571471 | 1.05E-74 | 3.52E-73 |
| EPS8L3 | 3.539494 | 4.594608 | 1.055114 | 4.60E-05 | 5.32E-05 |
| SGK1 | 5.194842 | 3.273663 | -1.92118 | 2.66E-45 | 7.19E-45 |
| AL355802.1 | 0.474407 | 1.558958 | 1.084551 | 1.50E-72 | 3.44E-71 |
| CDHR3 | 1.953985 | 0.709866 | -1.24412 | 1.68E-63 | 1.23E-62 |
| RPN2 | 5.646439 | 7.573123 | 1.926684 | 1.10E-84 | 3.62E-81 |
| UNC5B | 1.886333 | 2.914346 | 1.028013 | 4.43E-41 | 1.07E-40 |
| TSSC2 | 1.832848 | 0.465178 | -1.36767 | 2.84E-61 | 1.68E-60 |
| FAF2 | 2.906691 | 3.925404 | 1.018713 | 8.73E-80 | 1.37E-77 |
| WASH6P | 3.901173 | 1.328289 | -2.57288 | 1.56E-64 | 1.27E-63 |
| F12 | 0.627913 | 1.964394 | 1.336482 | 4.44E-49 | 1.35E-48 |
| HMGA1 | 4.702461 | 7.959687 | 3.257226 | 1.22E-77 | 7.42E-76 |
| CHMP2B | 3.50992 | 4.534737 | 1.024817 | 2.32E-59 | 1.19E-58 |
| ME3 | 3.315714 | 1.953459 | -1.36225 | 4.74E-66 | 4.50E-65 |
| AC011511.4 | 1.857343 | 0.02012 | -1.83722 | 1.09E-61 | 6.66E-61 |
| AC242842.1 | 0.410912 | 1.628524 | 1.217612 | 3.14E-56 | 1.31E-55 |
| HES4 | 2.498641 | 3.994835 | 1.496193 | 5.23E-45 | 1.40E-44 |
| CDKN2A | 1.080024 | 2.22638 | 1.146356 | 4.80E-14 | 6.72E-14 |
| MRGBP | 2.149827 | 3.295202 | 1.145376 | 4.35E-71 | 7.88E-70 |
| SPATA20 | 4.534641 | 3.498763 | -1.03588 | 1.31E-52 | 4.54E-52 |
| GNE | 1.848821 | 3.024107 | 1.175286 | 4.04E-39 | 9.33E-39 |
| FAM199X | 2.155592 | 3.214446 | 1.058854 | 5.82E-75 | 2.04E-73 |
| WIPF2 | 2.702062 | 3.968039 | 1.265977 | 7.10E-65 | 6.00E-64 |
| CYTH2 | 4.112533 | 2.767858 | -1.34467 | 3.17E-62 | 2.03E-61 |
| DLG4 | 2.6597 | 1.387686 | -1.27201 | 5.56E-58 | 2.56E-57 |
| IGHV1-58 | 1.17015 | 2.369601 | 1.199451 | 1.73E-16 | 2.52E-16 |
| CTSK | 3.805926 | 5.12099 | 1.315064 | 8.64E-30 | 1.63E-29 |
| MPDZ | 2.289853 | 1.194279 | -1.09557 | 7.46E-38 | 1.67E-37 |
| AC025259.1 | 2.136673 | 0.011182 | -2.12549 | 1.11E-70 | 1.89E-69 |
| PID1 | 3.086893 | 1.95522 | -1.13167 | 3.25E-25 | 5.62E-25 |
| PRR15 | 2.29516 | 4.552713 | 2.257553 | 2.82E-53 | 1.01E-52 |
| AC009362.1 | 0.213002 | 1.267246 | 1.054244 | 6.71E-63 | 4.60E-62 |
| WDR27 | 3.300366 | 1.103932 | -2.19643 | 1.82E-53 | 6.56E-53 |
| ONECUT2 | 0.243143 | 2.244739 | 2.001597 | 3.12E-71 | 5.77E-70 |
| NUTM2B-AS1 | 2.624209 | 0.944943 | -1.67927 | 1.90E-54 | 7.18E-54 |
| C4orf48 | 2.511278 | 4.256587 | 1.745309 | 1.54E-48 | 4.61E-48 |
| IKZF3 | 0.923499 | 2.001792 | 1.078293 | 2.27E-36 | 4.95E-36 |
| TNFRSF12A | 3.73595 | 5.057167 | 1.321217 | 1.03E-31 | 2.04E-31 |
| SGO1 | 0.515396 | 1.944779 | 1.429383 | 2.19E-74 | 6.84E-73 |
| SELL | 1.11489 | 2.654593 | 1.539703 | 3.57E-48 | 1.05E-47 |
| NUDT15 | 2.334581 | 3.898745 | 1.564164 | 1.57E-75 | 5.95E-74 |
| RPS29P16 | 1.029559 | 2.271805 | 1.242246 | 2.31E-42 | 5.78E-42 |
| FLYWCH1 | 3.45153 | 2.264119 | -1.18741 | 1.68E-63 | 1.23E-62 |
| HACD1 | 3.131508 | 0.957502 | -2.17401 | 6.79E-69 | 9.05E-68 |
| COL8A1 | 1.403473 | 2.830382 | 1.426909 | 4.63E-33 | 9.38E-33 |
| HOXC10 | 0.127686 | 2.239188 | 2.111501 | 9.59E-48 | 2.79E-47 |
| TXLNGY | 2.025067 | 0.814898 | -1.21017 | 4.67E-14 | 6.55E-14 |
| MTCO3P12 | 2.500607 | 5.80037 | 3.299763 | 1.19E-58 | 5.73E-58 |
| PINLYP | 1.800566 | 0.547696 | -1.25287 | 9.56E-70 | 1.43E-68 |
| RTRAF | 5.710711 | 3.783694 | -1.92702 | 1.09E-62 | 7.27E-62 |
| KRT17 | 0.8008 | 3.660104 | 2.859304 | 1.32E-45 | 3.62E-45 |
| FCER1G | 4.053473 | 5.941404 | 1.887932 | 1.30E-51 | 4.37E-51 |
| BCAT2 | 4.528494 | 3.152499 | -1.376 | 6.12E-55 | 2.38E-54 |
| EEF1D | 7.154575 | 3.51798 | -3.63659 | 8.44E-69 | 1.12E-67 |
| CEP95 | 3.500533 | 1.979163 | -1.52137 | 1.10E-50 | 3.53E-50 |
| LINC01089 | 3.281508 | 1.189048 | -2.09246 | 6.64E-60 | 3.53E-59 |
| HARS | 4.143694 | 2.6501 | -1.49359 | 9.77E-68 | 1.12E-66 |
| TPTEP2 | 1.679685 | 0.673356 | -1.00633 | 3.59E-58 | 1.67E-57 |
| AL031727.1 | 1.686302 | 3.048288 | 1.361987 | 2.49E-53 | 8.92E-53 |
| NPIPB4 | 3.816447 | 0.649735 | -3.16671 | 5.97E-52 | 2.03E-51 |
| DUS1L | 5.463189 | 4.153677 | -1.30951 | 2.97E-51 | 9.77E-51 |
| LAT | 2.514765 | 0.127049 | -2.38772 | 3.35E-61 | 1.96E-60 |
| AL450998.1 | 0.67875 | 2.43183 | 1.75308 | 1.65E-63 | 1.21E-62 |
| TRAF3 | 1.639779 | 2.695879 | 1.0561 | 7.77E-64 | 5.87E-63 |
| BCL11B | 0.546242 | 1.777676 | 1.231434 | 8.25E-65 | 6.93E-64 |
| LRRCC1 | 1.620989 | 2.674411 | 1.053422 | 2.53E-65 | 2.23E-64 |
| AKAP11 | 2.197384 | 3.286283 | 1.088899 | 4.20E-61 | 2.45E-60 |
| USH1C | 2.596121 | 3.861829 | 1.265708 | 1.90E-27 | 3.42E-27 |
| MAN1A2 | 2.304259 | 3.353074 | 1.048815 | 3.86E-64 | 3.02E-63 |
| AC144530.1 | 0.684078 | 2.225267 | 1.541189 | 1.17E-59 | 6.12E-59 |
| LUC7L | 4.550959 | 2.331598 | -2.21936 | 3.87E-56 | 1.60E-55 |
| APOD | 6.487546 | 4.321472 | -2.16607 | 4.18E-30 | 7.96E-30 |
| HACD3 | 3.446076 | 4.483838 | 1.037763 | 3.91E-55 | 1.53E-54 |
| REST | 1.884975 | 3.169983 | 1.285008 | 3.24E-69 | 4.50E-68 |
| MT1F | 3.956861 | 2.676719 | -1.28014 | 3.79E-21 | 6.04E-21 |
| ZNF655 | 4.185081 | 2.454521 | -1.73056 | 4.35E-69 | 5.93E-68 |
| RALBP1 | 3.353865 | 4.518917 | 1.165053 | 5.77E-67 | 6.01E-66 |
| POMT2 | 2.534777 | 1.461184 | -1.07359 | 8.91E-54 | 3.26E-53 |
| SKAP2 | 2.251456 | 3.553202 | 1.301746 | 6.47E-47 | 1.84E-46 |
| SBSPON | 2.505293 | 1.240947 | -1.26435 | 1.47E-43 | 3.79E-43 |
| SNX10 | 0.85845 | 2.902891 | 2.044441 | 3.66E-82 | 1.37E-79 |
| DYNC1LI2 | 4.690294 | 3.612921 | -1.07737 | 1.17E-59 | 6.09E-59 |
| SYTL4 | 2.675056 | 1.349683 | -1.32537 | 7.00E-68 | 8.18E-67 |
| EPOP | 0.78883 | 1.800755 | 1.011925 | 1.38E-42 | 3.46E-42 |
| RNU2-27P | 0.313723 | 1.551831 | 1.238108 | 5.92E-58 | 2.72E-57 |
| C2orf40 | 4.625865 | 1.417318 | -3.20855 | 2.74E-54 | 1.03E-53 |
| AC138866.1 | 1.988869 | 0.362639 | -1.62623 | 2.35E-55 | 9.33E-55 |
| FOSL1 | 2.212541 | 3.518058 | 1.305517 | 6.20E-23 | 1.02E-22 |
| PLCG1 | 4.199361 | 3.178733 | -1.02063 | 7.12E-44 | 1.85E-43 |
| HMGN4 | 3.724875 | 5.041415 | 1.31654 | 2.73E-55 | 1.08E-54 |
| AMMECR1 | 1.240945 | 2.437467 | 1.196522 | 1.01E-70 | 1.75E-69 |
| GOLM1 | 4.573689 | 6.172353 | 1.598664 | 8.49E-36 | 1.82E-35 |
| CATSPER2 | 1.604197 | 0.557969 | -1.04623 | 2.44E-49 | 7.49E-49 |
| RNPC3 | 2.905604 | 1.37928 | -1.52632 | 1.05E-58 | 5.07E-58 |
| TTYH3 | 2.849232 | 5.34247 | 2.493237 | 1.02E-83 | 1.43E-80 |
| COL17A1 | 1.476493 | 2.520829 | 1.044336 | 1.85E-14 | 2.61E-14 |
| AGR3 | 2.580695 | 4.814981 | 2.234286 | 9.21E-31 | 1.77E-30 |
| RNU4-1 | 0.755606 | 1.881189 | 1.125583 | 9.12E-28 | 1.65E-27 |
| CD2 | 1.876907 | 3.293498 | 1.416591 | 7.15E-37 | 1.57E-36 |
| LINC01133 | 2.890651 | 4.142539 | 1.251888 | 5.46E-17 | 8.07E-17 |
| ACAA1 | 4.649724 | 2.578762 | -2.07096 | 9.32E-74 | 2.64E-72 |
| MTCO1P2 | 0.482191 | 3.033914 | 2.551722 | 6.30E-64 | 4.81E-63 |
| NDFIP2 | 2.864649 | 4.092444 | 1.227795 | 1.27E-55 | 5.09E-55 |
| DMTF1 | 4.0562 | 2.453153 | -1.60305 | 2.22E-53 | 7.97E-53 |
| DPM1 | 4.084758 | 5.373974 | 1.289215 | 2.02E-77 | 1.16E-75 |
| SPACA6 | 2.354083 | 0.675475 | -1.67861 | 8.23E-62 | 5.10E-61 |
| NBPF14 | 2.109139 | 0.420178 | -1.68896 | 4.58E-63 | 3.19E-62 |
| BRF1 | 2.794814 | 1.124658 | -1.67016 | 7.90E-59 | 3.87E-58 |
| LINC00671 | 1.314526 | 0.064056 | -1.25047 | 3.63E-76 | 1.57E-74 |
| AL135999.1 | 2.237511 | 0.695538 | -1.54197 | 1.15E-52 | 3.99E-52 |
| STRADA | 3.314506 | 1.108667 | -2.20584 | 1.15E-60 | 6.50E-60 |
| MTND1P23 | 4.388059 | 5.54746 | 1.159401 | 3.65E-22 | 5.95E-22 |
| GZMB | 1.370655 | 2.675531 | 1.304876 | 2.75E-35 | 5.84E-35 |
| AC004982.2 | 2.838338 | 1.671846 | -1.16649 | 9.08E-31 | 1.75E-30 |
| A1BG | 1.783542 | 0.0676 | -1.71594 | 2.34E-72 | 5.23E-71 |
| IGLV1-47 | 3.775723 | 5.522673 | 1.746951 | 2.01E-14 | 2.83E-14 |
| AC098935.1 | 0.289969 | 1.336448 | 1.046478 | 1.38E-62 | 9.12E-62 |
| PPP2R5C | 4.33556 | 3.249786 | -1.08577 | 4.63E-67 | 4.89E-66 |
| MCM3 | 3.504036 | 5.183585 | 1.679549 | 1.62E-82 | 8.57E-80 |
| FAH | 2.740957 | 1.428301 | -1.31266 | 2.06E-63 | 1.49E-62 |
| HSD17B11 | 3.857945 | 5.079732 | 1.221787 | 7.31E-48 | 2.14E-47 |
| NECAB3 | 4.067384 | 2.773984 | -1.2934 | 1.84E-60 | 1.02E-59 |
| RMI1 | 1.376867 | 2.568477 | 1.191611 | 4.33E-72 | 9.13E-71 |
| CDK6 | 1.852074 | 3.227641 | 1.375567 | 3.74E-63 | 2.63E-62 |
| GATD3A | 2.55675 | 0.590214 | -1.96654 | 7.58E-63 | 5.16E-62 |
| ADGRD1 | 2.861701 | 0.627686 | -2.23402 | 9.40E-72 | 1.89E-70 |
| SH2D2A | 1.2445 | 2.347502 | 1.103003 | 4.31E-56 | 1.78E-55 |
| MAN2A2 | 4.147992 | 2.988807 | -1.15918 | 1.24E-51 | 4.16E-51 |
| ANP32E | 2.594804 | 4.434017 | 1.839213 | 3.20E-82 | 1.37E-79 |
| GHR | 1.758686 | 0.620272 | -1.13841 | 8.89E-59 | 4.33E-58 |
| EFNA4 | 1.957232 | 3.59634 | 1.639108 | 2.31E-70 | 3.79E-69 |
| SEPHS2 | 4.193389 | 5.925818 | 1.732429 | 1.50E-70 | 2.53E-69 |
| LCN2 | 4.507187 | 8.198214 | 3.691027 | 3.79E-34 | 7.86E-34 |
| TNFSF13B | 1.10974 | 2.625662 | 1.515922 | 6.11E-62 | 3.83E-61 |
| ZNF83 | 3.776765 | 2.040681 | -1.73608 | 7.10E-56 | 2.89E-55 |
| GPR155 | 3.080876 | 1.692153 | -1.38872 | 2.43E-51 | 8.04E-51 |
| RPL10P12 | 0.364598 | 1.391846 | 1.027247 | 3.04E-62 | 1.95E-61 |
| VSIG2 | 5.766846 | 4.105305 | -1.66154 | 1.32E-19 | 2.05E-19 |
| RPL12P16 | 2.268115 | 0.002008 | -2.26611 | 1.78E-78 | 1.35E-76 |
| IRF9 | 4.436583 | 1.612804 | -2.82378 | 1.96E-51 | 6.51E-51 |
| HAND2 | 3.123742 | 1.342804 | -1.78094 | 4.95E-28 | 9.03E-28 |
| LINC00476 | 1.89474 | 0.767252 | -1.12749 | 8.25E-74 | 2.35E-72 |
| DDX39B | 6.259224 | 3.419541 | -2.83968 | 4.73E-52 | 1.61E-51 |
| UBA52P5 | 0.395074 | 1.693657 | 1.298583 | 1.07E-61 | 6.55E-61 |
| CHRM3-AS2 | 1.411149 | 0.258471 | -1.15268 | 9.35E-50 | 2.91E-49 |
| PLCXD3 | 1.296659 | 0.276327 | -1.02033 | 2.09E-70 | 3.44E-69 |
| C2 | 1.921485 | 2.994908 | 1.073424 | 2.95E-32 | 5.87E-32 |
| AL732372.3 | 1.788775 | 0.048935 | -1.73984 | 6.15E-60 | 3.28E-59 |
| IL2RG | 2.60708 | 5.154257 | 2.547177 | 1.07E-57 | 4.84E-57 |
| CDK11A | 4.283369 | 1.491971 | -2.7914 | 6.04E-58 | 2.77E-57 |
| GOLGA2P5 | 2.123841 | 0.703225 | -1.42062 | 3.64E-56 | 1.51E-55 |
| IKBKG | 3.106398 | 1.161717 | -1.94468 | 4.04E-69 | 5.53E-68 |
| RPL13AP20 | 0.687036 | 2.579998 | 1.892962 | 5.87E-68 | 6.95E-67 |
| AP1M2 | 3.727023 | 5.332947 | 1.605924 | 6.46E-35 | 1.36E-34 |
| PDLIM7 | 5.552425 | 4.464263 | -1.08816 | 3.68E-16 | 5.35E-16 |
| TNXB | 3.910756 | 1.627049 | -2.28371 | 9.35E-59 | 4.54E-58 |
| ADAMTS14 | 0.578307 | 1.642593 | 1.064286 | 4.69E-65 | 4.03E-64 |
| PELI1 | 2.754343 | 3.994677 | 1.240334 | 1.01E-57 | 4.56E-57 |
| CTU1 | 1.497959 | 2.732844 | 1.234886 | 3.40E-59 | 1.72E-58 |
| SPAG1 | 1.517554 | 2.67107 | 1.153516 | 4.04E-54 | 1.50E-53 |
| CORO7 | 3.033655 | 1.336943 | -1.69671 | 9.95E-52 | 3.35E-51 |
| COL4A2 | 4.900295 | 6.112239 | 1.211944 | 1.11E-23 | 1.86E-23 |
| SMTN | 5.929718 | 3.724884 | -2.20483 | 7.44E-51 | 2.41E-50 |
| SCP2 | 5.354262 | 4.262012 | -1.09225 | 8.84E-69 | 1.17E-67 |
| AP003419.1 | 3.225535 | 0.021178 | -3.20436 | 2.99E-69 | 4.19E-68 |
| AC008738.3 | 1.780213 | 0.002008 | -1.77821 | 1.78E-78 | 1.35E-76 |
| UBR4 | 5.207963 | 3.449846 | -1.75812 | 2.08E-59 | 1.07E-58 |
| VDR | 1.366946 | 3.479539 | 2.112593 | 2.51E-62 | 1.62E-61 |
| SAPCD2 | 1.112591 | 3.732693 | 2.620102 | 1.80E-76 | 8.35E-75 |
| PLAU | 2.390655 | 4.472197 | 2.081542 | 1.29E-64 | 1.06E-63 |
| TMEM255B | 2.082652 | 1.00929 | -1.07336 | 2.48E-51 | 8.20E-51 |
| GJB5 | 0.301917 | 1.368337 | 1.06642 | 3.48E-23 | 5.77E-23 |
| MMAB | 3.56903 | 1.965471 | -1.60356 | 3.90E-71 | 7.13E-70 |
| PIGU | 2.61451 | 4.226258 | 1.611748 | 7.47E-81 | 1.74E-78 |
| LSS | 4.031995 | 2.937227 | -1.09477 | 1.67E-63 | 1.22E-62 |
| LINC00893 | 2.429426 | 0.427822 | -2.0016 | 6.69E-62 | 4.19E-61 |
| OGT | 5.069612 | 4.069079 | -1.00053 | 7.12E-38 | 1.60E-37 |
| DMPK | 4.930013 | 2.727879 | -2.20213 | 5.59E-61 | 3.22E-60 |
| FABP5P7 | 4.178158 | 1.621353 | -2.55681 | 4.31E-55 | 1.68E-54 |
| CD86 | 0.923317 | 2.036712 | 1.113394 | 3.31E-49 | 1.01E-48 |
| MAPRE3 | 3.650462 | 1.99491 | -1.65555 | 4.69E-71 | 8.41E-70 |
| TNFRSF21 | 3.980733 | 5.836471 | 1.855738 | 1.45E-65 | 1.31E-64 |
| RAP2A | 1.771535 | 3.175502 | 1.403967 | 5.05E-71 | 9.02E-70 |
| C5AR1 | 1.946612 | 3.013999 | 1.067387 | 2.98E-28 | 5.46E-28 |
| CYP2R1 | 3.110981 | 2.056085 | -1.0549 | 8.93E-48 | 2.60E-47 |
| HSD3B7 | 2.500216 | 3.516201 | 1.015985 | 5.42E-45 | 1.45E-44 |
| TACSTD2 | 2.62708 | 5.52367 | 2.89659 | 4.99E-44 | 1.30E-43 |
| RTF1 | 3.176823 | 4.206798 | 1.029975 | 1.02E-61 | 6.25E-61 |
| PARP6 | 4.374539 | 2.09208 | -2.28246 | 8.60E-64 | 6.46E-63 |
| AL139317.3 | 1.573035 | 0.002008 | -1.57103 | 1.78E-78 | 1.35E-76 |
| AMN | 2.250279 | 3.320504 | 1.070225 | 1.51E-18 | 2.30E-18 |
| ALDH1L1 | 1.821762 | 0.30524 | -1.51652 | 1.46E-66 | 1.46E-65 |
| ZNF514 | 2.533244 | 1.46287 | -1.07037 | 3.12E-49 | 9.53E-49 |
| NRBF2 | 2.623965 | 3.766519 | 1.142553 | 2.13E-63 | 1.54E-62 |
| TAGLN2 | 6.98211 | 8.295831 | 1.313721 | 1.01E-60 | 5.73E-60 |
| MT-TC | 0.690886 | 3.533273 | 2.842387 | 5.56E-55 | 2.16E-54 |
| MTX1P1 | 0.303504 | 1.895979 | 1.592475 | 2.30E-76 | 1.04E-74 |
| ORC1 | 0.563713 | 2.116057 | 1.552344 | 3.46E-73 | 8.81E-72 |
| TECRP1 | 1.321629 | 3.193756 | 1.872127 | 3.86E-64 | 3.02E-63 |
| RPL30 | 8.647401 | 7.02283 | -1.62457 | 4.06E-53 | 1.44E-52 |
| STARD10 | 6.419568 | 5.298641 | -1.12093 | 1.05E-17 | 1.58E-17 |
| ANTXR1 | 2.749422 | 4.196383 | 1.446961 | 1.43E-37 | 3.19E-37 |
| SYNE1 | 3.437317 | 1.010235 | -2.42708 | 1.15E-69 | 1.70E-68 |
| TBCD | 4.707114 | 2.755456 | -1.95166 | 1.73E-54 | 6.55E-54 |
| ELOF1 | 4.510403 | 3.140958 | -1.36944 | 3.82E-68 | 4.64E-67 |
| BIK | 2.203514 | 4.078173 | 1.874659 | 2.01E-53 | 7.23E-53 |
| SFRP5 | 2.088607 | 0.952406 | -1.1362 | 1.13E-35 | 2.41E-35 |
| SLC35F2 | 1.826492 | 2.97807 | 1.151578 | 1.12E-45 | 3.06E-45 |
| LIF | 1.364228 | 2.791292 | 1.427063 | 1.54E-47 | 4.46E-47 |
| SPIN4 | 1.059254 | 2.298248 | 1.238995 | 4.71E-65 | 4.04E-64 |
| IKBKB | 3.879447 | 2.248314 | -1.63113 | 2.38E-55 | 9.43E-55 |
| EFR3A | 3.040192 | 4.3009 | 1.260709 | 4.56E-63 | 3.17E-62 |
| MGST3 | 5.578394 | 3.69364 | -1.88475 | 2.05E-70 | 3.38E-69 |
| NACC1 | 3.068819 | 4.722456 | 1.653637 | 2.09E-71 | 3.95E-70 |
| WASH3P | 3.719584 | 1.621359 | -2.09822 | 2.40E-61 | 1.42E-60 |
| PTMAP9 | 0.476895 | 3.00577 | 2.528875 | 3.36E-76 | 1.46E-74 |
| MRPS18C | 3.321792 | 1.702213 | -1.61958 | 3.36E-70 | 5.40E-69 |
| AKR1C3 | 5.384586 | 3.982145 | -1.40244 | 1.27E-18 | 1.93E-18 |
| AURKA | 1.499104 | 3.809037 | 2.309933 | 9.82E-74 | 2.77E-72 |
| NPIPB2 | 1.993652 | 0.322236 | -1.67142 | 1.15E-68 | 1.49E-67 |
| MAGEA3 | 0.039767 | 1.549302 | 1.509535 | 2.96E-08 | 3.69E-08 |
| AMY2B | 2.804297 | 0.395502 | -2.40879 | 4.27E-63 | 2.99E-62 |
| RERGL | 1.921918 | 0.571933 | -1.34998 | 1.72E-58 | 8.20E-58 |
| IGFBP6 | 5.299612 | 3.544969 | -1.75464 | 3.95E-36 | 8.54E-36 |
| WASHC5 | 3.11791 | 4.228546 | 1.110636 | 2.42E-77 | 1.37E-75 |
| AL450405.1 | 3.488497 | 4.912613 | 1.424115 | 3.03E-36 | 6.58E-36 |
| TAF10 | 5.549617 | 3.889916 | -1.6597 | 2.94E-63 | 2.09E-62 |
| ERG28 | 2.942324 | 4.318496 | 1.376172 | 1.20E-60 | 6.74E-60 |
| RAC1P2 | 1.790117 | 3.780788 | 1.990671 | 2.50E-68 | 3.10E-67 |
| UBE2S | 2.851361 | 4.104855 | 1.253494 | 2.83E-49 | 8.64E-49 |
| AC091053.1 | 2.358457 | 0.002008 | -2.35645 | 1.78E-78 | 1.35E-76 |
| PTMAP5 | 1.442263 | 3.412048 | 1.969785 | 2.98E-72 | 6.53E-71 |
| RHEX | 1.673692 | 0.621388 | -1.0523 | 1.90E-52 | 6.57E-52 |
| RSRC2 | 4.470576 | 2.943622 | -1.52695 | 4.03E-55 | 1.58E-54 |
| ARHGEF10 | 2.820236 | 1.721348 | -1.09889 | 9.78E-51 | 3.15E-50 |
| IFITM3 | 7.613668 | 9.072052 | 1.458384 | 1.50E-44 | 3.97E-44 |
| NPIPB12 | 3.927461 | 0.396936 | -3.53052 | 1.50E-59 | 7.77E-59 |
| KHDRBS1 | 4.403677 | 5.575791 | 1.172115 | 2.75E-81 | 7.33E-79 |
| ATP6V1G2-DDX39B | 1.644014 | 0.00424 | -1.63977 | 2.17E-75 | 8.13E-74 |
| STK26 | 1.858847 | 3.560999 | 1.702152 | 2.88E-73 | 7.43E-72 |
| GABRP | 0.92961 | 2.855934 | 1.926324 | 1.24E-24 | 2.12E-24 |
| MFSD14B | 3.288719 | 4.650092 | 1.361373 | 1.12E-77 | 6.90E-76 |
| NUCKS1 | 4.471198 | 5.62642 | 1.155222 | 2.77E-61 | 1.64E-60 |
| AL035413.1 | 0.781059 | 1.934372 | 1.153313 | 3.66E-62 | 2.34E-61 |
| GBP4 | 1.8836 | 3.771752 | 1.888153 | 1.43E-55 | 5.71E-55 |
| AL590867.2 | 3.667505 | 5.239204 | 1.571699 | 5.30E-57 | 2.30E-56 |
| PRPS1 | 2.97107 | 3.974505 | 1.003435 | 4.35E-58 | 2.02E-57 |
| CGAS | 0.891326 | 2.40987 | 1.518544 | 2.45E-79 | 3.43E-77 |
| ANXA9 | 1.112657 | 2.578908 | 1.466251 | 5.44E-62 | 3.42E-61 |
| AL365226.2 | 0.590566 | 1.802693 | 1.212127 | 1.41E-15 | 2.03E-15 |
| SUMO1P3 | 0.722118 | 1.803489 | 1.081371 | 5.75E-65 | 4.90E-64 |
| NAPA | 5.40558 | 4.172058 | -1.23352 | 2.03E-72 | 4.57E-71 |
| PDZD11 | 3.742639 | 5.122287 | 1.379648 | 7.60E-66 | 7.04E-65 |
| BECN1 | 4.389217 | 3.197458 | -1.19176 | 1.34E-72 | 3.11E-71 |
| CFLAR | 4.244066 | 2.884897 | -1.35917 | 3.25E-63 | 2.31E-62 |
| GALNT3 | 3.170516 | 4.389063 | 1.218546 | 4.80E-21 | 7.64E-21 |
| CCNK | 2.964989 | 1.776773 | -1.18822 | 1.26E-52 | 4.40E-52 |
| NTAN1 | 3.667481 | 2.664501 | -1.00298 | 1.35E-61 | 8.23E-61 |
| AC110285.2 | 1.868222 | 0.617041 | -1.25118 | 1.31E-51 | 4.39E-51 |
| CXCR4 | 3.46746 | 4.922898 | 1.455438 | 5.88E-38 | 1.32E-37 |
| FLVCR1 | 1.496699 | 2.681766 | 1.185067 | 1.61E-61 | 9.74E-61 |
| TOP1 | 3.794323 | 5.666753 | 1.87243 | 1.70E-81 | 5.01E-79 |
| RFWD3 | 1.793645 | 3.154618 | 1.360973 | 1.48E-81 | 4.41E-79 |
| AC006254.1 | 1.207137 | 0.127196 | -1.07994 | 6.69E-70 | 1.03E-68 |
| ZNF274 | 2.70637 | 1.576512 | -1.12986 | 3.02E-72 | 6.61E-71 |
| AC136443.1 | 1.326225 | 0.047305 | -1.27892 | 6.18E-52 | 2.10E-51 |
| GIPC2 | 1.440361 | 2.555961 | 1.1156 | 6.42E-38 | 1.44E-37 |
| GPM6B | 2.093821 | 0.9259 | -1.16792 | 2.20E-45 | 5.98E-45 |
| AP001267.1 | 2.434246 | 0.060525 | -2.37372 | 1.76E-66 | 1.74E-65 |
| HHLA2 | 0.944006 | 2.220578 | 1.276572 | 9.71E-25 | 1.66E-24 |
| ABHD11 | 2.998407 | 4.583201 | 1.584793 | 9.30E-68 | 1.07E-66 |
| SUSD4 | 2.331693 | 0.940116 | -1.39158 | 2.96E-43 | 7.57E-43 |
| E2F1 | 1.416379 | 3.433795 | 2.017416 | 7.48E-78 | 4.82E-76 |
| EEF1G | 7.084768 | 0.912156 | -6.17261 | 1.97E-61 | 1.18E-60 |
| THSD4 | 2.316228 | 1.214749 | -1.10148 | 6.75E-45 | 1.80E-44 |
| GTF3C4 | 1.58849 | 2.790749 | 1.20226 | 1.56E-73 | 4.24E-72 |
| ANKHD1 | 3.879134 | 0.827083 | -3.05205 | 7.25E-60 | 3.84E-59 |
| MIR4635 | 0.098879 | 1.382706 | 1.283827 | 2.21E-56 | 9.28E-56 |
| CELA3A | 2.772396 | 0.354323 | -2.41807 | 1.87E-49 | 5.75E-49 |
| ASPN | 2.092106 | 4.200312 | 2.108206 | 1.32E-41 | 3.22E-41 |
| ABT1 | 2.536559 | 3.576493 | 1.039934 | 6.03E-66 | 5.65E-65 |
| DNAJC22 | 1.463784 | 2.753195 | 1.289412 | 4.84E-48 | 1.42E-47 |
| RNPEP | 4.075549 | 5.283573 | 1.208025 | 1.08E-65 | 9.96E-65 |
| CLU | 7.979819 | 5.027209 | -2.95261 | 4.83E-63 | 3.35E-62 |
| TUBGCP6 | 3.830631 | 2.705291 | -1.12534 | 2.81E-58 | 1.32E-57 |
| LRRC58 | 1.940175 | 3.701877 | 1.761703 | 5.15E-77 | 2.72E-75 |
| IGLV4-69 | 2.875616 | 4.209636 | 1.33402 | 3.27E-12 | 4.43E-12 |
| EFEMP2 | 3.985122 | 2.88214 | -1.10298 | 3.78E-27 | 6.77E-27 |
| AC099336.2 | 3.385889 | 4.441185 | 1.055296 | 9.02E-34 | 1.85E-33 |
| TP53 | 3.017147 | 4.442292 | 1.425144 | 6.11E-52 | 2.07E-51 |
| TMED2 | 5.223062 | 6.348275 | 1.125213 | 5.29E-75 | 1.86E-73 |
| DPP6 | 1.409364 | 0.150154 | -1.25921 | 1.02E-68 | 1.34E-67 |
| DELE1 | 4.447074 | 3.213548 | -1.23353 | 4.93E-75 | 1.74E-73 |
| RPS2P55 | 0.711009 | 1.985596 | 1.274587 | 3.91E-62 | 2.49E-61 |
| AC084125.2 | 1.648848 | 0.416709 | -1.23214 | 8.53E-56 | 3.46E-55 |
| CHAD | 1.983743 | 0.685296 | -1.29845 | 4.14E-34 | 8.57E-34 |
| RGMB | 3.811743 | 1.943708 | -1.86804 | 6.42E-68 | 7.54E-67 |
| AL022323.1 | 0.444056 | 1.516417 | 1.072361 | 9.57E-61 | 5.42E-60 |
| YEATS4 | 2.519286 | 3.696473 | 1.177187 | 5.42E-70 | 8.44E-69 |
| YWHAH | 5.133286 | 6.3957 | 1.262414 | 1.45E-67 | 1.62E-66 |
| CTIF | 3.35841 | 2.322739 | -1.03567 | 5.58E-56 | 2.28E-55 |
| LINC00674 | 1.26095 | 2.907656 | 1.646706 | 2.40E-58 | 1.13E-57 |
| MT-TP | 1.887244 | 12.00709 | 10.11984 | 1.59E-62 | 1.05E-61 |
| SIRT3 | 3.086813 | 1.92923 | -1.15758 | 1.07E-68 | 1.39E-67 |
| CHMP4A | 4.389876 | 1.320622 | -3.06925 | 4.38E-61 | 2.55E-60 |
| SOX9 | 2.657372 | 6.079407 | 3.422035 | 2.09E-82 | 9.83E-80 |
| GTF2H2C | 2.269564 | 0.477506 | -1.79206 | 5.52E-57 | 2.40E-56 |
| COQ8A | 4.93114 | 3.229866 | -1.70127 | 2.47E-60 | 1.36E-59 |
| FP671120.4 | 6.891303 | 2.017694 | -4.87361 | 7.01E-65 | 5.94E-64 |
| SAMD1 | 3.78231 | 4.962199 | 1.179888 | 2.49E-67 | 2.71E-66 |
| SFT2D2 | 2.110992 | 3.627922 | 1.51693 | 4.31E-78 | 2.93E-76 |
| CCL18 | 0.787438 | 3.751491 | 2.964053 | 5.33E-65 | 4.57E-64 |
| GAMT | 3.59417 | 1.901957 | -1.69221 | 1.15E-48 | 3.44E-48 |
| AC090984.1 | 1.563294 | 0.002008 | -1.56129 | 1.78E-78 | 1.35E-76 |
| E2F3 | 1.276403 | 2.608593 | 1.33219 | 1.65E-84 | 4.21E-81 |
| SLC44A4 | 4.423954 | 5.943951 | 1.519997 | 1.22E-13 | 1.70E-13 |
| HRH2 | 1.993305 | 0.77446 | -1.21884 | 1.15E-47 | 3.35E-47 |
| AC007387.1 | 0.326281 | 1.478233 | 1.151953 | 1.71E-52 | 5.91E-52 |
| C2orf92 | 1.88383 | 0.292093 | -1.59174 | 1.16E-55 | 4.67E-55 |
| AL138693.1 | 0.141194 | 1.375293 | 1.234099 | 6.11E-68 | 7.21E-67 |
| FTLP2 | 0.358123 | 2.427567 | 2.069444 | 4.29E-72 | 9.06E-71 |
| RNU6-531P | 0.287627 | 1.783162 | 1.495535 | 1.99E-58 | 9.42E-58 |
| RHOG | 4.369295 | 5.632436 | 1.263141 | 5.10E-60 | 2.74E-59 |
| SNORD3A | 3.902297 | 0.022905 | -3.87939 | 1.02E-67 | 1.16E-66 |
| BOLA2-SMG1P6 | 2.370343 | 0.950534 | -1.41981 | 3.89E-55 | 1.53E-54 |
| SFRP1 | 2.914525 | 1.504658 | -1.40987 | 4.68E-25 | 8.06E-25 |
| C2CD4A | 0.681708 | 2.125456 | 1.443748 | 4.83E-41 | 1.17E-40 |
| SFTA2 | 1.815493 | 3.024532 | 1.209039 | 5.55E-12 | 7.48E-12 |
| MAP7D2 | 1.8243 | 0.66409 | -1.16021 | 2.20E-38 | 5.00E-38 |
| AC098613.1 | 0.344644 | 1.490373 | 1.145729 | 1.68E-58 | 8.02E-58 |
| PSMB9 | 3.476135 | 4.567291 | 1.091155 | 5.73E-31 | 1.11E-30 |
| LACTB2 | 2.590833 | 4.503011 | 1.912179 | 1.65E-72 | 3.76E-71 |
| SLC25A15 | 1.426765 | 2.523919 | 1.097154 | 1.88E-61 | 1.13E-60 |
| RANBP9 | 3.400178 | 4.420477 | 1.020299 | 5.15E-59 | 2.56E-58 |
| TRABD2A | 0.587173 | 1.678539 | 1.091365 | 4.33E-32 | 8.58E-32 |
| HNRNPCP2 | 0.929338 | 2.00082 | 1.071483 | 4.64E-71 | 8.34E-70 |
| IGHV3-30 | 4.087228 | 5.991701 | 1.904473 | 7.14E-15 | 1.02E-14 |
| AGGF1 | 2.189323 | 3.216295 | 1.026972 | 7.36E-68 | 8.57E-67 |
| PWWP3A | 3.33187 | 2.099887 | -1.23198 | 4.07E-56 | 1.68E-55 |
| CBSL | 1.477603 | 0.055143 | -1.42246 | 2.56E-59 | 1.31E-58 |
| EDRF1 | 2.675838 | 1.641985 | -1.03385 | 1.31E-50 | 4.21E-50 |
| SERPINB1 | 5.03166 | 6.209772 | 1.178112 | 8.30E-37 | 1.82E-36 |
| HLA-DOA | 1.180981 | 2.64321 | 1.462229 | 3.47E-40 | 8.22E-40 |
| TMEM8B | 3.068096 | 1.975879 | -1.09222 | 1.59E-60 | 8.88E-60 |
| ASTN2 | 1.551577 | 0.510487 | -1.04109 | 7.28E-67 | 7.54E-66 |
| INO80C | 3.278132 | 1.708533 | -1.5696 | 5.46E-72 | 1.13E-70 |
| HERC2P9 | 3.198164 | 0.890391 | -2.30777 | 4.31E-58 | 2.00E-57 |
| AL355309.1 | 0.552802 | 3.416017 | 2.863216 | 4.62E-62 | 2.92E-61 |
| PLAGL1 | 3.349242 | 1.732005 | -1.61724 | 1.81E-59 | 9.31E-59 |
| NXF1 | 4.992209 | 3.703047 | -1.28916 | 2.66E-59 | 1.36E-58 |
| TM4SF5 | 2.178109 | 3.993765 | 1.815656 | 7.53E-32 | 1.49E-31 |
| WBP11 | 3.316389 | 4.357403 | 1.041014 | 1.62E-71 | 3.11E-70 |
| AGPAT2 | 4.596399 | 6.274928 | 1.67853 | 2.69E-64 | 2.14E-63 |
| SNRPB2 | 3.822439 | 4.831031 | 1.008592 | 6.92E-60 | 3.67E-59 |
| ZFHX2-AS1 | 1.78835 | 0.178968 | -1.60938 | 1.27E-57 | 5.71E-57 |
| HIF1A | 3.942472 | 5.531213 | 1.588741 | 9.91E-68 | 1.13E-66 |
| NDUFB1 | 6.379233 | 4.007728 | -2.37151 | 7.30E-69 | 9.72E-68 |
| NUAK2 | 1.165169 | 2.831808 | 1.666639 | 3.68E-65 | 3.19E-64 |
| CELA3B | 1.975087 | 0.279733 | -1.69535 | 2.35E-47 | 6.75E-47 |
| FAM229B | 2.799206 | 1.200691 | -1.59851 | 3.12E-66 | 3.01E-65 |
| LINC00543 | 0.541447 | 1.620878 | 1.079431 | 4.96E-46 | 1.37E-45 |
| GMCL1 | 1.946403 | 3.33563 | 1.389227 | 9.53E-77 | 4.69E-75 |
| CLCN7 | 4.419502 | 3.181453 | -1.23805 | 3.50E-51 | 1.15E-50 |
| IGHV1-18 | 3.166894 | 5.980475 | 2.813582 | 3.60E-32 | 7.15E-32 |
| ATP5F1EP2 | 1.251189 | 2.992551 | 1.741361 | 6.83E-63 | 4.67E-62 |
| EXOC3 | 4.470073 | 3.09138 | -1.37869 | 7.91E-68 | 9.17E-67 |
| SDHA | 5.263156 | 4.23299 | -1.03017 | 3.92E-55 | 1.54E-54 |
| ATOX1 | 4.652301 | 2.965584 | -1.68672 | 1.15E-64 | 9.50E-64 |
| GTF2A1 | 1.979036 | 3.135397 | 1.156361 | 9.34E-68 | 1.08E-66 |
| ALG3 | 3.883975 | 4.898112 | 1.014137 | 6.14E-66 | 5.75E-65 |
| TARDBP | 5.227691 | 3.984984 | -1.24271 | 1.38E-46 | 3.88E-46 |
| PFDN6 | 4.439074 | 3.192783 | -1.24629 | 5.71E-51 | 1.86E-50 |
| RPS29 | 9.015634 | 5.387045 | -3.62859 | 1.82E-63 | 1.32E-62 |
| ACHE | 1.157805 | 2.248998 | 1.091194 | 1.06E-20 | 1.67E-20 |
| FZD7 | 1.968581 | 2.989205 | 1.020623 | 3.17E-29 | 5.92E-29 |
| MIR4767 | 0.470627 | 2.2416 | 1.770973 | 1.33E-52 | 4.63E-52 |
| KLHL11 | 0.834858 | 2.012002 | 1.177145 | 4.50E-64 | 3.49E-63 |
| NHSL1 | 1.715943 | 2.810639 | 1.094696 | 1.12E-46 | 3.16E-46 |
| IGKV1-9 | 3.409227 | 4.645932 | 1.236706 | 8.10E-09 | 1.02E-08 |
| LAGE3 | 3.259342 | 4.395276 | 1.135934 | 5.84E-63 | 4.02E-62 |
| RBP1 | 3.265912 | 1.809377 | -1.45653 | 5.33E-50 | 1.67E-49 |
| GATB | 2.423094 | 1.051372 | -1.37172 | 2.67E-69 | 3.77E-68 |
| AURKB | 1.516611 | 3.355455 | 1.838844 | 3.15E-53 | 1.12E-52 |
| MCRIP1 | 4.530286 | 3.154202 | -1.37608 | 6.42E-60 | 3.41E-59 |
| CACNA1H | 3.55001 | 2.457411 | -1.0926 | 5.06E-19 | 7.77E-19 |
| UBA7 | 4.620702 | 3.619249 | -1.00145 | 1.06E-45 | 2.92E-45 |
| RPL23AP43 | 0.292445 | 1.509319 | 1.216874 | 7.93E-61 | 4.52E-60 |
| GABARAPL1 | 5.405215 | 3.133589 | -2.27163 | 1.63E-74 | 5.26E-73 |
| MMP9 | 0.973141 | 3.884004 | 2.910863 | 1.25E-70 | 2.12E-69 |
| MREG | 1.026389 | 2.137178 | 1.11079 | 2.19E-61 | 1.31E-60 |
| GPRIN1 | 0.587826 | 1.74857 | 1.160744 | 1.07E-59 | 5.61E-59 |
| CSRP2 | 3.822662 | 1.399634 | -2.42303 | 9.63E-71 | 1.67E-69 |
| TNFRSF10A-AS1 | 0.721136 | 2.044668 | 1.323532 | 3.62E-70 | 5.79E-69 |
| HPGD | 4.402998 | 2.97945 | -1.42355 | 7.77E-27 | 1.38E-26 |
| H3F3A | 6.615232 | 4.458139 | -2.15709 | 2.15E-65 | 1.91E-64 |
| CIDEB | 1.905682 | 0.142638 | -1.76304 | 4.27E-77 | 2.28E-75 |
| ZFPM1 | 2.155543 | 3.56448 | 1.408937 | 2.12E-47 | 6.11E-47 |
| SLC17A5 | 2.364908 | 3.42799 | 1.063082 | 9.58E-59 | 4.66E-58 |
| LUC7L3 | 5.455217 | 4.095848 | -1.35937 | 9.27E-48 | 2.70E-47 |
| SHCBP1 | 0.692461 | 1.870027 | 1.177566 | 7.57E-64 | 5.73E-63 |
| AP003555.2 | 0.100317 | 1.131873 | 1.031556 | 2.43E-74 | 7.53E-73 |
| PARPBP | 0.76545 | 1.910351 | 1.144902 | 4.68E-64 | 3.63E-63 |
| FAM219B | 3.775109 | 1.565655 | -2.20945 | 3.55E-75 | 1.29E-73 |
| EEF1A1P13 | 1.138145 | 2.644323 | 1.506178 | 9.59E-58 | 4.34E-57 |
| NADSYN1 | 4.291501 | 2.220913 | -2.07059 | 2.22E-61 | 1.32E-60 |
| FAR2 | 1.26911 | 2.449561 | 1.180451 | 2.59E-51 | 8.55E-51 |
| NAA60 | 4.577912 | 2.746547 | -1.83137 | 8.92E-69 | 1.18E-67 |
| CCNDBP1 | 4.413192 | 2.733513 | -1.67968 | 5.67E-79 | 6.96E-77 |
| C1orf53 | 1.056586 | 2.081139 | 1.024553 | 1.24E-51 | 4.16E-51 |
| PPP1R1B | 2.20079 | 4.185349 | 1.984558 | 3.17E-26 | 5.58E-26 |
| TPT1 | 9.97276 | 8.519082 | -1.45368 | 1.43E-53 | 5.20E-53 |
| JPH1 | 0.749013 | 1.916064 | 1.16705 | 3.84E-38 | 8.68E-38 |
| NCBP2-AS2 | 3.456857 | 4.469436 | 1.012578 | 4.38E-61 | 2.55E-60 |
| MYO15B | 4.919213 | 2.927109 | -1.9921 | 5.72E-55 | 2.22E-54 |
| GPX3 | 7.232602 | 4.013799 | -3.2188 | 1.34E-76 | 6.30E-75 |
| SAP25 | 1.37631 | 0.271912 | -1.1044 | 2.83E-51 | 9.34E-51 |
| SOD2 | 5.408652 | 4.276042 | -1.13261 | 1.42E-29 | 2.68E-29 |
| LASP1 | 4.776163 | 5.891821 | 1.115658 | 5.80E-54 | 2.15E-53 |
| RPL39 | 8.62392 | 6.798081 | -1.82584 | 9.32E-52 | 3.14E-51 |
| RASD1 | 3.320721 | 2.195055 | -1.12567 | 4.22E-30 | 8.03E-30 |
| ACSM5 | 1.297357 | 0.155356 | -1.142 | 6.31E-77 | 3.26E-75 |
| POLR3E | 3.586246 | 2.512286 | -1.07396 | 1.70E-52 | 5.87E-52 |
| NSUN5P2 | 3.381014 | 0.644785 | -2.73623 | 1.32E-53 | 4.82E-53 |
| CALU | 4.355585 | 5.544359 | 1.188774 | 1.06E-47 | 3.09E-47 |
| SPART | 3.461362 | 2.061925 | -1.39944 | 4.86E-52 | 1.66E-51 |
| WDR83OS | 5.859893 | 4.731207 | -1.12869 | 4.04E-70 | 6.40E-69 |
| RGS3 | 3.605667 | 2.339256 | -1.26641 | 1.69E-62 | 1.11E-61 |
| DUSP10 | 1.427571 | 2.554723 | 1.127153 | 1.39E-50 | 4.44E-50 |
| RSPO2 | 1.583205 | 0.386091 | -1.19711 | 5.47E-54 | 2.02E-53 |
| REEP1 | 2.12364 | 1.101246 | -1.02239 | 1.78E-34 | 3.71E-34 |
| AC008738.7 | 2.514368 | 0.002008 | -2.51236 | 1.78E-78 | 1.35E-76 |
| TMEM100 | 3.102774 | 0.769692 | -2.33308 | 2.40E-66 | 2.35E-65 |
| ALG9 | 3.00046 | 1.103265 | -1.89719 | 2.43E-65 | 2.15E-64 |
| MIR4737 | 0.235572 | 1.390635 | 1.155063 | 2.68E-51 | 8.85E-51 |
| HMGN2P3 | 0.275932 | 1.556357 | 1.280425 | 3.87E-68 | 4.70E-67 |
| EMB | 1.761834 | 3.327667 | 1.565833 | 3.37E-49 | 1.03E-48 |
| AC018638.8 | 0.368233 | 2.754198 | 2.385964 | 1.01E-70 | 1.74E-69 |
| RBBP5 | 1.7948 | 2.95376 | 1.158959 | 1.51E-77 | 8.97E-76 |
| KLC1 | 4.57089 | 1.421737 | -3.14915 | 1.88E-60 | 1.04E-59 |
| MMP1 | 2.293889 | 4.96052 | 2.666631 | 6.79E-42 | 1.68E-41 |
| SEZ6L2 | 2.597077 | 4.487995 | 1.890918 | 1.20E-52 | 4.18E-52 |
| CBWD5 | 3.119036 | 0.570535 | -2.5485 | 6.79E-56 | 2.76E-55 |
| PPT2-EGFL8 | 1.797009 | 0.233815 | -1.56319 | 1.47E-66 | 1.47E-65 |
| ACTR2 | 4.281671 | 6.106753 | 1.825082 | 1.72E-80 | 3.52E-78 |
| IFNAR1 | 2.738472 | 3.873799 | 1.135327 | 2.24E-61 | 1.34E-60 |
| TTC28-AS1 | 2.792274 | 0.739065 | -2.05321 | 6.63E-68 | 7.77E-67 |
| FOXS1 | 0.642162 | 2.14267 | 1.500508 | 2.47E-67 | 2.69E-66 |
| AP000936.3 | 0.660701 | 2.586787 | 1.926085 | 4.68E-63 | 3.25E-62 |
| IFI30 | 4.309897 | 0.90827 | -3.40163 | 2.95E-50 | 9.34E-50 |
| RRN3P1 | 2.205063 | 1.068829 | -1.13623 | 1.96E-49 | 6.02E-49 |
| SAMD10 | 1.904357 | 3.057335 | 1.152978 | 2.87E-47 | 8.23E-47 |
| AP001107.2 | 1.55165 | 0.002008 | -1.54964 | 1.80E-78 | 1.35E-76 |
| CCL22 | 0.452728 | 1.718329 | 1.265601 | 1.17E-57 | 5.24E-57 |
| SF3B4 | 4.03943 | 5.957271 | 1.917841 | 2.77E-82 | 1.22E-79 |
| CARD11 | 1.035134 | 2.611889 | 1.576754 | 1.98E-46 | 5.56E-46 |
| CASTOR1 | 2.128657 | 0.408833 | -1.71982 | 1.04E-74 | 3.51E-73 |
| TBCA | 5.697886 | 4.195516 | -1.50237 | 3.24E-65 | 2.83E-64 |
| MEX3D | 2.271612 | 4.089203 | 1.817591 | 3.05E-71 | 5.64E-70 |
| TSC22D1 | 5.606726 | 4.575303 | -1.03142 | 1.44E-51 | 4.83E-51 |
| RPL15 | 8.175464 | 6.947454 | -1.22801 | 1.03E-62 | 6.90E-62 |
| AZI2 | 3.564862 | 2.028293 | -1.53657 | 2.06E-74 | 6.46E-73 |
| NDRG4 | 1.855294 | 0.526698 | -1.3286 | 1.22E-69 | 1.80E-68 |
| SAP130 | 2.540353 | 3.761994 | 1.221641 | 2.65E-67 | 2.88E-66 |
| PTPN1 | 3.369699 | 4.439228 | 1.069529 | 3.12E-62 | 2.00E-61 |
| COL15A1 | 3.36621 | 4.67642 | 1.31021 | 2.27E-29 | 4.24E-29 |
| GSTA4 | 3.985672 | 2.818369 | -1.1673 | 1.05E-44 | 2.79E-44 |
| KSR1 | 2.739352 | 1.616731 | -1.12262 | 3.89E-56 | 1.61E-55 |
| AL662899.2 | 1.843654 | 0.125432 | -1.71822 | 1.48E-64 | 1.21E-63 |
| BOLA2 | 1.745022 | 0.002747 | -1.74227 | 2.48E-78 | 1.79E-76 |
| NDRG1 | 6.331968 | 5.01438 | -1.31759 | 2.02E-53 | 7.29E-53 |
| TUBG2 | 3.987743 | 2.304328 | -1.68341 | 9.51E-69 | 1.25E-67 |
| SIAH1 | 2.696448 | 1.692332 | -1.00412 | 6.77E-61 | 3.88E-60 |
| MCRIP2 | 4.231103 | 3.001192 | -1.22991 | 1.54E-43 | 3.98E-43 |
| ZSCAN32 | 1.967045 | 0.734491 | -1.23255 | 2.17E-59 | 1.11E-58 |
| WDR91 | 2.81097 | 1.443736 | -1.36723 | 2.88E-64 | 2.28E-63 |
| RPSAP58 | 3.95399 | 0.361234 | -3.59276 | 3.33E-56 | 1.38E-55 |
| MAGEF1 | 3.598311 | 4.645889 | 1.047577 | 2.57E-62 | 1.66E-61 |
| IP6K2 | 4.839151 | 3.418277 | -1.42087 | 1.05E-56 | 4.47E-56 |
| LINC02038 | 0.664391 | 1.706487 | 1.042096 | 1.43E-34 | 2.98E-34 |
| HLA-DQA2 | 1.020349 | 2.936907 | 1.916558 | 9.67E-34 | 1.98E-33 |
| ZSWIM8 | 5.168402 | 3.155117 | -2.01329 | 3.71E-72 | 8.00E-71 |
| RPS15AP1 | 1.352533 | 3.426263 | 2.07373 | 1.28E-60 | 7.16E-60 |
| BMPR2 | 2.21163 | 3.605294 | 1.393664 | 3.03E-65 | 2.65E-64 |
| CMC2 | 4.060913 | 1.429901 | -2.63101 | 4.35E-59 | 2.18E-58 |
| CCNB1 | 2.112893 | 4.329854 | 2.216961 | 6.09E-65 | 5.18E-64 |
| C1QB | 4.965499 | 6.626175 | 1.660676 | 3.14E-34 | 6.51E-34 |
| ANKS4B | 0.493988 | 2.385129 | 1.891141 | 6.20E-56 | 2.53E-55 |
| KLHDC8B | 3.567079 | 2.495943 | -1.07114 | 1.25E-45 | 3.42E-45 |
| ILVBL | 4.352644 | 3.304396 | -1.04825 | 7.06E-68 | 8.25E-67 |
| SLC9A3-AS1 | 3.178746 | 1.361657 | -1.81709 | 1.25E-48 | 3.73E-48 |
| ARMT1 | 2.279562 | 3.362965 | 1.083403 | 2.56E-68 | 3.17E-67 |
| SAMD9 | 1.382174 | 3.298066 | 1.915892 | 7.38E-61 | 4.21E-60 |
| STK38 | 3.297721 | 4.645701 | 1.347981 | 1.87E-72 | 4.23E-71 |
| MRPS6 | 3.964062 | 2.760432 | -1.20363 | 1.08E-54 | 4.16E-54 |
| IGFBP4 | 6.540375 | 7.567227 | 1.026852 | 1.76E-14 | 2.48E-14 |
| METTL7B | 0.931937 | 4.026591 | 3.094654 | 7.10E-71 | 1.25E-69 |
| FAXC | 1.73321 | 0.348789 | -1.38442 | 1.70E-62 | 1.12E-61 |
| BORCS8-MEF2B | 1.645666 | 0.163452 | -1.48221 | 9.86E-68 | 1.13E-66 |
| NEDD4L | 4.114408 | 2.660776 | -1.45363 | 1.86E-36 | 4.05E-36 |
| MAEA | 4.263393 | 3.153523 | -1.10987 | 5.26E-60 | 2.82E-59 |
| SERINC3 | 4.349391 | 5.461385 | 1.111995 | 4.96E-69 | 6.71E-68 |
| PDILT | 3.532126 | 0.130834 | -3.40129 | 3.15E-53 | 1.12E-52 |
| C18orf32 | 3.997946 | 1.364828 | -2.63312 | 8.58E-76 | 3.46E-74 |
| AL391121.1 | 1.762998 | 3.378178 | 1.61518 | 5.46E-45 | 1.46E-44 |
| ESRP1 | 3.260166 | 4.909863 | 1.649697 | 2.88E-46 | 8.01E-46 |
| REEP3 | 2.771962 | 4.124453 | 1.352491 | 2.82E-60 | 1.54E-59 |
| FAM47E | 1.405265 | 0.314422 | -1.09084 | 1.40E-58 | 6.70E-58 |
| RNY1P16 | 0.460738 | 1.729822 | 1.269083 | 6.92E-47 | 1.96E-46 |
| NDUFS8 | 5.714137 | 4.67347 | -1.04067 | 2.49E-60 | 1.37E-59 |
| GJB1 | 2.383266 | 4.017959 | 1.634692 | 8.83E-34 | 1.81E-33 |
| AL590682.1 | 0.218493 | 1.468938 | 1.250445 | 1.29E-65 | 1.18E-64 |
| PPIAP11 | 0.257755 | 1.468965 | 1.21121 | 1.34E-72 | 3.10E-71 |
| ACTB | 9.895929 | 11.51947 | 1.623544 | 2.74E-34 | 5.70E-34 |
| FTLP3 | 2.572256 | 5.274075 | 2.701819 | 3.84E-71 | 7.04E-70 |
| NUP153 | 2.637637 | 3.917928 | 1.280291 | 3.57E-73 | 9.07E-72 |
| MZF1 | 3.654004 | 2.261303 | -1.3927 | 1.30E-56 | 5.51E-56 |
| ABHD16A | 3.891936 | 1.497526 | -2.39441 | 7.61E-59 | 3.73E-58 |
| COL13A1 | 1.663818 | 0.644274 | -1.01954 | 5.76E-54 | 2.13E-53 |
| PLA2G6 | 3.621988 | 1.60851 | -2.01348 | 5.83E-61 | 3.35E-60 |
| HNRNPDL | 6.281924 | 5.058374 | -1.22355 | 7.00E-55 | 2.71E-54 |
| RPL14P1 | 2.982153 | 4.243579 | 1.261426 | 2.59E-53 | 9.28E-53 |
| AL513534.1 | 0.155604 | 1.607429 | 1.451826 | 8.38E-66 | 7.75E-65 |
| FP565260.1 | 1.924795 | 0.609678 | -1.31512 | 2.30E-49 | 7.05E-49 |
| CD24 | 6.081569 | 8.251966 | 2.170397 | 1.56E-28 | 2.87E-28 |
| ZNF839 | 2.052221 | 0.879048 | -1.17317 | 7.29E-68 | 8.50E-67 |
| PPP3CA | 2.660484 | 3.924729 | 1.264244 | 1.01E-69 | 1.51E-68 |
| CDC42EP1 | 5.774971 | 6.875991 | 1.101019 | 5.89E-28 | 1.07E-27 |
| RABL2A | 2.503421 | 0.854792 | -1.64863 | 8.59E-62 | 5.32E-61 |
| FAM118A | 3.423107 | 2.229312 | -1.1938 | 2.25E-41 | 5.47E-41 |
| MAATS1 | 1.605208 | 0.320937 | -1.28427 | 4.45E-69 | 6.06E-68 |
| EEF1A1P19 | 0.701892 | 2.008516 | 1.306624 | 2.58E-60 | 1.42E-59 |
| CHCHD2P9 | 0.447363 | 2.071502 | 1.62414 | 7.14E-63 | 4.87E-62 |
| GCH1 | 1.784347 | 2.860733 | 1.076386 | 2.37E-55 | 9.40E-55 |
| BBS2 | 3.352682 | 1.825233 | -1.52745 | 1.09E-67 | 1.24E-66 |
| IRS2 | 2.284414 | 3.539699 | 1.255285 | 3.07E-33 | 6.24E-33 |
| SSBP1 | 4.988597 | 3.807871 | -1.18073 | 1.11E-54 | 4.24E-54 |
| TRIM41 | 3.036176 | 1.638919 | -1.39726 | 6.99E-62 | 4.36E-61 |
| PPHLN1 | 3.287121 | 2.262089 | -1.02503 | 3.61E-50 | 1.14E-49 |
| NISCH | 4.559736 | 3.2378 | -1.32194 | 1.03E-66 | 1.05E-65 |
| AC093809.1 | 0.494901 | 3.098283 | 2.603382 | 8.48E-64 | 6.38E-63 |
| SULF2 | 3.439945 | 4.740788 | 1.300843 | 8.11E-43 | 2.05E-42 |
| FCHSD1 | 2.936836 | 1.703164 | -1.23367 | 1.21E-58 | 5.85E-58 |
| IRAK1 | 3.964107 | 5.472219 | 1.508112 | 1.36E-75 | 5.24E-74 |
| RWDD1 | 4.146551 | 2.899704 | -1.24685 | 4.41E-71 | 7.97E-70 |
| AC010970.1 | 0.812978 | 3.9239 | 3.110922 | 7.30E-56 | 2.97E-55 |
| TMEM87B | 2.653088 | 4.178911 | 1.525823 | 1.07E-76 | 5.23E-75 |
| TMEM91 | 2.372772 | 0.923452 | -1.44932 | 1.22E-71 | 2.40E-70 |
| ANAPC11 | 5.280088 | 3.781035 | -1.49905 | 5.86E-61 | 3.36E-60 |
| AKAP2 | 2.727421 | 0.227687 | -2.49973 | 1.36E-77 | 8.19E-76 |
| ACY3 | 1.981881 | 3.365581 | 1.3837 | 7.52E-33 | 1.52E-32 |
| IGLV3-25 | 3.825747 | 5.889747 | 2.064 | 5.28E-18 | 7.95E-18 |
| FAAP20 | 4.2587 | 2.321769 | -1.93693 | 1.01E-66 | 1.03E-65 |
| CHCHD2P6 | 0.883591 | 2.053397 | 1.169806 | 9.10E-57 | 3.90E-56 |
| ZBED4 | 1.645667 | 2.919154 | 1.273487 | 3.74E-75 | 1.35E-73 |
| MAGEA12 | 0.044028 | 1.076167 | 1.03214 | 9.10E-06 | 1.07E-05 |
| BUB1B | 0.86597 | 2.754118 | 1.888147 | 1.40E-73 | 3.85E-72 |
| WNT5A-AS1 | 0.38521 | 1.417961 | 1.032751 | 1.51E-54 | 5.73E-54 |
| EIF4A2 | 6.872897 | 5.697775 | -1.17512 | 9.62E-59 | 4.67E-58 |
| AL136131.3 | 3.431132 | 0.002008 | -3.42912 | 1.78E-78 | 1.35E-76 |
| LMOD1 | 5.260748 | 3.270485 | -1.99026 | 1.76E-29 | 3.30E-29 |
| CDHR2 | 1.57996 | 2.59863 | 1.01867 | 3.31E-14 | 4.66E-14 |
| SLAMF8 | 1.119727 | 2.620947 | 1.50122 | 1.46E-58 | 6.99E-58 |
| B3GAT1 | 2.790576 | 0.185319 | -2.60526 | 7.65E-69 | 1.01E-67 |
| UGT8 | 1.699 | 3.120537 | 1.421537 | 8.23E-41 | 1.98E-40 |
| AOC3 | 4.174563 | 3.12981 | -1.04475 | 2.71E-15 | 3.89E-15 |
| AC087292.1 | 2.373193 | 0.002008 | -2.37119 | 1.78E-78 | 1.35E-76 |
| AP000759.1 | 0.755462 | 1.868499 | 1.113037 | 1.55E-59 | 8.03E-59 |
| ARHGEF1 | 4.942717 | 3.824604 | -1.11811 | 2.43E-45 | 6.59E-45 |
| RPL7AP6 | 2.573116 | 3.90355 | 1.330434 | 1.21E-50 | 3.89E-50 |
| CRY2 | 3.611144 | 2.566714 | -1.04443 | 3.11E-66 | 3.00E-65 |
| PCOLCE2 | 1.589734 | 0.49031 | -1.09942 | 9.10E-42 | 2.24E-41 |
| LRRC61 | 2.204675 | 3.252318 | 1.047643 | 1.57E-58 | 7.52E-58 |
| TREM2 | 0.656325 | 2.966541 | 2.310216 | 2.76E-76 | 1.22E-74 |
| WDR72 | 0.315628 | 1.703856 | 1.388228 | 7.06E-32 | 1.40E-31 |
| ISCU | 5.783512 | 4.20979 | -1.57372 | 5.01E-70 | 7.83E-69 |
| FBXO44 | 3.420058 | 2.138474 | -1.28158 | 8.24E-59 | 4.03E-58 |
| KCNE3 | 2.269447 | 4.461399 | 2.191952 | 5.92E-51 | 1.92E-50 |
| NPSR1 | 0.07573 | 1.090485 | 1.014755 | 2.65E-32 | 5.28E-32 |
| FGA | 2.536749 | 0.623831 | -1.91292 | 1.62E-39 | 3.77E-39 |
| BDH2 | 4.269245 | 2.426451 | -1.84279 | 5.51E-78 | 3.68E-76 |
| HYI | 4.338322 | 1.880964 | -2.45736 | 1.73E-77 | 1.02E-75 |
| THBS2 | 1.565153 | 4.299269 | 2.734117 | 8.78E-62 | 5.43E-61 |
| UBA52 | 7.928281 | 6.731691 | -1.19659 | 8.46E-61 | 4.82E-60 |
| CXCL13 | 1.356636 | 3.451861 | 2.095225 | 2.87E-38 | 6.50E-38 |
| KDM1B | 1.754149 | 3.385705 | 1.631556 | 2.43E-76 | 1.09E-74 |
| NRIP1 | 1.733474 | 2.92586 | 1.192385 | 5.98E-61 | 3.43E-60 |
| IFFO1 | 2.607232 | 1.287941 | -1.31929 | 4.38E-57 | 1.92E-56 |
| CST7 | 1.971547 | 3.358443 | 1.386896 | 6.73E-38 | 1.51E-37 |
| HIST2H2BA | 1.320922 | 0.183177 | -1.13774 | 2.94E-69 | 4.12E-68 |
| STIL | 0.674629 | 2.361199 | 1.68657 | 1.17E-78 | 1.21E-76 |
| SPINT1 | 4.391218 | 6.226396 | 1.835179 | 3.23E-38 | 7.30E-38 |
| ITGA11 | 0.91681 | 2.168944 | 1.252134 | 1.21E-47 | 3.52E-47 |
| TAPBP | 4.67313 | 6.568135 | 1.895006 | 6.28E-78 | 4.12E-76 |
| UBE2D3 | 5.639462 | 4.499023 | -1.14044 | 2.37E-69 | 3.38E-68 |
| IGKV1D-8 | 0.794323 | 1.941676 | 1.147353 | 1.44E-34 | 3.00E-34 |
| HIST2H2BF | 1.865799 | 0.712004 | -1.15379 | 8.45E-35 | 1.78E-34 |
| FARP1 | 3.564216 | 1.761676 | -1.80254 | 5.95E-53 | 2.10E-52 |
| AC027644.1 | 0.155747 | 1.439814 | 1.284068 | 1.44E-73 | 3.94E-72 |
| LMTK2 | 2.116118 | 3.460224 | 1.344105 | 7.96E-65 | 6.70E-64 |
| ORAI1 | 3.144278 | 4.477408 | 1.33313 | 9.65E-61 | 5.47E-60 |
| ARMCX6 | 3.271956 | 1.82557 | -1.44639 | 6.25E-68 | 7.36E-67 |
| TPT1P4 | 0.857802 | 2.397909 | 1.540107 | 1.29E-56 | 5.48E-56 |
| OVCA2 | 2.955647 | 0.002008 | -2.95364 | 1.78E-78 | 1.35E-76 |
| LSP1P4 | 2.983611 | 0.629492 | -2.35412 | 7.07E-70 | 1.08E-68 |
| TUBAP2 | 0.364106 | 1.678238 | 1.314132 | 2.86E-70 | 4.64E-69 |
| CNFN | 1.229412 | 2.5305 | 1.301087 | 5.03E-44 | 1.31E-43 |
| CSF2RB | 1.477695 | 2.478661 | 1.000967 | 1.45E-30 | 2.79E-30 |
| TPSAB1 | 5.073235 | 3.371488 | -1.70175 | 5.70E-40 | 1.34E-39 |
| PRSS1 | 3.500965 | 1.464851 | -2.03611 | 5.24E-25 | 9.02E-25 |
| PYCR1 | 3.607834 | 4.934984 | 1.32715 | 7.16E-31 | 1.38E-30 |
| POLE3 | 3.615685 | 4.79879 | 1.183105 | 6.99E-78 | 4.55E-76 |
| IGKV3-15 | 4.05207 | 5.698603 | 1.646533 | 1.98E-13 | 2.75E-13 |
| MTFR2 | 0.707982 | 1.897309 | 1.189327 | 4.89E-62 | 3.08E-61 |
| STAG3L5P | 3.628187 | 1.393589 | -2.2346 | 6.89E-47 | 1.96E-46 |
| DUOXA1 | 3.187226 | 0.775611 | -2.41161 | 6.10E-43 | 1.54E-42 |
| EFCAB2 | 2.194265 | 0.682332 | -1.51193 | 9.22E-63 | 6.21E-62 |
| GAPDHP60 | 0.233112 | 1.24898 | 1.015868 | 2.44E-66 | 2.39E-65 |
| RPS27A | 8.52725 | 7.475206 | -1.05204 | 4.39E-55 | 1.72E-54 |
| IPO7 | 3.539237 | 4.955703 | 1.416466 | 6.37E-68 | 7.49E-67 |
| TNFRSF11A | 0.975508 | 2.280859 | 1.30535 | 2.64E-43 | 6.76E-43 |
| DYRK4 | 3.451004 | 1.70799 | -1.74301 | 2.47E-70 | 4.04E-69 |
| PKD1P6 | 3.065744 | 1.308985 | -1.75676 | 9.92E-60 | 5.19E-59 |
| FAM243B | 1.411177 | 0.006004 | -1.40517 | 1.75E-73 | 4.69E-72 |
| TPI1P1 | 1.201208 | 2.568598 | 1.36739 | 4.46E-58 | 2.06E-57 |
| GM2A | 2.664485 | 4.353305 | 1.68882 | 2.49E-62 | 1.61E-61 |
| EPHB6 | 2.192035 | 1.147844 | -1.04419 | 1.61E-44 | 4.25E-44 |
| ST6GALNAC2 | 2.101818 | 0.991928 | -1.10989 | 2.17E-52 | 7.47E-52 |
| POP7 | 3.603784 | 4.61345 | 1.009666 | 4.11E-63 | 2.88E-62 |
| IGLC1 | 5.038324 | 0.002008 | -5.03632 | 1.78E-78 | 1.35E-76 |
| MTRNR2L12 | 0.884503 | 3.652419 | 2.767916 | 7.90E-59 | 3.87E-58 |
| MYO6 | 3.036765 | 4.12265 | 1.085885 | 1.09E-51 | 3.66E-51 |
| TXNDC17 | 5.175635 | 3.585601 | -1.59003 | 2.42E-53 | 8.69E-53 |
| IGLV2-23 | 3.838381 | 5.569669 | 1.731288 | 4.15E-14 | 5.82E-14 |
| UPF3A | 4.239518 | 2.851794 | -1.38772 | 2.50E-57 | 1.11E-56 |
| FAM3A | 4.150967 | 3.148718 | -1.00225 | 3.48E-61 | 2.04E-60 |
| ZNRF2 | 2.168492 | 3.518777 | 1.350285 | 5.24E-63 | 3.62E-62 |
| MMEL1 | 2.229498 | 0.562588 | -1.66691 | 9.29E-53 | 3.25E-52 |
| RPLP0P6 | 2.90639 | 5.005659 | 2.09927 | 1.93E-65 | 1.72E-64 |
| CCDC18-AS1 | 3.035894 | 1.293731 | -1.74216 | 2.96E-57 | 1.30E-56 |
| AC024995.1 | 0.318081 | 2.334867 | 2.016786 | 1.89E-68 | 2.38E-67 |
| U2AF1L5 | 3.477312 | 0.101349 | -3.37596 | 1.56E-66 | 1.55E-65 |
| PLEC | 4.415913 | 6.209279 | 1.793366 | 4.39E-63 | 3.06E-62 |
| COX6C | 6.769369 | 5.470756 | -1.29861 | 6.47E-51 | 2.10E-50 |
| STK17B | 1.975504 | 3.142393 | 1.166889 | 5.38E-54 | 1.99E-53 |
| ATPAF2 | 2.95648 | 1.819045 | -1.13743 | 2.20E-66 | 2.17E-65 |
| TIMP1 | 6.004312 | 8.694027 | 2.689715 | 8.57E-75 | 2.93E-73 |
| CNDP2 | 5.044891 | 3.76192 | -1.28297 | 2.90E-42 | 7.23E-42 |
| DDX47 | 3.47729 | 0.684337 | -2.79295 | 3.33E-50 | 1.05E-49 |
