# Supplementary figures and images for: Key Roles of p53 Signaling Pathway-Related Factors GADD45B and SERPINE1 in the Occurrence and Development of Gastric Cancer
Source: Mediators Inflamm. 2023 Aug 24;2023:6368893. doi: 10.1155/2023/6368893 (PMC10471451; doi:10.1155/2023/6368893)

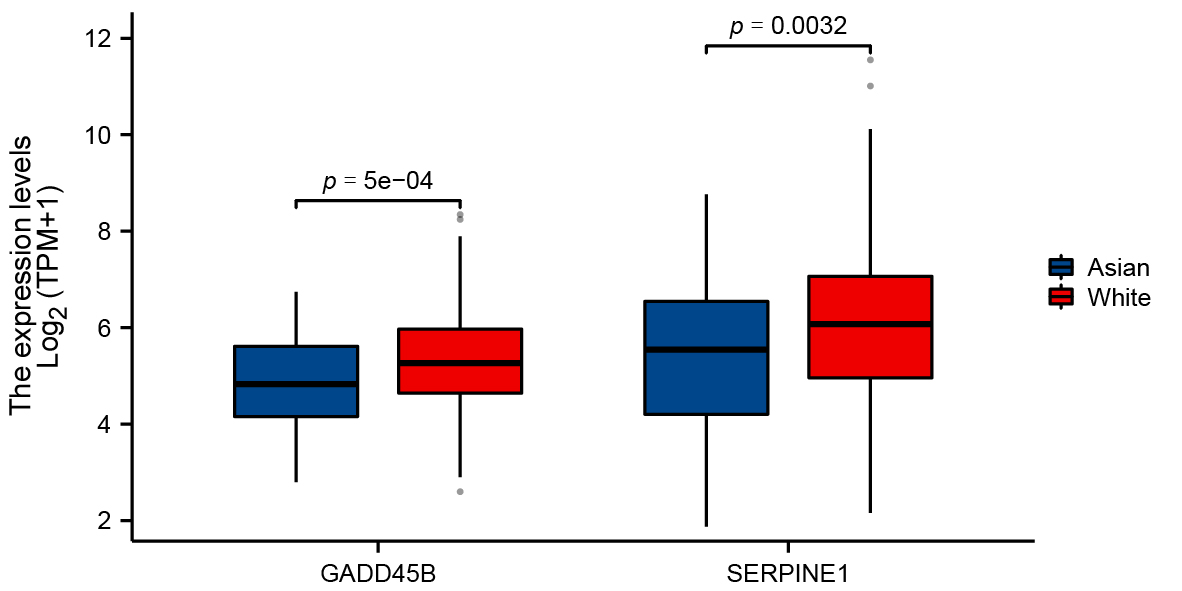

Supplement: Supplementary 2 — The boxplot of GADD45B and SERPINE1 expression in gastric cancer samples of different races (Asian and White) in the TCGA_STAD dataset. [file 6368893.f2.jpg]
